# Supplementary material for: Understanding implementation of a complex intervention in a stroke rehabilitation research trial: A qualitative evaluation using Normalisation Process Theory
Source: PLoS One. 2023 Sep 8;18(9):e0282612. doi: 10.1371/journal.pone.0282612 (PMC10490858; doi:10.1371/journal.pone.0282612)
Supplement: S1 File — (PDF) [file pone.0282612.s001.pdf]

IMPS
Implicit Learning in Stroke

INTERVENTION GUIDANCE

Chief Investigator: Dr Louise Johnson

Contact Details: louise.johnson@rbch.nhs.uk;

REC reference: 18/SC/0582
IRAS project ID: 250540

Contents

1.0 About the IMPS Trial 3

2.0 The Implicit Learning Approach 4

What is Implicit Learning? 4

3.0 Frequency of Coaching Statements 6

4.0 Focus of Attention 7

Ideas for how to promote an External Focus of Attention 8

5.0 Exercise Examples 9

6.0 Summary 14

7.0 Using the Video Recorder 15

8.0 References: 15

1.0 About the IMPS Trial

This document contains guidance on the Implicit Learning Approach (ILA). Your service has been randomised to the ***intervention*** arm of the IMPS Trial. This guidance, alongside the training package, will help you to adopt an implicit learning approach for participants enrolled in the study.

For participants who are enrolled in the study, we are asking you to use the ILA for **all** rehabilitation sessions that focus on sitting, sit to stand, stepping, transfers and gait. This is from the point of enrolment, until discharge from hospital. It is important that participants in the trial receive rehabilitation that is **strongly biased** towards the ILA. To do this, you need to adopt coaching behaviours that will promote implicit learning processes.

The guidance outlines the principles of implicit learning, and provides a series of examples for how common exercises could be adapted to bias the implicit system. You can use these exercises and/or adapt them for the individual. You can also create your own exercises; as long as they follow they remain true to the ILA principles, which are to:

- Reduce the quantity of verbal coaching statements that you give (instructions and feedback) and;
- Promote an external focus of attention as much as possible.

Further information about the study design and process can be found in the IMPS Protocol.

If you need help or guidance at any time, you are very welcome to contact your local Principal Investigator [add name], or the Chief Investigator – louise.johnson@uhd.nhs.uk

2.0 The Implicit Learning Approach

What is Implicit Learning?

Implicit learning occurs when the performer (in this case, the patient), does not gain verbal knowledge of movement performance (e.g. facts and rules about *how* to move) (1). It is often describe as *learning without awareness*.

In contrast, someone who is learning *explicitly* will be able to describe how they are moving. They will know and be able to verbalise facts or rules, such as:

> "I am not controlling my ***knee*** very well"
>
> "I know that I need to focus on picking up my ***foot*** and putting my ***heel*** down first".

During *implicit learning,* the performer does not accumulate these types of facts or rules. They cannot easily describe **how** they are moving or what they need to do differently, but they might be able to **show you**. It is a more automatic and subconscious form of learning.

To promote implicit learning, we are providing guidance on how you can alter the delivery of rehabilitation, by changing elements relating to:

- Instructions (timing and type)
- Feedback (timing and type)
- Task organisation (including focus of attention)

As this is a clinically grounded trial, you have the freedom to tailor the specific content of each treatment session to patient need. You will need to adapt your approach depending on the skills and ability of the patient. However, you must remain true to the principles of the ILA. The aim is to create a **strong bias** toward implicit learning processes.

We have provided examples for how common rehabilitation exercises can be adapted to incorporate these principles. Adapting exercises in this way is more likely to promote implicit learning. The principles are summarised in Table 1.

	Bias<br>Explicit ←--→ Implicit	
	**Control Group**<br>Standard Care*	**Experimental Group**<br>Implicit Learning Approach
**Quantity and Frequency of Coaching Statements (Instructions and Feedback)**	**HIGH**<br>• Instructions should be frequent, and given at the beginning and throughout the task (i.e during practice)<br>• Feedback should be given during task practice – at least once for every 5 repetitions of any given task	**LOW**<br>• Instructions should be limited, and only given at the beginning of task practice<br>• Use demonstration to highlight the desired movement<br>• Feedback or further instruction avoided throughout task practice
**Focus of Attention**	**INTERNAL**<br>• Direct attention to the action itself.<br>• Include the rules or steps that need to be followed – break it down.<br>• Use the term "focus on [a reference within the body]" or "think about [a reference within the body]"<br><br>Example: *"Bend your knee and lift your foot onto the block. Focus on your leg"*	**EXTERNAL**<br>• Direct attention to the effects of the action.<br>• Specify the task/goal, but avoid the use of rules or steps that need to be followed.<br>• Use references in the environment to achieve the desired movement.<br><br>Example: *"Step onto the marker on the block."*
**Task Set Up**	No specific guidance given.	Activity and environment set up to facilitate a focus on the environment – through use of external reference points, markers, audio feedback.
**Organisation of Task**	No specific guidance given.	Where possible, incorporate **whole task practice.**

* Guidance for standard care is based on published observational studies describing usual practice in stroke rehabilitation [1, 10, 11].

Table 1: Overview of Intervention

3.0 Frequency of Coaching Statements

The term coaching statements refers to verbal instructions, feedback and motivational statements (e.g. good, keep going, and another). To encourage implicit learning, the **quantity and frequency** of coaching statements should be **limited.**

- Instructions should be given at the beginning of task practice; their focus should be external (see below)
- Feedback should be given at the end of a series of repetitions and/or delayed; it's focus should be external (see below)
- Concurrent instructions and feedback (i.e. given during task practice) should be avoided.

In addition, the **content should be simple.**

- Use as **few words** as possible to convey the message.
- Only give **1-2 pieces of information** at a time.
- Try to use **positive** instructions – tell someone what to do, rather than what not to do.
- Use **demonstration** to reinforce the desired movement pattern and minimise the need for additional verbal instructions.

4.0 Focus of Attention

The term focus of attention refers to the location of an individual's attention in relation to the performance environment/task. It can be either internal or external.

- ➔ An **internal focus** is directed toward components of the body movement, where the learner will be consciously aware of *how* they are performing.
- ➔ An **external focus** is directed toward the effect of the movement on the environment, or the end goal.

To promote implicit learning, an **external focus of attention should be used**. To achieve this, the mention of body parts should be minimised.

- The task and the instruction should always focus on the outcome of the targeted movement to maintain a bias toward an external focus (e.g. tap the *marker*).
- The movement required to achieve an outcome should not be mentioned (e.g. do not say - *lift your* foot; or *bend your* hip).
- When required for clarity, the body part itself can be mentioned (e.g. tap your *foot* onto the *marker*), but efforts should be made to minimise this.

In summary, to promote implicit learning:

- **SAY LESS!** Use fewer instructions and feedback and;
- **KEEP THE FOCUS EXTERNAL**. You might need to adjust the task, to facilitate this.

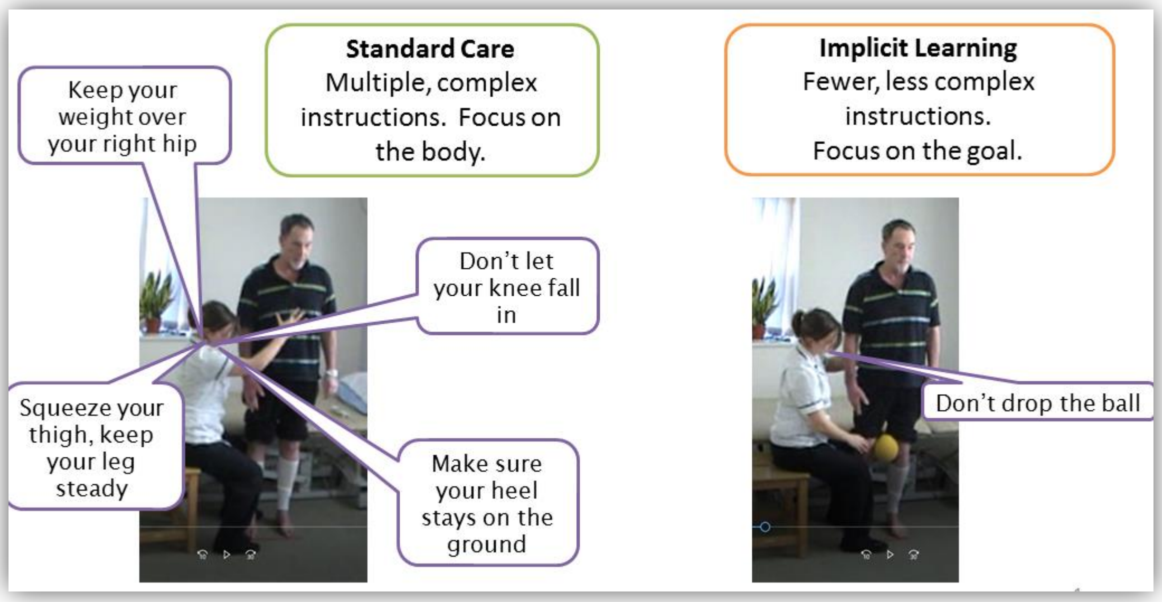

Ideas for how to promote an External Focus of Attention

To promote an external focus of attention, you may need to do more than just change your instruction. You may also need to think about what the exercise/task is specifically aiming to achieve, and then alter the set-up to facilitate this. The table below includes ideas for promoting an external focus of attention:

	Concept	Example instruction
**SOUND** 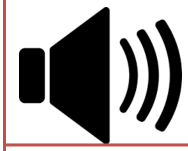	• Use a buzzer or squeaker to provide an externally focussed target	*"Touch the buzzer like this"* [demonstrate hitting a buzzer with your heel]
**MARKER or TARGET** 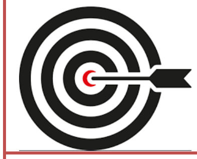	• Use tape to highlight desired positon (e.g. of the feet)<br>• Include a target to aim for, or a reference point to keep to	*"Keep your feet/shoes behind the line"*<br><br>*"Keep within the lines as you step"*<br><br>*"Keep against that marker [with your hip]"*
**OBJECT** 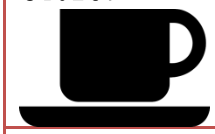	• Reaching for an object<br>• Tapping a cup<br>• Stepping over a beanbag	*"Touch the red dot"*<br><br>*"Tap the cup without crushing it"*<br>*"Step over the beanbag"*
**TIME** 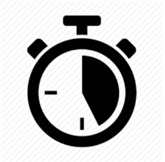	• Trying to achieve a certain number of repetitions within a certain time<br>• Tying to complete a task in a faster time	*"See how many times you can [add task] in 30 seconds"* [ideally have clock or countdown app visible]<br><br>*"Hold it against the marker for 5 seconds"*
**DISTANCE** 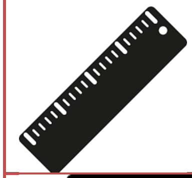	• Moving over a greater distance (e.g. reach, foot placement)<br>• Use tram lines on the floor/wall	*"See how many lines you can cross"*<br><br>*"Reach as far as the blue line"*
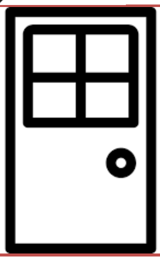	•	

5.0 Exercise Examples

IMPS 1

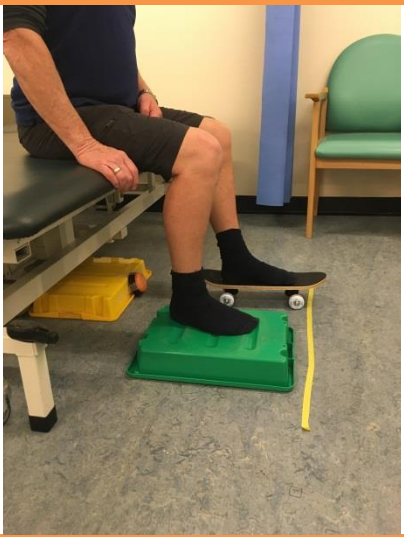

Instruction:	*Hit the buzzer with the back of the skateboard*
Notes:	For eliciting knee flexion and ankle DF - required for adequate foot placement prior to STS
Set Up:	Target line in front; buzzer behind. Non-affected foot on block for symmetry/posture.
Progression:	Add a weight to the skateboard for strengthening
Regression:	Bring the buzzer closer to enable success

IMPS 2

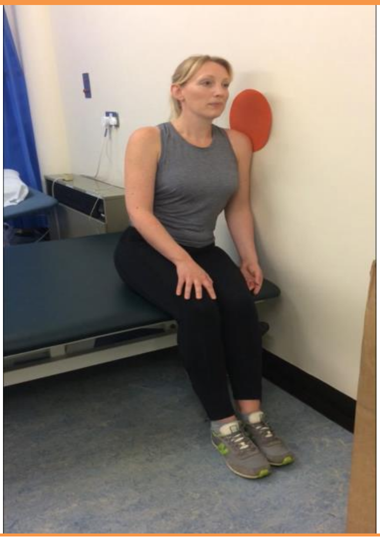

Instruction:	*Keep against the red spot/wall.*
Notes:	For eliciting right leg activity in sitting (pushing with right leg towards the wall).
Set Up:	Marker on wall, and marker placed on shoulder (e.g. tape). Ensure the patient is pushing through the hemiplegic leg.
Progression:	Hold it there for 5 seconds.
Regression:	Sit closer to the wall<br><br>NB – RIGHT HEMIPLEGIA IN GTHIS EXAMPLE

IMPS 3		
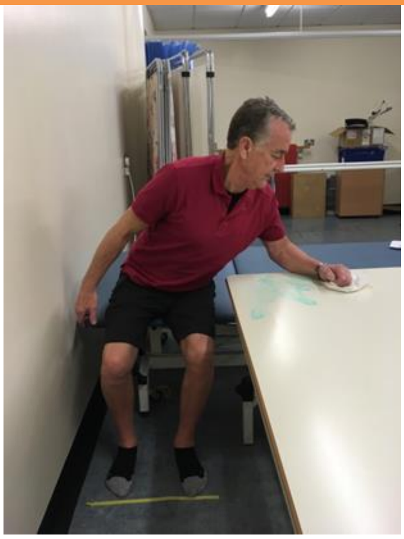	Instruction:	Clean the dots off the table, and bring yourself back to the wall
	Notes:	Loading the affected leg in sitting – aim is to bring weight over the affected leg, and then use the leg to push back to the wall
	Set Up:	Draw arcs of dots on table with whiteboard pen. Line for foot position. Close to wall on affected side.
	Progression:	Vary the position of the reach; reach for objects if UL function allows.
	Regression:	

IMPS 4		
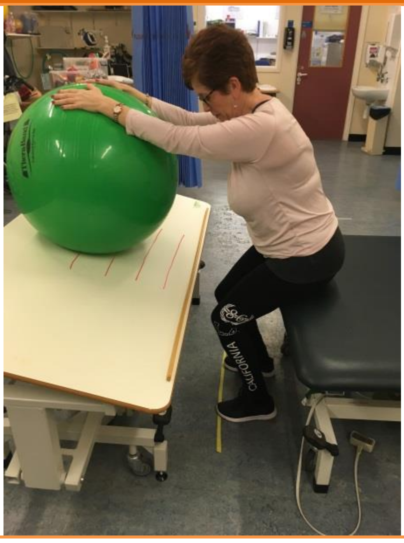	Instruction:	*Roll the ball forwards until you can see three coloured lines*
	Notes:	Designed to encourage forward weight transfer. Make sure feet are well placed and the trunk is extending.
	Set Up:	Table slanted to promote extension.
	Progression:	Increase the speed or distance. Move into part stand.
	Regression:	

IMPS 5

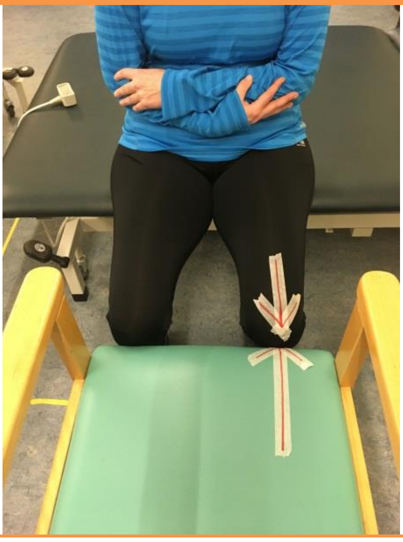

Instruction:	Stand up, keeping the arrows pointing to each other.
Notes:	Sitting with the unaffected side close to the wall, markers on the floor indicate desired foot placement.
Set Up:	Tape arrows on thigh and on a stool or chair in front. Line of tape on floor to indicate foot position.
Progression:	Move into full sit to stand; control on stand to sit as well
Regression:	Raise the height of the plinth, and initiate sit to stand

IMPS 6

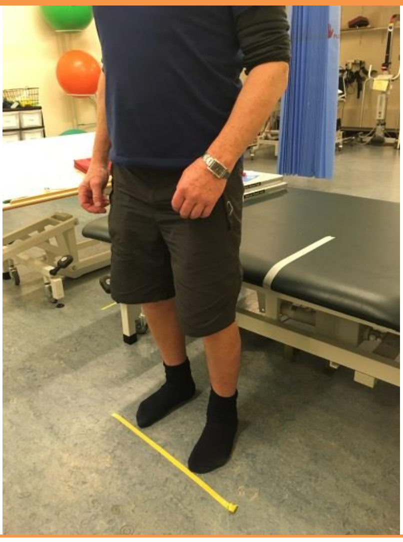

Instruction:	*Stand up, keeping your feet behind the line. Push through the floor on your [affected side]*
Notes:	Whole task practice, encouraging increased WB on affected side.
Progression:	Lower height of plinth.
Regression:	Raise height of plinth. Position feet in step stance with affected leg behind.

IMPS 7

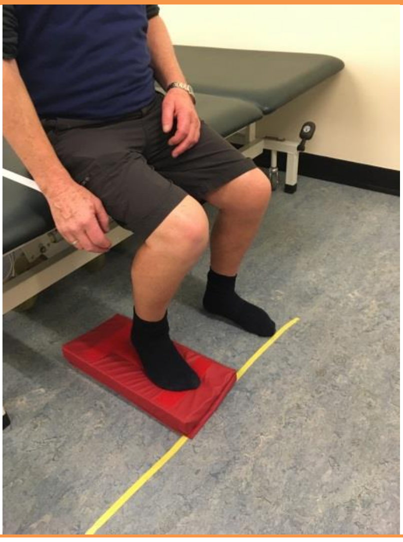

Instruction:	*Stand up, pushing through the floor on your [affected side]*
Notes:	Whole task practice, encouraging increased WB on affected side.
Set Up:	Sit to stand with non-affected leg in raised or unstable surface, to challenge postural control and increase weight on left.
Progression:	Increase height of object under foot, or reduce stability of object.
Regression:	

IMPS 8

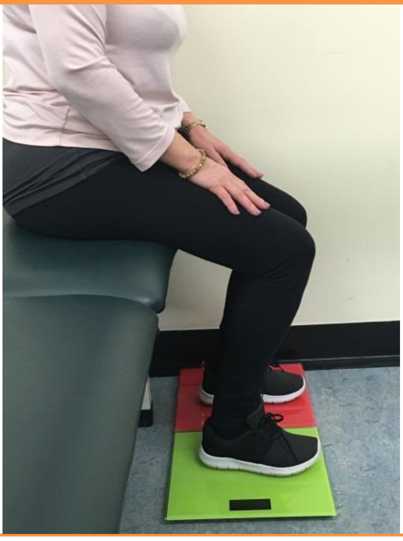

Instruction:	Push xx kg through the scales as you stand.
Notes:	Sit to stand with weighing scales under feet. Aim to increase weight through affected leg.
Set Up:	Therapist gives feedback on numbers, to avoid person looking down.
Progression:	Increase desired weight going through affected leg, until 50:50
Regression:	Same activity, but in sitting – or just into a bottom lift.

IMPS 9

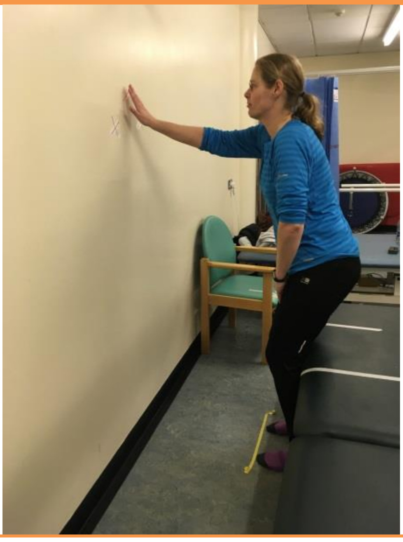

Instruction:	Reach for the red cross as you stand
Notes:	STS and touch coloured markers for whole task practice.
Set Up:	Tape on floor to ensure adequate foot position; tape on plinth to show desired sitting position.
Progression:	Vary position of dots. Increase speed.
Regression:	Raise plinth to perch position.

IMPS 10

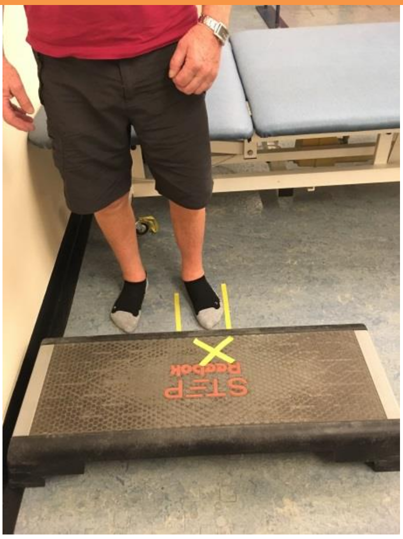

Instruction:	Touch the yellow cross with your heel, and then back between the lines<br>[or “touch the yellow cross like this (add demo)”]
Notes:	Stepping with affected leg onto an external marker on a step
Set Up:	Marker on step, and for foot position on floor.
Progression:	Vary the position of a few markers.<br>Start with foot behind (hip extension) to mimic walking.
Regression:	Lower height of step or put the cross on floor.

IMPS 11

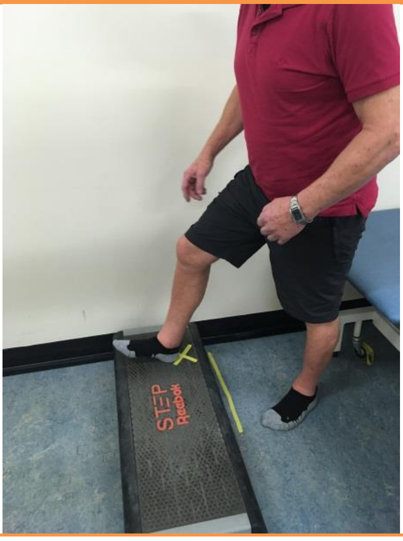

Instruction:	Touch the yellow cross with your heel, and then back between the lines<br>[or touch the yellow cross like this (add demo)]
Notes:	Stepping with unaffected leg onto an external marker, to increase loading of affected leg
Progression:	Hold it there for 5 seconds.<br>Tap a plastic cup, and try not to squash it.
Regression:	Toe or heel lifts with non-affected foot.

IMPS 12

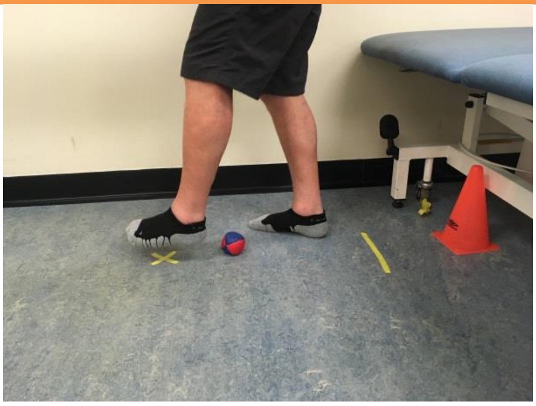

Instruction:	Step over the ball, and back again – touch the lines.
Notes:	Stepping over an object to practice swing phase.
Set Up:	Line and cone behind to promote a starting position in extension.
Progression:	Change height or position of objects for variability.<br>Move into extension prior to step.
Regression:	Step over a line on the floor.

6.0 Summary

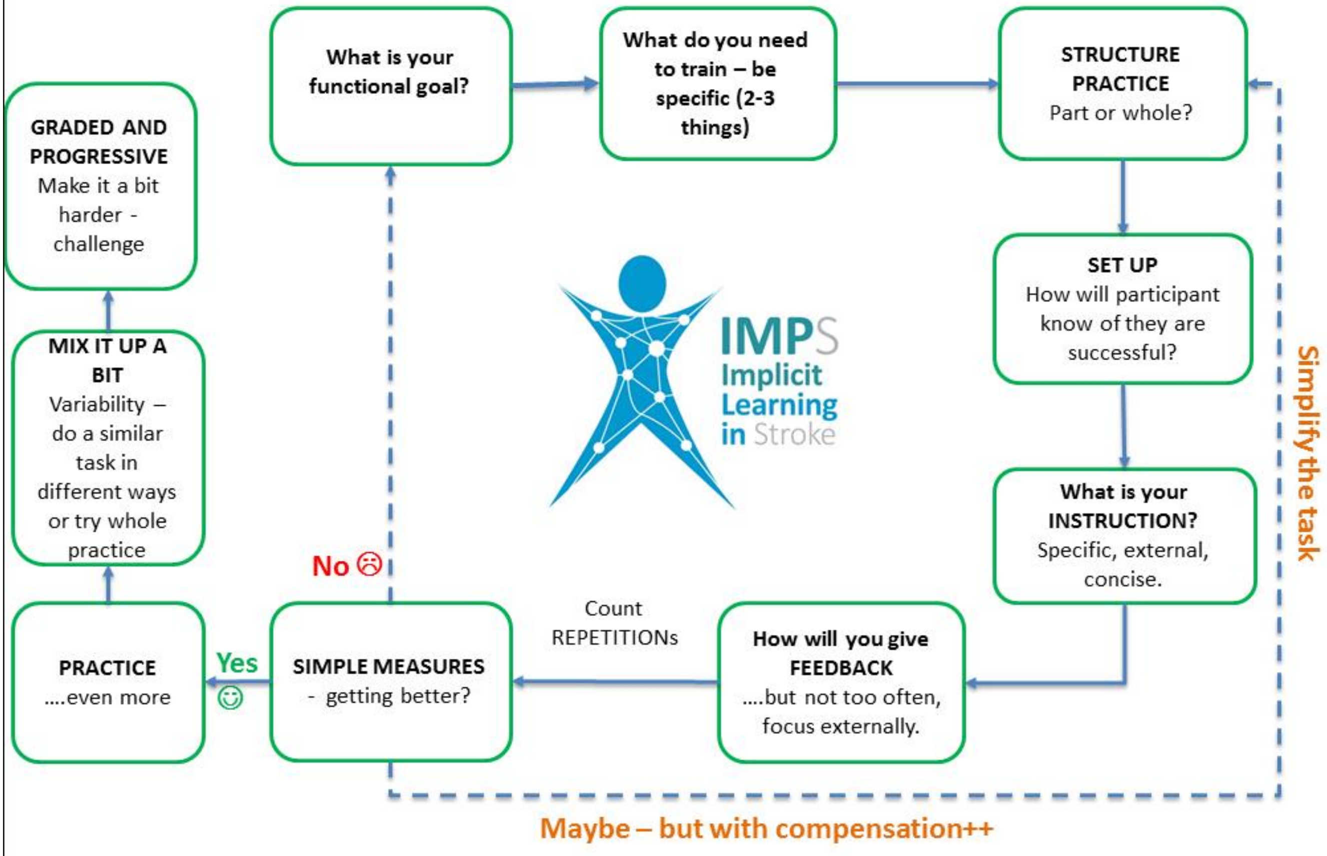
What is your functional goal?
What do you need to train – be specific (2-3 things)
STRUCTURE PRACTICE
Part or whole?
SET UP
How will participant know of they are successful?
What is your INSTRUCTION?
Specific, external, concise.
How will you give FEEDBACK
....but not too often, focus externally.
Count REPETITIONs
SIMPLE MEASURES
- getting better?
No ☹
Yes ☺
PRACTICE
....even more
MIX IT UP A BIT
Variability – do a similar task in different ways or try whole practice
GRADED AND PROGRESSIVE
Make it a bit harder - challenge
IMPS
Implicit Learning in Stroke
Simplify the task
Maybe – but with compensation++

7.0 Using the Video Recorder

Wherever possible, every treatment session should be video recorded. This will help us to understand how easily you have been able to apply this treatment guidance.

When recording treatment sessions:

1. Gain the participants **verbal consent** every time, before starting any recording. You must not record without the participant being aware that the camera is on. You can stop recording at any time if you, or the participant, wishes to.
2. Ensure that patient dignity is maintained – avoid asking the participant to undress.
3. Be aware of the environment – the video camera will pick up sound and images from others who are not part of the study, so if possible, avoid treating in a communal space and make other people aware that recording is taking place.
4. Record the **whole treatment session**, from start to finish. However, you do not need to record any preparation at the beginning, for example if you are hoisting the person from their chair to the plinth.
5. Check there is sufficient battery (or plug in) and sufficient storage on the memory card, to capture the full session.
6. Place the camera in a position where it can capture most of the session – check the frame before starting.
7. Make sure that the camera is recording – green light is on.
8. Start the recording by stating:
    a. Participant ID
    b. Date
    c. Time
    d. Treatment session or assessment session (state week of assessment)

8.0 References:

1. Kleynen M, Braun SM, Bleijlevens MH, Lexis MA, Rasquin SM, Halfens J, et al. Using a Delphi Technique to Seek Consensus Regarding Definitions, Descriptions and Classification of Terms Related to Implicit and Explicit Forms of Motor Learning. PLOS ONE. 2014;9(6):e100227.
