# Supplementary material for: Understanding implementation of a complex intervention in a stroke rehabilitation research trial: A qualitative evaluation using Normalisation Process Theory
Source: PLoS One. 2023 Sep 8;18(9):e0282612. doi: 10.1371/journal.pone.0282612 (PMC10490858; doi:10.1371/journal.pone.0282612)
Supplement: S2 File — (DOCX) [file pone.0282612.s002.docx]

**IMPS – Implicit Learning in Stroke Study**

**Focus Group Topic Guide (Clinicians)**

| **NPT Construct** | **Question** | **Prompts** |
| --- | --- | --- |
| **A** | **Describe what you thought when you first heard about this research trial?** | What did you think when you heard about the ILA?  How much did you already know about implicit learning?  Did your views/knowledge change when you attended the training? How did you feel about that?  Do you think others shared this view? What was the view of the Unit? What is the view of our professions – more widely? |
|  | **How did you feel it [the ILA] related to current practice?** | What are the differences?  Can you give examples?  Can you be specific?  Is this something you had thought about before? |
| **B** | **Tell me about your experience of integrating the ILA into your practice?** | How easy was it?  Has this changed over time?  What has led to this change?  Were there any circumstances in which it was particularly difficult [type of patient; other impairments etc]  Specifically, what was hard?  Were there any circumstances where it seemed easier? A better fit? |
| **B** | **Do you have any thoughts on the relative benefits of an ILA?** | Did you notice any differences in how patients responded to the ILA, compared to your normal approach? |
| **B** | **What do you think about the application of the ILA in practice?** | Is it important to consider our instructions and feedback?  What makes you think this?  What informs this view? |
| **B** | **Is there anything about the approach which you feel is unclear?** | Can you comment on the training you received?  What about the written manual?  How easy was it to put this into practice? What made it easy/not easy? |
| **C** | **If the future study demonstrated that the ILA was beneficial, what would need to happen to get the approach widely adopted into practice?**  **(e.g. more knowledge about how to do it; more evidence that it is of benefit; colleagues around me who think it is important; evidence that patients think it is important)** | What would lead you to change your practice in the future?  What stops you from changing your practice?  What support do you think clinicians would need?  What type of training is helpful?  Is there anything that you found particularly helpful as a result of your involvement?  What do you think the challenges would be? How could we overcome these? |
| **C** | **How would you know if the ILA was benefitting a patient, in practice?** | Can you give any examples where you thought the principles of the ILA were particularly helpful? What happened? What made you think it was beneficial?  What would you look out for, to tell you that it wasn’t working? |
| **C** | **In terms of motor recovery, what information do you collect to determine how effective your interventions are?** | What works?  What doesn’t work? How easy is it to collect this?  What do you do with the information?  Are these measures that you would want to see in a future research trial?  Is there anything else that you think should be monitored? |
| **D** | **Throughout your involvement in the study, has the ILA been something you’ve talked about as a team?**  **I ask this to get an understanding of the sorts of issues that may have been raised – tell me in general terms, without naming individuals.** | Has it been a topic of informal conversation – in the office or on the ward? What sorts of things have been discussed? Were there any common aspects that people have chatted about? |
| **D** | **Having discussed the ILA today as a group, have your views changed at all?** | Have your colleagues expressed views that surprised you, or were very different to yours?  Has this opportunity for reflection made you think differently? Can you give a specific example?  How important is it that the whole team buy in to a certain intervention?  Do you feel everyone works in the same way, or are therapists very individual? How does this relate to the ILA? |
|  | **Is there anything that we haven’t discussed, that you would like to talk about?** |  |

**Normalisation Process Theory Constructs**

**A = coherance**

**B= cognitive participation**

**C = collective action**

**D = reflective monitoring**
