# Supplementary material for: Understanding implementation of a complex intervention in a stroke rehabilitation research trial: A qualitative evaluation using Normalisation Process Theory
Source: PLoS One. 2023 Sep 8;18(9):e0282612. doi: 10.1371/journal.pone.0282612 (PMC10490858; doi:10.1371/journal.pone.0282612)
Supplement: S3 File — (DOC) [file pone.0282612.s003.doc]

**Interview Topic Guide: Post Intervention Interviews with Patient Participants**

**Purpose:**

- To understand patient perceptions of the ILA (compared to standard care)
- To identify if there are any differences in the experience of those receiving the ILA, versus standard care (e.g. motivation)
- To determine the extent to which participants were aware of what was being learnt during their treatment sessions.

**Interview Guide:**

I’d like to ask you some questions about the rehabilitation you have been receiving whilst you have been in hospital. I am particularly interested in hearing about the therapy which has focussed on recovery of movement in your leg, and activities such as standing, stepping, walking.

| **Question** | **Prompt** |
| --- | --- |
| Can you tell me about what you have been doing in your therapy sessions? | What have you mainly been working towards in your therapy sessions? [goals]  Can you give some examples of the exercises that you have been practising?  Is there anything in particular that you have been focusing on?  Is there anything that has been particularly difficult for you? Any types of exercises or particular activities? What made it difficult? |
| You said that you have been working towards [insert activity]. When you have been practicing that, what do you think about?  *[understanding patient held “rules”]* | Is there anything about the movement that you focus on?  Do you use any other techniques to help your performance? |
| Think about how your therapist(s) worked with you during those therapy sessions. How would you describe their approach? | How did you feel?  Is there anything that you would have liked to be different?  Is there anything about how they talked to you, which you felt was good?  Is there anything about how they talked to you, which you would have preferred to be different?  Did you see more than one therapist during your stay? Were they similar in their approach/or different? What did you notice that was similar/different? |
| Thinking about the way they gave you instructions – was it always clear what they wanted you to do? | What made it clear/not clear – can you give an example? |
| How did you know if you were doing the movement in the right way? | Did your therapist give you feedback?  How did they do this? Was it helpful? Can you give an example?  Would you have preferred something different/more/less? |
| Have you been practicing exercises outside of therapy? | Tell me about the type of exercises you have been doing…  How have you found this?  What do you think about when you are practicing outside of therapy? What do you focus on? Is this different when the therapist isn’t there?  How have you known if you were doing them in the right way?  Have you used any techniques to get feedback when the therapist isn’t there? |
| How do you feel about that the progress you have made whilst here on the Stroke Unit? | What makes you feel that way? |
| What words would you use to describe your therapy sessions? | If you were telling your family about your therapy session, how would you describe it?  How have you felt during those sessions?  How have you felt after those sessions? |
| Is there anything else about your therapy that you would like to tell me about? |  |
