# Supplementary material for: Understanding implementation of a complex intervention in a stroke rehabilitation research trial: A qualitative evaluation using Normalisation Process Theory
Source: PLoS One. 2023 Sep 8;18(9):e0282612. doi: 10.1371/journal.pone.0282612 (PMC10490858; doi:10.1371/journal.pone.0282612)
Supplement: S1 Data — (ZIP) [file pone.0282612.s004.zip › Supporting Information - Data/Focus Group Transcripts.docx]

**FOCUS GROUP 7**

**I: What I will get you to do if it’s OK I know we’ve introduced each other but just to get you to introduce yourself. It’s more for the purpose of the tape so the lady who transcribes can just try and pick out voices. So if you are happy just to say your name and just what your role is here and we’ll just go round to start with that would be fab.**

MP1: My name is [name] and I’m a recently qualified physiotherapist on the stroke ward.

FP1: My name is [name] and I am a band 6 physiotherapist joining from July and I’m from India.

FP2: I’m [name] and I’m a physio assistant on the stroke unit, band 3.

FP3: I’m [name] a physio assistant on the stroke unit.

MP2: [Name] a team leader physiotherapist in stroke.

**I: Brilliant, thank you. So we’re just going to kick off, so we’ll start obviously we’re talking about the IMPS study which some of you have been involved in and some have not been involved in, which is absolutely fine. I’ll say for the purpose of the tape we’ve recruited a couple of participants here about a year ago. From what I remember they were both quite dependent patients weren’t they. MP2 is nodding.**

MP2: Yes.

**I: And those of you that were here were you all involved in a seeing those patients? Yes so FP3 was. Were you involved at all?**

FP2: I don’t think I was.

MP2: You did at least one with us.

FP2: One session.

MP2: Yes a couple of sessions but that was, it was mainly me and FP3 at that time.

**I: OK that’s fine. And you guys are new so obviously you’ve not been seeing patients. So we’ve got a mixture of people in the room and you’ll be answering therefore from slightly different perspectives which is absolutely fine and both are really helpful. So for those that were more involved and for who we did the training which actually the training was back just before we went into first lockdown I think wasn’t it. So there was quite a gap and then we did some refresher training.**

MP2: Yes.

**I: Thinking back to then when we first started talking about the study and what we were looking at can you recall what your first thoughts were?**

MP2: Yes from my perspective I was kind of encouraged and interested in the study itself for the fact that I’ve just in terms of fairly recently doing normal movement science approaches, done a bit of MSc in movement science and I wanted to try and put some more into practice and also support the recent research evidence based. So that’s kind of interesting. I thought this would be a useful way of doing it. I thought this team would be able to recruit sufficient numbers which weren’t that many. I think when we talked about it we talked about potentially having seven would be our target. But yes I was kind of enthused about participating in a bit more research again.

**I: With regards to what the research was looking at more specifically the kind of concepts of different ways of learning implicit and explicit learning did you have any thoughts on that?**

MP2: I think like with lots of things like physio tends to be very much evidence based and you kind of think, oh OK what’s the new bit of research doing, oh well we should be trying to do this, this and this. Obviously by doing the MSc module I have started incorporating that into our general physio practice more so than we used to do and then onto that with all the kind of recent CPD that’s been available again driving more movement science approach again we’ve been utilising that pre-this research. Yes so I suppose it didn’t really necessarily change our way of working, I think the only things I found slightly more different were having to do the assessment outcome measures that I wouldn’t normally do. But in terms of the practical, the intervention aspects of it, it’s been quite common for us to draw lines and put targets out. And I think from my perspective just making sure that the team minimised the amount of verbal feedback we give, really emphasising that much more so.

**I: So it felt like it was kind of in line with the way you would describe yourself working and the team working.**

MP2: Yes as a team here we’ve been doing that for quite a while and we’ve had various other therapists that we worked with that have also been doing that as well. Then also the OTs have also come on board to doing movement science as well so again it has been much more of an easier approach to do because it’s not necessarily we’re not doing that much different.

**I: FP3 and FP2 do you feel the same from your perspective? I know it was ages ago we did that training do you remember did you think, oh this is going to be really hard and different?**

FP3: I was quite excited to try something new. I was a bit concerned about not saying the body parts and to tell myself to keep quiet a bit for the feedback. But I enjoyed using the different things you could find and the buzzers, the patients reaching and we had some positive feedback from that didn’t we.

MP2: Yes.

FP2: Yes the less verbal you had to think, don’t say too much about moving body parts and things.

**I: So that bit of it did feel like it would be a little bit different.**

FP2: Yes but better for the patient in the sense that they’ve got to think more and how they’re going to move and what they’re going to move. Puts the emphasis on them more than us giving them instructions all the time.

FP3: Not overloading them with too much technical terms like the weight shift and things like that just aim for the red mark.

FP2: On the floor, yes.

FP3: We did change didn’t we the approaches.

FP2: Yes definitely.

**I: So to recap for you guys frantically reading the presentation. So what we were looking at in the study is, I’ll explain it, so it was cluster randomised so there were eight stroke units that have taken part and the stroke unit is randomised. So four were just control they carry on as normal but they recruit people and we measure their progress and then four including here were randomised to the intervention. What that meant was for people who were enrolled in the study we did some training, it was about an hour, hour and a bit, so fairly brief training with the team and provided some guidance and we were asking them to use some principles of what we call implicit learning. So supporting people in their physical recovery to learn in what we call an implicit way. The way that we would do that is by asking the therapists, there are different bits but the main things are to not give too much verbal instruction and feedback, so to not overload patients with lots of specific instruction about what we want them to do like say I want you to bend your knee or straighten your knee or shift your weight onto your left leg. Avoiding all of that information about the body and trying to set them up to practice so that they achieve all of those things but without thinking about it necessarily and when we do that we’re learning more implicitly rather than explicitly. So reducing how much we say, so not to say nothing but to really try and cut that down and to use instructions and feedback that focus on something external to the body so a target or a goal. So maybe setting up tasks slightly differently so that the person is thinking about achieving the task to get the movement rather than thinking about the movement. They were really the two main things. We were videoing the treatment sessions to see how easy or not it was for therapists to put those principles into practice really. We know there’s probably a variation in how we all work but observational studies have shown us that on the whole therapist do tend to talk quite a lot when we’re working with people and we tend to give them quite a lot of instructions and to correct them when they don’t do something quite quickly we correct them. So patients need to process all of that whilst they’re doing and we are trying to look at slightly different approach, which some people might work in that way a little bit already. So it would just be interesting to know based on that very brief introduction to the topic just whether you have any immediate thoughts on that, on how easy or not you might find that to do or whether actually you think in your practice you don’t give lots of instructions or you don’t use an internal focus.**

MP1: I think from a therapists point of view it is sometimes quite hard to keep the task more functional and aiming for external objects and things rather than saying, it’s easy for us to say lift your leg up because that’s the initial task that we want them to do, we want them to lift their leg up but in actual fact there's another reason why we want them to do that. So sometimes it’s quite hard for us to say that because what comes naturally for us is to want that initial task for them to do that. But I think from a patient point of view it’s obviously a lot easier for them to take into consideration what we want them to do if we’re saying reach for this, reach for that rather than saying lift your arm up. It’s a lot easier for the patient to take into consideration but I think from a therapist point of view I definitely think sometimes I speak in a little bit too much jargon basically and it might be that the patients don’t understand me as much, you’re not getting the better results. I definitely think keeping it more functional like that would be a better approach.

FP1: I also think that giving a patient a target will also help us to see how can they plan the activity after a stroke which is also a very big part of our assessment. So it might help from a therapist’s point of view to know how the patient is planning the activity but if the patient is cognitively affected then I think that could be a challenge to ask the patient to go for a target. Also for giving one word commands to a patient who is critically affected might find it difficult so in that way I think less commands is right but it’s quite difficult and challenging to me with a patient who is quite at a critical level who has a stroke. So functional activities I like functional activities as well so that the patient is aware of what he’s doing rather than telling lift the leg. If we say that you have to reach there that’s what they are going to do after they recover from stroke so that would help them in their recovery as well. But I think the real challenge would be as I said with the critical patients who cannot follow commands or who are literally not following what you are saying. It’s quite difficult with them to manage with less commands. It makes them understand what they are supposed to do or what we want them to do.

**I: Yes so patients whose language has been affected or cognition. Yes. So in your experience to anybody what do you feel works best for those types of patients in terms of learning styles?**

FP1: For me I think showing them if I am doing it and if they can see and their vision is alright and they can follow at least what I am doing and then ask them to imitate it or something would help more than giving any commands or asking them to do something. So I think first if I do it and then ask them to just follow then I think that would be a better approach rather than just asking them to reach for a buzzer or just say hold the cup or something. Or just if the patient is not following commands but the patient might do everyday activities like you know how to drink just give them a drink and see how they are doing. That would be a non-verbal command thing which we can assess because patient might know what to do and just follow it from the previous memory that they have.

**I: So using demonstration.**

FP1: Use demonstration.

**I: Yes it’s another way actually of learning implicitly using demonstration. It depends whether the demonstration comes with a lot of verbal instruction or not so it could be either. Do others have thoughts on that so working with people, I don’t know if the people you involved had cognition problems?**

MP2: Both those sets of patients were quite, they were more on the heavily dependent side than you would ideally want in a research intervention arm personally but that’s in terms of fitting in with the criteria of your inclusion criteria they still fitted into that but it was slightly more difficult in terms of one of them having language impairment. So again there was lots of demonstration, a lot of demonstration and repetition of that demonstration to get them to understand that task. But again choosing an activity that they should be able to understand instead of reaching for targets or reaching out for random areas we then gave them specific targets or cups or drinks and things like that that they were able to not have to think about they just that’s obviously what you want me to do OK.

**I: Automatic.**

MP2: Yes and they improved with the activity level there. But definitely there is a swing towards those doing more automatic tasks. It’s harder to then break down what you might want to potentially do with a bit of part practice but some of these patients with cognitive language impairments might not necessarily understand what part practice means or what part of that practice you actually want me to practice so then I just do the, you are better off doing the whole task and seeing how they manage with that task and modifying that whole task to make things slightly easier or more difficult depending on the task.

**I: So the criteria were really broad to reflect stroke really, the stroke unit, and then that’s part of the pilot study. So do you feel having even though it was only a few patients and having worked with them would your normal practice be a little bit different? Would it have been different? How would you have normally worked?**

MP2: I think to get best out of those two types of patients that we have I don’t think we would have done anything more different personally. Would I have been more hands-on in terms of facilitating trunk and doing a lot more feedback I think for those particular patients I tried not to. I think in the past I would have probably done more but because of the intervention criteria I did less so I was having to be much more specific with cueing and external cues. Yes definitely so it did change it for them and it did make it as a therapist harder to then try and get the right activity at the right time.

**I: So they were physically dependent weren’t they so there was that element. Just generally knowing that so to learn implicitly is about reducing some of the verbal information and changing the feedback. What are people’s thoughts on any specific groups of patients that might seem to make sense for or work well with and any groups that it might not? Any thoughts on that?**

MP2: Yes I suppose we got those patients that are able to understand information a lot better the ones without or don’t tend to suffer with expressive or receptive dysphasia as significantly as one of those patients did. Ones that are able to have an understanding and retain that information to then be able to move on to the next task. Although these guys were heavily dependent I still think the two that we recruited did not necessarily have as sufficient cognition and language as I would have wanted to try and do the activities with. I could have probably done more and explored a lot more with patients who had improved cognition and improved communication. The ones that we have had who were able to do a lot more specific implicit learning we’ve done really well with but then they don’t necessarily fit in with the criteria for being in hospital for long enough to participate because they’re actually good, too good sometimes to actually stay in hospital.

**I: So it feels easier to apply it with those patients who are slightly higher level, is that?**

MP2: Yes.

**I: OK.**

FP1: I’d also like to add so there was a patient who was not doing very well with the cognitive aspect and had dysphasia and everything but I was fortunate to see her in community and I found that the amount of or the level of commands or the frequency of commands that I had to give during the hospital stay was quite a lot and compared to what I saw in the community the rehab and the process of getting better was so quick which was in her own environment. Then the level of commands and frequency just got to one command telling her to do this she would just do it. So I think the environment plays a bigger part in a patient who has dysphasia and cognitive impairment if they are quite in an environment which is very much known to them they will tend to respond better to you in such an environment. So maybe that as well leads to the implicit learning less than commands it shows that she can now just plan the activity and do it where it would if you were to give ten commands or repetitive commands she wouldn’t do it. So I think there is quite a lot of things we need to keep in mind for the patient to learn implicitly. It’s quite difficult.

**I: Yes that’s really interesting. Do you think that’s because it’s easier for the patient in their own environment or is it also easier as a clinician to be able to work on those things and therefore adopt more implicit approaches. Or is it both of those?**

FP1: It can be both of those but I think with the sudden cognitive impairment that you might have because of a stroke it takes time to obviously it takes time to resolve it and get back better and it might be that the patient is back to their environment they came from so that’s why we can see that the patient is doing well in the community and not in the hospital because it’s an acute setting. So that can be one of the reasons as well and I think even the environment plays a bigger role because you are there with the people you know from a long time you might respond better to them than the people who are completely new and you had a stroke you don’t know where you are, what you are doing, it’s just so confusing for anyone who would be going through so it might be they don’t want to, they don’t feel very or the new patients, the new people they see it with the cognitive impairment we make them feel more [*24 mins 48 secs*]agitated or just not very well. So that might also affect their response to us.

**I: So its sounding like people with cognitive and/or language impairment the sense I’m getting you feel that you would tend to use more explicit approaches and you feel that that’s needed in someone who has got.**

FP1: That’s what I think that’s what they need because they need more commands or more demonstration to follow what we want them to do compared to who are very good with the cognitive and expressive things. If they are finding that they can do what we ask them but they are not cognitively right they won’t do it.

MP2: I think just tagging onto the same kind of familiar environment does aid in terms of giving more appropriate feedback because patients might recognise oh that’s my chair, that’s my room and instead of us saying, oh we’ll walk 10m do a 180 and then come back in a very unfamiliar environment can be sometimes more difficult for those patients that suffer with maybe communication or cognitive deficits rather than saying get over to your chair over there and they’ll just go and spontaneously do a nice automatic activity which is what we want them to do but it being back in their own home or using pieces of equipment familiar to them from home sometimes you get a better, you might not even have to provide them any cues because they actually realise what’s intended out of them. I think especially when we’re assessing some of our newer patients and we’re working out what their level of cognition and communication is on top of their physical deficits sometimes if you provide patients with their usual forms of equipment albeit it might be more difficult for them physically and actually they get on better and complete an automatic task better with a Zimmer frame rather than say a stand aid or a Sara Stedy just because that’s something they’re familiar with and then you can start seeing their more automatic abilities and then you don’t have to prompt where you have to place your hands, how much you have to straighten your knees etc. because they are so much more familiar with the piece of equipment they have used in the past.

**I: So rather than thinking about the types of patients in terms of their impairment if you also think about things you might be working on. So in the study we were very broad in that it was to do with anything lower limb really including rolling, sitting, sit to stand again which your patients weren’t at that level but just thinking about applying more implicit approaches for different types of lower limb task have you got any thoughts on how well that fits, how easy or not so good in gait re-education versus doing some sitting balance. Are there any areas where you feel it would be more beneficial or easier to do or not? Or where it seems like a better fit.**

FP1: I think so for the patients who can follow giving them a target like drawing lines and saying to step it’s much better because we can get the assessment for how long they can step, how much can they weight bear and we can just adjust this(?) and it’s easy for the patient to just know where to put their feet rather than they have to assume or they have to think how long should my stand be. So in that way it might be helpful and also the sitting balance and using the buzzers I think and if they are colour coded and everything we can just give one instruction that you have to press the buzzer and just keep on repeating that red, yellow, blue whatever so that reduces the amount of verbal commands that we need to give. So I think in that way the target and functionally treating steps with the patient it would be beneficial for them and for us to just little bit less commands and see how they are doing with the stepping and walking.

**I: And any types of tasks that you might be working on or trying to refine where you think oh that would be really hard to not give instructions that include body parts. So more hypothetically.**

MP2: I suppose when we’re thinking about sitting balance I think trying to adapt it to use physical prompts we were using walls and big blue cushion to lean on, rest on, get yourself next to this and we were often catching ourselves with ah rest your shoulder on here, put your right arm on here yes but you had to keep biting your tongue to not say those kind of specific words. So it was slightly trickier from that respect.

**I: I think also it’s always a bit false in a research study isn’t it as well when there’s a video camera pointing at you.**

MP2: Yes.

**I: Because actually what we were trying to do in the study was just create a bias towards implicit learning so it wasn’t like there’s a complete ban on saying the word hand or foot but because you’ve been asked to do something in the study I think I found that across all sites like, oh and every time we’ve said the word foot we feel like we’ve done something wrong, which was not the case it was creating the bias. So it is a little bit of that false scenario and it is OK.**

FP2: But you just don’t realise how many times you say it. Oh god do I really say it that much.

FP1: We are so used to just saying that so it’s just involuntary we would just say what we want.

FP3: I also think when we only had the two patients it didn’t come natural, you knew the camera was there when you were trying hard not to and it was a little bit disappointing because I would have liked more that fitted the criteria to work with but then we did carry it on to lots of other patients with different things we did.

**I: So do you think with time had the study had there not been this little pandemic going on if you’d have recruited more patients do you think it would have got easier?**

MP1: Yes. Personally just in terms of setting it up and getting the camera right, putting the camera in the right position, making sure that we are in the picture there was added elements that made the intervention slightly more difficult than it would normally be. That was a little bit tricky but having the opportunities to just get used to that and I think it would have made each session a little bit less stressful sometimes.

FP3: Or they suddenly needed personal hygiene care and things like that. It was just so unfortunate a few times ones we had.

MP1: Yes if you had more patients it wouldn’t be so difficult I suppose.

**I: No absolutely and actually if we weren’t doing it as research we wouldn’t be video recording and so that’s the only way for us to understand it in the research context but you wouldn't be doing that in real life.**

MP1: No because at times we have used video recording features to record some of our patients just to show this is what you are doing, these are the things that we are looking at and this is how these are what things we want to improve on this is why this went well and they’ll go oh yes it looks so much better doing that. So we did use video feedback at times for some of our patients.

FP3: Then they can see the progression in themselves can’t they.

MP1: Yes and we showed them the videos as well of the ones that we did record, look this is what you did. Reach the number one, oh yes. I think they found that again beneficial even the ones that were maybe slightly more impaired in terms of cognition and communication they kind of showed an improved insight or interest I suppose into the activity.

**I: Out of interest do you think that, if someone was watching themself on video you are showing them, thinking about learning do you think that would fit with implicit learning or explicit learning. So actually rather than you telling someone what to do if they’re looking at it just where would that.**

MP1: I think that would probably fit more in the implicit side in terms of them being able to recognise their own, well hopefully they would recognise their own, well that’s the problem I suppose if they don’t recognise their problems then it doesn’t help but if they’re doing some of their own error correction by looking at the video and thinking actually because I think in the past prior to doing courses and prior to doing this research you talk to the patient and say oh how did it go and you wouldn’t really let them say oh I did really well. You’ll say oh you did really well because you did this, this and this but following the training and doing the study you hold yourself back and wait for the patient to say oh actually I did do that really well and then I kind of go into exploring why do you think you did that really well. Oh because I did this, this and this. It just shows the level of ability of the patient to understand, comprehend and potentially learn from their own mistakes, which I don’t necessarily think I was doing as much as I do now.

**I: Yes it’s interesting isn’t it. Just thinking back to the approach generally and what you were asked to do in the training was there anything about it that was unclear, was there anything that you thought oh I’m not sure am I supposed to do this or that, or I’m not sure how this fits with what we’re being asked to do. Were there any elements that you weren’t sure about? I think everybody takes home the speak less don’t mention the body part.**

MP2: Yes yes. No I think just the added use of the lines, tape, markers, buzzers they were useful, continued to be kind of useful and kind of made it clearer. It did make you have to think a lot more so because in the past you might have said oh look practice doing a bigger step and I want you to get your heel down first, get your heel down and make sure you lift your foot up high enough and then just even speaking to [name] earlier he kind of modified his treatment to get a better step pattern by using a step which he did and he was successful in that activity. Just having to have time to have to clinically reason what activity are we trying to get out of this person, right, OK, now how does that then relate to a functional task that we can try and imitate. If we can’t imitate it then we go through demonstration. There was a bit more kind of you had to process the information a lot quicker or be prepared much more so.

**I: Yes, quite a few people have said that they had to plan a bit more in advance about how am I going to set something up to achieve this. Even if it’s not a functional task but an exercise about how we’re going to achieve it without being too explicit and it took a bit more planning, which I guess with time would get easier as well. Just going back on something you touched on earlier about handling and facilitation just what were your and again for anybody really your thoughts on just knowing a tiny bit about implicit learning and explicit learning where did you think handling sat within that or what did you perceive you’d been asked to do in relation to handling? It’s not a test question because there’s not a right or wrong answer to that so its’ more your opinion.**

MP2: Yes so it’s sometimes I couldn’t seem to relay the information I wanted, I didn’t get the result I wanted from the patient and so sometimes it was just easier to assist them to get there but then it didn’t necessarily, sometimes it worked and sometimes it didn’t and they got themselves there and they realised what they needed to do and you sometimes had to physically get them in there to help them understand. Again the same with some of the stepping practice that you might need practicing the person’s awareness of their limbs, movement or limb activity isn’t necessarily good enough for them to actually activate their own leg independently so sometimes you do need to get on to their leg and say look this is what we’re going to have to be doing and going through dorsiflexion, knee flexion, hip flexion to get heel strike and then repeating that. So that felt slightly less so of the activity that you wanted but it was the only way to sometimes trigger a patient to actually realise, oh there’s my right leg, I do have a right leg, oh I’ve got some movement now. And then they’d get it and then you can revert back to maybe a specific target practice activity or part of an activity because they’ve now realised it, so sometimes I felt it warranted in those cases.

**I: So when you do that do you think well I’m not sticking with the implicit thing then. Is that your?**

MP2: Yes.

**I: You felt that you shouldn’t be as hands on?**

MP2: Yes but then realised that I can’t do anything more and my skills I couldn’t think of anything else that would help them bar doing this.

**I: Obviously with the more dependent patients you recruited as well you can’t not be hands on but it’s more with the approach it’s more what you say alongside being hands on. So if you were helping someone to move their leg and saying right think about your foot and your knee bending and your hip bending that would make it explicit but just being hands on you could argue that’s neither or it’s implicit I don’t know. You don’t really know how someone is learning. It’s interesting how we think about that the hands on. Just for others just for working in stroke generally how much do you think about how hands on or hands off you are – is that something you consciously think about or?**

MP1: I think on the flip side of what MP2 was saying so he talked about the more dependent patients who need that more hands on to get them in a certain position and I feel that sometimes you can be almost too much manual handling where for example someone with poor sitting balance you might be on their left-hand side and they’re leaning into you but that’s because you are there. Whereas if you were to take yourself away from the situation then they might actually independently find their mid-line and sit up nice and straight. So I think on the flip side of some people do need that manual handling to understand the task and what they’re doing I think then you can do too much manual handling where they’re not really sure what you want them to do because you are there whereas if you just left them to their own devices then they might actually right OK I need to actually sit up rather than lean on someone else because it’s easier. I need to actually engage my core and my trunk and then they’ll do it themselves. I think there is the kind of fine line between this patient needs a lot of manual handling to understand what they need to do and then on the flip side you need to be aware that sometimes the amount of manual handling you give someone might not be that beneficial, you might need to take a step back a little bit.

**I: So it’s really individual.**

MP1: Yes I’d say so.

FP3: I had someone this morning I was walking down the corridor who wanted to hold onto my hand but I didn’t want him to so I persuaded him in the end to let go but on that then he held onto the rail on the other side of the wall and when that went he wanted my hand again so. I was trying to get him to be independent but.

**I: Yes a confidence/anxiety thing, yes. You have to judge it from the scenario don’t you. OK so just thinking about so this is obviously a pilot study so it was really to understand does the study design work, can we recruit people, does asking you to video things work and we wanted to understand what patients in the intervention sites got ended up being any different from control sites as well or does everyone just work in this way anyway. I don’t have the answers to any of that until we’ve analysed the data. But if there was a future study that showed that actually working in a more implicit way is beneficial maybe in certain scenarios or for certain groups of patients but it did show that what do you think would need to happen to get therapists broadly not just you guys to adopt different ways of working into practice. That’s a big question.**

MP2: I think it’s just having the opportunity of getting different types of equipment and support so just even the buzzers was really helpful for you to give us. Sometimes you think oh it’ll be alright we’ll just buy them in a minute but you never do so yes I tend to use tape, use markers. I think the added extra equipment that you might need to support you with that having a little box of goodies somewhere that can support you in that kind of intervention for those particular, yes.

**I: So having stuff available.**

MP2: Yes.

**I: Is there anything else? Not necessarily in your service but just generally that.**

MP2: Well I think there are a couple of us that have been through the movement science courses and I think they’ve been very well received and it’s been really useful to use those skills which is very much the study’s approach. A lot of our qualified team have gone through that, it’s been very useful and we’ve been drip feeding that to the rest of our team and the stroke early supported discharge team and the OTs and they’ve all taken on that extra bit of knowledge and training and incorporated that into their normal routine now. So having the opportunity to get extra training would be even just the, because I think you did an online lecture as well didn’t you during the middle of COVID and I think staff found it very useful. So even that kind of, they didn’t need to go off to the mainland and spend two hours to get there to get the training done just to get somebody else to go through some of the information which we brought up has been useful.

**I: So accessibility of the training being as easily accessible as possible. Anything that you think is a particular challenge with regards to adopting changes in practice, this type of change in practice. So in the training we gave you some principles and we gave you some examples and said well these are just examples to help you visualise what we’re on about. In terms of that type of resource is there anything.**

MP2: Yes I suppose it goes on with the education in training you kind of see new ways of how maybe other colleagues are doing similar activity and how they are using a different kind of intervention and you think oh why didn’t I think about that. So having potentially a resource base or opportunities to link in with your peers to discuss how they are treating certain client group just again to support that in all the different Trusts I suppose. Yes because there’s levels of experience in different areas so it would be useful to share that knowledge.

**I: Obviously you guys have really heard about it today but if you were being sent off to try and put some of this into practice tomorrow what more do you think you would need to make that as easy as possible for you as clinicians?**

MP1: I agree with what MP2 said equipment wise obviously you need more equipment and things to make it more accessible to do it. I’m not sure really I think the hardest thing for me would be just, I feel like sometimes at Uni you get the way because obviously being newly qualified for me at Uni you don’t necessarily learn as much in that way and it is kind of a lot more because you are trying to express your knowledge more at university you then kind of speak, I don’t really know how to explain it, but you speak in more jargon because you are trying to express your knowledge to your lecturers so if it was more, I don’t really know how to explain it because I know that at university it’s a bit different they kind of need to know how much you know. I almost feel like you are working more explicitly because you are talking, oh bring your hip up, do this, do that especially with MSK kind of side of it. So I think if they implemented it throughout Uni and things like that that would definitely be a lot beneficial. But I think that would be the biggest challenge for me especially being newly qualified is just changing my language and not speaking in jargon and being more layman terms with it and being more functional with it and stuff. I think that would probably the biggest challenges for me at the moment.

**I: It’s hard to unlearn habits isn’t it. I think you are right we are taught, I suppose when you come on placement as well you are being encouraged to demonstrate what you know so the way you do that is by talking out loud and it becomes part of our pattern in doing things. I was going to ask something else about that but it’s just gone out of my head. Oh just to go back to a point you made earlier MP2 you said quite a lot of staff here have done movement science training of some description or modules and I just wondered how much you felt just the general culture and way of working in the team was important. Do people all work in the same way and do you think that might be different from other stroke units?**

MP2: Yes I suppose my experience here in this Trust and I’ve worked with lots of different people in different, well I have worked very closely with a variety of OTs and some OTs have had different experiences which then inform their treatment choices and sometimes I feel their treatment choices aren’t necessarily as in line with movement science and you then discuss the scenario and you try and breakdown the clinical reasoning of what they’re trying to do. It’s sometimes not overly clear so our joint practice is not always married up sometimes. Most recently that has changed quite a lot and there is a very much more, seems to be a more MDT PT/OT approach which is slightly more in line in terms of the information on that patient given in terms of what is expected in their treatment sessions and what kind of activities they then do.

**I: Is that just for any approach to really work it needs to be consistent.**

MP2: Yes.

**I: Especially when you see people in therapy. Just out of interest for all of you do, I’m only asking because it’s come up in a few other focus groups what your thoughts are nursing staff and the nursing team on the ward when you see them working with patients transferring them out of bed or helping them to mobilise – anything you observe about how they work and where that would fit with implicit/explicit generally or not.**

MP2: What are your thoughts.

FP3: I just feel they are swimming against the tide a lot of the time. They haven’t got enough people.

FP2: We do help as well we do therapeutic washes and things and prepare patients if we’ve got time it just depends.

FP3: But sometimes it’s ridiculous isn’t it how many they’ve got to run round to and for the amount of needs of the patients. We’ve not been very happy with things.

FP2: Not lately, no.

MP2: But that’s because of staffing.

FP3: That is a staffing situation.

**I: I suppose so regardless of the staff situation if you were helping a nurse to get somebody out of bed or you are in the bay next to them and you were overhearing what they are doing do you think they tend to instruct people a lot as well? Like I’ve said I think therapists do that, do you think nurses do?**

FP3: We have a mixture don’t we, good and bad don’t we. Some will just say get to the chair, very simple and the patients can follow.

FP2: Yes they’ll say that we’ve seen you with the therapist, physios have got you up you can do this with the frame, you don’t need a Sara Stedy. Some of them are like that which is good really because it pushes them on a bit further. We can go down the ward sometimes and we just know a certain HCA has been on because it just smells wonderful. She’s put people who have had strokes in the bath and that’s all therapeutic though, it’s really helpful I think. Sets them up for the day. I think they’re better really doing their therapy after.

FP3: And then you’ll get some that seem to leave them all in bed.

MP2: We do sessions where you try and do work jointly with them and kind of express how we might want to get the patient out of bed or get them up into standing and again reiterating actually you don’t necessarily need to be as hands on as you might need to be but there is still a general trend for nursing staff to be maybe more hands on when they don’t really need to.

**I: So doing to.**

MP2: Yes they are more doing as opposed to encouraging, not encouraging but more of an active rehab process I suppose.

**I: It’s just interesting it’s not the focus of what we’re doing but yes.**

FP3: In fairness sometimes I do think it’s time.

MP2: Yes.

**I: Oh yes there will be lots of different reasons why.**

FP3: If we do the therapeutic wash and dress to help them it’s very different from.

**I: Yes absolutely. No absolutely. Last few questions although I keep saying that and realise I’ve got some on the back. Every time I’ve said that I’ve thought oh it’s not the last one but we are nearly there. I think you’ve covered a lot of this. So in some of the centres where they have been recruiting to the trial some of the things I’ve heard people say, oh we’ve got a patient and they are ideal for implicit learning approach, this is the perfect patient. In your view what would make somebody ideal? Obviously there is trial criteria and then there’s like, oh this one seems to make sense. What would make somebody ideal for using this approach with?**

MP2: So usually the ones I think are ideal usually disappear off within about two days. So the ones I think are ideal are the ones that aren’t cognitively impaired, the ones that are they can have some kind of mild language problem but mainly as long as they’ve got some kind of motor activity that’s really helpful. Those ones with dense zero out of five upper limb and lower limb and then to have to work trying to recruit some kind of, trying to get some activity and there is nothing there I find that generally difficult anyway but it’s even more difficult when you are trying to go through a specific approach. There are numerous ones I could have taken but yes those ones like the gentleman we’ve got now in HASU 3 he’s really good, he’d be good for that study although he’s got quite significant language impairment he’s got movement patterns there that you can encourage and he’s also very visually driven as well at the moment so again you can rely on his vision to actually go for targets, which is a lot easier than touch I suppose, searching out finding somewhere to go and do. So yes people with no visual impairment or minimal visual impairment is good. They would probably be my ideal patient.

FP1: Even with less hearing impairment because oh cannot hear you you need to repeat yourself more than once with a voice like me he needs more repetitions and got a softer voice.

**I: Yes so it’s a slightly different thing isn’t it if it’s a hearing impairment because actually if you are repeating it a lot for that purpose then that doesn’t necessarily make it explicit because actually you are repeating it because they’ve not heard it. So actually they’re only probably hearing it once but anyway it’s a different thing but yes it makes it more difficult doesn’t it to reduce the amount of communication. The nature of it maybe not. It sounds like they need to be in hospital for two weeks, was that quite a challenge and something you felt limited recruitment?**

MP2: Yes. So interesting since COVID there’s been a big rush of getting patients out of hospital sooner just to make sure that they’re not in hospital and potentially getting COVID or to making sure there’s a bed available for maybe worsening cases coming up so there’s been a big drive. So our length of stay has dramatically reduced in the last two years. But when I was doing the recruitment stuff, no not recruitment the.

**I: Screening log?**

MP2: Screening log, the amount of times I’d say oh less than two days, less than three days, less than four, it was so common. Then I was trying to work out was it always like that and I couldn’t, it just feels like we have been discharging patients a lot sooner. But also when I was screening them a lot of them have been fairly independent as well, I was thinking what’s, is it just a funny year or is this a new thing. I don’t think I’ve ever explored it in that way before how quickly and how good some of the patients are but when I was doing the screening I was like.

**I: It’s like you had two ends of the spectrum didn’t you, in and out the door very quickly or actually quite unwell.**

MP2: Yes. The only ones that we could recruit tend to be the ones that were going to be in hospital and the reason they were in hospital is because they were more medically unwell and because they’re more medically unwell they were slightly on the more severe end of the stroke scale. But yes anyone mild-moderate they were out of the door and having SESD input pretty much straight away because this Trust has got no kind of in between. So you either have hyper acute, acute, rehab in this ward and then SESD as opposed to other hospitals where you might have a separate area or separate location where you get transferred to. This is the only stroke inpatient unit.

**I: And at this phase of the study it was really just keeping it to inpatients just again because it’s a pilot feasibility and there’s lots to understand but I think for lots of different reasons including some that you’ve talked about ideally in a future study we would be looking at inpatients into ESD as a continuum which I’m sure would A) help recruitment but also B) more clinically relevant.**

MP2: Yes I think the way things are going I don’t think it’s going to change that we’re going to have less days, more days in hospital I think it’ll just be less days and more.

**I: No one is going to suddenly turn around and say keep them in.**

MP2: No exactly. We have to fight to keep them in because they don’t have the equipment out in the community.

**I: We know. We are really nearly there. So just last few questions really. I suppose maybe it might be difficult because you have had some turnover in staff as well haven’t you since we started the study.**

MP2: Yes.

**I: But just thinking about throughout the involvement in the study was there anything applying the implicit learning approach is it something that you’ve talked about more informally as a team? I ask that to get an understanding of the issues that might have been raised generally but I don’t know if you’ve had enough opportunity because not everyone who’s been trained obviously and is no longer working here was actually involved I don’t think but just whether there were things that cropped up or cropped up after the training where you all came away after I’d left and said oh God what was she talking about.**

FP3: The only one interesting thing that surprised me was when you did the initial training and I took it all on board and then we had the gap didn’t we and you did a refresher training and most of it I remembered and I was fine with and the only thing I had blocked from my mind totally because it’s something I didn’t want is when you brought up about being videoed again. I thought oh, oh yes but everything else I could remember but that was the one thing I had chosen to block. Funny.

**I: Do you think the video camera changed what you did having it there? I suppose it made you more aware.**

MP2: I don’t think it necessarily changed, for me it was just a bit more

**I: Faff**

MP2: Faff yes. Bit more time consuming that I would want to be and having to keep checking it and turning it off and pausing and making sure the angle was alright.

**I: Do you think the patients were bothered by it?**

MP2: One of them didn’t seem to mind it he was quite, he would put a smile on the camera.

**I: It was quite funny when we watched them back some of the patients talked to us and were like hi.**

MP2: Yes so one of them was a little bit more engaging with the camera.

**I: It’s their moment of fame.**

MP2: Yes. The other guy was, I don’t think he really minded whether it was there or not. He knew it was there but he was having to concentrate on so many other things. Yes I don’t think there was anything.

**I: It wasn’t a particular barrier?**

MP2: No.

**I: So just reaching the end. It’s been really useful to hear all of your thoughts and there’s so much, I mean we’ve been chatting for nearly an hour actually and there’s so much in what you’ve said that will be really valuable for us as we think about if and what we do with the research going forward. So finally is there anything that we haven’t talked about that you wanted to bring up or anything that you think that we’ve not touched on in relation to the study or in relation to the concepts of implicit learning?**

MP2: No I think me if you said oh you are only going to have to recruit for four days or five days I think we would have done fairly well.

**I: Patients within four, yes only here for a short amount of time. Yes.**

MP2: Yes because like you said if we kind of moved the study out to SESD that’s especially in this Trust this where it all goes to now so yes anyone that is able to be cared for would leave this hospital.

**I: And it’s going to be hard for us to understand what was challenging because of the pandemic and how that changed services versus what actually would have just been challenging anyway it’s going to be difficult but it has been really variable in every site in terms of recruitment.**

MP2: I think for us as well we didn’t have our typical gym so we were still ad-hoc making up our own gym on top of trying to do the research as well. As a physio service we didn’t feel like we necessarily had a base for our patients to go to which felt secured, quiet, had all the equipment there and then as opposed to, oh no, where have I left that tripod again, oh it’s back down in the office. It was all just a bit, not because of the research it’s just the actual period of time it occurred it just made it a lot more difficult. We were working in one gym in [name] and we also had some working in [name] physio gym, so it never.

**I: And the reality is even without a COVID pandemic some of those things around research they do make it more difficult having to remember the camera, where do people store a camera and has anybody got a locked drawer to put it in. So those are the logistical things, they’re extra things to think about in busy days aren’t they.**

MP2: It’s just being prepared to make sure that I’ve still got a plinth where I left it all set up or it’s not moved or I’m having to rearrange equipment again.

**I: And your patient is still where you left them. Therapy assistant is still where you left her.**

MP2: I’d quite happily do the project again but I think I’d have more luck if I get the or if the recruitment time was a criteria slightly less.

**I: Things have changed a lot since we initially planned it back in whenever it was. This bit of paper says 2018 on it so that was obviously thought about a long time ago. Right I’m going to stop the recorders but normally the minute I do that someone says something really insightful so if you’ve got something in your brain can you just say it now. If not I’ll stop them.**

**FOCUS GROUP 2**

**I: Just before we start with the questions to help [name] and then also to help the transcriber I’ll just ask you each to introduce yourself if that’s OK and it’s just so we pick up a little bit of your voice with the name. So if you are happy to say who you are and what your job role is that would be great.**

FP1: I’m [name] and I’m a band 7 physio over at [hospital] now on the stroke unit.

FP2: I’m [name] band 7 physio at [hospital] stroke unit.

MP1: I’m [name] a band 5 physio at [hospital].

FP3: I’m [name] a band 6 physio at [hospital].

FP4: I’m [name] a band 6 physio at [hospital].

**I: Thank you very much. So obviously you’ve all had a different level of involvement in the study and it’s quite a while ago for some of you that we started this. I don’t know even know how long ago it is, it’s a long time ago isn’t it because of COVID.**

FP4: Before I went on maternity.

**I: Before you had a baby. At least 10 plus months ago, it’s more like a couple of years I think since we very first started. Because of the fact that the trial has been quite disrupted I’m aware that some of you have had more involvement than others which is absolutely fine. So just to start us off it would just be helpful to know how many patients if any you’ve each seen roughly that you’ve trialled the IMPS approach with, so how many patients have you been involved with. So is there anybody who hasn’t actually treated any? So FP4 and FP3?**

FP3: Not really. I’ve seen the outcome measures mainly. I haven’t really done the treatment.

FP4: Yes I did some of the outcome measures. [*2 mins 13 secs*].

**I: Yep OK.**

MP1: I’ve seen one session and then I’ve seen a couple of outcome measures. I’ll be doing some later on today.

FP2: I’ve seen 9 and then the 10^th^ today.

**I: Brilliant so FP2 has done quite a lot of treatment.**

FP1: I’ve only seen maybe 2, 3 for outcome measures. We’re missing [name] who saw quite a few.

**I: Yes, fine, so there’s a mix in the room and that’s absolutely fine. So we’ve adapted the focus group questions to account for that so knowing that FP2 has had the most experience and is probably speaking from that experience and the rest of you might be more about what you think might happen or not happen. So that’s absolutely fine. So because it’s a pilot study we just want to understand more about how the IMPS approach might work in practice and also the best way in research to evaluate it. But our discussion today is focused mostly on how the IMPS approach works in practice and less about the logistics of some of the research stuff like recruitment and that kind of thing but more about actually just as clinicians your views on the different learning approaches. So just to kick us off I just want to ask you to describe what you thought when you first heard about the research trial and when you attended the training about the IMPS approach that we’re testing. So if you can think back which was a while ago for some of you but what were your initial thoughts?**

FP1: It’s interesting to be part of a research trial so that was intriguing, interest, excitement I suppose. Listening to the approach it sounded quite structured which was something that potentially would be quite easy to follow. There were all the pictures that you gave, there were some exercises, there were some ideas of what we could use so in theory it sounded like it would be quite easy to implement.

FP2: I think it carried on quite nicely from movement science training courses that I’d been on so it was good to be able to focus on implementing some of those treatments.

FP1: I think as well in terms of the reps it sounded like a good basis in order to be able to build a lot of repetitions which obviously is what we are trying to do.

MP1: I think us not having much experience in treatments being able to use a completely different way of treating people and coming up with exercises would be quite helpful in terms of not just going through exercises in a booklet or anything like that it’s completely changing how we do it is more good.

FP4: It makes you think about your treatment approach regardless of whether you are treating a patient in the trial or not and it makes you realise sometimes how much you actually do talk during a treatment session.

FP2: And how hard it is not to say body parts.

FP4: How often you are not implicit.

**I: How much would you all say you already knew about implicit learning, so before you got involved in the study was that a concept or a term that you knew about or concept that you knew about?** **Or not necessarily.**

MP1: I’d never heard of it, no.

FP1: Heard of yes just when you read about different principles of learning and things like that and how we learn stuff but never really applied it.

FP2: Familiar with it through the movement science course but probably haven’t focused in on trying not to give as much verbal feedback and like the need for fewer words and more just to make the exercises obvious so they’re learning more implicitly.

**I: So you knew a bit about the theory but maybe didn’t always.**

FP2: Put it into practice as much.

**I: The others are nodding. When you attended the actual training did your views change in any way or is there anything you remember about the training particularly?**

FP3: I thought the comparison to athletes and how they learn helped me understand what you meant and made me realise oh yes that’s how we learn so why aren’t we applying that to our patient group. I hadn’t really thought about it like that before.

**I: So that’s something that stuck out for you. Was there anything that stuck out for other people?**

FP1: I think the focus on body parts was something that stuck out probably for all of us just in terms of how difficult that was going to be to recognise that that was going to be a difficulty to apply straight away because we’re aware of how much we talk about different parts of the body when we treat people.

FP3: And just limiting our language in general like limiting how much you say to someone especially I think with cognitive impairment so I just thought I don’t know how I’m going to do that. You feel like you really need to speak.

MP1: I think the type of feedback as well instead of just always saying well done or good or anything like that actually because sometimes they haven’t done well for example so having that more quantitative measure of their success is a bit better I think.

**I: So it sounds like the communication bit of it felt like it would be different to what you were already doing?**

FP3: And hard because and not giving, well you can still give positive feedback but not saying well done is quite hard to hold yourself back because you want to be encouraging and limiting how much you are talking feels like you are just.

FP4: It doesn’t come naturally does it.

FP2: We’re not comfortable with silence I think are we so when you are just letting the patient carry on I think that was really hard.

FP4: Because you were just thinking oh what’s the patient thinking, are they worrying about what they’re doing, do they need reassurance because we do need reassurance as humans a lot of the time.

FP1: It’s that filling in the gaps isn’t it, filling in the silences that we’re desperate to do.

**I: Why do you think that is? Why do we do that, we collective therapists.**

FP2: I think it’s because we want to be friendly to the patients and yes I mean when there was is it [name] when he does his training with [name] they were saying basically the patients go to the gym to work and they’re not there as their friend they’re there to give them things to do. But I think we try and be everything for the patients on the stroke unit probably because there’s not enough volunteers, not enough healthcare assistants so you are there as the person, as the highlight of their day sometimes. So if that’s just focused on exercises it doesn’t feel like the friendly thing to do but I think it’s shown that actually that is important because then the person can be really focused on that and then have their time talking to you at the beginning or at the end.

FP4: I think sometimes motivation if the patients are struggling with motivation you are trying to fill that gap to help keep the motivation going.

FP2: And like FP3 was saying the cognitive ones like the cognitively impaired ones they are definitely much harder to engage in the approach because they do lose focus and that is when you end up using your words to keep them at the task.

FP1: But maybe we’re doing that a little bit too much if they’re cognitively impaired.

FP4: Yes because they’ve got the overload.

FP1: Yes distracting.

**I: Have you had many people in the study with cognitive impairment?**

FP2: Yes they were the really hard ones to treat.

**I: So your experience was that they were harder.**

FP2: Yes.

FP1: Because it was one stage commands wasn’t it the criteria.

**I: So with those patients what was your experience? Do you think you didn’t manage to adhere to the principles or you did but you just had to think about it more?**

FP2: It was a lot harder to adhere to the principles and it made it more difficult for the therapists because you’d have to really go away and think about the exercises again after the session and how you could make it more implicit and more obvious. The worst ones were the ones that were cognitively impaired but also had inattention to that side because then you felt like you needed to constantly give them prompts and just saying take your cross to the marker I found that didn’t work well with those patients because it was some of the earlier ones.

FP1: Yes I’m thinking I probably did see another one, yes.

FP2: I think those are the ones that [name] found hard as well because they met the criteria but their inattention was such that, yes. I guess also you wanted to focus on more functional things with them and these exercises are very much it feels more difficult to make them functional because you are taking their, like things like step ups obviously are but where you are thinking about taking crosses to targets it’s not always.

FP1: It’s not very much in context is it for them and it’s not or where there’s a goal there it’s not like you said it’s not functional when you are not reaching for, well I suppose you could reach for a cup.

FP2: Well this is it it’s probably just in the way that I’ve interpreted it and the way I’ve managed to carry out the exercises.

FP3: It depends on what you were focusing on with them in your treatment session.

FP2: Yes and for lots of them it was still because they had to have sitting balance didn’t they.

**I: Yes only five seconds but yes.**

FP2: Which is quite a brief amount of sitting balance. So some of them we were actually working on the sitting balance and that was often they were the hard ones.

FP1: And they’re the ones who often had such high levels of fatigue and all that kind of stuff which was really limiting as well.

**I: So thinking about that group do you think they needed a different approach. Do you think that the approach didn’t work for them or just that it was harder to apply it just so I’m clear.**

FP2: Personally I think it didn’t work as well with them and I would prefer to use my hand, a lot more hands on with those patients.

FP1: There was one lady we had and I don’t, she must have been part of the study, who we tried to do all the IMPS stuff initially and got nowhere and had to go back to sitting, hands on more trying to get sitting balance better and then we could progress onto the IMPS type stuff. But initially and when her cognition improved a little bit as well, but initially it wasn’t going very far.

**I: So just thinking about that a little bit more so you felt you needed to be hands on what about the communication part – do you feel those types of patients needed more explicit communication as well or was it the handling bit or the verbal bit or both?**

FP2: Being able to do the handling helped more because like FP1 was saying giving them lots of verbal information probably wasn’t that helpful to that kind of patient anyway but I think just being able to put hands on for where that patient needed to be was more helpful than trying to get them to go a target.

FP1: To them why am I taking my shoulder to that cross it doesn’t make any sense.

FP2: Especially when it was on their inattentive side that they’re not really aware of anyway was really hard.

FP1: And it seemed a bit meaningless potentially to them I don’t know whereas if you were working together to get say a reach or whatever it is you are doing but that needs a bit of hands on then maybe that worked a bit better. But it could be just the types of patients we had because there weren’t that many of those patients either so it’s difficult.

FP2: Yes it felt like the earlier ones we got were those lower level ones and then more recently when we’ve had the higher level ones one person in particular he was so motivated and really motivated to do really high reps and it worked brilliantly for him.

**I: I know who you mean.**

FP2: But that kind of patient that’s very self-motivated and can understand the exercise that you’ve taught them to do it independently it worked brilliantly but we haven’t had lots like that sadly. We’ve had ones where, I had one the other day where I basically felt like I was fighting against his limb to try and just make him understand the exercise because he just didn’t have a feel of where he needed to be. So I’m getting him to go to a target but the leg is doing exactly the opposite to what I’m asking him to do and he says that he’s understanding but actually the leg isn’t going, so then what do you do other than go like we took the exercise back loads of steps to the easiest exercise but then it had to be hands on for him to feel which then isn’t really the approach is it.

**I: I’m trying to think of my next question. I think what I’m wanting to ask is, I’m trying to think of how best to word it though, so was your interpretation that in the IMPS approach you can’t be hands on. Was that your understanding? Do you think being hands on means someone can’t learn implicitly or that they can.**

FP1: I thought as soon as you put your hands on to that person that it’s becoming more explicit but I kind of thought we were aiming to be hands off as much as possible but if we had to be hands on we could a little bit but I didn’t think we could support the patient and things like that which is why I found it very difficult moving it into gait but that’s probably another thing. That’s probably another question.

FP3: I would have from the training thought it’s much more hands off because I would say hands on is more explicit because you are doing something for, you are facilitating movement but I don’t know.

**I: So when you are doing that what do you think the patient is aware of? How does that – there’s not a right or wrong answer I’m not sitting here thinking there’s a right answer to this that you should be saying there’s not it’s just interesting how handling comes into it and where we think that sits.**

FP1: I suppose if you are guiding movement you are looking for the patient to follow so you are guiding and they are following and then you are hoping that they’ll recruit and participate and join in kind of thing whereas if your hands aren’t on them they are initiating the movement and then kind of, so.

FP3: I guess with handling you are giving external, outer feedback sensory like something that you are doing to them rather than them feeling it themselves. I don’t know if I’m explaining that.

FP2: Yes because to me your handling is more like your Bobath handling approach that like obviously was done for years and years to me the lower level patients I feel like I’ve used more things that I’ve learnt on Bobath and that’s how I’d liked to, that’s how I think they are better managed generally to feel the movement and then like FP1 was saying try then recruit more for themselves. But the patients that are cognitively able to I think the implicit thing of giving them a target driven exercise to do works brilliantly for but I think we still need the two different approaches for the two different types of patients.

**I: When you are facilitating movement how do you think you use your communication in that as well so when you think about some of those earlier patients you recruited to the trial who you felt were lower level and therefore needed physical assistance.**

FP4: I guess you can still use external targets while facilitating but I think I would naturally use our body parts and if you are facilitating an elbow and a hand or something you would naturally.

FP2: You’d be like reach your hand towards the.

FP4: You probably can if you change your thinking you could still use the external.

FP1: Yes you can can’t you but it’s very difficult to compute it when you are doing it.

FP2: It’s almost easier with upper limb than lower limb to do that like with upper limb you could use the implicit approach like reach for this item, reach for that item and then just grade where they are. But I think for the lower limb it was a lot harder because it’s only when you are walking and stepping and kicking a ball that are kind of things in context that are functional the other bits of it to try and get those more integrate movements are very exercise focused.

**I: I suppose this is quite difficult maybe for everybody apart from FP2 perhaps who has used it a little bit more but has your view changed with time. So the more patients you’ve seen has it got easier or harder or has it just been different because every patient is different in terms of applying it, sorry, is what I mean.**

FP2: Yeah I mean I feel we’ve been quite fortunate in that more recently we haven’t had the ones that have met the criteria that have been more cognitively impaired so we’ve not had those lower level ones so it’s been easier in that respect but everyone has been so different it’s quite hard to compare them. I guess the knowledge of the exercises you want to use gets easier as you are more familiar with them but I don’t really feel like I’ve used a big amount of different exercises, a wide variety of exercises. I feel like it’s been quite similar ones for each patient and then with ones that don’t do as well, aren’t doing as well, trying to think back and make the exercises simpler has been more of a challenge for me.

FP3: Do you think it’s got easier to not use body parts.

FP2: No I still do it all the time. All the time.

MP1: I definitely find I’ve used more of the IMPS exercises in normal treatment. Some of the easier ones like the skateboard one and step ups and things like that I’ve definitely used a lot more just because once they are set up it is easier and you don’t have to then give as much or do as much.

FP1: I think as well the idea that you can get a patient into the gym and you set them up with an exercise and then they can virtually bang out 20 or 30 reps of it or whatever it is it’s becoming easier to do that for me than it was at the start definitely and using external bits and pieces which you wouldn’t have thought about doing before. You wouldn’t have thought about using a buzzer at the back of the knee or whatever to get knee extension. That’s becoming a lot easier. I think we did have a lot of obstacles in our way though didn’t we because we had the gym move so we didn’t really have any equipment, then we had COVID and then, so it’s been very stop start.

FP2: It’s been a bit of a slog hasn’t it.

**I: Yes it’s been really sporadic hasn’t it which does make it hard. One of the reasons of having a cluster design is that supposedly you can kind of embed it a bit but for lots of reasons out of everyone’s control that’s not been possible so you probably have to really think.**

FP2: Because that’s a good point because it’s actually been the easiest to run it now because we’ve generally got the gym and our space with all of the stuff in so it has been much easier because it felt quite stressful. I know [name] when she was seeing most of it we were on a completely different ward, didn’t have a gym and.

FP1: All the equipment just kind of disappeared didn’t it.

FP2: Yes so that was really hard.

FP1: So I think in hindsight to have boxes of all the equipment you need or whatever would be really useful.

FP2: Yes which we kind of, so you had set us up with stuff it was more just the fact that everything was just.

**I: Yes there were some challenges weren’t there. Just thinking a bit about that so you’ve mentioned some of the exercises that were given as examples in the training how easy has it been to just take the principles but use them for other exercises because obviously you weren’t being asked to just necessarily use the ones you were given they were examples. Is that difficult to do when you’ve got a patient in front of you?**

FP1: I think when you’ve got a patient depending on for me, you’ve done it more than me, for me if I’ve just got the patient in front of me and I need to change my idea about what they need because you can plan it beforehand and come up with a really lovely treatment session but that takes a bit of time that bit of it is difficult if you haven’t done it that much. It’s kind of that, oh actually they’re a bit better now I need to upgrade these exercises very quickly to think of them on the spot is a bit challenging I found.

FP2: Yes and [name] found the same because she was doing a lot of them over there and quite often she’d want to run them past me then and that was because they were the ones that needed tweaking because they weren’t finding them too hard. That’s what this.

FP1: Or too easy.

FP2: Yes like this week with one of the patients was finding it hard to coordinate which way to go it was harder to suit the very low level patient I think that struggles to recruit anything.

FP1: I think it’s that kind of you don’t feel like you’ve got the freedom to be that creative when you are in a treatment session because you are stuck to these parameters of doing this, this and this and you’ve kind of got an idea before you go into it of what the treatment session is going to be so if they are worse than you are expecting or really tired or if they are suddenly a lot better that’s not helped with having a video recording you, going oh God what am I going to do now. I think that would obviously come with practise but it was quite difficult.

**I: Which does kind of reinforce that it was a bit different though to what you’d normally do. Sometimes because some people might think well I work in this way anyway and this is what I do anyway but actually if you are having to think about it a lot that suggests it’s a little bit different. Did you have many patients enrolled who had communication difficulties? I think you had a few?**

FP2: Yes early on we did.

**I: How did you find that group or for those who haven’t seen them how might they anticipate that?**

FP2: Those were some of the harder ones. Yes they generally it wouldn’t have been so bad if they were just expressively, I’m trying to think of the ones we had. I think it’s the more receptive so they were only following one stage commands and then we’re getting them to take crosses to mark the targets. That was really hard.

**I: Do others have any thoughts on that on using, how it might feel to use the approach with someone with communication problems.**

MP1: Yes again I think if they’re receptively if they are really struggling then because again I think they’re the patients we would probably tend to communicate a bit more with or change how we do something and make it a lot more functional and therefore then sometimes again with the lower limb sometimes those exercises were a bit hard to think of.

FP4: It’s funny though because you think if their communication is impaired that actually not using communication would be a better way to do it and something a bit more visual might be easier for them but it clearly didn’t work necessarily work that way.

FP2: No and that’s what I would have expected but I found it harder.

FP1: I think it’s going back to that thing of you can introduce function to them but if you are just looking at taking weight transference across so getting your shoulder to a cross it’s not that functional and it’s a bit abstract for them I think for some of them those lower level ones.

**I: Do you think there are ways you could have made for those lower level patients could have, so made it more functional but kept it more implicit because the two go together really.**

FP2: It depends on how low level they were. I think generally those patients were the ones that actually were still working on sitting balance with and they didn’t really have enough activity to be at the point of doing the step ups and things that would have been more functional. I think it’s hard to think of the functional low level.

FP1: Yes especially if you are looking at trying to get some weight transference or something with a reach with the arm but if you’ve just got an arm that has got absolutely nothing in it and is very neglectful and sensory deprived and all that kind of thing then it’s difficult to introduce that as a functional or tap into them with that.

FP2: Because their body schema is so impaired anyway so they’re just not able to engage but then the people who have got good body schema and are really aware of that side brilliant they can just plough through the reps.

**I: You have touched on this, you’ve talked about applying implicit learning and how that’s different with different groups of patients just we might be going back a bit but just to ask you whether you think it’s, how important it is to consider instructions on feedback that we are giving patients just more broadly but in treatment sessions. Is it important to think about our instructions and feedback and what makes you think that or what informs that view?**

FP3: Yes I think it’s really important because I think we don’t think about what we’re saying sometimes like FP4 was saying that we will just say well done but actually when that’s not necessarily like it might still be our opinion that they’ve done well but it might not be their opinion so.

FP4: Also some of them struggle to see their progress but actually if you are giving them you say last week you managed three out of ten and this week you managed six out of ten it’s showing them their progress a bit more than just saying oh well done. It doesn’t necessarily mean as much to them.

MP1: Yes I think it’s saying the right things that again we’d normally we just blurt out something. And it’s thinking about actually.

**I: I’m going to quote that.**

MP1: And again it’s the communication, again from not working in the stroke for that long making sure that you communicate in the right way to the stroke patients because you’ve got to change completely how you communicate with some. And that’s quite nice.

**I: So how do you know if you are doing it in the right way for an individual, what would you use, how would you know?**

FP2: I guess before we would have always said things like tuck your bottom in, straighten your knee and that doesn’t always achieve the movement you want because that’s always generally when we get a band 5 and we’re like no don’t say that give them more of a goal because we don’t want them just forcing their knee into extension. So I think we definitely used a lot more body terminology before using the approach and definitely said well done far too much. So this does mainly it’s every word you are saying actually counts a little bit more and it’s making the patient think about achieving a movement rather than thinking of all the individual sections of that movement which then don’t actually end up creating a good movement pattern anyway.

**I: So you would know based on what you then see them do is that what you mean?**

FP2: Yes.

**I: The output or the response.**

MP1: Yes. And I think it would be more difficult if you don’t know if you are meeting that patient for the first time and doing treatment it really helps knowing that patient a little bit to know even within saying less it’s knowing what to say even for that patient compared to someone else.

**I: Just picking up on that how would you know what you are doing is working from the patient’s perspective?**

FP3: I think sometimes patient expectations like when we come to a SUM meeting often we’ll have patients that have got unrealistic expectations and that might be because we’ve constantly just been saying you are doing amazing, you are doing amazing and they assume that that means they might walk or something but actually if we’ve been specific with them and said today you managed to sit on your own next time we’re going to try a stand they might be a bit more realistic about what they are achieving rather than us just being like, yes you are doing really good, you are doing really good but not being specific about it. I think when it comes to the meetings some patients being very confused by us being so positive sometimes maybe, which we’re being positive because we want to build a relationship with them, we want to make them motivated to come to physio and engage and try but yes but maybe being a bit more specific is actually better and maybe that’s how we see it as if the patient actually is aware of what their specific goals are or what they’re achieving.

**I: We may have touched on this a little bit as well but with regards to the approach and the principles that you are being asked to try and apply was there anything that was unclear do you think? Is there anything about what you are being asked to do or about the implicit learning approach that wasn’t really clear to you or didn’t make sense?**

FP1: I think I had a problem initially with sometimes we just chose slightly abstract exercises that maybe didn’t translate enough into, we’re getting back to this into function I’m thinking of we do the skateboard one, we do a bit of, I can’t remember them but some of them it seemed how do you translate that now into standing up on your leg. It seemed like a big leap from one exercise to doing something functional and standing for example and the progression from just being able to do the skateboard exercise to that seemed huge but we didn’t seem to be progressing them always in the right way I felt I don’t know. Do you know what I mean?

FP2: I see what you mean.

**I: So would it help to have more examples that showed progression?**

FP1: Yes I felt sometimes we maybe for want of not quite knowing what else to do other than standing them and helping them into standing which is very much supporting their leg in extension or whatever and getting them to stand on their leg we’d kind of do just some lots of exercises but not actually the real stand if you like because it was too difficult to achieve without helping them too much. If you see what I mean.

**I: Yes, which goes back to the question about so if you were helping someone to stand because that’s the next progression that they need to do is what is your view on whether doing that is implicit or explicit or neither. What are your thoughts on some of these functional things and what are they in terms of learning.**

FP1: I think maybe that’s where I potentially was a bit confused by the approach because I would have said that standing somebody and really helping them to stand in extension with their affected leg wasn’t implicit enough so maybe wasn’t the right next stage kind of thing.

FP3: I feel like at that level that’s where it gets difficult to be implicit as well because well it depends on the level of the patient but they might be ready to stand but they’re still not able to fully extend their knee and get a good standing posture and then it’s quite hard to use an implicit approach like yes you can try a buzzer behind the knee but they might not necessarily be able to recruit enough to hit the buzzer with their knee.

FP2: And it’s quite hard to get enough power from the knee extension because you don’t want them like going into hyper extension so that’s actually quite hard to get the buzzer for that so I was going with a target just above the head to stand up to but then that doesn’t necessarily mean that they’ve got the weight over the leg.

FP3: They’re not necessarily getting glutes. There are so many things with standing to try and get right.

MP1: I feel like even if you are sitting to standing if there are a max assistance of two to get them to that point of standing it doesn’t feel like it’s very implicit if you are really having to help them stand. I feel like it’s difficult if they need a lot of help or they initiate really well but there would be no way for them to possibly stand without that much help.

FP3: Yes I feel like the approach is easier if someone is sitting lower limb exercises or if they are standing and looking at stepping and can do some kind of more activity but it’s like the in between stages or the sitting balance between them not having sitting balance and them being dynamically like those little in between bits of movement.

FP1: I think it’s when you first try a stand with somebody who, so that first stand you do with those patients who are longer term where you need two people to help them up or whatever it is or you need somebody on the leg and somebody, those are the ones where you feel like, oh this isn’t really implicit what we’re doing but I don’t really know how else to achieve what I want to achieve.

FP2: So when you put in a token target it doesn’t necessarily work it feels like you wouldn’t necessarily do that unless you were being video recorded for doing IMPS.

**I: So with that example that you’ve all given based on that describe what you would be doing with that type of patient or in that type of scenario and do you think that that is explicit based on your understanding would you describe that as being explicit or just not implicit?**

FP3: In the approach we would take it back to a lower level thing. So we wouldn’t then be able to progress them because we weren’t doing the approach so we would take them back to the lower level task.

FP1: You’d progress them I think but you just would like FP2 said introduce some.

FP2: Introduce a token target.

FP1: Oh take your hip to or take your cross, take whatever it was on there to the table or something so you are getting weight transference, so take the cross to the cross but you are still supporting their leg for them to enable them to do that kind of thing. So you are introducing a bit of implicit stuff there but actually you’re kind of extending their hip and knee for them.

FP2: It’s a bit passive the fact that you’re yeah

**I: So if you weren’t adding in the target, how would you describe or if you weren’t being videoed and you weren’t part of the study what would you be doing and how would you describe it in terms of the spectrum of implicit/explicit learning.**

FP1: I think if the implicit bit was taken out and telling them.

FP2: I think if we could say the word hip it would be helpful.

FP1: Yes straighten your knee, tap your knee and try to get them to straighten their knee or stand up straight and bring their bum forward all that kind of stuff so you’d use all those body parts.

MP1: I think naturally you’d be more hands on obviously because you’d need to be which again becomes less implicit to me.

**I: I’m trying to tease out the bit about being hands on because the person cannot obviously do the activity without it versus what you might want to say to them and where you feel those two things fit in the implicit/explicit approach.**

FP4: If they’ve got a lot to think about if they’re standing it’s like you say it’s hard just to focus on one area because if you have a focus an implicit focus on their knee they’re not necessarily thinking about what their head, their trunk and their arms are doing and they could be fixing through their arms and their trunk could be offline. It’s hard to correct those without using explicit instructions for the rest of it other than.

FP2: Yes because it ends up then with really lengthy instructions which actually you saying that reminds me of what [name] was saying it’s the set-up, she as having to write such a lengthy set-up because she’s trying to get rid of all the compensations by having a ball being kept in one place and then a certain target and so she was finding she was having to write more and more and give more and more actual instruction for the person, you’ve got to keep your, this cross to here and then aim for this, and I think then that became, like then there are lots of verbal instructions to process so it’s kind of defeating the whole point of it. So then you take them back to an easier exercise but is that then really progressing them onto what you actually want because the easier exercise would be those ones working on taking your foot back so you are ready for the sit to stand then you want to actually work on the sit to stand itself as well.

**I: So your instinct is to support them manually but also to give the verbal instructions for what to do if there was a hip, knee, shoulder.**

FP2: Yes so that you can just then focus on one bit I guess a little bit more rather than having to worry about the set up of everything being so non-body part focused.

FP1: And also if they’re needing that amount of support at their hip and knee which a lot of those early standers do then to try and get them to fully if you are looking at above and behind the knee or whatever it’s just too difficult for them so they’re too low level to be able to do some of those implicit exercises in standing other than they take your hip to the table, across to the table or whatever just too many things and they’re too low level for that.

**I: I’m going to keep saying this because I think we’ve flittered over lots of these questions but in different ways so just again based on your experience or if you haven’t actually treated patients on what you think you would look out for how would you know if the implicit learning approach was benefiting a patient in practice. How would you know if it was the right approach to use for somebody. Is there anything more you can expand on about that?**

FP2: Where they’ve been able to achieve higher reps and certainly for one person they couldn’t move the skateboard at all, I keep going back to the skateboard exercise but they couldn’t move the skateboard at all and then they got to the point of doing really high reps with that and then that had translated into function of their improved ability to sit to stand themselves.

FP1: We had goals for them as well don’t we. We carry little goals.

FP2: Yes so while they were achieving their goals.

FP4: I think in some ways the implicit learning is really good for getting the quality of movement if they are doing independent practice. If they’re managing to do some reps themselves and they’re getting the right quality of movement because they know that we set them up set up specifically they might be just getting more repetitions in themselves.

**I: Did you find that, did you set patients up with things to do outside of therapy?**

FP2: We did but not with every patient because lots of them.

**I: For those that could.**

FP2: Yes for the ones that could and the ones that could did actually really, really well.

FP1: They were really motivated. It’s a real motivational thing to have reps being counted and for them to be able to chart it or whatever you did and for them to see that their reps are going up is really good.

MP1: I feel like those patients who have specific exercises as well do it a lot more, you see them doing a lot more than when we’ve given them a leaflet on level two lower limb exercises.

FP2: Because it’s just a couple of really focused exercises rather than however many normally.

MP1: I just think they know and it’s probably a little bit more enjoyable as well but again because they’re doing it to a target or doing something rather than just doing 20 hip flexions and reading it from a booklet.

FP2: I think with a booklet you tend to do fewer reps of each exercise because there’s a lot more exercises whereas with this it was really high reps on just a couple of exercises which seems much better.

FP1: Much better for them to engage I think wasn’t it because they just, I don’t know whether they just got bored or we often give them way too many exercises and it was nice to condense them and just give them a few.

**I: In terms of recruiting people to the study one of the things that I’ve heard at some of the sites is therapists saying oh we’ve got a patient who would be really ideally for the IMPS study or we’ve got somebody but we’re not sure if they’re ideal or not regardless of whether they meet the criteria. So in your view what would make somebody ideal or less ideal for the approach if they meet the criteria for the study.**

FP2: I’d love it if they could follow three stage commands.

**I: Three stage commands.**

MP1: Yes cognition.

FP1: It’s down to that cognition isn’t it mostly.

FP2: And have sensory attention to the hemiplegic side.

FP3: And cognition like there could be a measure for MoCA or something like that they have a certain level of cognition.

FP2: Some of them might have done not so well on the MoCA though but because they can follow three stage commands it might be quite hard to measure it against the MoCA but I know what you mean cognition is so important.

FP3: But in terms of their attention.

FP2 Yes maybe it was just based on the attention markers, yes that’s true because it’s the ones with poor attention as well that have done badly. Maybe just focusing on the attention.

MP1: I think us being a rehab ward of course means that we’ll often some patients we reakkt want they’ll be 15 days on post stroke particularly on rehab I’ve found that there are lots of patients I’ve wanted to recruit but they’ve been three weeks.

FP3: Yes definitely there have been loads that I thought oh they could have been.

FP2: It would almost be better on ESD if it could have carried on into ESD because the ones that do really well are the ones that have actually gone ESD fairly soon and I feel they would have been quite motivated to carry on with the approach but it wasn’t the remit of the study to do that.

FP3: I think it would still be good like it was good that they needed to have sitting balance and they wouldn’t have been ready to start the approach before that and sometimes even past that but once they’ve got to that stage that they were a bit better that it would have been a really good approach for some of them.

FP2: But it’s just taken them a while to get to that.

**I: So to have more sitting balance because it was quite low level.**

FP3: Yes I think more sitting balance. I think once they’ve got dynamic sitting balance I think that’s a good stage and then some of them yes I’ve thought oh and we’ve used the approach haven’t we with some of our patients but obviously they couldn’t be part of the study because they were too far down the line especially some of them that were really poorly at the start and then get better quite quickly but they’re still two weeks or.

MP1: I didn’t really see many of the lower level ones but whether five seconds is too little I don’t know.

FP2: Yes I think so.

MP1: Often it feels like if they can only sit for five seconds they probably can’t tolerate a 30 minute treatment.

FP2: But that was the other criteria so they had to be able to do 30 minutes.

MP1: Yes it was that they had to do 30 minutes.

**I: General consciousness to tolerate it.**

FP1: But also five seconds sitting balance a lot of them can just.

FP2: You can literally prop yourself up.

FP1: Sit there for five seconds but it’s not sitting balance is it and it’d be like, oh yes OK they’ll meet the criteria but really as soon as you lift their arm up they tumble over so yes a bit of dynamic sitting would be nice.

**I: Which is the whole point of doing a feasibility study is that we don’t want to make it too narrow to start with and then wish you’d made it more broader. It’s better to do it the other way. I think you’ve probably covered that of anything else you think that could have been done that would increase the recruitment to the study just talking from your experience here if we just ignore the global pandemic and stroke service transformation and everything else that’s happened.**

FP1: I think some patients we definitely had the patient who withdrew and we haven’t considered necessarily their thoughts and feelings and some patients they were very worried about that they were missing out on what we normally do which if that’s what we normally do then that must be better kind of thing. I think that some patients definitely were worried about joining in the study because they felt that they were going to be missing out on the better treatment. I mean obviously we tried to reassure them and that kind of thing but there’s not much you can say about that.

**I: That’s the nature of any research study is that you can’t.**

FP2: And some people were just too overwhelmed like the people who were really appropriate for it but they were just so overwhelmed by the fact that they’d had a stroke that they didn’t want to consider doing anything extra so we had a few that wouldn’t do it for that reason. And relatives being a bit protective of them as well because they’ve had a stroke and then they didn’t want to do any extra.

MP1: Yes I think sometimes the patient seems fairly keen for it and then the next day they’ve spoken to a relative or whatever and their relatives aren’t particularly happy, they’re not keen that they’re having the treatment. There have been a couple who the patients have been keen for it and then the relatives asked us why we were doing it for example, which is tough.

FP4: Do you think if we could recruit them further down the line maybe they might have had a chance to get their head around the fact that they’ve had a stroke and get used to.

FP2L Yes I guess maybe having a longer recruitment window but then you risk them going home but then if you were going to overlap into ESD it wouldn’t matter if it just carried on into ESD.

**I: The reason for the earlyish recruitment window was that, well there’s a few reasons but the main one being that actually if you don’t recruit someone until maybe six weeks for the sake of argument potentially they’ve already learnt a lot of explicit cues by that point so you don’t really know then when they’re doing stuff where are they focusing, what have they learnt. So if we think standard practice is quite explicit then they’ve had that for quite a chunk and so it just muddies the water a little bit with what they’ve received. So that was the reason for the two week cut off but perhaps that’s particularly if someone has been quite unwell in that time and really hasn’t had a lot of therapy at all maybe there’s some way of having a bit of flex on it or something.**

FP2: I think that’s one of the reasons why trying to get 10 participants should have been quite easy I think when we were basing it at the very beginning we thought actually that shouldn’t be too bad to get but then because we merged and then we had a period of getting the much more poorly patients they were the ones that weren’t as appropriate so we went through a long period of there being no-one that was really appropriate.

**I: It’s been the same everywhere.**

FP2: Oh has it.

**I: Yes.**

FP3: One of the criteria’s other health, isn’t it, other health.

MP1: Oh yes the second.

FP2: People who have old strokes and stuff.

MP1: Yes that one limited us a couple of times.

FP3: Yes missed out because of that.

**I: OK so just last few questions. So just thinking a little bit more broadly in terms of motor recovery, movement recovery, what information so not necessarily in the study but just generally in your service what information do you collect to help determine how effective your interventions are.**

FP1: We don’t do enough outcome measures do we.

FP2: No so obviously we have our goals so whether they are achieving goals and they’re reviewed on a weekly basis and then obviously there’s the Barthel and the Modified Rankin that is done for everyone. And then for balance we tend to use the Berg Balance a lot as an objective measure, which is then carried on into ESD. But we don’t routinely use lots of the outcome measures that were on here and that’s another thing that made it quite difficult because they’re not outcome measures that we’re as familiar with. It was a bit more time consuming and I did think today on my last patient that maybe it would have been quite useful to write down to actually do a format of doing the outcome measures just like a written method because you end up trying to look at three different things and it just felt a bit bitty whereas if it was just this is the outcome measures the order you do it in which makes more sense for the bed mobility components and stuff it would be so much easier.

FP1: Yes much easier.

FP2: Sorry I digressed a bit.

**I: No that’s alright. So collect Barthel and Rankin goals, use goals. What works about that and what doesn’t work – do you feel that’s sufficient?**

FP1: No. We’ve got that bit of work that we want to have more at our finger tips and easy to use but yes the ones we use like you say the Berg Balance maybe a 10m timed walk that kind of thing they are all much higher level perhaps more mobility or on your feet goals it’s harder to find a goal that is specific enough or lower level, which we’re not very good at are we collecting those lower level outcome measures.

MP1: Yes.

FP2: Yes it’s just having them so they are comparable enough because you could do like a trunk impairment scale for anyone it was appropriate to do a trunk impairment scale for but then you’d probably need a different outcome measure for your upper limb and then a different outcome measure for your lower limb and that’s what we haven’t, it’s been on the band 7 agenda for a long time.

FP1: It has

FP2: No time

**I: Are there any measures that you would want to see in a research trial, are there any things that you think are important to measure if there was a future research study that’s looking at implicit learning.**

FP3: We haven’t really got a patient feedback.

MP1: I was going to say yes actually what patients think of it.

FP3: Because you do wonder sometimes with the feedback and not speaking too much and having more quiet sessions like what they are thinking and how their experience is of it. Obviously they’ve got nothing to compare to but just to see what they.

**I: So we’re interviewing patients in the study in control and in intervention sites we’re interviewing patients.**

FP3: It would be nice yes for us to know what their thoughts are.

FP2: Because that’s done a little while after they leave hospital isn’t it?

**I: Mostly just before they go but because of COVID and people going quickly sometimes it’s once they’ve gone home but normally within a week.**

FP2: OK so it should still be quite fresh in their minds.

**I: Yes.**

FP4: Do you ask them which group they think they were under?

**I: Yes, so after the three month measures you ask them don’t you which group they think they were in as well. Describe it back to them and ask them.**

FP1: I know it comes up with all research studies when you are looking at a therapeutic technique but the fact that we’re trying to do sort of implicit stuff here I mean the rest of the time on the ward the 23 other hours they spend on the ward it’s all explicit really isn’t it so it’s kind of also asking them what kind of group they’re in whether they actually demarcate their physio time from when we’re in here doing those exercises or when we’re chatting to them about I don’t know home, discharge, SUMS, all this kind of stuff so it’s difficult for them maybe to know what we mean by their therapy sessions when we spend a lot of other time with them doing other things.

**I: Yes, some patients absolutely are just like I know I was in this group and others have taken a guess 50:50. I think we’re probably there. Is there anything else that we haven’t discussed in relation to the study and the implicit learning approach that you want to share?**

FP1: I think it’s been a really useful thing to do and really worthwhile because it’s created an idea about lots of other things to use and lots of other techniques and strategies to use because not always one thing works for every single patient so definitely the communication as we said the talking, verbal diarrhoea is a point of consideration.

FP4: I think it was quite good for setting up with their own exercises to do in their own time but focusing us on one or two exercises that they could do really well and it was useful to get your head and think in different ways to try and get the quality of the movement and not just them forgetting what they’re meant to be doing and doing it wrong and when there was a target it was quite clear what they had to do.

FP1: I think the one thing that I still found difficult was translating you do the exercises in here and then when you do gait re-education that I found very, very difficult to keep implicit because you can’t set it up. You are walking along the corridor and you can’t necessarily set up an environment that’s easy to make it an implicit experience so I found that difficult and I still don’t really know how you do that.

FP2: When you get to the point where they need to get ready for discharge and you want them to be practicing on the Return and just drilling that that was when it was, then we wouldn’t end up videoing some of those sessions because actually you needed to look at the more functional tasks which is quite hard to make implicit.

MP1: Yes I was going to say it’s probably completely wrong but sometimes if we’re not doing it in the session then probably we’re not doing it we’re not continuing that implicit learning when the camera is off kind of thing. I think sometimes if we’re doing a session with an OT or something and we’re trying as you say we’re getting more ready for discharge the kind of ?[*59 mins 34 secs*] goes out the window sometimes which is really difficult because usually because of time you’ve got to go and do the other things that need doing for them to go home.

**I: Which is perfectly right and actually it’s about you are not, you can’t apply something to every scenario if it’s around discharge then it might not be as relevant to what you are practising with that person necessarily. It sounds like actually I think you’ve all expressed quite similar views, I don’t feel any, would you agree, I feel like you are all quite similar views. No one has said anything that you disagreed with or that wasn’t my experience. How important do you think it is that a whole team buy into an intervention if we’re thinking about implementing IMPS in the future?**

FP2: Definitely and for recruitment as well because one patient got missed because it ended up being OTs and assistants that had done the initial as soon as they got here and then physio didn’t actually see that one until day 15. So I think if the whole team have a greater understanding. The trouble is for us is that we did when we launched it we did do training for the assistants and for the wider because you came in and spoke to the wider team but that was then so long ago now that it goes out of people’s mindset understandably.

**I: Would you think on a broader question that generally you all work in a similar way or do you think therapists are quite individual in how they work with people? Not necessarily you five guys but just generally physios generally.**

FP1: It’s difficult to answer.

MP1: Yes I don’t know.

FP2: Based on when I’ve done doubles I would say we kind of think of doing similar things so based on that.

FP3: Similar things but then I think you get ideas, I think from earlier on maybe in your career when you do doubles you then get lots more ideas as you work with different therapists so I think yes.

MP1: Yes for me again having been six months of stroke most of what I know is probably because of what you guys know in terms of if I was to work in loads of different hospitals then of course everyone is going to be slightly different so maybe as a ward or the specific people who work on the ward it’s probably a bit of a general thing but I don’t think there’s not someone who is so anti-hands on and someone who is so hands on there’s not that really everyone’s quite

FP3: I think if you go to different areas in stroke and neuro you do get different approaches and different levels of speed of progress and things like that. So I think there are differences but generally we’re all working towards the same outcomes in the end but just different ideas for, I don’t know how to explain it, like ABI for example it’s a lot slower rehab and might strip it right back whereas on stroke we’re looking to get people home quicker sometimes so we’ll go for a much more functional quicker approach maybe.

MP1: And even like NHS or private I guess there’s so much difference. Also what’s available to you is going to also influence what you can do.

FP2: And I guess time as well so normally we’d use quite a lot of adjuncts like electrical stimulation, also the therapists assistants doing exercises with the patients going on the exercise bike but when we’re short staffed which happens quite a lot all of those adjuncts go out of the window and their only treatment session might be that 45 minutes of the IMPS session. And then if you have to work on discharge planning with the OT then that will be a joint physio OT and that’s their only 45 minutes of therapy within that day.

**I: So dictated by those constraints and time rather than you all being very different in what you might do.**

FP2: Yes I think if we all had all the time in the world we would do quite a lot extra with the ?.

FP1: Yes definitely.

FP2: But you end up just focusing on the immediate, like if you look at our list now it will probably be all full of sitting balance, gait re-ed and parallel bars because that’s the main thing that you need to do with that patient to get them home. You don’t necessarily get the time to do all the extra bits that might go towards [*1 hr 4 mins 25 secs*] [improving that?]

FP3: Yes bed exercises or chair exercises aren’t always getting done at the moment which is a shame because it would add to their functional bits but.

MP1: Even just the set-up of it we try and get them for the nurses to be able to go and set them up on the skateboard for example, I know we keep on using the skateboard, but set them up for that and then again when the nurses are busy and we’re busy that’s never going to happen, which is difficult because again for those lower level patients they can’t set themselves up necessarily particularly the sitting balance work.

FP2: So slight variances in therapy approach but more limited by time I would say. I think lots of people have got lots of good ideas it’s just whether we have the time to do it.

**I: Lovely. Right I think it’s a minute past 2pm. Let me turn these off.**

**FOCUS GROUP 5**

**I: So mainly for the purpose of the audio recorder is it alright just to go round and introduce yourself and what your role is and which bit of the service you work in.**

FP1: I’m [name]. I’m the clinical lead physiotherapist covering across the acute ward and the rehab ward.

FP2: I’m [name] and I’m the team lead physio on acute stroke.

FP3: I’m [name] I’m the team lead for stroke rehab physio.

FP4: I’m [name]. I’m Band 4 physiotherapy assistant practitioner.

FP5: I’m [name]. I’m one of the Band 5s. I’ve been working on acute stroke.

FP6: I’m [name] I’m one of the Band 5 rotational physios on acute stroke.

**I: Lovely, thank you. So you all did the IMPS training that was relatively recent. Some groups I’ve been to its two years ago that we started with them so actually your experience is a little bit more recent and great that you’ve got some current patients enrolled as well. Has everybody here been involved in delivering some IMPS intervention to people on the trial or not?**

FP: No I haven’t been involved in delivering.

**I: OK. So you guys have.**

FP: Only this week.

**I: Just as you are about to take a bite of cake. And you and FP6 hasn’t, OK that’s fine. So you can speak from your experience or just actually from attending the training and what your thoughts might be. So I’m following a little bit of a topic guide today just to make sure we cover the things I want to cover. But just to kick off I’m just going to ask you to think about when you first heard about the research trial, which presumably was from FP1 who might have told you about the study. If you can recall what you thought about the study when you first heard about it. Whether you had any general thoughts?**

FP5: I think from hearing about the way we deliver the IMPS treatments and you have to hold back on your prompting and not give too much feedback felt quite counterintuitive to me and that felt like it was going to be quite tricky to deliver, which I found in parts but I’ve also managed to do more so than I would with my normal treatments. It’s kind of holding back that prompting and asking people more so how they are feeling and then responding to that following it. But I think from the training I was like I think this is going to be quite difficult.

**I: So it did feel like it was something different to what you might normally do?**

FP5: Yeah

FP2: I think I was the same, I think I’m probably completely the opposite end of the spectrum and give lots of feedback probably during my sessions so knowing that I have to be completely the opposite end I thought it was going to be hard, not easy to do.

FP?: I think that was my thought as well that the concept made sense and it felt like it should work and that perhaps altering the delivery of exercises a little bit felt relatively easy but it was the holding back on the verbal feedback and the changing of instructions the feedback that felt like it would be most challenging for me.

FP1: I think when I first heard about it I was really excited. It really feels like as a study it fits really well with my values about working as a therapist and I think in my experience of seeing patients and how quickly they become institutionalised and how they lose control and direction of what’s happening to them so rapidly it just disappears away from them and it felt such an opportunity for exploring how to give that control back to people. Interestingly I think we’ve had a couple of people who have had a stroke going through the service who have been really resistant to handing control of what’s happening to them to their to us as a team. They’re just really sort of like no, no I don’t want to become a standard patient. It feels to me so much like the future of being really aware and insightful about where does the control for decision making sit and choices about motivation.

FP5: I think I gave more thought to the recruitment of the patients as well and I think almost like patient’s personalities and what motivates them and drives them. I think when I’d hear about patients who had cognitive difficulties or communication difficulties I’d automatically think that they probably require more prompting so maybe not engage as well with it as some of the patients have.

**I: So that’s interesting so you expressed that there was something about initially knowing that you were going to have to do something different and that might be hard but also you were thinking about what it might mean for patients and how it might or might not work for different patients.**

FP5: Yes.

FP3: I also found those different words that you try and use, so just not giving people as much instruction just trying to use one word and not trying to give them a whole load of sentences and being able to correct them. I found that the hardest thing initially.

FP6: Not that I’ve done it but from a training point of view I think when we were looking at the training and then looking at the training and then looking at examples I think it’s definitely made me think more creative rehab wise and actually making it a bit more fun for patients because you are giving them a bit more of a target, you are pointing at things, you are pressing things, which actually maybe I wouldn’t have chosen to use if I wasn’t doing the trial,

**I: You’ve all reflected that you felt that it was something different to what you normally do as in you probably would normally give them a lot of information or instruction. Have you got any thoughts on why that is? Why we therapists tend to work in that way?**

FP3: I think we often are very quick to try and correct things. I think my initial thought was if somebody is standing in a certain way and I wanted them to do it in a different way I’m then always trying to correct that. But I think with IMPS we’ve had to pull back on that a lot more and only give them say one instruction and then it’s up to them to try and correct things a little bit more. That’s probably the hardest bit for me.

FP1: I was really aware and we’ve talked about it a bit for 006 in our session yesterday about how allowing somebody to do something that looked really bad. She was just sort of hobbling along and not really standing up straight but it was her first walk. How often you are aware of other people around you and wanting to make a treatment session look good that I found it quite difficult to allow somebody to do something functional in such an awful way.

FP2: I also wonder if there’s something about having a treatment session sounds and I sometimes think about in treatment sessions there’s not often a lot of silence and not talking. But actually if I’m learning a new skill or task actually quite often I need it to be quiet to concentrate and focus but actually I don’t probably often give my patients that because I’m too busy talking at them. So I think that that change of letting there be more silence in the sessions is quite different.

FP3: It’s almost uncomfortable sometimes isn’t it.

FP5: I think I was talking to FP1 yesterday so I only graduated last year and all the things we went through at University was having a look at what normal movement is and we were saying if something is functional they may not get back to a normal pattern of movement so we’re not actually looking for normal movement per se we’re looking for how they manage it and how they problem solve themselves.

**I: That thing about how can we label it quality of movement or normal movement how do you feel when you’ve been trying to apply the IMPS principles do you feel that’s been more difficult then like that example you’ve given with the lady walking badly.**

FP1: I think just because it’s so recent in my memory the session was yesterday that you asked the question didn’t you FP5 about do you think people get more compensatory. What was the term you used?

FP5: It was almost like getting into bad habits.

FP1: Bad habits, yes. Do people get more bad habits if they’re learning this way.

**I: What are your thoughts on that?**

FP5: Well me and FP1 kind of had a discussion and said it depends how if we can, we are always inclined to try and correct it and get them into the pattern of movement that we have in our head but then problem solving it and learning it in a different way to what we would but they’ve had a stroke and things are going to be slightly different and I think the way this lady specifically that we were working with yesterday she was really pleased that she’d managed this afterwards and it wasn’t that I think she would have been just as pleased if she’d managed it in the exact way that we wanted her to. I don’t think trying to correct that habit as we were perceiving it at the time would have changed how she felt about the intervention. I think she was just chuffed that she’d done it.

**I: Do you think it’s harder, I’ve had other people reflect this to me, feels harder to correct the movement or compensations in an implicit way because the natural thing is to correct them verbally maybe. How have you found trialling actually still achieving the right quality of movement but sticking with the implicit?**

FP3: I think the way we’ve probably used, we’ve used the buzzers a lot. There was a chap a little while ago now who really flexed at the hips so we’ve been using buzzers on say the front of tables with tape around to try to get him to standing up tall but then pushing the buzzer against the table. So trying to use the buzzer and the tape to try and correct it without using those verbal prompts, so it’s really hard. Your natural reactions want to correct it verbally.

FP1: I think I totally agree with that, it feels like it’s made us adapt the task and be aware actually we’ve given a task that’s too complicated, that’s too high level and therefore if we’re not producing the movement we want we’ve got to drop that down and do.

FP3: I think we’ve started doing that a lot quicker. I think before I would have maybe given it one or two sessions to see if we could get through that. But I think we’re dropping the level down much quicker when we realise that they can’t do something.

FP5: I think I would struggle to correct that within a session if I was doing something and say I thought that somebody maybe at an increased risk of falling from the way they are doing something and I probably would end up stepping in and maybe saying something that wasn’t an implicit way of correcting it and then I’d try and reflect on that for the next session of how I could try and change that. But I don’t know if I would be able to correct it within the session that we’re doing there and then.

FP1: We had that chappy in D1 didn’t we.

FP5: Yes I was just thinking about him.

FP1: That we were assessing and then he didn’t recruit because I can’t quite remember why he didn’t recruit.

FP2: I think he went home didn’t he.

FP6: What did you say, sorry?

FP1: I couldn’t remember why we didn’t recruit him.

FP6: He had had a previous.

FP1: Ah that was it.

FP6: Yes so we weren’t able to do but we did do a session, we practiced didn’t we.

FP1: Yes and I think he showed an improvement in his function because of the activities we practiced with him.

FP6: Yes because we stood him didn’t we and I think we were getting him touching targets and actually I think you got him to reach to the side by touching something, I think it might have been the buzzer or something, which then actually corrected how we wanted him to stand.

FP1: Yes he was a really nice example.

FP6: Yes he was.

**I: It does require a lot more thought doesn’t it, that’s my own experience as well like how am I going to get this person to move in the way I’m trying to get them to move without just telling them to do it.**

FP5: I think that probably reflects in some of the videos where you can almost see us stop mid-sentence and then just leave it.

FP3: I think it takes more preplanning. I always find that I’m just sitting down for a couple of minutes at the beginning and just think what am I going to do rather than just, sometimes you just go with the flow a little bit don’t you. But I think I have to plan further ahead than just going into a session and doing it.

FP2: I think it’s having those regressions to hand and progressions if you are not getting the outcome that you are expecting.

**I: Do you think that would improve over time because obviously actually you’ve only seen a small number of IMPS patients in the grand scheme of things haven’t you.**

FP2: I think you would probably develop a bank of activities and progressions and regressions that you probably end up using more frequently and it will become more slick I would have thought easily.

FP6: I guess it’s a constant transition as well between our IMPS patients and our non-IMPS patients and going between them both especially for you guys on rehab.

FP5: I think as a background for me and FP6 as well we’re rotating through so we’ve only been here for six months looking at stroke overall so now learning and then changing and then trying to adapt new things is obviously all new to us.

FP1: I was wondering FP4 whether as you come into physiotherapy as a new profession have you seen a difference between the two treatment approaches like IMPS versus non-IMPS? Or actually is it all a set of skills that you can see would be appropriate sometimes or not. Would you say oh I’ve seen something quite different when I’ve seen an IMPS session?

FP4: I don’t think so.

FP1: No, oh that’s interesting.

**I: With the patients you have seen or maybe you’ve not seen enough but just thinking about it hypothetically are there any groups of patients you feel this works better with or is more difficult with or doesn’t work so well with. Have you got any thoughts on that?**

FP3: I think you almost just sometimes get a vibe of patients that need a little bit more feedback or prompting but then you also get those patients who just need quiet just to get on with it. I think those bigger strokes sometimes it’s easier to have a little bit more feedback but then we haven’t been doing this for very long so.

FP2: I did a session yesterday that really surprised me, it wasn’t an IMPS patient but we just thought we’d try and do an IMPS style session.

FP3: Yes that was really successful.

FP2: Someone who was really distractable, wasn’t really sure how well they’d get on and actually when they were just given a task of tapping the green triangle 10 times they were just completely absorbed in it and completing it. It worked much better than I had thought it would have so I wonder if perhaps we’re getting a little bit thinking about oh they’ll be a good IMPS person and perhaps narrowing that pool down again.

FP3: Maybe using it a bit more on a variety of even the ones that aren’t in the trial.

FP1: I was wondering whether possibly the combination of dementia and stroke because I remember being very enthusiastic about the lady who is in F2 who ended up going to a care home and I was enthusiastic about recruiting her and then we didn’t recruit her.

FP?: Because of her cognition.

FP1: Yes. She had really difficult tolerance of any sort of activity. She was very frail. I think it was that combination whoever she was it was a combination of frailty and therefore actually difficulty actually engaging in rehabilitation whatever type of rehab it was. Plus the fact that she had no memory session to session of activities so the whole meaningfulness of therapy was quite limited. Actually I think she from what I recall she didn’t stay long because then she was identified as for placement. She wasn’t identified for any rehab, she didn’t respond really to any let alone IMPS.

FP2: On acute patients that we thought they’re not going to be for IMPS at the moment has definitely been the group that have been more acutely unwell and just tolerating very short sessions. There’s a gentleman at the moment who probably for the first fortnight really wasn’t getting out of bed a huge amount and we were just having to take it quite gently because of his medical status so that’s definitely been one of the big things that’s been a barrier to patients participating.

FP5: I think it’s patients who have a stroke, well because we were talking I think at the beginning about are they staying in hospital long enough for the recruitment process to occur but then I think also patients who have a larger stroke and are within that group of staying slightly longer also have quite low mood, don’t feel like they have quite as many goals. Whereas the two patients I've seen this week have goals set out in their mind and they don’t really need to be prompted to think of those goals. So I think when I’ve been recruiting people and they’re talking about their goals going forwards then they’re more likely to be motivated to engage in therapy and therefore need less prompting.

**I: Do you think the acute ones, so they weren’t able to be recruited because of the criteria for the study but in terms of just the principles of using more implicit learning do you think that fits with someone who is quite maybe early after stroke and perhaps has had quite a severe stroke where maybe you are just working on things like sitting balance. Or do you feel that would be harder to apply or less relevant in that group if you put the study protocol aside?**

FP2: I think the principles I think we would be able to apply to that early stroke patient but it would be having to keep the tasks much simpler, potentially thinking about the need for physical assistance in somebody who might be very early after a stroke. Also just thinking about the complexity and how long the sessions would be. So I think you could apply the principles but it would need more modification I would have thought.

**I: And that raises a question that’s come up in some of the focus groups as well around handling and facilitation. So in the training that is not something we are really looking at so use the principles but quite clearly in your patient groups you are going to need to be hands on sometimes and I just wondered what your thoughts are knowing a bit more about implicit learning about where that fits, where handling fits with it. There’s not an answer to that in my head that I’m trying to get at it’s just if you are manually supporting somebody where would you think that fits with their style of learning?**

FP3: I did a session with you at the beginning of the week and we were really trying although this chap hasn’t got perfect sitting balance he does have intermittent sitting balance and just really trying a hands off approach, haven’t we. But I think I found the principles easier with those slightly more impaired patients. I think I found that when they start to be walking and the higher level tasks I think I found it harder to put those principles in place so when they’re looking at sitting balance we’re using the buzzers, the tape, the targets. But then moving those principles forward when people are more able I think I’ve found more difficult. But handling we are trying to keep it much more hands off as much as possible. I think it worked because he had some good saving reactions. When you are doing lots of handling they almost fall back into you sometimes don’t they. It worked really well with him at the beginning of the week.

**I: Is it more difficult those high levels because you are trying to fine tune, are you thinking gait and things and actually how you do that without.**

FP3: I think so yes and having those ideas of we’ve seen the rehab team especially we’ve had a few come through now so we’re starting to get some ideas of our own and being able to branch out a little bit more but the higher level patients it’s still trying to build up that box of exercises I guess to try and use. But it’ll come.

FP5: I think I’ve almost found it the reverse like with the higher level patients where you don’t require physical handling it’s easier to just give them a task and you know they are problem solving it because you see physical movement, whereas sometimes with patients who maybe only just have flickers in their leg or something like that you’re not sure whether they’re not problem solving the task or they physically can’t move it until you ask them what they’re trying to do and what they need help with. Whereas I think although I would agree with FP3 in terms of the people who are at a higher level it’s getting all those ideas and almost trying to ignore trying to find normal movement. I think I can follow the verbal prompting a bit better with those patients than I can with patients who require physical assistance.

**I: I can’t recall of the patients you’ve had enrolled in the study have you had any patients who have got language deficits or cognitive deficits and have you got any thoughts on how this fits with those groups of patients?**

FP1: 005 who I’m doing.

**I: I bet you’re the only one who can remember them by number.**

FP1: She had aphasia. She wasn’t a really profound aphasia but.

FP3: I think she was probably the only one with no speech wasn’t she. I think everyone we’ve had has been fairly cognitively intact. I think the very first ones were.

**I: Or more from your general experience if you were thinking about somebody with cognitive deficits or communication deficits.**

FP1: I suppose it’s when you get a situation where you’ve got an aphasia and apraxia combo possibly then that becomes harder.

FP2: I think if somebody would be able to copy then that would work well but if somebody might be receptively or apraxic as FP1 said and would be unable to copy tasks then I think that would probably become more challenging to implement if they couldn’t understand the instruction and they couldn’t copy you then that would be difficult to not have to be hands on in demonstrating a task to them.

FP3: Maybe the foreign use of the buzzers that’s something they’re not especially if somebody cognitively impaired.

FP2: They might tend to be more functional.

**I: Do you feel then if you were needing to be more hands on or be more functional would you see that as being more explicit do you think?**

FP3: I think yes now having done the IMPS thing.

FP5: I would say so too because you are kind of giving that hands on feedback almost correcting it to what you would like in your mind’s eye.

**I: It’s interesting because I think the jury is out as to how someone is learning if you are doing that. There’s not a wrong or right answer but it might depend on actually what you are saying as you are handling. So actually if you are helping someone to move but you are not saying just feel what your knee is doing right now and don’t let your knee snap back but you are actually just helping then whether that’s implicit/explicit/neither. I don’t know. So it’s probably not the handling as such it’s maybe the context of it all.**

FP1: A little bit like as I say with the two that we got at the moment that feeling like you do the manual handling to get them onto the plinth and then you start the session and then you do the IMPS activities and then you stop them and then you do the manual handling off the plinth. They become two discrete activities.

FP3: And when we’re doing the transfer one and I’m often not using the implicit you give them much more instruction don’t you whereas even after the session when you’ve just gone through all of that you are still then giving lots of feedback it’s really hard to swap between the two.

**I: And it is a little bit of a false scenario when you are doing a research study and you are being video recorded and you are really consciously thinking about what you’ve been asked to do.**

FP3: I think maybe if we left the recorder going whilst doing the transfer maybe that would prompt not to slip back into those habits.

FP5: I think you were saying yesterday FP1 when I was doing the session that I managed the implicit instruction better during the treatment session than in the transfer where I probably included more prompts.

FP1: Yes that’s probably what I was thinking of. I was doing the same I think, suddenly it was like oh we’ve got to organise to do this task isn’t it because the task is so alien to actually doing a stand transfer with an electric Arjo is just a very alien thing isn’t it. It’s nothing you’ve practiced before. So the technique needs to be explained.

**I: Yes and you do that all the time with people don’t you so also your habit of how you explain that because you are constantly doing it. But also it doesn’t necessarily mean that description is explicit it might just be somewhere in.**

FP5: I think between treatment sessions it maybe that I explain it more in an explicit way the first time you use the electric stand aid for example and then the patient becomes more familiar with putting on the belt and attaching things and then you don’t have to give that prompting next time. But that’s not to say that in the follow up session you are doing it implicitly you’ve just already given them the instructions another time.

FP1: They’ve learnt the task. I thought it was a shame. Sorry.

**I: I was just going to say I think the other thing is just remembering it was all about creating bias towards implicit learning as well so it didn’t mean that every single word you said had to fit with that. It is OK sometimes you need to maybe give a cue that is explicit, it’s not working in any other way or you’ve got to show how to use a piece of equipment. So that’s the reality of looking at it in clinical practice.**

FP1: I was just going to say I thought it was a shame that it didn’t include the early supported discharge service because I’m sure that, my assumption is that they work quite implicitly anyway because they are setting up tasks in the home environment so almost it would tune very easily and also I think you just said FP3 didn’t you that you thought actually it worked better with more dependent patients and it was harder to do with the people who are up on their feet with higher level tasks. I almost had the converse and I wondered if those would be easier to do with that group.

**I: Because a functional task is naturally a bit more implicit do you feel?**

FP1: Yes. Saying these are the exercises that you are going to be practicing but you didn’t want to include a home based exercise with them.

**I: At this stage it was just sort of limiting it to a because it’s a pilot study just something that was defined and manageable but I think you are absolutely right and as time goes on people are in hospital less and less and less aren’t they and the bulk of their rehab is happening out of hospital so probably if we were doing a future study we would want it to continue through into ESD to look at it in that setting. Yes I agree. Going back to something somebody said earlier about patient preference as well and you alluded to different patients might respond in different ways or might like to learn in different ways and I just wondered what your thoughts were on that and if you have any way of knowing what a patient’s preference might be. So not necessarily someone in the study but just more broadly. Do we pick up how people might prefer to learn.**

FP3: I think you sometimes get an idea from people just in conversation don’t you. You get those some patients that tell you everything before you’ve asked everything and you’ve got the other patients who you have to delve a bit deeper and a bit harder into their likes and dislikes and what they do at home. I think sometimes you can get a vibe from that which way it’s going to go and how much feedback and support they’re going to need. I think sometimes that’s how I’ve judged it.

FP2: Also trying different things with people, trying different approaches with them whether it be very vocal and enthusiastic and trying to really be very positive and motivating them or whether or not you are trying to set them up with a task and see how they respond and then judging the outcome that you’ve got from altering your approach as a therapist particularly if you think perhaps someone might not be achieving the task that you set up or doing as well as you might expect trying to think well is it me, is it the instruction I give them or the task. If I change that is that going to change the outcome. Also obviously talking to other people who have seen the patients about you seem to be getting on really well in the sessions, how are you approaching it, how are they responding.

FP1: Was there much in the courses, you’ve both been through the physio course recently about learning styles, about how people learn.

FP5: I think there was in our first year we looked at what type of learners we are and what our role would be in a team but I can’t say there’s anything that sticks in my mind now particularly.

FP6: We did the psychology score thing to say what type of, like whether you are a more outgoing person or you are more reserved. I can’t remember what it’s called.

**I: Like a personality trait type thing.**

FP6: Yes that’s the one we did.

**I: Do you find again whether in the study or otherwise do you often find patients leading that in any way asking for more feedback? Have you experienced that.**

FP2: Sometimes, well only if I’m asking a patient how I think they did or how they experienced the session, what they thought I frequently find that they’ll look to me for what do you think, did I do well. And they seem to value the therapist’s assessment of the session kind of over their personal feeling of how successful the session was. So whether that’s because the [*34 mins 36 secs*] are possibly too therapist led or as FP1 said earlier the patient doesn’t have much autonomy over what goes on in the sessions so therefore the control is with the therapist and so the therapist assessment is more important. But certainly I think that it’s that how did you think I did if you think I did well then I’m happy rather than yes I’m really pleased with what I achieved.

FP3: It’s really hard to then bounce it back to patients though isn’t it if they are asking you then how do you answer back to them and say well actually how did you do. I think I find that really hard.

**I: Have a stand-off, well how do you think I did, well how do you think you did, well how do you think you did.**

FP6: They can be quite negative patients as well can’t they. Oh I’ve done rubbish and actually from our point of view we think they’ve done great.

FP2: Particularly if their goal is quite, if they’ve got a long term goal of walking, walking to a toilet and they’re at quite a low level at the moment then trying to get them to understand that the short term goals are so important in achieving that goal because quite often I find patients just won’t see that necessarily. If they’re still not walking to a toilet then they haven’t achieved it so they’re not happy.

FP4: They can be quite hard on themselves.

FP5: Give themselves quite tough time frames of when they want to achieve things by and then being disappointed or not engaging as much when they don’t achieve that short term goal or it takes them a bit longer.

FP3: I think walking as well is always that big goal isn’t it and patients often find it hard that we have to do this, this and this to be able to get to the walking. I think sometimes when you’ve had a big event like a stroke I think it’s hard to strip that all back to realise that you have to do those things in order to be able to walk. I think that’s what we struggle with a bit at times.

FP6: That’s like the guy we saw yesterday, when I took the bed back he was like I can stand up and get to my bed. I know he’s a bit cognitively but it’s like unfortunately at the moment you can’t and it’s the steps in between that we need to achieve first before you can do that but that’s what we want to aim for. But obviously at the moment you need to do it safely.

FP1: I helped him to stand this morning. And then he told me, there wasn’t even any point in asking him how he thought he’d done because he told me was doing that every day. He was standing every day when he wanted to get to the toilet except people wouldn’t let him get to the toilet.

**I: It’s interesting because I’ve been interviewing patients as part of the study who have been at the control sites and the intervention sites and regardless of which site they’re at there’s a really strong theme of, I talk to them about their experience with their therapy and it’s always positive, it’s good and the therapists are really lovely and I know it’s helping me. But any more than that and they can’t give me much more than that so it’s like how are they really feeling about their progress or how do they feel in therapy when they’re working on things. It’s always quite superficial and it’s good – it makes you wonder about that autonomy and where it all sits really. Maybe that’s OK and that’s quite appropriate at this stage of someone’s recovery.**

FP6: I wonder as well because obviously we think about that more whereas patients like from our training and our experience that’s what is automatically in our mind whereas someone that’s just completely different wouldn’t – I’m like yes that’s fine. They don’t actually think about the details.

**I: No. They can often recall very little about what they’ve actually been doing either.**

FP1: And isn’t that when you hear about that doesn’t it make you really question or wonder what that experience that they’re going through is really like because you kind of think as you are seeing them and working with them every day you sort of think I understand what you are experiencing but probably it’s probably miles away from what it is actually like being inside them going through the experience.

FP5: I think as well a lot of people don’t really, so stroke or otherwise a lot of people don’t really expand. If someone asks you how you are and you are like I’m fine thanks. You’re not giving them an update on everything that’s going on in your life. I think a lot of the time as humans we don’t necessarily reflect that much on what’s been going on in our lives people just are looking forwards rather than looking at how they’ve done so far.

FP1: Or that you are not experiencing it physically, that you don’t really centre into your body very much like the chappy who thinks he’s been standing every day and he could easily stand up to get into the chair. Do people actually integrate the experience of what their body feels like.

FP3: I think we see that with the goals as well with trying a lot harder to get patients to do their own goals especially if we’re having a break on the plinth or something and we ask them what do you want to achieve. It’s always quite a hard conversation often that’s always we try and make it patient led but you often don’t get an awful lot. So it usually becomes therapy led doesn’t it the goal setting however much you try.

**I: They’re looking for guidance.**

FP3: It’s hard.

**I: Just thinking back to the IMPS training when we did the training was there anything about the approach that you felt was unclear, anything you thought not really sure what we’re being asked to do or how I’m going to do that.**

FP3: I think it just felt like we needed to give it a go. I think initially when I first saw the video there was a little bit of panic inside of how am I going to carry that through. Once you get going and you see the examples of some of the sessions it becomes much clearer.

FP2: I think one of my initial thoughts was probably about the time. Time to plan the sessions, set up. I think at the time that we had the training most of our sessions were for whatever reason happening on the ward rather than in the gym so just thinking about the time factor of all of that and set up and that was my initial.

FP3: I think this week especially because we haven’t got the gym we’re doing it on the ward having the video camera going and being very conscious about what people are saying around you. I know it’s only going to you guys but we had a situation in the gym the other day there was only one patient in the gym and his family had arrived to pick him up but they were waiting for medication or something and we wanted to start the video camera but they were there talking. They did leave and they were fine with that but I think it’s just being conscious of who is around you as well when you are recording.

**I: So some of the logistics around actually being able to do.**

FP3: Unless you’ve got a proper therapy space.

FP5: We had an early discussion as well about what our ideas may be, what equipment we wanted to incorporate versus equipment we had already and how we were going to use what we had or whether we can source any more as well. I think that was quite early on.

**I: So those sorts of conversations, so when you’d done the training and you knew you were looking for patients was it something you talked about as a team and what kind of things were coming up in more informal team conversations.**

FP3: I think we bounced some ideas around about specific because there was some of the team that had done this, was it last year you did it or the year before?

**I: Must have been going on for years.**

FP3: There was still some of that team around that were bouncing some ideas around and talking about different interventions that we could give a go because it was quite a new team so a lot of us didn’t know anything about it. So that was quite useful.

**I: Just sort of brainstorming and sharing ideas.**

FP3: Yes almost have like a toolbox of ideas just to get going basically.

FP1: I thought the photos were nice. That was quite an explicit way to just immediately see, ah yes I could try that.

FP3: Was there a video we could watch.

**I: Yes there were videos in the training and pictures of them in the manual.**

FP5: I think we started with an idea as well of giving each one of us a specific functional activity so whether that’s a sit to stand or a sitting balance or a mobilising and then thinking of implicit ideas around them I think with how busy the hospital is that kind of got a bit lost and a bit carried away.

FP1: I forgot we did that.

FP5: Yes. So I know from my end I was given mobilising and didn’t necessarily think about ideas for that in the end but I think that was a good start of ideas that would have come through quite well.

**I: So definitely getting brains together felt like a helpful thing to do. Was there anything that came up in conversations just amongst colleagues in terms of more sort of challenged what you are being asked to do.**

FP2: [name] was like how am I going to shut up, how am I going to be quiet. She is so chatty.

FP3: She’s so verbal isn’t she.

FP2: So it seemed to be the, gosh we’ve got to be quiet I can’t. That seemed to be something that we all felt conscious of

FP6: It’s like [name]’s opposite.

FP5: Most people I went into training with we all wanted to care for the patients, we don’t want to leave them to struggle too much with certain tasks. It’s like how long do you allow them to try and accomplish a task before you can see they’ve just about given up with it and giving them that physical assistance to correct it as well. I think that was quite difficult just to allow somebody to fall to the side a little bit before helping them back.

**I: We’re nearly there. This has been a pilot study so really we’re learning about all the things you are talking about like how do we put this into practice and can we and can we recruit patients. But if in the future there was a beautiful study that showed actually working in a more implicit way is beneficial for a certain group of patients or whatever what do you think would need to happen to get that widely adopted into practice. How do we get that? What would lead to people changing their practice in the future do you think?**

FP3: More staff.

FP5: I think you’d almost need an implicit learning champion at each centre so like have somebody who goes through slightly more intense training and then working with them with patients over a couple of months and things like that just to get the practice in. But then you have the prompting from these champions you are working with so that you can go, oh yes no I mustn’t do that and trying to change behaviours in practice I think would require somebody to be with you for a fair few amount of those sessions in person.

FP1: I think having the training in the university setting before people have had that much patient exposure would be really appropriate. I really noticed over the two years that the band 5s who got involved with patients were very engaged and committed and free with them and we’ve had somebody who has retired who had been working as a neuro therapist for a long time and I would say if you compared the two she found it a real challenge to try and take on board the techniques compared to say the band 5s coming through who could really easily and flexibly do, ah yes I can see what I’m doing now. Partly because what it does is it gives you a toolkit of ideas once you’ve got that and you’ve started building on it you’ve suddenly got a lot of ways of working with patients.

FP2: I think that the more you use an approach the more automatic it will become and then the more other people around you will learn from it and then it will just become more widely embedded. I think just our mindsets changed a lot I think even in the relatively short time that we’ve known about IMPS and have been trying it.

FP3: We’ve been using each other’s exercises and ideas but if we had a toolbox of exercises or something just to bounce off of would be really useful initially maybe when it’s kind of oh my God what am I going to do. Having that toolbox of exercises initially would be really helpful. Then you kind of expand on that don’t you and make your own exercises.

FP2: So possibly some sort of resource whether it’s online or videos or something.

FP3: Just to get you going.

**I: More examples of perhaps the common things that you might be working on.**

FP3: I think because you are being filmed as well you are like oh I must know what I’m doing.

**I: Well on all the films**

FP1: Almost like a physio tools type of resource isn’t it, oh there it is you can open it up.

**I: Actually from all the films from all the hospitals we’ve got loads of videos there must be lots of good examples in there of what people have done that we can probably pull out. I was just going to ask you said people around you and how much do you think culture within a team or a service matters to something being adopted?**

FP2: I think it’s important. I think that if a team is flexible and willing to try new things then they’ll give it a go but if somebody is quite fixed in their approach and they know it works for them and not necessarily willing to try other things then that is going to have a knock-on impact I think to everybody else.

FP3: I think it’s probably that balance as well between a therapist and the nursing staff. Therapists are normally quite flexible aren’t they, always willing to try anything but then as soon as they’ve gone back to that other environment with the nursing staff you lose that implicit learning don’t you and you are being told exactly what to do, when and how. I guess it’s a balance of the two isn’t it.

FP1: Do you think we’ll keep on doing it?

FP3: The IMPS? I think so yes. I think the patients have enjoyed it with the different buzzers. It gives you a bit of light heartedness as well doesn’t it when you’ve got, yes definitely. I think it will become more natural as well for us won’t it. It’s definitely becoming easier already compared to when you look back at the first patient we had.

**I: Just picking up what you were saying about the rest of the MDT would your observation be that nurses work in an explicit way do you think generally?**

FP3: Yes. You only have to stand outside the curtains don’t you. Yes exactly where they want their legs or where they want their arms and how you are going to use this piece of equipment. We do it as well when we fall out of it but I think it would be hard to change their way of working potentially.

FP6: Especially when they have so much else to do.

FP3: They’re so busy, yes.

**I: I suppose finally thinking about, again not in the context of the study but in just your service generally when you are looking at their motor recovery or functional recovery what information if any do you collect to determine how effective your interventions are and are there things that you think we should measure in a research study.**

FP3: We use the outcome measures don’t we.

FP2: Yes we tend to use the modified Rivermead don’t we. We do the Barthel weekly in MDT as well.

FP3: Probably the Rivermead is more appropriate for physios isn’t it rather than the Barthel. The Barthel tends to be used more by the OTs doesn’t it. We use the Rivermead as a measure.

FP1: We’ve also had the impact of MyCare and a lot of problems with the set up.

**I: Is this your electronic records?**

FP1: Yes. Which has had a big impact. When did MyCare start?

FP2: October.

FP1: Oh a year.

FP2: It’s so depressing.

FP1: So all our impairment motor scoring we’ve been struggling with a system that isn’t really fit for purpose because it was set up as a system across physio rather than specific to neuro so it’s got a very musculoskeletal bias. So that’s caused us problems. I have to say the combination of Rivermead, Fugl-Meyer, lower limb and SwePASS I love it, absolutely love it. So what I’ve done is created a, FP3 will say she has, but I’ve created this flow sheet where you start off in sitting and go sitting to lying, you go supine activities, you go side lying activities, side lying the other activities, up into sitting activities up into standing. So it all lists for the three measures what you do through the functional ability [mobility?] flowsheet and what was lovely was with [name] was seeing her going from a 25 score of the total score to a 50 score to 100 score with her three sets of measures. I’m really keen, I’ve said it to FP3, I don’t know if FP2 will also be into it I’ve not really talked to FP2 about it, but I would like to use that set of measures on an ongoing basis to give a numerical score. I think it might have the opportunity to say ah this is an 18 score which was [name] who you spoke to versus this is a can’t remember what [name] got.

FP2: 40 something.

FP1: 40 something, yes, and actually prognostically that’s really interesting because you can see that say [name] is really having such a struggle to get through to any sort of standing activity whereas she’s already up on her feet and walking. So prognostically it’s been really helpful. But also the way it monitors change because with [name] it was a real feeling of oh she’s got a bit better and then you did the measures and though, oh my word she’s really changed on these measures.

**I: I’m just speaking from my clinical experience now we tend to measure, use fairly crude measures don’t we like the Barthel which are perhaps important for showing when someone has made a big improvement but I’ve found doing the Fugl-Meyer even useful and never would have done an impairment measure of that detail before because sometimes we think is someone improving and actually yes they are. It’s not jumping them up onto a functional scale yet but there is a change in what they can do.**

FP2: It’s nice to have that feedback to take back to the MDT as well having just come out of MDT and someone is still on a 2 on their Barthel after three weeks but actually if we had something a bit more specific at the impairment level then we could have been a bit more optimistic about their progress rather than a consultant just sitting there saying well they’re not getting any better are they.

**I: Especially when are forced to make quite early decisions aren’t we and therefore actually having something quite specific is maybe more valuable.**

FP3: We’ve found that with [name] at the minute. He’s sitting on the fence with which way he’s going to go but with having those scores it’s really nice that you can really see in impairment level and he’s not being rushed out either because we’ve got those really small goals. Everybody is saying well we’ve got more time, we’ve got more time and we don’t just feel like we’re having to make drastic decisions that he’s going to end up in a placement. I think having those much smaller impairment level goals has made life for us slightly easier with keeping them a bit longer, which has been really nice.

**I: That’s interesting. We digress. So I think we’re coming to the end, it’s been really useful to hear your thoughts. Just whether there is anything else that we haven’t discussed today that you came here wanting to share or particularly anything that’s been a challenge because you’ve been quite positive. If I can tease anything out that’s been particularly challenging around this.**

FP1: I think the challenge has been the recruitment and I don’t know how much of that has been us being cautious versus we’ve been stopping and starting with the pandemic and actually every time we started it’s felt like we’ve gone into about a three week lull of absolutely nobody coming in with appropriate strokes. FP2 you’ve been really and actually the two of you have also just been really, oh any candidates at all. You’ve been really great at looking out for people.

**I: I think that is the reality. You look on paper and you think we should be able to recruit lots of people who meet that criteria and then the reality is for many different reasons you can’t. So it’s been the same in every site.**

FP1: I know initially you were saying that actually the control centres were recruiting more people than the intervention.

**I: They did. So two of the control centres in particular recruited their ten target. Not quickly but fairly quickly. They achieved one of them before the pandemic started and the other one fairly quickly. Whether there is something in that, whether it felt easy for them because they knew then they didn’t need to do anything different and actually whether there was a little bit in the intervention centres of trying to find the right patient or a good patient or just thinking oh we’re going to have to think a bit harder about this. So subconsciously whether there was something going on or whether that was just pot luck I don’t know. One of those was where I work so they had the pressure of me breathing down their neck. Also I think there’s a big difference in how much research teams are involved in the recruitment. So both control centres that recruited quite quickly the research nurses entirely do all of the recruitment. They screen every patient they recruit and then they just come to me and say we’ve got this person we’ve just recruited someone for IMPS off you go and taking that off the therapists has got to make a big difference I would have thought.**

FP1: Although interestingly I would say because we lost [name] quite recently and actually then we didn’t have somebody who was doing it. I would say that meant for me as the PI here I had almost handed a lot of responsibility to her and didn’t have it quite so high on my agenda and when she was gone it was then sort of, oh my word it’s down to me, I’d better do something here. I then got the two at the same time. I was anxious about having two IMPS people at the same time but actually.

FP3: I think it’s actually been easier.

FP1: It’s better isn’t it.

FP3: Because you are doing it more regularly I think. But we’ve also had quite a high turnover of quite mobile people as well haven’t we. They’re coming through a bit more now the more impaired people but we had quite a high turnover and people leaving a lot quicker I think.

**I: So actually the study was inpatient into ESD and some of those patients actually could have been picked up and carried on and that would have helped with recruitment as well.**

FP2: Or I think definitely when the study first started we had quite a few men didn’t we who were we were at a point with ESD where they were taking some of our patients on Returns or they were taking them just as practice step transfer whereas probably now they’d be going to rehab but at that point they were taking all those patients who might have been able to be with IMPS.

FP6: That lady I sent down, well not me personally but came down to you yesterday probably would have been.

[FP: *The big ICH*

FP5: I think although I’ve not necessarily seen many of the IMPS patients for treatment other than say this week I think what would be helpful in the training initially was to almost do a couple of case studies of this is how someone is presenting and then a bit of maybe like a group brainstorm of what would be our ideas and oh is that part implicit and having a bit of a, I think almost initiating our thoughts about treatment ideas from the beginning and then we can almost see those patients and recognise patients to recruit faster and then have more ideas further down the line of how we adapt and who has what ideas within the team as well.

**I: We did give you very minimal training like an hour really of training and off you go, which was a bit of the test really to see what would be needed and also the other thing with the study was that what we’d hoped is as we recruit people over time we could see when we analyse the videos how you putting it into practice might change over time so actually it might take seeing X number of patients before you are really delivering it at 80% of the time or something like that. But there’s not been enough probably to look at that in the data I don’t think. I think naturally the more you do something the easier it becomes. Lovely.**

FP1: I’ve really enjoyed it as a study, it’s felt really meaningful to us as a team and whilst there have been big gaps in it actually.

**I: I appreciate you persevering.**

FP1: I feel sorry that it’s coming to an end. It’s been a good study to do.

**I: I’m grateful for your input. Just a final question whether you think your views of what you’ve been doing you might not feel you’ve done enough but have changed over time or have changed as we’ve discussed it this afternoon. Do you think what you thought at the beginning has changed at all to now or has it been as expected?**

FP3: I think it still takes me a lot more thought but I’ve really enjoyed it and I think keep trying to use it but it does take that planning and I’m always trying to think what am I saying, should I be saying that, have I given too much. But I think that will just come with practice as well won’t it just to keep doing it over time and doing it as well with people that you aren’t being recorded with and sometimes you are a bit more relaxed then aren’t you when you are not having to think about it quite so hard.

FP5: I think that the giving verbal feedback has become a lot easier so asking somebody how do you think that went is feeling more natural than it maybe would have at the beginning like even with any of the non-IMPS patients as well but I think it’s the verbal cueing during a session and not correcting movement that I still think probably requires a fair bit of thought.

**I: Thank you. I will turn these off. No one is going to say any little pearl of wisdom the minute I turn these off because that’s what normally happens someone saves it up and I’m like oh my God write that down.**

**FOCUS GROUP 6**

**I: If it’s alright I know you all know each other and I know you know, [name] doesn’t and she’s doing the notes and it’s just really helpful if you don’t mind just introducing yourself and just say which bit of the service you work in and your profession and that’s also so the transcriber can identify your voices on the audio. So whoever hasn’t got a mouthful of food.**

MP1: I’m [name] and I am a clinical specialist OT for stroke rehab.

**I: Lovely, thanks MP1.**

FP1: I’m [name]. I am a physio in acute neuro but at the time I was working on the stroke unit.

**I: Thanks.**

FP2: I’m [name]. At the time I was a clinical specialist on stroke and was the principal investigator.

**I: Yes you were.**

FP2: That’s the right title isn’t it.

MP2: I’m [name] and I was the physiotherapy team lead on the stroke and rehab at the time.

**I: Has everybody changed job?**

MP2: It’s the titles that have changed.

**I: OK. Obviously you had some kind of involvement in this study, you all came to the training way back when so you’ve had some knowledge of it. Just to understand who is in the room who saw patients who were enrolled in the study? Did you all? OK so everybody did see some patients and deliver some intervention. That’s perfect. So just thinking back to the beginning and when you first, if you can remember, when you first heard about the trial and this was going to be a site for the trial can you recall what you thought about the study and about what it was looking at - can you recall any of those initial?**

MP1: I think at the time we were quite excited to be participating in some research within the team. I think it’s something we were looking forward to doing actually, we’d not done it before MP2 and I and we’ve been working together for a couple of years in the team and we wanted to start trying to become not a centre of excellence but certainly a centre for doing things rather than just stagnating.

**I: So taking part in research broadly was.**

MP2: Yes I definitely think I agree. That appetite was definitely there. I think we were mindful at first of making sure we found out what it entailed because of the limited time we’ve got and space and I think we had good chats about things like videoing patients how that is really tricky on the ward environment. But equally when FP1 became prime principal or whatever it is, queen of research, there was how much time would that need to be set aside to be able to do that because obviously those conversations we then have to have as to we say it’s a fun thing but then also how do we protect that time. So yes a definite hunger to do it and then can we protect it when we actually are involved. The nature of the study itself made a lot of sense. It’s not completely new to us because some of the elements is something that we’ve done already but looking at it more closely it’s very valuable.

FP2: It was a real conversation generator wasn’t it in how we were going to deliver the different intervention and make sure that we were trying to adhere to the structure of delivering therapy in a different way.

MP2: Well acute stroke was a big thing.

FP2: Yes we tended to recruit them from there but yes I remember lots of people talking and beginning to share lots of ideas but initially I guess there was a little bit of hesitancy around how we were going to manage doing it. But everyone was on board.

FP1: I think at the time thinking back I think I was split between acute stroke and stroke rehab so I guess I was in a good position to be thinking about the recruitment because there was a maximum number of days post stroke was there?

**I: Yes, two weeks.**

FP1: So we had to thinking about them in the early stage because they may have gone over the time frame. So looking back I think and I think nobody else on acute stroke, Cath may have had, I think Cath had been to the training but she wasn’t delivering.

**I: Yes she did come to the training.**

FP1: The therapy being on F4. Yes I remember the recruitment part of it and with each of our new patients thinking will they be appropriate for the trial.

**I: With regards to what the study was looking at the implicit learning approach was that a new concept or how did you feel about that when you heard about that?**

FP1: I think I felt it wasn’t how I usually delivered the therapy so it definitely took me, and I think then as we started to try and do it I realised how much I explained things or how to do things to patients. So it definitely took.

MP1: I still do that now. I talk too much even now.

FP1: It took some time to try to get into that mindset I suppose.

FP2: I think when we understood it I think everybody thought that we sort of did elements of it already but actually when we got down to delivering it and trying to do it as true to what we were trying to achieve in the research then it was harder to plan wasn’t it and I think that it would have been nicer in a way if we’d had a few more patients that we could have we were just getting into a bit of a swing of it we were getting into working out what worked really well but.

MP1: It’s exactly as you say I think we’ve all built up a repertoire over the years of how you approach a therapy intervention and then suddenly you have to change, you have to gain new skills for your repertoire and that takes time to bed in doesn’t it and that’s exactly right we were just getting into the zone and just trying to, picking up techniques and thinking about things in different ways and then we had to stop, which was a bit of a shame.

MP2: It’s like you said about having elements of it with the study it had a pure, you were tripping over yourself going on hang on that’s not implicit learning and having to rethink it and plan it more. I definitely went into the study like FP2 said thinking OK with some of our heavily aphasic patients people with receptive difficulties I do an element of this already but like MP1 says when you are actually doing the study you realise how much verbal explanation you give through habit isn’t it. It’s like when you’ve got a very poorly unconscious patient we’re used to talking to those patients and we encourage that and so we just do it naturally so stopping myself talking was a challenge.

MP1: Directing isn’t it, it’s not so much talking because you can talk about other things but directing what you are trying to get people to do is so habitual, is so built into what we do.

**I: So you’ve mentioned you felt there were elements that you probably do or did already. Can you describe what those elements were, which bits of it do you think are the ones that.**

MP2: Like having an obvious goal and target that was almost instinctive for the patient to get and we do that with patients who wouldn’t necessarily understand that we’re telling them to reach out that there is support. We’re saying OK let’s have a drink now or something and can you get your drink and they would actively do what we were wanting them to do through that purposeful [*8 mins 35 secs*]. So we would do that naturally. The basis of our rehab is functional anyway but like I said often we would have already said it as well prior to that so I guess that that’s element it’s something really obvious. The one thing I would say as well is if we had more patients and time I can imagine us setting up the area like the gym space which was where we did our implicit learning stuff because thinking about what equipment you are going to take in and where you are going to put it was really quite a challenge as well whereas you could have that set up and have buzzers on the wall and obvious feedback that you’ve reached a target would be set up ready for you to go, which we didn’t necessarily have.

**I: So needed a bit more planning.**

MP2: Probably.

FP2: I’m trying to think of an example where I felt like I would do it anyway but I often find myself thinking particularly if I’ve come into help in a session that patients are just overloaded with instruction. So I felt like I knew and had some kind of approach towards this end of the spectrum if you like because I’ll always try to dial that right back and allow the patient to learn from their own experience without us telling them exactly where to put their feet and how to lean forwards to stand up for instance with sit to stand and would often go to a chair like I’m sitting in, a free standing chair, so if actually they get, as long as it’s safe obviously, if they go from sit to stand and the chair moves they get that feedback from something else other than us telling them all the time that they need to learn forwards, they need to do this, that and the other. So you kind of look for environmental for the patients to learn for themselves, which was probably where I in my head I thought we were, yes we do this already, but we only do it for a little bit and only to a certain degree. I think it’s always diluted by our chatter and our instructions.

MP1: It is, it’s like we can’t shut up. I genuinely think it is we are hardwired to narrate what it is we’re doing with patients all the time. I don’t know if it’s for our benefit or theirs to be honest.

**I: That was the next question why do you think that is?**

MP1: I don’t know.

FP2: Silence is awkward.

MP1: Yes.

FP1: I think it might make them feel more at ease but that might not be the case.

MP1: I’d agree as well.

**I: So it’s that human nature isn’t it to communicate.**

MP1: It is reassuring sometimes when you are explaining to patients what they might feel in the moment. When you do this this might happen. It gives them some trust in you that you know what you are doing because you predicted what might happen if they do that. So I don’t know. It is difficult. But as MP2 said earlier on working with PDOC patients where we sit there and chat away around what we’re doing with people it’s what *we’ve always been brought up to do*.

MP2: I think also *it feels like engagement doesn’t it* because you are trying to engage in a conversation and although we actively do that through speech most of the time and it’s that dignified, OK you are the person and we’ll talk you through this sort of stuff. It feels like dignity whereas when you have the awkward silences it can sometimes feel like teach and learn and you want to try and avoid that. I think part of it is that. FP2’s point about the sit and stand was a good point, one thing that we do which I call controlled fall but it’s not controlled falling but if you’ve got someone who is actively pushing or overactive you’ll let them feel that because they’re learning more from the feeling of I’ve gone off balance than us telling them that. So that would be an example of where we’ve done it. But then we assure them afterwards and then talk them through what just happened so cutting that bit out was more of a challenge. I think it as well massively depends on the patient you’ve got in front of you. There are some people who want to know everything and there are some people that you hold back stuff because they need to learn for themselves. I can imagine long term which I’m sure is one of your questions is you pick who does this fit for and we’d get better and more skilled at deciding that.

**I: Yes you are right that is one of my questions.**

MP2: I told you it’s hard for me to shut up.

**I: In a few different ways so going back to just the person’s preferences is what you were alluding to there so how would you know what an individual’s preferences are for the way they learn. Do you have a way of.**

FP1: I had a patient that even during the therapy and in the implicit way she was just asking for my reassurance and my feedback. So she was almost the other end of the spectrum, she would have been probably happier with me telling her exactly what I wanted to do, how to do it but that was only by trialling it with her and then seeing how she responded I suppose. We wouldn’t have known that without, once you try that method.

MP2: She wanted to power on didn’t she that’s the thing. You use that as motivation, she was looking for your not approval but she would say I’ve done well but wanted you to also say you’ve achieved that thing, you’ve achieved that thing. She just thrived on that.

**I: So do you feel what she was wanting was something more explicit or was she asking.**

FP1: I wonder how to describe it. She almost wanted if I gave her the instruction she almost wanted more detailed instruction at times and then at times she wanted more feedback to know she’d done well rather than just feedback that the buzzer gave or that you did it ten times. Would you say that?

MP2: Yes, I remember asking questions about her knee and stuff like that and we were trying to avoid that.

FP1: Yes where should this be, where should my knee be.

**I: She was directly asking you?**

FP1: Yes.

MP2: And like I had a chap who just loved it really. He definitely wanted explicit analysis afterwards so he responded really well to the implicit like the buzzer we used a lot of and using the skateboard to get to the point and things like, he enjoyed that. Then the second part of the session he wanted to talk through it all and what did I see, and what did I think in all those respects and have that analysis afterwards. So it wasn’t that he wanted to change the session itself but he did want a moment to be able to talk in depth about it. That was him personally. I think it sometimes depends on the patient’s view of us, if they see that I’ve got this expert for instance in front of me I want to grill into that find out what they mean and I think that lady definitely had that perception rightly so of wanting to chat to you and [name] about it all and why they did those sort of things. It’s almost that you could separate the session and the active stuff you are doing and then an analysis afterwards.

MP1: The practical stuff, the learning stuff, implicitly but then some people want that explicit feedback about and quite understandably they want A) to know that we know what we’re talking about and B) to know that they’re making progress. So do we think they’re making progress rather than that implicit oh I must be making progress because I’ve done this.

**I: So that’s really led by the patient in those examples. They were able to say specifically what they wanted from you. Can you remember did they hold on to that information do you think, that analysis bit, and remember it the next time or the next time you saw them were they using that?**

FP1: I think so but I think my particular patient expected her to make progress every single time like noticeable changes every single time. She was quite hard on herself so she just wanted the next stage better each time.

MP2: My chap subscribed to what we were trying to do from a research point of view so he understood that actually we weren’t going to go into loads of depth analysis. He wasn’t continually asking for that but he was quite happy in that situation so he knew about the trial. So although he appeared to say that he was happy he would carry over more target based stuff, I achieved this. And that was nice actually so he could say last time I got this far or I hit it that many times. He definitely carried that over particularly for the skateboarding we did. But yes so it was a bit more challenging because he did subscribe to the idea of not going into too much detail about it, I don’t think we can ask anything other than that.

**I: From the patients you saw do you feel there were any other groups of patients it worked better for or not so well for or that you might anticipate it would work better or not so well for?**

MP1: Outside the study?

**I: Yes the ones you saw or actually just more broadly.**

MP1: I’ve seen two that I can remember but probably more non-English speakers, completely non-English speakers who have really done well. There was a Romanian guy that I worked with and another but I can’t remember where the other chap was from. But that was all implicit because it didn’t matter what you were saying to them they were completely unable to understand. And they made, certainly the two I’m thinking of, made actually really good progress compared to where you thought they might get to when they came to us with the level of their impairment when they started with us. And it’s using that, I think that taking the skills that we got or some of the experiences we got from doing this study you could take that forwards working with those guys, it worked really well.

**I: That’s a good example, you are forced to be a bit implicit aren’t you if someone doesn’t understand.**

MP1: Exactly, yes, yes, we had absolutely no choice other than to be implicit so we developed some of those strategies already that we could use which was quite handy.

**I: Maybe this could help my research in the future, recruit people who don’t speak English.**

MP2: There was also another lady who overanalysed where we proactively go very implicit. I’m thinking of a lady we had in a cubicle that ended up on that ward recently would overanalyse it, actually quite anxious about that and talk through too much and overthink things and so the process of being able to stand up to her became overwhelming and mostly led by what she wants to know and we really went back to stand up and actually some distraction as well but actually we are just going to talk about stand up. We’ll deal with things and were subtle, if we want to get the knee straight etc we might go and facilitate that. But we really whittled down how much we told her or how much biomechanics we talked about. It was just about OK let’s stand up today. The other thing I was saying gait re-education as well there’s a real place for implicit because we can sometimes, people have got to think about where they are placing their foot, how their knee is going to be and you forget they don’t have that experience and sometimes it’s better just to go, let’s walk across the room. We have the skills to keep people safe and therefore we can adapt where our handling is to help them. So I definitely think gait re-education would be somewhere I think I use it more and early standing where they’ve got too much to think about, they’ve got a lot going on, they’ve just had a massive stroke and they are going to stand for the first time. You want them to just think I’m going to stand up. But I think part of that is tapping into the automatic motions that we go through and walking is functional because most of us do it and standing up is functional so actually adding a new element of information about that isn’t necessary in some respects. So perhaps those two populations of people. Then aphasic patients as well where it’s not that we don’t use explicit learning we’re very mindful they’re not going to be able to completely process all the information we are giving them. So perhaps that could be somewhere we do a lot more implicit than people who can communicate more effectively.

**I: I think that’s probably naturally intuitively would have done that anyway.**

FP1: A little bit like the non-English speakers in a way because it’s just whether it’s to do with what type of language they are, again it’s understanding isn’t it.

FP2: Where we naturally like to chat to people and we have to really curb our tongue when we were trying to do the study there’s no point in chatting away with someone who is aphasic or can’t understand us because of language so it’s given us more of a tool, it’s made us think a little bit more diversely I think about what you can do with that group.

MP1: We’ve got a gentleman in my group where he’s exactly that like non, barely speaks a word of English. He was getting on really well, he’s not my patient he’s MP2’s patient but I’ve done some work with him and he’s really benefiting from just getting into a kitchen and cracking on. There’s your stuff let’s get going on it and being very goals and task led, goals led and less facilitating and stuff like that. He’s doing really well. No thanks to me it’s all MP2 and [name].

MP2: The tricky thing in that respect and he would now totally agree he responds really well to implicit stuff but it’s motivational so he’s been here a while and the functional stuff is quite obvious but when there’s maybe more of a choice to hold stuff back. So this person wasn’t in the trial but heavily ataxic very cerebellar and we wanted to do some static closed chain stuff, get that better before going into loads of walking and he was quite impulsive and most of that is implicit functional stuff, played some games and targets and stuff like that and just some general standing. But it’s more obvious through conversations through an interpreter and directly with him his motivation and mood was an issue kind of then going back and explaining why we are doing it helped because he definitely trusts me with what we were doing but when you’ve done it for four weeks and you feel like you are doing the same thing it does then need a conversation I think sometimes to say this is why we’ve done it. Hindsight now he’s happy as Larry because we’re now at a point where he’s doing walking and a lot of it again is implicit again because it’s very functional. But that time he wanted to walk, probably didn’t have the ability to but having that conversation helped so trust us, trust us where we’re going. I think it has a place and I wouldn’t necessarily say that patient is always going to have implicit learning styles it’s going to be I choose to do implicit in some sessions and that sometimes I’ll have to have a conversation about where we’re going and what we’ve done. The only group that’s perhaps less common is where you have people who perhaps analyse and don’t necessarily see our expertise straight on so I’m thinking perhaps more the acute stroke side where we have to do a bit more explanation to get them on side so we go almost we know what we’re talking about sort of thing. There are not very many people who do it and it tends to be more relatives of the patients to be honest but that would be another place where we, because it’s getting them part of what’s going on in our heads so they can understand that we know what we’re talking about sometimes.

**I: So it sounds like you and you’ve described that a few times like a bit of a hybrid that you might use some implicit techniques when you are actually doing something with a patient but actually there’s a need for them to understand what we’re doing in therapy and why some of that conversation might sit a bit more explicitly.**

MP2: It’s a spectrum isn’t it.

**I: Yes it absolutely is.**

MP2: You might say to them, you might talk to them about hyper extension of the knee because that particular person is going to be able to engage with that and understand it and other people you just want to say look how much stronger your leg is now and look how much stronger your, particularly inattentive patients where you want to draw their attention to that side. Yes we do it sensory but we also might want to say look how great this leg is doing and that wouldn’t be truly implicit because we’re just thinking about standing but sometimes their instincts particularly our pusher overactive patient who feel like midline is there, actually educating them about why it feels different from what the reality is can be really useful. A lot of those patients we just do through weight bearing and feeling your balance but there is the education is really important.

MP1: Agree. I think you can treat that implicitly and bring across the buzzer it’s not that big task but it’s only fair to let people know why they are feeling like that because it must be pretty terrifying to feel you are constantly being told you are out of midline and you feel like you are falling to one side and things like that. It’s only fair to reassure people I think but you treat it in a different way. I think that’s two different things though.

**I: We could probably debate whether that’s explicit, describing to someone their impairments and why they’re having the struggles that they’re having. Yes I suppose that’s open to debate as to whether that is actually explicit or whether that’s just an explanation of what’s happened to you, I don’t know. It’s interesting isn’t it. And did you have anyone in the study who had cognitive impairments? I can’t remember.**

MP1: I don’t think we did.

FP1: Was that a.

FP2: They would have needed someone to consent on their behalf wouldn’t they.

FP1: I don’t believe we did.

MP1: It wasn’t in the inclusion.

**I: In the very original inclusion they had to be able to consent but we changed that so it could be a consultee consent.**

FP2: No I don’t think we had.

MP1: I think we probably would have gone down that path if the study had gone on longer. But we wanted to get a few easy wins under our belt.

MP2: My chap had subtle memory difficulties but he was carrying over between sessions so there was nothing profound in the two that I saw actually.

FP2: We didn’t have anybody that couldn’t consent by themselves.

**I: From your general experience of working with people with cognitive impairment what sort of approach would you think you might use or works or what doesn’t work?**

MP1: Again I think you go through a range don’t you. I think you might be explicit but if that doesn’t work you might be implicit or you might balance those two things to get the most out that person during that session. I don’t know about the others but I don’t think we can purely go down the route of saying that’s it I’m only ever going to work implicitly from now on in terms of the therapy I provide. It’s always going to have to be a mixture but what this has demonstrated it’s highlighted to us that there is different ways of doing things and people do get better, you don’t need to talk all the time. With people with cognitive impairments they don’t want that cognitive overload, they don’t want people shouting instructions at them from all sides at the same time. I think it can be better to have more implicit therapy.

FP2: Sometimes they’re quite abstract tasks aren’t they so from a cognitive point of view sometimes they’re completely, they’re almost completely meaningless and you might confuse somebody even more by buzzing the shoulder.

MP1: As an OT I try to put that into meaningful occupations.

FP2: But if it can be in a functional way I can really see its value but some of them with our cognitively intact people you can explain the task that they’re going to do and that’s their implicit learning if they’ve managed to move the skateboard to the buzzer or whichever it was but for somebody who is cognitively impaired that can be really too out there and a bit bizarre. I think you’ve got to find where you can get the patient engaged.

FP1: I think that’s probably our experience of how we are like what’s in our toolkit of implicit tasks as well I think a little bit. If we’d have done more of it we could have I think I could have combined the function a bit better with I guess I didn’t feel like I needed to because they were responding to the tasks the cognition wasn’t because the cognition wasn’t there.

MP2: There’s a lot to say about that like familiarity as well. It’s not just that we go for functional tasks it’s is it familiar tasks to that person. We don’t get our bald patients to comb their hair.

MP1: You say that.

**I: I bet there’s a physio out there that does.** **Not an OT obviously but I bet there’s a physio that does.**

MP2: I think perhaps we’re so where MP1 and I are on the same unit we probably do more home visits than perhaps have done in the past but again that’s because of familiarity and it’s perhaps easier to do implicit stuff when they have that not control but awareness of the tasks.

MP1: It’s another thing that they don’t have to worry about. If you are doing something in an environment that’s familiar to them and doing a task that’s familiar to them then you actually get them to concentrate on what it is you are doing rather than all the outside influences that might be distracting them or they have to overcome in order to manage that task. We do do more home visits than we used to actually. I don’t think that’s drawn by what we’ve done in this research but it’s certainly been coincidental but we’ve just had a run of patients where our belief as therapists is that they would probably perform better in their own in a more familiar environment.

MP2: And it’s knowing what the output they’re expecting as well, what’s the reward in terms of the structure [*30 mins 42 secs*]and by them being able to achieve something. We had a blind patient who was newly blind because of bilateral strokes who we did a home visit for and by being at home a lot of that is going to be implicit, they know it’s their tap and what do they achieve when they’ve turned it off and turned it on etc. Perhaps it’s more than just the implicit/explicit learning is it, there’s more to it than that.

**I: Yes absolutely.**

MP2: It’s the familiarity.

**I: But a general feeling that building in things that are more functional and meaningful to the patient lends itself to being more implicit. Is that what?**

MP1: Yes. I’m an OT of course I would say that. It’s the whole science of my therapy is based on.

MP2: When you do training and stuff sometimes it reminds people about that. You know when we do goal setting and it’s like actually if that’s the way they’ve walked for 25 years then that’s what we should be aiming to get back towards rather than doing the perfect model so to speak or go exactly what the textbook says. What is your output, what are you expecting to come from it. If establishing that helps for them doing implicit learning then.

**I: Thinking back so when we did the training probably remember the skateboards, there were principles for implicit learning which were about reducing the amount of communication and also trying to have the focus extend onto the body were the two main principles and we gave you some quite discrete exercises as examples of those. How easy did you or not did you find it to take those principles and then put them into different scenarios and come up with different, you mentioned back to a box of ideas and if you had more time you’d have put together more of a toolbox but was it hard to take the principles and put them into different tasks for different patients?**

FP1: I found it difficult initially and I think my patient was on the lower level physically end of the spectrum so I was trying to make sure that I wasn’t compromising on movement quality. So it took me a while to figure out what exercises would, not exercises, what tasks would get the right quality of movement and be implicit. So yes I think it took me quite a while to get my head around. I think you had to have a go at one exercise and for this lady in particular she didn’t do very well with not achieving something so you had to be really careful that you weren’t setting an unachievable target because she was on the lower end of the - was it just they had to have independent sitting balance and they were starting to stand.

**I: Five seconds of sitting balance minimum. She might well have had more than that but that was the criteria.**

FP1: She was on the lower end. I think I found it easier looking for tasks when they were already standing, they could stand and reach and do a little bit more as well as the lower because I remember trying to do some things in crook lying with the buzzers and trying to, do you remember. I think was doing it with you or with [name]

MP2: Yes we had like a wobble cushion on their knee and so it was obvious that the wobble cushion would fall off or not fall off and we wouldn’t have to talk too much about that it was just that was the obvious feedback and she got that wrong and had to tell you keep your knee here and try to keep your hips stable when you’ve got a cushion there that falls off or doesn’t fall off. I can’t remember who counted, I can’t remember that bit.

FP1: Yes we did a count. We did like a

MP2: And we had to put how many seconds can you achieve it. Because part of the training was to refine it wasn’t it to something that was achievable and then open that up.

FP1: But for this lady it had to keep that part of refinement(?) quite short because I didn’t want her to not be achieving the task because that would have not gone down very well.

MP1: Grade it back to something that she could do.

FP1: Yes she was very hard on herself anyway so but it was almost like you had to explore a little bit before you got an exercise that worked well.

**I: Do you think you did find things that worked or do you think in that scenario it was too difficult to do that? You did.**

FP1: No I did manage to get things but it took a little bit of time and I think she was my main patient that I was in the study with I think I joined other people for some of the higher level ones but yes I wish I’d been able to do it for a bit longer because you would have built up a bit more ideas.

MP2: Perhaps what would be nice if having more patients for a longer time is linking it into the functional stuff because we know that we need to be functional but I think in my sessions I used a lot of the buzzers and I used a lot of tape where we achieved??[*35 mins 40 secs*] but actually how much functional stuff. Like I did functional stuff in standing but it wasn’t, it didn’t come second nature to put the two together not because they don’t fit together because you’ve got to make sure that you’re doing.

FP1: You liked the noises didn’t you with the buzzers.

MP2: Yes

FP1: They were really irritating but I liked them.

**I: Really irritating when you watch the video back.**

FP1: I like that we had a variety of different sounds.

MP2: We had like, I’m sure it was oh that chap, we might have done some functional stuff on the table but some of the feedback I had was I don’t want his trunk to go back and forward like this so that was holding a buzzer. If you heard the buzzer you knew you were too far forward and back over and over again. But then like I said that wasn’t necessarily the directly functional bit that was just his feedback about where he placed himself so yes it would have been nice to be able to fit the two a bit closer. I didn’t do any wash and dress sessions really I don’t think with my chap and so I didn’t have that opportunity to say how would that be implicit and how can I prevent that from being explicit. That would have been really nice to have done. Again we’d be able to identify groups of people perhaps that would have worked for and didn’t. More time and opportunity like everything

FP2: I think our environment is quite limiting as well to come up with ideas. You saw the ward environment and quite often we’re forced to deliver treatment sessions in the bedside and it certainly made the videoing side of it hard. But yes I seem to remember, I’m just trying to remember whether I was trying to help you with the lady in lying. But I remember trying to set up something in crook lying and we just couldn’t quite get it all set up in the hospital bed. That was quite limiting for us.

FP1: I know I definitely did some on the clip because we used the wall and things as well I think but I think we did try and do some stuff at the bedside as well.

FP2: Active extension into trying to set something up so she was pressing probably the buzzer.

[*38 mins – all at once*].

FP2: I don’t think we could properly get that to work. I think that was quite a limiting factor for her. The short time that we had on it really.

**I: You’re remembering a lot.**

FP2: Oh really?

**I: Yes.**

FP2: We’ve got 3 or 4 people who were helping treat some of the patients who’ve left so it’s a real shame [name] and [name] and there was someone else as well.

MP1: [name] maybe

FP2: Who?

MP1: [name].

MP2: [name] was definitely in it for a bit.

FP1: [name] and [name] but I don’t know if there was anyone else.

MP2: Because [name] had the chap who liked golf and she had stuff like having the tape down the middle of the room and he would just step each side of the tape. I remember that’s what she was doing a lot of with her chap but she’s not here so. I guess it’s because we’ve had COVID so we can kind of remember back to that time.

**I: So patients who are early post stroke and you might not have had in the trial I just wondered if you had any thoughts on how easy or not it might be to apply implicit learning principles if you are working on early sitting balance and things. And the second part of the question is where do you think handling and facilitation sits with – and there’s not a right or wrong answer to that, I don’t have an answer in my head with the spectrum of implicit and explicit.**

FP1: I think sitting balance could be done implicitly rather than move your shoulders, move your hips here it’s moving it to a target. I think sitting balance would work.

MP2: If you break that down so forget on our air mattress for a second because they’re the bug bear of my life so I definitely think implicit would work with sit balance because you give them the perception of falling safely so that they feel that and then how do they compensate. That’s when your handling might come in, are they just purely side flexing and then you can handle them so that they’re weight bearing better on their pelvis you are almost doing implicit plus the facilitation. Yes I would definitely agree with that, that’s the early on time when implicit would work really, really well. And also because the amount of information we have going in those first let’s say the first session is 48 hours later they can’t take on loads. You do a short introduction because you don’t want that to be just your session so yes I would say you do a lot of stuff implicitly. Roll to me and they can do how they want to. Maybe a part of it as well is because you are more finely assessing at that point and see what they do and do we have to change it, do we need to do this, that and the other and can they just roll over. So yes I agree.

FP2: There are some real subtleties as well around what you are trying to achieve so if you are trying to facilitate by getting into pelvic tilt or into bridging and that early activation around the pelvis so many patients don’t really know what it is that you are after and it’s quite hard to I’m being a bit rude but particularly blokes they can’t find their bottoms but if they get it right and to have something that reinforces what that movement is that you achieved but they probably wouldn’t get there without the facilitation so maybe there’s a little bit of a balance. But I think somebody to reinforce that movement, whatever it may be, just picturing that bridging or crook lying you are just trying to get them to find their pelvis a little bit so they can then move appropriately.

FP1: I guess it’s like the sensor mat would lift

FP2: Reverse falls alarm basically that sort of thing.

FP1: When you lift up and it. I guess you create the noise don’t you if you are lifting.

FP2: Yes something maybe more pleasant.

MP1: Well yes I was thinking when you were talking about the buzzers actually because it activates and maybe it might be more motivational to turn them off rather than turn them on so just leave them ringing and work out a way of turning that buzzer off. That’s certainly going to lead to better outcomes.

MP2: That’s called torture, that’s different. With the implicit side of things is we’re thinking about the kit that we had and obviously you don’t have to have kit to implicit so no one knows, if we tell someone to pelvic tilt like what are you going on about and a lot of people where’s your pelvis. But with that sort of thing it’s like well I need to get my hand under here in the small of their back and then you see can they do it for themselves or we need to get your underwear on and do they implicitly then bridge for that because yes pelvic tilt is a really good example of just trying, even somebody who has done hours and hours of physiotherapy or OT to know what a pelvic tilt and how to do that can be really challenging and the general population some people just aren’t very good at it are they. I think perhaps because it was a study and because we were really thinking hard to make sure we’re being implicit we’ve perhaps used the kit more because of that. It's like OK if in my head I’m using a buzzer that reminds me I’ve got to do it in a certain way whereas the reality of it is often we don’t use that kit and we still do implicit stuff. Acute was really actually quite an obvious place that we do quite a lot of it.

**I: And video camera and study makes it all a bit of a false scenario doesn’t it.**

FP1: I was really self-conscious.

MP1: [*43 mins 19 secs*].

MP2: I said what.

**I: So you were overthinking, you were trying not to get the patients to overthink it and you were doing it instead.**

FP1: The first time I was behind the camera it was just well on camera was trying to explore how to get these exercises right and I was like oh no I look really stupid. I’m doing it wrong.

MP2: That would be good to know is if we did it like a [*43 mins 41 secs*].

FP2: In your write-up you put the team at [name of hospital].

MP1: Great focus but terrible therapists.

FP2: They did try. Wooden spoon award.

**I: You are remembering loads given it’s such a long time ago. Do you think there was anything about the approach that wasn’t clear to you. Was there anything that you recall at the beginning that you thought I don’t really know what we’re being asked to do or how I apply it? Did you feel you knew what you needed to do it was just you had to think about how.**

FP2: Yes I think so. We had some head scratching moments didn’t we but.

MP1: Yes vaguely and sorry I can’t really remember but it’s about, I think we went back to principles rather than being about specific things like you said earlier external to the body or internal to the body rather than external direction and using those principles. I’m the same as FP1 I think that first couple of times this is really alien and I’m not sure how this is going to work but it was

MP2: It’s like a language isn’t it that you learn. Because there are other bits that I remember thinking that the nature by which I’m communicating isn’t explicit but the choice that I have made to do that task is because it’s not functional is almost, it’s breaking it down so much that they work out for themselves what they were trying to do. When you are trying to crook lying lift your knee up that’s not particularly functional it feels like it would be more implicit just to go through the functional task and then we break it down but when we’re refiningit so heavily they knew they had to lift their knee a certain amount even if we weren’t telling them to do that to hit a buzzer because it’s abstract and precise it kind of felt like that was perhaps a little bit explicit anyway. Does that make sense? Although I wasn’t verbally telling them you are hip flexing in order to hit that the choice almost, I don’t know if that makes any sense.

**I: And actually in any scenario we don’t actually know where the patient’s focus of attention is unless we ask them so we set things up to try and bias it but unless you said to the patient what were you thinking about when you did that, were you just thinking about the buzzer or were you thinking about your hip and knee then yes we don’t know we just make an assumption.**

MP2: Yes so there were times I remember afterwards in the sessions saying oh I said this and we got hooked up more on one of the other ones with your lady where I think we had said the word knee or foot and that was it. Knee or foot.

MP1: Oh my God.

MP2: You [*46 mins 26 secs*].

**I: This is the thing in the research that we are saying in the training or trying to say, create your bias towards one or the other but then you go away as a clinician knowing you are in a research and you are being filmed and you think oh I’ve got to be 100% implicit whereas it was only ever bias it heavily. So it was not wrong to say the word and there might have been scenarios where actually there was no other way to describe this apart from to say put your foot there.**

MP1: Patient slowly toppling over.

**I: Oh my God how do I, yes.**

FP1: That knobbly joint between your hip and your foot.

**I: So I think that is part of the falseness of being in a research study as well. The other thing what we had initially hoped is that we would be able to see over time how your practice changed. So you would anticipate the first person you recruit might be quite different to number 10 in terms of how natural it feels but there wasn’t the opportunity to do that. I think we’re nearly there. The last few questions were just about and you’ve probably touched on it a little bit about how you would pick up whether or not the implicit learning approach was benefiting or not with an individual patient over the course of time. How do you think you would know if what you were doing was the right approach for that person.**

FP1: If they’re progressing then I guess it doesn’t matter what, in a way whether if the approach isn’t working if it’s working like you wouldn’t necessarily need to change it if they’re making gains and their feedback as well even if you don’t necessarily ask for it they might be telling you yes this feels this is working, I’m enjoying this or just how they feed that back.

MP1: I think that’s exactly how you if they progress to a point along the expected pathway with the approach you are using then whatever approach you are using seems to be the right one. If they’re not then it’s the same with anything with any therapeutic intervention if they’re not progressing along that pathway then you’re probably not using the right approach and you need to reconsider how you do it. I think that’s in all of us, that’s just a natural way we manage rehab and therapy isn’t it.

MP2: I totally, so from a positive point of view that’s definitely I agree are you achieving their functional goals, are they getting to where they want to get like you were saying then it’s like well something is going right. It’s like the neuro isn’t it if it works change it if it doesn’t work change it.

MP1: Yes.

MP2: As long as we’re getting to where we’re getting to. I guess the flip side of is it not working it would be whether it’s explicit or implicit is are we getting into such compensations that we think we’re hindering. So the classic would be where you are using a piece of equipment so if we’re using a Stedy which is often something that we use we’ll have done plenty of free standing etc. but then are they so heavily overactive that we are going to hinder them from doing stepping in the future and that might be where it’s time to say OK. And again it may be not so much the implicit and explicit but more the educational side have we educated them enough and taken things away if we need to. So perhaps it’s that balance because we do have to think about the long term as well and there are things that we not necessarily hold back but we do things in a certain structured order in some respects so that they might get somewhere in the longer term.

MP1: I just thought as you were saying that though it’s not just us is it. If we’re looking at the 24 hour rehab approach and we’re doing half an hour of implicit therapy a day and they get 23½ hours of being instructed on how to wash and dress and stand by other staff then are you going to keep the value of what you are doing. You need to be a whole team approach if you are going to do it.

**I: How easy do you think that would be?**

MP1: Broadly speaking pretty impossible. I think it’s a challenge for us to even get people to try and promote non-compensatory strategies and movement patterns let alone do that implicitly.

**I: Do you think nursing colleagues are generally explicit?**

MP1: I think they’re either neutral, I think they’re either explicit or neutral. They either do stuff to people which is neither here nor there or if they are then they’re telling people what to do. And patients look for it, the patients expect to be told what to do by healthcare professionals especially nurses role and therapists. But I think that leads to the institutionalisation we see with some people that we adopt a role, we go in, we expect to get told what to do and get up for our dinner and when to go for a poo, when to go for a walk and all the rest of it. That’s a whole team approach that needs to be relooked at I think.

FP2: That’s a different research project though.

MP1: That’s a completely different research project. I completely get that.

MP2: It’s also when it comes to this sort of thing it’s how much we can rely on the patient’s own systems to accurately do something. So it’s like perception of vertical if they don’t have that perception as accurate we would have to structure quite heavily sometimes how they go through their sessions. Whereas our nursing colleagues won’t necessarily be explicit in that respect because they might just say roll over and the more experienced ones will then identify that they’ve not actively tried to use their affected side for instance. It’s hard to say because wash and dress like you say it might be very passive or if they are asking them to do something you could argue there is a lot of implicity there because they are just saying roll over and the patient knows they’ve achieved it because they’ve rolled over. It’s tricky. The challenges of 24 hour rehab we all know and a lot of that is to do with staff turnover and time to teach people and things like that.

**I: Yes a whole different topic. I suppose just building on that so this last but one question I think if there was a future study and it did show that for a certain population of patients or a certain time implicit learning is more beneficial what do you think would need to happen to get that widely adopted in practice? You guys have done it because you are part of a focused research study but.**

MP2: I think maybe population it’s not [*53 mins 18 secs*] what’s going to happen but if it ended up that a particular population of patient it worked really well for it could be a bit like when we do errorless learning for apraxia but it’s like you know that let’s hypothetically say they are heavily receptively aphasic one of the methods we would use is implicit learning. I think that would be sort of(?) that’s what this looks like. That would help the adoption of it because I think part of the skill which I certainly struggle with is identifying how to do it and when to do it. I think that would help. We can’t make that happen but if it happened to be that the research study was does it work with receptively aphasic patients or non-receptively aphasic patients and it did work really well with receptively aphasic then it’s obvious we try this with this patient, it’s not prescriptive because we will change things but I think that would work.

FP1: Inhouse training on it I think because experience people that have had experience of using it like reading it back and doing joint sessions with other therapists that have never potentially never used that sort of method before.

FP2: As long as it doesn’t involve our videos at the stroke conference. There’s such little research that tells guys therapy to a specific method that will be not guarantee I don’t want to say guarantee but that evidence sways that they will do better if you use a particular method we lack that don’t we in this profession. So I think if the results were indicative then this is a way forwards for patients in general or certain groups then you’d be wanting to shout it from the rooftops and I think therapists would be really quick to adapt and adopt that method in the interests of trying to get the patients improving. I think you are then potentially then looking at the next step of getting people doing it in their own homes. We can use our community stroke rehab team and I think that’s perhaps where we struggled in a way to recruit patients putting COVID aside so many of our patients are going quickly with CSRT that I think we missed some people who we would have otherwise been using. So that would be a cohort of professionals that could do that training and thinking outside the box but it would be incredible if we had some evidence that showed.

MP1: I think by virtually going round giving 10 local stroke units to do this study I think the 10 local stroke units are at least aware of what implicit learning is even if they weren’t chosen to take part in the study. I think that’s how you need to do it, it needs to be spread through local units, local networks. We did a recent upper limb pathway [Wessex] upper limb pathway that was done through us and collaboration with the guys at [hospital] and down in [hospital]. That’s what you need to be looking at if it worked and the research and the study demonstrates there’s evidence to say this works in a particular client group then that’s where you need to be networking through them.

MP2: I think what helped in the training we had here is the examples that you gave as well. You could imagine if it’s successful if you wrote down some of the classic ADLs that we do and say OK what examples of a strip wash how would you make that implicit I think that would be really helpful as well because like we said before we are second guessing ourselves sometimes as part of the training to have that would be really useful. I would definitely say if that was available to people let’s say it was obvious it worked in this population and therefore this is an example in a strip wash how you would do that that would really help but you’d probably find.

MP1: Or a YouTube video with ten different clips.

**I: [*57 mins 19 secs*]Library (?) and progression.**

MP2: Because you’d start off by doing that wouldn’t you, you would start off with examples because you know that’s right and then you would develop out of that a way of working.

**I: Great. Then just finally, I keep saying it’s the last question and then I realise I’ve not turned the page over but we are nearly done. You have touched on this a little bit because you said right at the beginning you said when you started talking about the study it stimulated some debate so just throughout your involvement is it something that you’ve talked about a bit as a team, what are those kind of office conversations or corridor conversations what people thought or people said?**

MP1: I remember at the time in the office there was lots of talk about how it could be done, how to problem solve different.

FP2: What different techniques had been used and how well it had gone. Sharing ideas. Obviously those conversations came to quite an abrupt halt when everyone had to.

**I: Why was that.**

MP1: Discharge all our patients and split the team up. About then

MP2: We had actually decided to meet for half an hour each week to talk about it and that disappeared quickly. But the idea of that was to share ideas and because a lot of the conversations was how am I going to do this, or am I doing this right.

FP2: Yes I’d forgotten that.

**I: I’m getting the sense but correct me what kind of positive conversations about how we are going to do this and not like oh God.**

MP2: Yes lots of laughing at each other.

FP2: Yes it was very positive I would say. That was the vibe I got from everybody they were embracing it and scratching their heads a few times but actually.

FP1: Rising to the challenge.

FP2: Yes absolutely.

MP2: Yes it’s about our limitations and not being used to it rather than limitations of implicit learning. We were very keen to do it it’s a case of learning a new language of doing it and it worked well to have peers around and say oh I did this and it didn’t work. And also having a little bit of accountability like there were times we were saying oh no I think that wouldn’t be implicit and talking that through.

**I: Sense checking with each other.**

MP2: Yes exactly.

**I: This is absolutely the last question. Is there anything else that you want to say that we haven’t discussed?**

FP1: Have you been able to get results? You might not be able to tell us what they are but have you been able to draw any conclusions.

**I: I’ve not analysed yet so we’ve got centres still recruiting so we’re just finishing up with recruitment this month, end of September, so the follow-ups with all those patients will end just before Christmas probably so I won’t be properly analysing the data until the New Year. I’ll be analysing the patient interviews and focus groups between now and Christmas. [name] has got the wonderful job of watching lots of the videos.**

**FP: I haven’t done yours yet.**

FP1: Have you seen ours?

**FP: No I haven’t.**

MP1: Yes as we walked in it’s like oh yes it’s that one.

**I: Researcher number 3 here. I keep losing them as well because they keep having babies. It’s been going on for so long and there’s two little IMPS babies have been born as well.**

MP2: Implicit parenting, I like this.

**I: Yes so when I’ve got the results I will absolutely share them with you and will be very happy to come back and tell you about what we’ve found but I’m not quite there yet. Obviously it’s a pilot study so we won’t have results in terms of something was better than something else. It will just be more about the logistics of the protocol but also what [name] is doing with the analysis of the videos is literally counting and coding everything that is said so we will then be able to compare the intervention sites with the control sites and say was it even different to control and how was it different and did you talk less, did you use more external focus, what were the differences. So it’s more about the fidelity of applying it at this stage and then the patient views and obviously the staff views. So we’ll be able to feed all that back. I’ve got to do it all before June.**

MP2: I think it will be interesting because it’s difficult to categorise people isn’t it but in my head it would be nice to do a study where you had those populations that we talked about so in my mind it’s very difficult with receptive communication would be one group, another group would be cognitive and how you would include and exclude that would be difficult but is there a method by which we could say the cognitive impairment side of things was another group that you did that and then somebody that had perhaps neither of those and was purely a physical thing would be kind of where I would lean towards because you’ve got possibilities are endless. You could do acute versus rehab or you could do other things but that would be probably what I would lend it to and then what you measure which again I’m sure is something you are constantly thinking about is whether you measure the improvement over a set time or actually the speed by which they improve. Like I don’t know really, I don’t know what we would do. Or you do a really long study and you have people at the end of CSRT and say overall was there improvement within that.

**I: I think you are right, I mean there is something within speed because actually it might well be that the end point people get to isn’t different but the theoretical evidence and evidence in other populations would show people learn more robustly if they learn implicitly so you could look at speed of meeting milestones maybe or something like that.**

FP2: It has massive implications for an acute hospital. If you can.

**I: Time to being able to transfer with one or whatever it is, yes. You can look at some of those milestones and change. Yes ideally it’s the challenge in all rehabs what you need is a really, really massive study so then you can look at all of the data and look at the patient characteristics and work out OK because most studies don’t show difference in therapy because they’re not big enough and they don’t have the scope then to say well it might not show the difference when we chuck all the data together but if we just then look at the people with communication or communication and cognitive impairment then what does it tell us. That’s what we don’t have with rehab study.**

MP2: And you couldn’t do a length of stay in because unless you are doing it in a single site.

**I: No it’s a dodgy measure really.**

MP2: Exactly. We know that we can get people out quicker than somewhere else that doesn’t have any community service so it would be more that you draw the conclusions then be able to say that we reduced the length of stay like you are achieving the functional goals quicker thereby within this site you are likely to get there quicker.

FP2: I’m just sorry that we couldn’t complete it in its fullness really. I don’t think we had the capacity to carry on recruiting over this.

**I: I’ll turn this off.**

**END**
