# Supplementary material for: Understanding implementation of a complex intervention in a stroke rehabilitation research trial: A qualitative evaluation using Normalisation Process Theory
Source: PLoS One. 2023 Sep 8;18(9):e0282612. doi: 10.1371/journal.pone.0282612 (PMC10490858; doi:10.1371/journal.pone.0282612)
Supplement: S1 Data — (ZIP) [file pone.0282612.s004.zip › Supporting Information - Data/Interivew Transcripts - Control Group.docx]

| **INTERVIEW TRANSCRIPTION**  Voice file name: Site 1-A Discharge Interview  Duration: 14 mins 14 secs  Typist comments regarding dictation:  **KEY:**  **I – Interviewer**  FP – Female Participant |
| --- |

**I: So we are recording. If you want me to stop it at any point then you just do say. So you are the first person I’ve interviewed as well so I’m going to look at my list of questions otherwise I might forget what I need to say. So what I’m going to do is be asking you some questions about the rehabilitation that you’ve had since you’ve been in hospital. You’ve been here for about five weeks now?**

FP: Six weeks.

**I: Six weeks now. And I’m interested in hearing about the therapy, so physiotherapy mainly that’s focused predominantly on your leg, so on sitting and standing, stepping and walking. So that’s what we’re thinking about a little bit if that’s OK. So can you just start by telling me a little bit about what you’ve been doing in your physiotherapy sessions, what sorts of things have you done?**

FP: Goodness me. At the moment I’m doing standing up, leaning over and reaching and I have to lock my leg in and push my bottom back and sit down but that comes from holding onto the chair or the edge of the bed. And other things sort of the same but just standing up and leaning over to the right-hand side because I tend to go to the left-hand side. That’s when I fell when I had my stroke. So to stop me leaning on the left they’ve got me to lean over to the right side. I can’t really think of anything else.

**I: That’s OK. So that’s the things you’ve been doing most recently.**

FP: Most recently, yes.

**I: So most recently standing.**

FP: Up and standing and leaning over to the right keeping my stomach into the front bar and looking up. And trying to remember all those things.

**I: All those things to remember, OK. What are the main goals that you’ve been working towards within that? Have you had some.**

FP: To stand up straight.

**I: Standing up straight, yes.**

FP: To look up and be over to the right rather than to be over to the left.

**I: Yes, OK. Is there anything amongst all of that that’s been particularly difficult for you?**

FP: No because the people, not carers.

**I: The therapists.**

FP: The physiotherapists have been so good so it’s no problem.

**I: It’s not been a problem.**

FP: No.

**I: OK. And as you are doing you’ve mentioned a few things already but as you are practicing standing up from the bed and standing what do you focus on, what do you think about?**

FP: What I’m told to do really.

**I: What kinds of things have you been told to do?**

FP: They just say stare at the TV, which I told them was absolute rubbish because there’s stupid people on it, I hadn’t put the TV on.

**I: Oh OK.**

FP: So just to look and to lean over to the right and concentrate on leaning to the right and concentrate on my stomach coming up to the bar and to concentrate on locking my left leg which is the one that had the stroke. So I don’t really think about things I think about locking my leg and looking over and doing what they say.

**I: OK. So in terms of which bits of the movement you focus on its mainly on your leg and locking your leg.**

FP: Yes.

**I: When you say locking your leg can you just tell me what you mean by that?**

FP: Not just putting it straight but if I put it straight then I click it, it seems to click in. I can’t explain it. They use the term lock and I use the term lock because I can now tell when it’s locked in. It’s not just banding about going anywhere it’s locked in.

**I: OK so it’s making your leg strong and steady.**

FP: Strong and stiff yes.

**I: OK. Is there anything else that you do that helps you with your performance of those tasks?**

FP: Probably but I can’t remember, sorry.

**I: That’s alright, that’s fine.**

FP: I’ll pop over and ask the nurse.

**I: Oh no I want to know what you think not what they think. I’ll ask them another time. OK that’s fine. Just thinking about you mentioned the therapists have been really good at helping you to do those sorts of activities. Thinking about how they worked with you during those sessions how would you describe their approach, how would you describe what they’ve done?**

FP: Very good. Very optimistic. I completely trust them. They’ve got all the patience in the world, they are certainly helping me and they’ve helped me an awful lot and I’m very pleased with them for doing that.

**I: How have you felt yourself throughout those sessions?**

FP: As if I’m doing well, getting better. They’ve never ever said that’s no good, they’ve always praised whatever I’ve done so they’ve made me feel much better.

**I: Great. When you’ve been doing something like working on your standing and your leaning over to the right how do you feel when you are doing that?**

FP: The same. When I started I felt dreadful because I couldn’t do it but as I’ve worked on it I feel fine because I can do it now. I was just doing it before you came, I was holding onto the bars of the bed and leaning over and that’s no problem at all.

**I: So you feel more confident as time has gone on?**

FP: Yes.

**I: Can you think back to the beginning and how you felt at the beginning? Does it seem like a long time ago?**

FP: Probably completely hopeless. I just thought I could never do it and again because they were so boosting my morale all the time it was fine.

**I: Is there anything about your therapy sessions that you’ve had, your physio sessions, that you would have liked to be different?**

FP: No, I can’t think of anything.

**I: Just thinking a bit more specifically about how they’ve talked to you when you’ve been doing exercises so more about how they’ve given you instructions or feedback about what you are doing can you describe that, anything specific about anything you’ve noticed about how they’ve instructed you?**

FP: Very clear, very soft, very definite. And they’ve shown me what they want me to do because I don’t always get it straight away.

**I: So demonstrated it to you?**

FP: Yes.

**I: They did it themselves.**

FP: They’ve had all the patience to show me. Always telling me I’m doing brilliantly, which helps an awful lot to be told that rather than you are not doing well enough. If that’s what you wanted to hear. I mean it’s what I would say.

**I: Yes. Obviously it’s clear from what you are saying that their approach has been one that’s been really kind and supportive so that’s all great.**

FP: Yes [*6 mins 53 secs – can’t quite make out what was being said*].

**I: In terms of how they’ve almost coached you so I just wonder if when they’ve asked you to do something has it always been clear to you what you need to do or is it sometimes not clear, and that’s not a criticism of the therapist that’s just how we give instructions to patients if you’ve felt that they’ve.**

FP: A couple of times I’ve had to ask them exactly what they mean and they show me.

**I: Yes so then they show you.**

FP: Yes.

**I: OK. So anything about how they’ve talked to you that you would prefer to be different or has it all been OK?**

FP: All been OK.

**I: Have you felt you’ve had enough instructions and enough feedback about how you’ve been doing?**

FP: Yes, yes fine.

**I: And it’s been specific enough?**

FP: Yes.

**I: I think you’ve seen a few different therapists haven’t you on different days?**

FP: Yes.

**I: You’ve got a few of them that have worked with you. Anything that you’ve noticed about them whether or not they’re quite different or quite similar in their approach?**

FP: I think quite similar. I’ve had [name] and another girl mostly. [name] and I do know her name, not [name] and.

**I: Who have you had, [name] and. Oh I can’t think who it’s been actually. No I can’t think.**

FP: Again somebody.

**I: And a bit of [name] and a few others.**

FP: [*8 mins 21 secs*].

**I: And you think they’ve all been quite similar?**

FP: Yes they have.

**I: OK. Thinking back to the types of exercise and activities you’ve been doing how did you know if you were doing the movements in the right way?**

FP: Because they told me all the way along the line.

**I: OK.**

FP: If I went wrong they told me it’s not quite right we need you to push out a bit further or something like that.

**I: So they gave you feedback.**

FP: They were clear, yes.

**I: And was that helpful?**

FP: Yes definitely.

**I: And can you think of any examples of the sorts of things they’ve given you feedback on?**

FP: No I can’t.

**I: That’s alright.**

FP: A couple of times I was standing up and I wasn’t reaching quite where I supposed to reach and things like that. Not reaching out to that but reaching out in front of me, I wasn’t quite reaching out properly so they explained what they meant and I had to do that again. It’s all been perfectly clear.

**I: So nothing that you would have preferred to be different or more of or less of.**

FP: No. I would have liked to have had more of it all the time because I want to get better quickly.

**I: More therapy or more instructions?**

FP: No, more therapy.

**I: More therapy generally.**

FP: More therapy.

**I: Have you been doing any exercises outside of therapy?**

FP: No.

**I: So you’ve not been practicing anything when you are in bed or in your chair?**

FP: No only my hand, clicking my fingers and moving my arm up and down but they didn’t tell me to do that.

**I: You’ve just been doing that. But nothing specifically for your legs?**

FP: No.

**I: OK. How do you feel generally about the progress you’ve made in the last six weeks on the stroke unit?**

FP: OK. I wouldn’t have progressed anything like as far if I hadn’t had the physio I’m sure I wouldn’t. Every time they come in and say do you want physio it’s yes!

**I: Yes, don’t say no.**

FP: I want to get better so I can go home.

**I: What makes you feel that way about your progress do you think?**

FP: How do you mean?

**I: So do you think back to what you were like when you first came in? Have there been clear goals that have helped you know you are progressing or is it just a general feel that you are getting better?**

FP: I think general feel but yes also goals because my daughter-in-law is in this sort of thing and she comes and tells me what to do with my hands and things. And I add that to what I’ve been taught here so I do that as well.

**I: Good. Nearly there, last couple of questions. This might be a tricky question I don’t know, we’ll see. So if you were thinking about your physiotherapy sessions what words would you use to describe them?**

FP: Helpful. Clear. Maybe not long enough, as long as I would have liked but they have explained that it’s probably enough for me because I’ll get tired on it. I think that’s about all.

**I: So if you were talking to your family about your therapy sessions how would you describe them?**

FP: Very good. Very helpful. I look forward to seeing the girls when they come. They always come in looking very sheepish and I say oh it’s you again but I’m always pleased to see them.

**I: And there’s going to be more of that when you go home isn’t there, it will keep going the therapy.**

FP: Well I’m told, I only just found out that I’ll have more therapy at home.

**I: Yes.**

FP: Which is brilliant.

**I: It absolutely needs to carry on. The time comes to go home but that doesn’t mean the therapy needs to stop. So just finally is there anything else about your physiotherapy that you would want to tell me about?**

FP: No I don’t think so just a pity these sessions are so short and also they’re not in a definite basis the girls, often they just turned up when my sons turned up to take me out.

**I: Oh I see.**

FP: And things like that.

**I: So it’s a little bit ad-hoc when it happens.**

FP: My son won’t even hear of my going out with him, the physio was far more important, he’s a physio. But you think there would be more structured it would have helped me.

**I: If you knew what was happening and when a bit more.**

FP: If I knew when they’re coming and how long it’s going to last. That’s a very definite one that I wanted to get in.

**I: OK.**

FP: I’d forgotten about that.

**I: We can feed that back.**

FP: But I have spoken to them about it and they said they can’t tell what’s happening or when.

**I: It’s difficult sometimes because a ward is busy and things change every day, but we probably could be better at that so we can think about how we could do that better.**

FP: If they at 9am in the morning and say we’re coming to you at 10 or 11 or 12.

**I: So even if they let you know on the day.**

FP: 15 minutes or 20 minutes. The day is fine, I don’t know need to know before the day but that would be helpful. That’s just one thing I have discovered.

**I: I guess otherwise you are kind of sat waiting and wondering aren’t you wondering when they’re coming.**

FP: Just wondering and then my son turns up and I’m in the middle of getting in my chair to go outside and they turn up. My son has come all this way to see me and I’ve had to say to him no I’ve got to see the physios.

**I: Which is tricky for you. OK. Thank you. So we’re all done. I’ll turn this off.**

FP: I hope my speech wasn’t very bad.

**I: Oh no your speech is very good. END**

| **INTERVIEW TRANSCRIPTION**  Voice file name: Site 1F Discharge Interview  Duration: 18 mins 38 secs  Typist comments regarding dictation:  **KEY:**  **I – Interviewer**  FP - Female Participant |
| --- |

**I: So what I wanted to do today because you are going home fairly soon, hopefully next week, is just ask you some questions about the rehab you’ve had in hospital particularly the physiotherapy that’s been working on you getting to where you are today with your walking. I’m particularly interested in the standing, stepping, walking side of things. There’s no right or wrong thing to say and what I’m asking you questions about is to do what you’ve done in therapy and your perception of that so not necessarily about whether the service you’ve received is good or bad, you can tell me about that but it’s actually what’s gone on in your therapy and how do you perceive what’s happened in therapy. So just to start off can you just tell me a bit about what you’ve been doing in your physiotherapy sessions, can you give me some.**

FP: Sitting and standing. Trying to keep my balance central. What did I do? Oh well we’ve gone through various pieces of equipment. I’ve had a go on the hoist to start with.

**I: So that’s where you were at the beginning.**

FP: And then I had a go with the two bars side by side and we’ve now progressed to, I had a gutter.

**I: The one that’s by your bed.**

FP: Yes. Then yesterday I tried a Zimmer. If anything I think I was probably better on the Zimmer than with the gutter thing although it looks bigger and sturdier.

**I: It’s just a little bit wobbly isn’t it the gutter frame sometimes.**

FP: It is a bit wobbly, yes.

**I: And it’s high.**

FP: And today I’ve had a go with a quad stick.

**I: Ah, great.**

FP: Well mainly because my thumb on the right hand isn’t really doing what I want it to do, I’m not aware of it so much. There is pain in it but it keeps slipping off everything. It’s nicknamed Rita.

**I: We have to clarify that for the tape.**

FP: I’m afraid Rita throws her toys out the pram occasionally. As you can see I’ve got a bruise which I think was from probably in the night flinging my arm up so it’s a matter of trying to control that hand. Somehow I’ve got quite a lot of strength in it but it’s controlling the strength.

**I: Yes so it’s doing what you want it to do.**

FP: It goes a little bit wild. So I’ve been very impressed really, they’ve tried so many things with me.

**I: And you’ve clearly progressed, that’s a really good stepping-stone from being on the hoist to now being able to at least even just be trying with the quad stick.**

FP: Unfortunately my right knee is not right. Right knee isn’t right.

**I: In what way?**

FP: Well as far as I can remember my leg never has been straight at the knee, it’s always gone back. If I look at myself in the mirror the right knee would always be further back than the left knee and of course it’s not holding me so well. Now and again it flicks badly and you know can throw me off balance.

**I: OK.**

FP: So they’re looking at, I think they’re going to look at orthotics to see if they can suggest any sort of support that might help.

**I: Great.**

FP: They’ve tried everything, I can’t say that I’ve been neglected at all.

**I: Oh no, good.**

FP: They’ve been wonderful.

**I: So you’ve described your progress and needing the equipment that was really helping you a lot to now being able to do much more for yourself. When you’ve had therapy sessions, so when you’ve been in the gym or the garden room have you been doing exercises?**

FP: Oh yes.

**I: What other kind of things have you done?**

FP: Well a lot of hand exercises for a start. Not so many leg although I’ve gone to the exercise classes which has involved picking the foot up and down and bending the knee so I have been having exercises but it’s not so easy to do leg exercises as arm exercises when you are sitting in bed or in a chair.

**I: When you are by yourself.**

FP: Yes. I’ve had a childish game where you pick up shapes, wooden shapes and put them into necessary holes you know. A two-year-old would do it in five minutes, I’m afraid my hand tends to throw them on the floor quite often.

**I: But you are persevering.**

FP: Well I’m trying to.

**I: You’re trying to persevere. For your legs in particular what are the goals that you’ve been working towards?**

FP: Standing up and keeping my balance and actually moving the feet.

**I: Stepping, working towards stepping.**

FP: Yes. So I’m stepping a bit but I have to be reminded to think about my feet, where I’m putting them. My left foot is alright because that’s automatic but it’s thinking where I’m putting my right foot.

**I: So you’ve been focusing on that in particular. And your knee you mentioned as well.**

FP: Yes it’s that right knee and right foot that are going a little bit astray.

**I: Are they the two things that are particularly difficult would you say?**

FP: Yes.

**I: When it comes to stepping.**

FP: Actually I’ve got orthotic shoes because I’ve got a problem with my left leg as well. I split the tendon in my left leg.

**I: Oh gosh.**

FP: I broke it to start with, the tibialis posterior tendon, broke that about 20 or 30 years ago. It was repaired but I don’t think the repair was as good as it might have been.

**I: So it’s never been 100%.**

FP: Then I started getting a lot of pain in that leg and swelling. The doctor thought at first it was a DVT but it wasn’t and I had a CT scan which showed that in fact I’d split the tendon from the ankle had split up my leg.

**I: Oh gosh.**

FP: So of course that doesn’t do me good. I was putting a lot of pressure on the right side, my right leg which was hurting and I was having massage for that before I came in. It’s been one thing after another.

**I: Not quite got two tip top legs.**

FP: No, no.

**I: To start with. But it’s the right leg that’s affected by the stroke.**

FP: It is the right leg.

**I: Just so we remember when we listen back.**

FP: That’s a bit painful at times.

**I: So thinking about because you are doing some walking now aren’t you, so when you.**

FP: Trying to.

**I: I’ve heard that you are. So when you first were starting to work on standing up you said balance was something you needed to work on is there anything else that you’ve had to focus on specifically with the stepping?**

FP: As I say the knee doesn’t always go quite where it should be. I have to think about trying to keep the knee straight.

**I: So when you are putting your weight on it.**

FP: I’ve had different ideas, one therapist said you must bend your knee slightly and take the weight but that doesn’t work. The other one accepted that the knee clonks back because it does actually go clonk now and again. You can hear me coming, can’t you.

**I: Yes. So this is when you are standing on the leg when all your weight is on it it clonks back.**

FP: Hmm.

**I: Yes, OK. And then when you try to take a step what do you think about when you are trying to take a step?**

FP: Where I’m putting the foot. Sometimes I can lift it and move it, other times I drag it. It doesn’t want to come off the floor easily.

**I: OK. Have you found any particular techniques that help you with that?**

FP: Not really only thinking, well thinking more of balance I think than anything. Trying to keep the leg in a position so that the knee won’t flop back.

**I: So when you are doing those stepping.**

FP: I feel quite happy just standing, getting up from the chair here although she said put that hand on there and it will go clonk like that.

**I: It slips off.**

FP: Slips off, it won’t hold on. My legs feel, I think there’s quite enough strength in there to hold me, I can get up and down quite easily.

**I: OK. So when you are standing up from the chair is there much that you have to think about then?**

FP: Not really as long as I am getting my weight in the right place. Get my feet level and get my weight forward over the legs and then use my legs to push up. I don’t know if that’s my technique but it works for me.

**I: It’s working, exactly it’s working. Perfect. If you think about the therapists have you seen quite a few different therapists?**

FP: Yes.

**I: Do you know a few of them. How would you describe their approach in therapy again just thinking particularly about the leg side of things.**

FP: Slight difference between them I think. All very good but some have, who have I been with today, I can’t think now.

**I: Did you have Emily? Emily today? No Emily doesn’t work on a Friday so it wouldn’t be Emily.**

FP: No not Emily. I don’t know that I’ve seen her before. I had Ellie to start with and who else have I seen. I’m terrible with names.

**I: Oh no don’t worry.**

FP: There are so many nurses and they’re all different names and I can’t remember who is who.

**I: So when you said they’re a little bit different in their approach and things in what way are they different?**

FP: One of them was very keen for me to try and stand up but not to let the knee go back. But whoever I’ve had the last couple of days has said well if you knee does that it does that sort of thing so I haven’t bothered too much about it.

**I: OK so a little bit different in what their focus might be.**

FP: Yes.

**I: OK. In terms of how they’ve given you instructions and feedback can you describe that? What do you think about that?**

FP: I think they’ve all been pretty clear on what they want me to do.

**I: So specific in the instructions they give? It might be hard to remember, don’t worry.**

FP: I’m just trying to think what sort of instructions do they give. No I think just a reminder now and again keep your weight this way or that way, you are leaning to the right try and go to the, you know, which is helpful.

**I: Do they give you feedback so you know whether or not you’ve done it in the way that they’re asking?**

FP: They all say I’m doing well.

**I: OK.**

FP: And I said is that hospital speak to keep you going.

**I: So kind of encouraging.**

FP: Very encouraging yes, they’ve been very good. It raises the spirits a bit and the hopes.

**I: Do they give you specific feedback though about maybe what your knee is doing or what your foot is doing?**

FP: Yes concentrate on this and that sort of. I think the latest one is holding on and just leaning forward on the chair and trying to stand. If not standing right up at least take my weight on my feet.

**I: So you have, I’m trying not to say it in a leading way but do you have a fair amount to think about when you are doing these things do you think, when you are practising exercises are you quite.**

FP: To be honest there isn’t that much time to be able to do much in the way of leg exercises because they involve taking your weight and standing and I’ve been told I can’t do that on my own, you’ve got to have someone with you so it’s very difficult to practice anything by yourself.

**I: I was thinking more in therapy when the therapists are with you or the nurses are with you or anybody like that.**

FP: When I’ve been in therapy they’ve been very good at giving instructions.

**I: Is there anything in particular you would have liked to be different about the way they’ve delivered your therapy?**

FP: No, wave a magic wand.

**I: Make it better quicker.**

FP: Yes.

**I: Yes. OK.**

FP: No I think they’ve all been excellent, they’ve been very helpful.

**I: OK.**

FP: This is why I wanted to stay on longer so I get the therapy because obviously once I get out I’m not going to get as specialised knowledge.

**I: Hopefully there’s a plan, I don’t quite know what the plan is but for you having some ongoing therapy when you leave hospital. Have they talked about that?**

FP: They’ve said I get two weeks of daily therapy I think and I’m going to Sunrise at West Bourne because apparently they have got some medical staff there, nurses and that who might be better.

**I: Yes, OK.**

FP: I’m wondering if that’s more because of the diabetes.

**I: They can all support with that as well. So we’ve talked a little bit about this already but doing exercises outside of therapy so when you are not with the actual therapists you feel it’s been more difficult particularly for your leg because.**

FP: Yes that’s right. It is difficult isn’t it to say to somebody exercise your legs.

**I: But by the way you can’t stand up.**

FP: Well other than just sort of practising bending the knees and that.

**I: Have you been given any specific leg exercises or have you just worked a few things out yourself?**

FP: Only one which was knees up and trying to bend side to side slowly in bed. But again that’s not really very easy when you are in bed.

**I: No. OK.**

FP: But I’ve got to the point where I’m pretty well able to sling my legs over the side of the bed to stand on the RJ thingy. The sack truck as I call it.

**I: Sack truck. Good. So overall how do you feel about the progress you’ve made since you’ve been here on the stroke unit. How long have you been here?**

FP: About five or six weeks.

**I: So how do you feel about the progress?**

FP: Well I’ve progressed a lot obviously.

**I: Yes you have.**

FP: Whether it’s the usual progress I don’t know because as you say different people go at different rates you know so I don’t really know what I should have done or shouldn’t have done. I can only go on people saying well you are doing extremely well and you couldn’t do that last time I saw you, that kind of thing.

**I: We’ve seen that in the measures we’ve done for the research as well that because we do them every two weeks actually it’s quite a nice, you have you need to believe that you’ve made really good progress here. I think this week being able to even just try the quad stick has surprised some of the therapists.**

FP: I was surprised when they said try this.

**I: Yes.**

FP: I do feel as if I need something on the righthand side as well, another stick or something.

**I: Yes you feel a bit odd being one-sided.**

FP: Yes.

**I: OK. So last couple of questions, so if you were telling your family about your therapy sessions and you have loads of family visits so you might have told them.**

FP: Yes I’ve told them.

**I: How do you describe it to them then, what words would you use to describe therapy or physiotherapy?**

FP: Just tell them I think the girls are doing a grand job and really encouraging. But they can see for themselves, I mean my granddaughter, one granddaughter was here the other day when I was trying to walk and she videoed it herself and sent it round the family.

**I: Oh did she.**

FP: They’ve got a family video, Grandma’s progress.

**I: Ah what a lovely idea. How do you feel about that?**

FP: Great. I don’t mind at all. I’ve had so many jokes about it, they’re a great family.

**I: They’re obviously very proud of what you are doing. In your therapy sessions so regardless of what you are doing how do you feel during therapy? Could you describe if you were to use feeling words.**

FP: Feeling words.

**I: Yes, tricky.**

FP: Little bit anxious, hopeful. And at least they will listen to me I’ll say that for them. I’ve said do you think I need a brace or something on my knee and they immediately went and fitted a brace to try it and unfortunately it didn’t fit very well.

**I: It wasn’t quite right.**

FP: It didn’t really do much for me.

**I: So you feel that you’ve been involved in your therapy.**

FP: Yes they’ve asked any ideas and how do you feel and what hurts and what doesn’t hurt kind of thing.

**I: Yes. Fine. Is there anything else, anything else about your physiotherapy that you feel stands out or that you want to tell me about?**

FP: I just want to thank them all.

**I: You can do that next week.**

FP: They really have been lovely. They are obviously dedicated to their jobs you know.

**I: They get a lot of satisfaction out of seeing people like you who work hard with them. It’s two ways.**

FP: One of them said that yesterday.

**I: Absolutely.**

FP: The plumpish girl.

**I: Who was here yesterday. Thursday. I’m not sure. Physio? I don’t know.**

FP: And we’ve had some laughs about it. This morning I said this is flying by the seat of your pants because she was hanging onto my trousers at the back.

**I: They live to tell the tale. Fine. Super. Right I’m going to stop the recorder. That’s all my little questions.**

**END**

| **INTERVIEW TRANSCRIPTION**  Voice file name: Site 1-H Discharge Interview  Duration: 11 mins 45 secs  Typist comments regarding dictation: A couple of places where I couldn’t quite make out what was being said.  **KEY:**  **I – Interviewer**  MP – Male Participant |
| --- |

**I: So this is just some questions about the rehabilitation you’ve had whilst you’ve been in hospital. So how long have you been here now?**

MP: What this time?

**I: Yes since you had this stroke.**

MP: Oh four months, three months something like that.

**I: I can’t remember when you came in.**

MP: I can’t remember.

**I: Three or four weeks maybe. Does it feel like longer?**

MP: Feels like longer.

**I: I’m particularly interested in the physiotherapy you’ve been having that’s been focusing on your legs. So all the things you’ve been doing to focus on being able to sit, get out of bed, get into a chair, take some steps with the Zimmer frame so that’s what we’re mainly thinking about a little bit.**

MP: Yes.

**I: So if you think about your physiotherapy sessions can you just tell me a bit about what you’ve been doing in those sessions?**

MP: Well mostly walking I should think.

**I: Yes.**

MP: Turning. They do get a bit enthusiastic and they might say.

**I: Who do the physios?**

MP: Do these five times or something. Well that’s marvellous we’ll do another five.

**I: Ah, OK.**

MP: And sometimes it’s a bit too much.

**I: OK.**

MP: Normally it’s alright.

**I: So you’ve been working on being able to do some stepping.**

MP: Stepping, sitting.

**I: OK. Can you give me any examples of the kinds of exercises they’ve done with you?**

MP: Getting up out of chairs. How to hold a table when you get up. Can’t think of anything else.

**I: No OK. So when you think of something like practising getting out of a chair so standing up, sitting down you’ve probably done lots of that. When you are doing that what are you focusing on, what are you thinking about?**

MP: Well what they’ve told me and then normally I’m looking out the window and if I’m not careful if I’ve got my eye on a bird it’s hands on the arms of the chairs, standing upright, taking two small steps forward and then holding the table.

**I: OK, yes. Has any of that been particularly difficult for you? How have you found it?**

MP: Probably getting out of the chair to stand up, sometimes I’m a bit wobbly.

**I: OK you feel unsteady. OK. You are using a Zimmer frame aren’t you to get out over to the chair.**

MP: Yes here I’m using it. I haven’t been home much really to use it.

**I: OK is that quite a new thing? Yes. But when you are doing that how do you find that?**

MP: Oh very easy, yes.

**I: Anything particular you think about whilst you are using the frame?**

MP: Getting my feet right. My left leg is although I had the stroke months and months ago my left leg is always a bit stiffer.

**I: OK. So when you say getting your feet right what do you have to do with your feet?**

MP: Well the left one has got to be facing that way.

**I: Facing forwards.**

MP: Because that is my, I’m just thinking which one I’ve broken on my motorbike, my left one, and when I get up I’ve got to make sure it’s the right way.

**I: Yes so you think about the angle of your foot and where it is on the floor. That’s good. When you are taking some steps is there anything about that movement that you focus on?**

MP: Not really. Just make sure that I’ve got my feet facing the right way.

**I: Then you’re good to go. If you think about the physiotherapists who’ve worked with you in your sessions how would you describe their approach?**

MP: Over enthusiastic.

**I: What do you mean by that?**

MP: Well as I said, oh you’re doing fine, oh that’s lovely now do another five. So then the next day I have two paracetamol.

**I: OK so they worked you quite hard.**

MP: Oh yes. I mustn’t be too enthusiastic myself.

**I: OK. How do you feel about that?**

MP: I can understand their point. As many times as they can get patients through them it’s good for them but then I think well why should I do that then be in pain the next day.

**I: A bit reluctant with some of that. So is there anything you would have liked to be different about their approach?**

MP: Not really. I’d like to be able to say well sorry that’s enough but I don’t like to be unkind to them and I’ll do it but then the next day I’m on paracetamol.

**I: Would you have liked to have been a bit more in control of how much you were doing is that what you are meaning?**

MP: Yes.

**I: OK. If you think particularly about how they talked to you, so how they gave you instructions or they gave you feedback about what you are doing.**

MP: Oh very good really because they come round and I go I don’t want them [*5 mins 41 secs*] but I have a little talk and then off they go. They’re not too persuasive. I can be led but I can’t be pushed and if I think they’re pushing me I cut it straight off.

**I: When you are actually in a session is there anything about how they were giving you instructions about the exercises or how they give you feedback about how you were doing that you can recall?**

MP: No just these blasted balloons.

**I: Oh right, patting a balloon.**

MP: They drive me up the wall. But they’re very kind really.

**I: So how did you know if you were doing the right thing when you are doing the task or the exercise?**

MP: They don’t say really they just say you are good, that’s all. How many times [*6 mins 24 secs*] to somebody and it’s quite good.

**I: And not too specific.**

MP: I wouldn’t go over the wall to say what a marvellous thing it is.

**I: So this is more really about actually what you were doing in your therapy as well and how you felt about that. Was it always clear what they were asking of you?**

MP: Oh yes, yes.

**I: Yes so you knew what.**

MP: They’re very good.

**I: OK. And when you were doing something, so for example if you were practising with your Zimmer frame maybe getting into the chair how would you know if you were doing it in the right way, did they tell you anything?**

MP: Don’t do it like that you’ll fall over. And I did it this morning and nearly fell over.

**I: Did you, bit wobbly this morning.**

MP: Getting from the Zimmer to my bed.

**I: OK.**

MP: I knew I’d done it wrong.

**I: Was that with the nurses this morning or?**

MP: That was the nurse was sat talking to somebody else and she said don’t and before she could say don’t do it.

**I: Ah were you trying to do it for yourself were you.**

MP: Yes.

**I: OK.**

MP: I’m a bit pig-headed sometimes.

**I: And have you been doing any exercises by yourself so like when you are here at your bed or your chair?**

MP: Oh yes, yes.

**I: What kind of things?**

MP: Doing that really but I should take my time and I think I can do that but I’m doing it too quick. But then I’ve got to the point where if I can’t do it I’ll sit down and then get somebody to help me.

**I: Yes, OK. So are you doing things that you’ve thought up yourself or did the therapist give you some bits to practice?**

MP: They give me some bits to practice because what I think is probably different to what they think but they’ve got more experience than me anyway.

**I: So how have you found that doing your own practice?**

MP: Well I think what they would say.

**I: Do you feel you’ve spent enough time with them to have a think about what they would say?**

MP: I mean if they were to climb a ladder I would tell them straight away whether they were doing it right or wrong because you shouldn’t. You see a fireman he’s never holding the rungs.

**I: Oh yes.**

MP: Because if [*8 mins 39 secs*] you hold the sides and the way they explain it to you it’s a good way they do it.

**I: That’s good. Any other techniques you use for yourself that help you with your movements of your legs.**

MP: Well yes I’m always twiddling my toes. When I wake up in the morning I look at.

**I: You having wiggle.**

MP: And I try and walk as much as I can but I do get tired so I don’t get over tired now. Like if you saw me now I can go to sleep just like that so I relax more than I used to.

**I: OK. That’s good. How do you feel about the progress you’ve made since you’ve been here in hospital since you had the stroke?**

MP: I’m not fast enough.

**I: You feel the progress isn’t fast enough or?**

MP: Yes my progress. They’re marvellous but I think next day I should be walking and then if I, when was it I was in the other hospital, where was that? New Milton way or was it Bournemouth. When I came back here I hadn’t walked for a day and I was wobbly. Because if I can walk one day I think marvellous I’ll be running tomorrow but I’m not running tomorrow so I’m a bit impatient.

**I: It sounds like you are quite a determined personality and it feels a bit slow.**

MP: I was 7 months in plaster on my leg when I broke it with a motorbike but within three days I got a piece of wood and I was driving my lorry.

**I: Oh my goodness. Oh dear.**

MP: So I am determined.

**I: You might not want to get that on tape.**

MP: No.

**I: If you were telling, do you have some visitors come and see you sometimes? Do you have family visit?**

MP: Yes.

**I: So do you tell them at all about what you’ve been doing in therapy?**

MP: Well yes but I don’t really bore them.

**I: I just wondered how you would describe it to them.**

MP: Oh very good you know. These keep fit people keep coming in.

**I: For the group.**

MP: There’s two with beards and I call them terrible twins.

**I: Oh yes I know who you mean.**

MP: I think one has gone away, I think he’s going to Malaya or the jungle or somewhere. And I say here come the terrible twins and I just go away and they go away and then they come back. They’re very good, it’s me.

**I: So you’d describe it as good. Anything else about how you’ve been working in therapy?**

MP: Oh yes they are good, I mean they’re good at their job but half the time when they come like when you came I was.

**I: You were resting weren’t you.**

MP: But when they come oh.

**I: Yes, OK. That’s fine, that’s all of my questions really unless there’s anything else particularly about your therapy that you think is pertinent?**

MP: No.

**I: OK. I’ll turn this off.**

**END**

| **INTERVIEW TRANSCRIPTION**  Voice file name: Site 1L Discharge Interview  Duration: 11 mins 41 secs  Typist comments regarding dictation:  **KEY:**  **I – Interviewer**  FP – Female Participant |
| --- |

**I: So if it’s OK with you I just want to ask you a few questions about what you’ve been doing in rehabilitation particularly what you’ve been doing with the physios to work on your leg strength. So this is more focused on that than on your arm because I know you’ve been working on both of those things haven’t you. And it’s less about the therapists really but more about the sorts of things you’ve been doing and what you’ve thought about that and felt about that. So there’s no right or wrong thing to say it’s just to get some of your thoughts.**

FP: They’ve all been very good and everything that you’ve done has helped me.

**I: So can you remember because you’ve been in hospital for just over two weeks now, is that right?**

FP: Yes.

**I: And can you remember when you first came in how the stroke had affected you or is that a bit of a blur?**

FP: It was all down one side of my face and my arm and my hand, my leg and my foot.

**I: Could you.**

FP: And weakness.

**I: Weakness. Could you move your leg and your foot at all when you first came in do you remember?**

FP: I thought I could a little bit but not as strong, so much stronger now.

**I: Were you able to do things like sit up by yourself and get out of bed and into the chair when you first came in?**

FP: No I don’t think I was.

**I: No, OK. So I think you were certainly having some help getting in and out of bed, I can’t remember if you were sitting.**

FP: Yes definitely.

**I: OK. So in the therapy that you’ve been having over the last few weeks when you’ve seen the physios what are the main things you’ve been working on?**

FP: Everything on my left side obviously. Mobility of getting me going and moving and the fact that I can walk now is amazing because I didn’t think I would. [*2 mins*] had in my mind I just want to get up from the chair and walk like I could normally. And it just seemed why can’t you do it, you know.

**I: So walking was one of the big goals was it for you?**

FP: Yes definitely.

**I: And any other things linked to your leg that you’ve been working on particularly? What have you needed to do to improve your stepping and walking?**

FP: Just got to keep up the exercises to get me stronger. And it does pay off. You think it’s not going to but then you can see the improvement. You can see the improvement more than I can you know but I can because I can get up from the chair and I can move about a bit.

**I: So you can see the benefits.**

FP: Oh definitely.

**I: Of what you’ve been doing. Is there anything in particular you’ve been focusing on in your therapy to do with your leg?**

FP: It’s just remembering you’ve got to stand tall and lock your leg.

**I: OK so remembering to stand tall and lock your leg – what do you mean by lock your leg?**

FP: Lock in place, straighten it.

**I: OK.**

FP: Say lock my leg but I mean straighten my leg.

**I: When you are standing. And because you are doing some walking now which is amazing using a quad stick aren’t you.**

FP: Yes, yes.

**I: What do you need to focus on when you are walking?**

FP: I get too distracted easily.

**I: Do you.**

FP: Yes I’m terrible. I’ve only got to hear a noise and I’m turning my head.

**I: So distracted by what’s going on around you.**

FP: Yes very much so. I need blinkers on, I need whatsaname to stop me from looking at things and hearing a noise and everything. That’s hard.

**I: So do you have to try and concentrate on what you are trying to do.**

FP: What I’m trying to do but it’s really difficult. I find it really difficult because I’m a nosey bugger.

**I: Have you always been nosey.**

FP: Obviously I must have been. If I hear something or something I’ve got to see what it was or who it was.

**I: With the work, so you’ve been working on stepping and now doing some walking is there anything about the movement that you focus on as you do that?**

FP: [*4 mins 50 secs*] my knees up. Placing my feet in the right places.

**I: So do you tend to watch where your feet are going?**

FP: I need to do that more.

**I: OK.**

FP: Think about where I’m putting my feet.

**I: OK. Anything else that you think about maybe as you are standing up and as you are stepping?**

FP: Hold your head up but not too high obviously otherwise you might go backwards. I suppose it would be good if you had a thingmabob to aim for to look at on the wall or something.

**I: Is that something that you’ve done in therapy?**

FP: No, I just been thinking of it here and there.

**I: OK. Having something that’s at the right level to look at.**

FP: Something to think you’ve got to keep your head up otherwise you won’t see where. I don’t know whether that would help but it’s just an idea.

**I: Yes, OK. Anything else you think about whilst you are practising movements with your leg?**

FP: Won’t be long and you’ll be able to go home.

**I: Yes, OK. So you are thinking about the overall goal.**

FP: Yes getting home is the goal.

**I: And that this is all to help with getting home.**

FP: Yes.

**I: Absolutely.**

FP: Something else ticked off the list. I don’t know how many more things I’ve got to tick off.

**I: I think you’re getting there.**

FP: I’m trying anyway.

**I: If you think about the therapists, the physios you’ve worked with so you’ve had a few different physios working with you haven’t you during your therapy sessions, how would you describe their approach with you?**

FP: They’ve all been very good, very kind and very understanding. I mean they must get a bit frustrated when somebody doesn’t do what you say to them and people are telling you things and you feel as though it’s not really going in. You know what I mean? That’s how I feel when you say we’re going to do this and that and I think oh my God how am I going to do this.

**I: So when they’re telling you things you mean about what you need to do.**

FP: Yes.

**I: What kind of things do they say?**

FP: I don’t know but it’s just like I say well we’re going to do this now, we’re going to stand up now. Like you just said about walking and I’m thinking oh my goodness how am I going to do this now.

**I: OK.**

FP: Am I going to manage this. You just want to get up and run, you know what I mean, run to the end of the thing.

**I: Like you would have done before.**

FP: Or walk quick anyway not run but walk quickly.

**I: Is there anything particularly about how they’ve talked to you that you think is good? Has it always been clear what they want you to do?**

FP: Yes oh yes but it just feels as though it’s not going in if you know what I mean.

**I: OK. A lot of information to process?**

FP: Not really but it just seems like oh my goodness, you know.

**I: OK.**

FP: It’s nothing against what any of you have been doing it’s absolutely been marvellous, all of you. I couldn’t highly recommend any of you enough.

**I: OK. If you think about how they give you instructions in your therapy, how they tell you what it is they want you to work on has that always been clear to you. What would you say about that?**

FP: Yes.

**I: It’s always clear?**

FP: What they say, yes. It’s whether I process it that way I don’t know.

**I: No, OK. How about feedback, so do they give you feedback on how you are getting on? What has that been like?**

FP: They always say you are doing very well, you are doing very well, come on you are doing really well. I just think it can’t be me they’re talking about. You know what I mean? You just think that’s not me they’re on about, I can’t walk down the end of the corridor without any help or a stick.

**I: So has the feedback been specific or has it been more general as in you are doing well overall?**

FP: They say you are doing really well, even the doctor said I’ve had good reports of you. You are doing really well.

**I: Is there anything that would have helped more or made that better?**

FP: No.

**I: OK. Have you been doing some exercises when you are by yourself so not with the therapists?**

FP: Yes I’ve been doing some like this and that.

**I: So lifting your foot up and down. Bending your knee when you are sitting.**

FP: Yes when I’m sitting in the wheelchair or sitting in the seat or my bed and turning it around just keeping it loose if I can.

**I: Are they exercises that have been given to you by the therapists or have you worked them out yourself?**

FP: It’s in a leaflet that was on the bed when I come back one day it was there and it said when you are sitting you can do these things and picture of what to do and I just thought well that’s a good idea.

**I: What do you think about when you do those kinds of exercises?**

FP: Come on keep going, this is getting nearer to getting out. The more you get your legs moving and stuff.

**I: I’m just repeating that in case it didn’t hear it. So getting nearer to getting out, getting home and getting your legs moving so you are just thinking about that overall goal again. Brilliant. Then last few questions about how you feel generally about the progress you’ve made since you’ve been here?**

FP: I’ve done really well. I hope I have anyway. I feel I have considering I thought I can’t get out of the seat and walk up and down and now I can.

**I: So you feel positive overall?**

FP: Yes, oh yes definitely.

**I: Great, that’s good. The last question is if you had to describe your physio sessions to somebody who hadn’t seen you for a while in three words what words would you use to describe them?** **That’s a tricky question.**

FP: Listen to what they say and do what they say and get on and do it and you’ll be fine.

**I: Yes, OK. Perfect, thank you.**

**END**

| **INTERVIEW TRANSCRIPTION**  Voice file name: 1I Discharge Interview  Duration: 14 mins 50 secs  Typist comments regarding dictation:  **KEY:**  **I – Interviewer**  FP – Female Participant |
| --- |

**I: So as I was saying I’m just going to ask you some questions about the rehab you’ve been receiving and we’re particularly thinking about the physio and the work you‘ve been doing on your legs. I know you’ve been working on your arm and other things as well. So anything from when you were working at the beginning on sitting, standing up, stepping, walking that’s what we’re having a bit of a think about. So you were telling me a bit as you came in but can you tell me about what you’ve been doing in therapy with regards to your leg recovery?**

FP: So on the bed with Kim we’ve been doing work with making the legs both go out and in which has been quite good. Every time we’ve done it this leg has become more controlled because she holds her hand there and I try not to touch it and pull it back, so you can see where I can’t quite.

**I: Yes so working on the, I don’t know what that would be called, so we would call that external rotation just because that can’t see your demo.**

FP: External rotation.

**I: So working on your control of your leg then.**

FP: Yes. And then we do I call them bum lifts and I can hold for the count of five. She said three seconds and I went oh I can do it for five, so yes we did that on the bed. And also with the slide sheet so sliding the leg out.

**I: So straightening it out and bending up.**

FP: Yes and up. I’m quite good at sliding out but it’s still very difficult to slide it back.

**I: OK.**

FP: So that was on the bed. Obviously walking with the walker, yes I can do, I’m pretty much independent now on the walker for when I get to my chair just have to feel for the back of my chair, put my hands on the arms and sit down slowly not flop down. So yes we’ve done all that. Then the other important one was the work on the steps. So yes that went really well and I managed it which I didn’t think I would.

**I: So you’ve achieved a lot. So if you think back to what was difficult when you first came in just over a couple of weeks ago.**

FP: I know.

**I: Can you describe what you were like and what had difficulty with when you first came into hospital?**

FP: Everything really. Everything. I was really worried and obviously my confidence was pretty low but with all the physio that I’ve been getting my confidence has just built especially obviously because I’ve got quite a lot of stairs at home and I now feel that even if it takes me an hour to get up them I could still manage them. I don’t care how long it takes me it’s the fact that I feel that I can manage them which is the main thing. It is confidence building just walking around, washing myself, getting myself to the bathroom and toilet, getting myself in and out of bed. Getting myself in and out bed that’s another one; I did quite a lot of work on that initially. Yes.

**I: So when you first came in could you move your leg at all? I can’t remember.**

FP: No I don’t think I could.

**I: It was completely weak.**

FP: Yes it was, I couldn’t even wiggle my toes but now I’m.

**I: It’s all coming back.**

FP: It is coming back. Obviously there are certain things that I am finding difficult but hopefully with a couple more weeks of physio that will get better.

**I: You’re all on the right path aren’t you.**

FP: Yes definitely.

**I: With some of those things you’ve described things like so now you are walking with a frame, so when you are practicing stepping and walking what do you think about?**

FP: Oh right, shoulders down, breathe because obviously I’ve had a really bad chest infection and my breathing was really bad but what I try and do is talk out loud to myself to remind myself what I’m doing. So if you hear me muttering as I’m walking along I’m just talking to myself. So yes when I stand up it’s like get my balance, feet apart, usually my shoulders are up round my neck so tell myself to drop my shoulders, breathe. That’s pretty much it really.

**I: OK then you go.**

FP: Go, yes. Repeating as I go. And also like when I get to my chair I’ll say to myself feel the back of my chair and then put my hands on the arms and drop down, don’t flop down, controlled sitting down in the chair.

**I: Are they things so things like dropping your shoulders and breathing are they things that the therapist have prompted you to do?**

FP: Yes, definitely. I know my posture because I used to do Thai Chi as well and my teacher was always coming and pushing my shoulders down so that’s why I like to talk out loud to myself because I’m listening to myself think and reminding myself to do it all the time, so yes.

**I: Do you find that’s a helpful strategy?**

FP: I do, yes.

**I: When you go from lying to sitting on the edge of the bed which you can do by yourself now what do you think about then?**

FP: Then I think about again getting my back, putting my feet apart, getting my balance, pushing myself up from the bed and then I’d go for my walker which is going to be close. Getting into bed again I’ve been getting into bed it would be lift my good leg up, make sure that my right hand is behind me for support and then use that lead thing to lift my other leg up into bed which makes me independent of having help to get in and out of bed. I can get out of bed without the lead thing. I just shuffle to the end of the bed.

**I: You think about the steps of the movement really.**

FP: Yes, shuffle to the end of the bed, push up from the bed, grab the walker.

**I: And you go. With your stepping and walking what do you think the next things are that you need to improve on? What do you feel that next?**

FP: I know that my good leg I’m tending not to push it back which makes my hips a little skewwhiff which makes my walk odd so what I’m trying to remember when I’m walking is to click the good leg back into place which does make my walk better when I get the rhythm of it because this one kind of clicks, the bad one clicks back into place. I know that they say like my hips aren’t aligned properly and that I’ve got to concentrate on getting that walk right lifting my legs, lifting the bad leg up and putting it down. Like I say that one kind of clicks back into place but that one isn’t for some reason and I know it’s my good leg but for some reason I don’t know why it doesn’t go. Yes that’s it really.

**I: So you focus a little bit on that as you walk do you do you think?**

FP: Yes. I’m trying to anyway.

**I: OK. From your therapy sessions how would you describe, sort of how do you feel when you are practicing all of these things, how do you feel in yourself?**

FP: It builds my confidence because they have been excellent, I think what they’ve achieved has just been amazing. I’m totally impressed. He’s over there. Don’t want his head to grow.

**I: He’s not listening.**

FP: I’m not sure about that. I don’t want his head to get too big do you know what I mean.

**I: Is there anything particularly about how they’ve given you instructions that you’ve noticed?**

FP: No they’ve been clear. Like I say they’ve been clear, they’ve been helpful and they’ve been confidence building. And I’m able to repeat the instructions to make myself remember what I’ve got to do. Definitely.

**I: And what about feedback, so as you’ve practiced different things and you’ve progressed have you received feedback from the therapists about what you are doing?**

FP: Yes.

**I: How would you describe that?**

FP: Well they’ve all said it’s been really good. Not that I want to blow my own trumpet or anything but it has been with their help but the nurse, everybody, said.

**I: Lots of positive feedback about how well you’re doing.**

FP: Yes.

**I: Do they give you specific feedback about actually how you are moving?**

FP: Yes because obviously especially with the way I’m walking they need to give me the feedback for me to be able to work on what I’ve got to improve which is obviously the way I walk. So I just repeat what they said really.

**I: Can you think of any examples of feedback that they’ve given you?**

FP: That I’ve definitely got to keep, I think the main thing with the walk is that I’ve got to keep this good leg clicking back into place because it tends to want to keep bending and that makes my hips not aligned. Honestly I can’t fault the feedback.

**I: So it’s always been clear, you’ve always known what they want you to do.**

FP: Yep.

**I: So you wouldn’t have preferred anything about that to be different or anything about the way they communicate?**

FP: No I think it’s been amazing.

**I: To be different. OK. Perfect. Then when you are not in therapy have you been practicing some things by yourself?**

FP: Yes.

**I: What sort of things have you practiced by yourself?**

FP: When I’m sitting in the chair I’ve tried to lift my leg up because I know that it’s working these muscles up here.

**I: Yes hip flexion.**

FP: So I try to do 10 at a time which is quite hard but I manage and then I stop.

**I: Did you set that target for yourself?**

FP: I did, yes. That one.

**I: OK so sliding your knee back.**

FP: Without a slide sheet so I can do it in my socks.

**I: Yes.**

FP: What else do I do? In the bed I bend my knees like I say, I do that one that I was doing trying to keep it controlled and do the bum lifts. I know they’re not called bum lifts but I call them bum lifts.

**I: Ah yes we call them bridging but I know exactly what you mean.**

FP: OK. I can lift myself up off the bed and hold for the count of five. Marching on the spot, I can’t march.

**I: Oh OK like in sitting?**

FP: Yes when I’m sitting in the chair but I do try and lift the leg up. I can’t do it because the table is here and hold it for the count of five which is quite strenuous as well. I did break out into a sweat.

**I: So you are doing quite a lot of different things.**

FP: Yes.

**I: And are they things you worked out for yourself to do or were they set by the therapy team?**

FP: No they’ve kind of been set by the therapy team.

**I: They’ve given you some guidance.**

FP: Yes definitely.

**I: When you are doing those so in your chair when you are practicing lifting your leg or especially some of the things that are a bit more strenuous what do you focus.**

FP: Marching.

**I: Marching. Are you focusing on anything in particular to help with that?**

FP: Mostly it’s about trying to lift my leg up like obviously I can’t march but it’s like trying to keep that, that’s the most difficult one.

**I: That’s the movement that’s a bit weaker.**

FP: Yes.

**I: That’s good.**

FP: Yes that’s about it really.

**I: That’s fine. So we’re nearly there really, so I think I’ve probably got a really good sense of this already but how do you feel about the progress you’ve made since you’ve been here?**

FP: I can’t believe the progress I’ve made. From at the beginning when I was just really worried that this leg would never recover. I pretty much knew straight away, I mean I got movement in my arm and my hands pretty much straight away and that’s just got stronger and stronger obviously the colouring sort of helps so that’s all good really. There’s just a little bit of a funny feeling in my thumb but that’s nothing. It’s a bit of a weird sensation. Yes like I know the leg is getting stronger every day. I mean you just can’t hurry these things really, I think the progress that I have made has been amazing.

**I: Two weeks you’ve had you’ve made amazing progress.**

FP: Yes.

**I: So last question which might seem like a bit of an odd one but if you were describing or telling if some friends came to visit you who hadn’t seen you since you’ve been in hospital and you were describing to them what you’ve been doing in therapy in a couple of words what words would you use to describe your therapy?**

FP: Oh my goodness. What do I always say I am, not an abstract thinker so it’s a little bit of a problem for me.

**I: Oh OK.**

FP: What words would I use to describe my therapy.

**I: The therapy sessions that you’ve had.**

FP: Beneficial.

**I: Yes.**

FP: Very beneficial. And confidence building, yes both of those things definitely.

**I: You use very positive words about it.**

FP: Yes definitely.

**I: Perfect.**

FP: I have nothing but positive words to say about my progress with the physios.

**I: You should be proud of yourself as well because you’ve worked hard.**

FP: Proud of the physios as well really. They’ve been amazing.

**I: OK. Anything else that you would like to tell me?**

FP: I think I’ve said it all.

**I: Thank you.**

**END**

| **INTERVIEW TRANSCRIPTION**  Voice file name: 1P Discharge Interview  Duration: 27 mins 49 secs  Typist comments regarding dictation:  **KEY:**  **I – Interviewer**  MP - Male Participant |
| --- |

**I: So what I’m going to do is ask you some questions about the rehab you’ve been having in hospital particularly thinking about your physiotherapy and about everything you’ve been doing to get back to being able to get from bed to chair to stand up and that kind of thing so mainly focused on your legs basically. There’s no right or wrong thing to say it’s just about hearing about your experience. As I say it’s all treated confidentially. So just to start us off can you remind me how long you’ve been in hospital actually, do you know roughly how long you’ve been here?**

MP: Probably about 12 days but I could be wrong on that.

**I: I think it must be a little bit longer than that.**

MP: My birthday was 24^th^ May and I was in here for that.

**I: Oh OK. So it’s now the 22^nd^ June so probably about a month or so that you’ve been in hospital.**

MP: Wow.

**I: Time all becomes a bit of a blur sometimes when you in hospital.**

MP: It certainly does.

**I: Can you remember when you first came in what the sorts of things that you were having difficulty with, what brought you in?**

MP: What brought me in was just to go right back initially I live in a block of flats, I’m on my own and one morning I experienced a back pain around here that I’d never had before. So I phoned my keyholder who also lives in the block of flats who are husband and wife and they said I’ll dial 911 for you. And then when the paramedics arrived they said oh you’ve had a stroke we’ll take you to the hospital. So that was the first that I knew that I’d had a stroke. Now whether I had the stroke that morning or the previous day I don’t really know.

**I: Not sure.**

MP: I did ask about from the doctor I think only yesterday and they said it could have been over a period.

**I: Did you have difficulty moving your arm and leg do you remember?**

MP: Well I did on that morning yes but up until then I hadn’t.

**I: No but the stroke had caused some weakness in your leg.**

MP: Yes.

**I: So what kinds of things have you been working towards in your physio when you’ve seen the physios here?**

MP: Sorry what kind of things?

**I: What kind of things have you been working towards in physio. What have your goals been?**

MP: I see people walking around all the time and walking frames and I think I’d like that for independence because at the moment I keep asking for a bottle and I use those RJ trolleys, the hoist system for transference and it’s tying up equipment and people’s time. I wish if I was a bit more independent I could do my own thing. I seem to know when I need the toilet so if I can get up and do it myself at the moment I just have a bottle.

**I: OK so being able to stand and walk are the things that you are working on.**

MP: We’ve had a few good days I’ve been transferred out to sit in the garden.

**I: Oh great.**

MP: And again I have to rely on people pushing me about all the time and I’m an independent person normally anyway so that’s what I’m striving to do.

**I: When you first came into hospital did you need a lot of help to be able to stand up?**

MP: Everything.

**I: Is it improving.**

MP: Yes surely.

**I: OK great.**

MP: In fact because the hoist is such a good strong, simple system and you are holding onto a piece of iron, bit of equipment it’s been reassuring.

**I: Yes the steady isn’t it you are using to stand.**

MP: That’s right. So I’m very pleased that I can use that bit of kit.

**I: Good.**

MP: Obviously sometimes it’s already in operation somewhere else so you can’t instantly have these things but I appreciate that.

**I: So when you’ve been seeing the physiotherapist what sorts of things have you worked on with them?**

MP: Everything from hand movements to dusting movements to standing up. In the gym there is a settee thing or a seat.

**I: The plinth.**

MP: In the front push the table like this but bigger and so I stand with my hands on that and push myself up but they are standing beside me obviously for reassurance. To stand on my own there wouldn’t be a chance of doing that.

**I: No, OK.**

MP: Then say we’ve done complete standing up and then we’ve done the hand movements and the arms. The arm is getting a lot better now and with assistance I can try and hold it up for the count of ten and that type of thing.

**I: Great.**

MP: The physio I’ve got to say they’re a great team and they’ve motivated me to do it.

**I: That’s good, that’s really good. As well as working on standing have you been doing any other specific exercises for your leg with the physios?**

MP: Yes I guess I have. Marching on the spot, extending your leg, holding it up for a count of ten and I’m practising this all the time like if you weren’t talking to me now I’d probably be doing it underneath the table. Things that you can do by your bedside I’ve been doing it and now I’ve been able to get my hand up and reach the rails on the sides of the bed here so I’ve been doing that this past two or three days and trying to twist grip movements. And also the hand spreading out, this hand obviously I can do that but I can’t manage to do it with the left hand so I’m keeping on at that.

**I: So that’s working on finger, we call it opposition for the tape.**

MP: I haven’t been able to clench it together and squeeze and a friend brought that stress ball to try and help me do that.

**I: OK good. So when you are doing things like working on your standing with the physios what do you think about when you are doing that, what are you focusing on?**

MP: Well they say look straight ahead of you like as if there was a target there. So they put a smiley face on the wall because I was deviating with balance a little bit.

**I: So it gave you something to line up towards.**

MP: Correct yes.

**I: Was that helpful?**

MP: Yes definitely. The one walking along the wall with the handrail attached to the wall is difficult because you are about there, you are about 2” from the wall so you haven’t really got a focus point because it’s too near. So I found that a little bit difficult walking sideways. Whereas when you are looking ahead you can centre yourself up. But the mirror I found a good thing. There is a cheval mirror, a full length mirror so I could actually see myself in it so then I could actually see whether I was deviating or not. So I think perhaps more mirrors would even be better and perhaps a mirror with a target in it so that you could see whether you were deviating.

**I: That’s interesting, so having something like a mirror gives you feedback of what you are doing and helped you to correct yourself?**

MP: Yes because my business was photography. I’m very much into visual images and you know so I think that would probably be quite a help would be to have a couple of mirrors and say if one had a target on it so that you could actually see when you are going off course. Because without the physios I wouldn’t have known I was leaning. They’d say oh you are leaning towards me or you are leaning away from me. I used to say that’s because you’re a babe magnet.

**I: Flattering them. OK. So for did it feel better to have something so you could correct that yourself rather than the physios saying to you oh you need to.**

MP: That would be a plus point. But I’ve got to say I’m very pleased with the physio I’ve received here from them all.

**I: Good.**

MP: I can’t fault it.

**I: Is there anything you’ve done with them that you’ve found particularly difficult?**

MP: Well probably just transferring my full weight onto my left foot. Unfortunately I’ve got bad varicose veins in my left foot going back hereditary so when I first came into hospital the bed at night I had the air pumps with the anti-DVT.

**I: Oh yes.**

MP: On our trips to Florida I’ve always worn flight socks so I know the importance of it but what happened was two nights of those compressing my left leg sent this leg crazy. They were obviously compressing the varicose veins up and I wanted to speak to the doctor and at that time every time I asked they weren’t on their rounds or I’d missed them or it was Saturday/Sunday. I was getting myself stressed out really on that one. Anyway to cut a long story short they’ve come up with the idea of not having the air pumps anymore but to give me injections in the stomach which I think is the blood thinners.

**I: Yes the Heparin. Yes exactly.**

MP: So I’m happy with that now.

**I: Once you are more mobile or not in hospital you won’t need that so it’s a short term thing as well. OK brilliant. Thinking about, so at the moment sometimes when you get out of bed and into the chair you are doing it a little bit with two people and not always with the steady, is that right?**

MP: I always try and use the steady if I can.

**I: Have you been practising with the physios doing it without though?**

MP: No.

**I: Oh not yet.**

MP: One person could be doing it with just the RJ but there’s a simplified version of that like a [*10 mins 35 secs*] red one which has got the knee pads at the front and I feel safe on that.

**I: OK.**

MP: It depends when it is. If it’s in the middle of the night and you’ve just woken up and you just want to go to the toilet I say can I use the bathroom and perhaps have a shower I feel confident with that. The transference if I was to get up now and go into the bed I wouldn’t feel quite secure unless there was somebody behind me.

**I: When you are standing up from a chair whether it’s using the steady or not is there anything in particular that you are thinking about or focusing on?**

MP: Just doing it.

**I: Just getting on and doing it.**

MP: Yes. These socks that I’ve got on.

**I: Oh yes to stop you slipping.**

MP: They’re great and they’ve got the grip pads underneath but probably for practising the walking and stuff I would be better eventually if you can get to be using trainers or something with ankle support because these are a little bit slippery.

**I: Yes so it’s a bit normal isn’t it to wear trainers.**

MP: So I think if I progressed to the next stage of using the walking stick with a spider bottom which is the next stage like I was using this morning down the corridor I did quite a few, ten full lengths of it. But I think I would have been better had I been in shoes. I brought slippers with me but I don’t want to wear them because the bottoms are a bit slippery.

**I: Sometimes slippers aren’t ideal but you are right trainers give the best because they’ve got a good grip and good support as well.**

MP: You’ve got to lift your foot up to actually traverse it rather than slide it.

**I: So you’ve been doing a good amount of walking then with the physios and we call it the quad stick the spidery stick.**

MP: I’ve been pushing it when I see them about I say could I have a session this afternoon or when you are free I’d like to have a go so I have been pushing myself.

**I: So how do you feel when you are walking, how does that feel for you at the moment?**

MP: I get tired by the end of it but like when we were going up and down they say do you want to do another one and I always say yes go for it. So I’ve sort of milked it.

**I: Is there anything to do with your walking that you are working on in particular?**

MP: You are obviously experienced in dealing with multiple of people so I’ve gone along with the sequence that’s been put ahead of me really.

**I: OK. By sequence do you mean the way you are progressed?**

MP: Yes because I said could I practice in a frame because some people were using frames but I haven’t yet gone in a frame. I suppose that’s the next stage really. But I found a little bit apprehensive is removing my hands shall we say from the handrail so then releasing and then looking back to see the chair handle and gripping onto that. So it’s that transition where you are in free air.

**I: Yes you’re not holding anything. So thinking about this morning when you were doing your practising with the quad stick and the walking were the therapists giving you any particular instructions about how to do it?**

MP: Possibly not specific no. I suppose you could say it would be pretty obvious but again I hadn’t seen a video of somebody using it good and bad type of thing.

**I: More in terms of your walking and your movements is there anything they’ve asked you to work on?**

MP: If I had to make a suggestion on that like you’ve just said did they show you it wouldn’t hurt to have some sort of like YouTube videos on an iPad that you could say look we’d like you to try this today, this is it being done well and this is it being done badly. Being into visual stuff or even a picture, just still pictures on it one with a big cross on it of how not to do it because it’s all to do with positioning your feet as well at the beginning before you start isn’t it have them underneath you a little bit so when you push down you are at the right balance point.

**I: So when you are going from sitting to standing having your feet in the right, yes, tucked back.**

MP: That’s probably what I find eventually when I get back to go home it’s a balance situation because I have noticed for example when I’m getting dressed in the morning at home when I’m on my own I lift a foot to put my socks on or something like that I’ll sit on the edge of the bed to put my socks on rather than try and balance because I can feel I’m deviating a bit. Your home can be an unsafe environment can’t it.

**I: Yes.**

MP: So I’ve got to think ahead when I’m at home.

**I: Yes find ways to make it safer.**

MP: Correct, yes.

**I: Absolutely. With your actual stepping and the way in which you are walking is there anything specific that you are working on or is it just practice?**

MP: No again it’s just practice. When that first started about doing the steps I said shall I imagine that I’m pushing my heels into sand shall we say and they said that sort of works.

**I: OK. You worked that out yourself did you?**

MP: Yes.

**I: That’s not what was said.**

MP: I mean obviously you go for a fluid movement if possible don’t you not jerkiness but if you see somebody walking who has got a false leg or something it’s always very jerky of course. I try to keep it fluid.

**I: That’s great. Any ways in which the therapists have given you feedback that’s been helpful or not helpful?**

MP: Oh yes they are always encouraging and complimentary on what you do which is good. You want to hear the good stuff not failure sort of thing so I think they are probably over complimentary on me at the moment I don’t know.

**I: I’m sure that’s not true. They are an encouraging bunch though.**

MP: Well I think you’ve got to do that.

**I: That’s important to you.**

MP: That’s part of your job isn’t it. It’s not just doing certain things it’s having a personality to go with it. I’ve found everybody here very helpful. And our block of flats we all clap for all the NHS on a Friday night, did our bit, on the balcony. People used to drive by and toot their horns. I think it’s been long awaited the recognition of what the NHS does. But everybody you speak to thinks they’re doing a great job but then at the same time probably you’re not probably paid anything like what you are worth.

**I: Well we choose to do the jobs that we do don’t we.**

MP: I think it’s turning the point now where recognition is being done on a much bigger scale than what it ever used to be.

**I: Which is important. Have you seen different therapists whilst you’ve been here. Have you met a few different physios? Yes. Have they all been similar or are they different in how they.**

MP: Personally but that’s another slight thing there are so many people involved and I think bigger name tags would be a great thing.

**I: OK.**

MP: Some of the nurses have got some that you can hardly read because they’re behind a transparent pinny thing. A strong name tag is important when you are speaking to somebody.

**I: You know who.**

MP: You can call them by their name rather than not. I always do try to call people by their name and get a rapport going. But even the name on the boards out the, the board on the hospital wall there the clocks aren’t lit at night so when you wake up you don’t know what time it is. I’ve mentioned this before, not to you but someone, a light onto the clock would be a good idea especially if you wake up.

**I: OK so yes at a glance you could see.**

MP: Perhaps a bed is being transferred next to you and you think wow what on earth time it is. You hear people saying what’s the time, it’s 2.30am or it could be 6.30am or whatever when it’s dark outside you don’t know. There’s a big symbol of a sun and to be honest you don’t need that but what would be better would be a bigger name plaque for the nurses names especially if they are foreign names because what seems to be prominent is state nurse or whatever it’s called.

**I: Yes staff nurse.**

MP: So they put more prominence on your status that what your name is.

**I: OK that’s definitely something we can look at.**

MP: And to do it because some days it’s Thursday and they’ve still got Wednesday or something.

**I: Well that’s right either the board needs to go or needs to be up to date doesn’t it. I hear what you are saying.**

MP: Otherwise it’s useless isn’t it. That’s only a side comment drifting away from what you are asking me.

**I: That’s alright. It’s useful to hear what you think.**

MP: Bigger name tags that the patient can read and not so much worry on the status of it. I thought perhaps it was senora.

**I: Not senora staff nurse.**

MP: Or what’s the other one that’s the contract? There’s other initials isn’t there if you are a care assistant isn’t it or something?

**I: HCA often, healthcare assistant.**

MP: Technically Ann Smith is all you need isn’t it.

**I: Or just Dan, yes.**

MP: We don’t need to know what their status is.

**I: You see a lot of different people don’t you. So with the different physios.**

MP: Oh yes the day and night crew. I often say because if you’ve been asleep and then suddenly you wake up and it seems a few hours have passed you’ve got a different crew sorting you out and I usually say are you night crew and they say yes we’ve just come on or no we’re going off. So again from a patient’s point of view it’s nice to know who is actually on duty so that board is important.

**I: Yes I will look into that.**

MP: So that’s really feedback on something else whilst I’m speaking with you.

**I: No that’s useful to hear. It’s the little things isn’t it that can make a difference and easy for us to solve as well. When you’ve seen different physios in your physio sessions have you felt that they’ve been quite similar in their approach and what they’ve been doing?**

MP: Yes they are and as I say they’re all very encouraging to do it. But I can understand if you were not so motivated and a bit lazy or you’ve had a bad day you’d probably decline. If they say do you want to do some physio this afternoon I’ve always taken it up and said yes I’ll do it.

**I: Good for you, that’s what helps you to get better.**

MP: Well that’s right.

**I: Has it always been clear in your physio what they’ve wanted you to do, what they’ve been asking you to do?**

MP: Yes I’ve got that purple folder and that’s been very helpful.

**I: Good. Just here next to you.**

MP: Yes that’s right, that’s it.

**I: So you’ve got some bits in there as well.**

MP: That’s all so helpful when visitors come round to see me I say if you open up that purple folder you’ll see the reason for the stroke, well not the reason for it but as you’ve got it this is what life is like at the moment for you and they all thought that was very helpful.

**I: Yes it’s good to have that extra bit of information.**

MP: So that is good not only for the patient but for people who are around you to know what you are going through.

**I: Have they given you some exercises that you keep in that folder as well or do you have things?**

MP: There are ones there. Obviously I’ve tailored it to the position at the time. I’ve got some room underneath me I’ll do those. The marching on the spot there’s never enough room for there to be able to do that unless you push the table away so I haven’t done that. But I’ve done the lifts and try and hold for 10 seconds and down.

**I: Good so you are doing those things by yourself between.**

MP: Yes definitely plus the dusting action there to try and go left and right they said as well to do that.

**I: That’s good. Do you have a way of knowing if you are improving or not? Do you monitor your progress with the exercises?**

MP: No I haven’t done that. I’ve got this book going which I ask everybody if possible to sign it and I’ll ask you if you could put your name on it. It’s not a diary, they are all out of order, but people I say oh could you put your name on that and just sign what you’ve done for me so perhaps you could do that.

**I: Yes I can do that. On that page there?**

MP: Yes that will do fine. And just put the date and time.

**I: So what did we say, it’s the 22^nd^ June.**

MP: Tuesday.

**I: And it’s, what is the time actually, it’s about.**

MP: 2.30.

**I: 2.30.**

MP: It’s funny I’m asking you to fill in something I’ve just done yours.

**I: I know and then I’m asking you. I’ll put Tuesday. So my name is Louise.**

MP: That’s funny there’s two of you, two Louise’s.

**I: What we’ve done is.**

MP: Talked through a questionnaire or whatever you want to put.

**I: I’ll put as part of an interview.**

MP: Lovely thank you.

**I: For a research study.**  **Great.** **There we go.**

MP: That’s it, that’s added to my book well done thank you.

**I: I’m in the book. I think probably I’ve asked you most things. I’m just going to check through my list of questions. We’ve talked a bit about how you feel about your progress.**

MP: I’m positive on that.

**I: Yes good. If you had to describe your therapy sessions, your physio sessions how would you describe them to family or somebody else who hasn’t been here.**

MP: Definitely as good as they can get in the circumstances. You are working in a corridor and occasionally beds come by. But when I’m in the gym that’s quite nice because you can actually see out but you can also concentrate. It’s very much a concentration thing as you know. Your mind is flitting from one thing to another and I’m a Gemini which doesn’t help on that one. I go from one thing to another. They physios sometimes say concentrate and it draws me back again and that’s when we put a target of a smiley face on the curtains. When they said look straight ahead and concentrate on something I said.

**I: That gave you a bit of a focus.**

MP: That smiley face so they did it. Hopefully it’s a two-way thing, I give them some info back which is what you are basically asking me now.

**I: Exactly. Lovely. Is there anything else that you want to tell me about your therapy that we’ve not talked about?**

MP: No I’m appreciative of what’s been done for me and everything else that the NHS has been doing. I’m very pleased with it. I wouldn’t want to go through it again but.

**I: No we wouldn’t want you to either but if you are going to be somewhere this is right place to be after a stroke.**

MP: That’s right.

**I: Obviously we would all much rather nobody had strokes.**

MP: My daughter is in Florida and she speaks with the doctor and they’ve got a thing going with information going backwards and forwards.

**I: He’s keeping her updated.**

MP: So he’s keeping up to date.

**I: Lovely. I’m going to turn this off now because I think that’s fine.**

**END**

| **INTERVIEW TRANSCRIPTION**  Voice file name: 3D Discharge Interview  Duration: 41 mins 26 secs  Typist comments regarding dictation:  **KEY:**  **I – Interviewer**  MP – Male Participant |
| --- |

**I: So what we’d like to chat about is the rehab you’ve been having whilst you’ve been here particular the physiotherapy. So you’ve seen Amelia haven’t you, Steve and some of the other team.**

MP: Yes Steve, Ashley.

**I: You know them all.**

MP: Yes, yes but they’ve been by my side all the time.

**I: So we want to talk about what they’ve been working on with you particularly around your recovery for your legs, so sitting, standing, stepping because obviously you would have been doing some physiotherapy for your arm as well.**

MP: Oh yes, yes.

**I: And we can talk about all of that but we’re particularly focusing on the leg end of things. So just to start us off can you just tell me, I know you’ve been here for quite a long time but tell me about what you’ve been doing in your therapy sessions. What sorts of things have you been doing?**

MP: The first thing I was taught to do was my leg. Why I say my leg it’s because it was a Saturday afternoon came back from shopping and I had my phone in my left hand, I won’t bore you, I had my phone in my left hand and I kept dropping it. I was putting it back in my hand again and I dropped it. I said to my wife what the bloody hell is going on, why do I keep dropping this phone for. So anyway I picked it up and dropped it again. So this time I just put it on the dashboard of the car and got in and then when I started changing gear I could feel that this was like a rock, this hand, so heavy and that was that. I got home, brought the shopping in, she put it away, I sat down and I got up to put the kettle on and I sat back down, no that’s not right. I sat back down after putting the shopping away, she put the shopping away, sat down, watching the cricket on the TV and I got up and just fell like that.

**I: Gosh.**

MP: Then I fell on the floor, tried to get up and I couldn’t get up. I thought come on get up here, I was swearing at myself. I’ve fallen over before, not through that but messing about with grandkids and things like that and tossing them around. This time I couldn’t get up. I said to my wife I’ll be alright later I said it will go away. So anyway I just laid there and I stayed there until Sunday.

**I: Crikey.**

MP: I went to bed. I crawled upstairs and went to bed but I was aching and I wanted to turn to get that aching out of the way to put so I could rest this shoulder.

**I: Take the pressure off, yes.**

MP: And as it was I just couldn’t it was still hurting. So it was hurting me Sunday morning and I said to my wife I said I’ve had enough of this. I said something is not right here and then I found I couldn’t move this hand.

**I: Was your leg affected as well?**

MP: Was my what?

**I: Your leg affected as well?**

MP: Well it had to be yes, had to be. It’s something that I could remember when people asked me this before and then it drifts away the memory, it drifts away.

**I: It’s a bit foggy that time.**

MP: Yes. It was just the, it was something I was trying to think of and think of and I’d go through the motions, had I missed anything, am I over reacting but I wasn’t over reacting because I couldn’t move the arm, the leg was playing up not painfully or anything like that just couldn’t move it.

**I: No just not right, something wasn’t right.**

MP: It was all hard, it felt like I could hit it with a sledgehammer and it wouldn’t hurt me. So anyway I said that’s enough I said I’ll ring the ambulance. Why I didn’t ring [name] because I was embarrassed because we live in an area where people nose the windows, you know.

**I: Yes.**

MP: I said I don’t want them nosing into my business, oh what’s up with him. Two and two gets put together and then they get the wrong conclusion. So anyway I said that’s why I didn’t go Saturday and then Sunday I said that’s enough.

**I: You needed to. So you came here to Winchester.**

MP: Yes this is the only place I think and I came here.

**I: Did you start having physiotherapy quite soon after coming here?**

MP: I was laid up for a little while.

**I: Were you.**

MP: There was a guy next door to me I think I should mention it to you he’s got the same symptoms, he’s got what I’ve got. I said you’ll be sorted out, I said you’ll be getting up because I couldn’t walk. I couldn’t walk. That was annoying, I couldn’t go to the toilet, couldn’t do the things I used to do. Couldn’t even put a shirt on. But the physios, I was in one ward they called it heart ward when you’ve had a heart attacks or you have strokes, I was there, I was looked after there superbly. Excellent, can’t describe it. It’s when people come to you and help you, it’s like the nurses and even the people that bring the food to you, even the cleaners. We’ve got a cleaner here, Ken, fantastic man.

**I: It all makes a difference doesn’t it.**

MP: Doesn’t it. It makes you think Christ this is all inclusive. This is all inclusive, I’ve got everything here.

**I: So can I get you to think about so when you’ve had physiotherapy in those early days you said you couldn’t walk, your leg was playing up, so when you were having physio what kinds of things were they doing with you in physio? Do you remember?**

MP: Yes. I came to physio and it was Sarah and Kirsty they looked after me like a father, which was nice. Being a little way from home, I live up in [name] not getting many people come to see you it’s a lonely, lonely place. When everyone has got visitors around their bed I didn’t. But then my nephew came and he sorted everything out for me. He was coming down and brought my wife down and that made a lot of difference. It bucked me up, you know.

**I: It’s important isn’t it to see people.**

MP: It’s important. Always remember when you’ve got family look after them, forget about stupid little squeaky arguments, forget about them but it’s family and you stick together even if you are right or wrong you still stick together. That was a buck up as I said.

**I: So who did you say were the physios, Sarah and?**

MP: Sarah and Kirsty were my first two girls.

**I: In the beginning. And what were they doing with you, how did they help you?**

MP: They were helping me with my arm, with my fingers, my wrist. I couldn’t even move this, this was all.

**I: Brilliant.**

MP: Yes I know, I know. This is why it’s so fantastic that people care so much, you’re not just a person you are a person, you’re not just somebody with an illness and you come in but you are special to them.

**I: They are working with you.**

MP: Yes and for those people they want you to get better. They don’t want you sat there moping or whatever they want you better and out so they can see you as you were in your proper form.

**I: Absolutely.**

MP: What was nice was that was all sorted and then things started, little improvements.

**I: So were they doing exercises with you for your leg?**

MP: Oh all the time, all the time.

**I: What kinds of things were you practicing?**

MP: I was like getting my leg up in the arm, I couldn’t do that. I couldn’t even get it up there.

**I: OK so getting your leg moving.**

MP: Yes getting that all moving. Then I’d go back to, because down there it’s hard work because I don’t give in see, I’m not a giving in-er. I just keep going.

**I: Push it.**

MP: Oh yes big time. Then I come back, they wheel me back to the pit as I call it and I might have half an hour sleep because it’s so tiring. Probably because of what I’d gone through and that.

**I: Yes you’ve worked hard.**

MP: And then if I wake up in the night I try and do them exercises. I do them in the bed.

**I: Yes, good.**

MP: There’s still bits and pieces I’m still working on. That was the start and then the other start was, the second part would be balancing.

**I: OK.**

MP: That was the important thing, I couldn’t balance.

**I: So once you could stand but needing to work on your balance.**

MP: Once I could stand, yes, once I could stand I could balance. It’s like down there now I could balance for 10-15 minutes. I can now put something down like Amelia did the other day, put a pen down on the ground, can you pick it up and I picked it up.

**I: That’s really.**

MP: Because I knew I’m going to pick it up.

**I: Tricky thing to do.**

MP: But they were there with the guidance all the time, that was the important thing.

**I: So what sort of guidance did they give you?**

MP: Making sure that they had hold of me so if anything had happened I wasn’t going to hit this hard floor.

**I: So made you feel safe.**

MP: Oh yes because when I started doing stuff like rolling, I was on a cage and I had to roll and they’d be doing different things to me, bring my arm back, bring my leg up. That was still going on.

**I: So they were helping you do that?**

MP: Yes.

**I: Is that what you mean they’d be lifting.**

MP: And it was that with that because it was still ongoing because the physio for the arm, the leg was still ongoing because it’s not going to happen overnight. So that was that to that point and then when I started rolling, rolling, rolling to help them to move my frame I used to keep seeing this floor that was going in my head because it was not a floor that I’d fell on that was on the carpet but it was a fireplace and I used to shy away a bit because I thought oh no how secure are they because they’re fit people and they’ve got muscles those girls I tell you, they’ve got muscles.

**I: They’re stronger than they look.**

MP: Oh yes big time. They knew a little bit of a phobia that I was going through and they dealt with that by talking to me.

**I: OK.**

MP: In the end I thought you silly bugger why are you fretting about, you’ve got three pretty girls holding you and there’s no way you’re going to fall. That’s how I put it. And that’s how I got over that phobia. But sometimes I do get easily distracted and they say oh you do get distracted. I said yes hang on a minute I said yes I do I said but please just bear with me a minute on this sentence. I said those girls that say cor aren’t you doing well to me and they were the ones, first ones, like the servers anything to do with that ward were there for me. For me. They could have done another occupation but they didn’t they wanted to care for people that is the difference. And that caring was great and I always remember certain faces, how nice they were to me. None of this not brutal but none of this oh hurry up or something like that.

**I: Yes so people being kind to patients.**

MP: If somebody had had a bad day or bad night with the husband or whatever and they bring it to work that wasn’t there. It was just that when I’m talking down the corridor myself just with an aid sometimes, sometimes I just clatter off on my own, probably shouldn’t do really but when you feel confident.

**I: Well that’s part of your recovery.**

MP: There’s always a wall there I can touch. If you are going to fall just use your hands, that’s what they’re for. Sorry just last bit.

**I: Yes go on.**

MP: As I’ve been walking through the corridor and everybody says cor you are doing well and I say, thanks ever so much. I just like that. And they call up and I’m concentrating but I am not the person that’s just going to walk by somebody like that, that’s helped me because you always remember nice faces, and just ignore them. It doesn’t work like that.

**I: No and that’s sort of automatic as well isn’t it.**

MP: Of course it is.

**I: That’s part of you.**

MP: That was something that obviously went against me in some stage or another but I’d still do it now. I’ve got names written down that have been nice to me because they wave to me, it doesn’t cost nothing to be nice. But it does cost something to be horrible because that plays back on you.

**I: Yes that’s true.**

MP: Go on, your turn.

**I: So you talked about at the beginning you were practicing the things like rolling and the therapists were helping you with that and then practicing standing so getting from sitting up to standing.**

MP: Yes, yes.

**I: And when you were doing those sorts of things what do you focus on?**

MP: I just focus on getting it right. They’ve told me what to do and I don’t want to be there like a thick head thinking what’s he just said something like that.

**I: So what sorts of things would they tell you to do? Can you recall?**

MP: Oh it would be walking along bars hand like that, like that, like that, still with guidance just in case anything happens, faint or something maybe. But they were still around me tight as can be, so I felt so safe, so secure, so secure that I knew nothing was going to happen to me so I just went it like a bull [*16 mins 33 secs*].

**I: So you could give it a go.**

MP: Some of the stuff on the walking because that comes a little bit later the walking.

**I: Yes.**

MP: Was that I’d say to them this isn’t my pace. I’m messing it up I’m walking too slow and I’m not a slow walker. OK. So they just listened to what I had to say and do your own pace and I still had tight guidance around me just in case my pace was too quick if you like and I start tripping over one of my feet or arse over head but I knew the safety was there and I found it better then walking at my steady pace without going slow. When I was going slow I was just tripping over myself, I couldn’t get control of it. That was another good part because I had a little, I mean they’re trained I just do what they say. They’re trained, that is their profession and when they’re treating you like, because you see the determine, you can have the one to one if you like and when you see that determination they say oh well done, well done. You don’t want to let that guy or that girl down. You just want to please them.

**I: Yes so you are working hard for them as well as for yourself.**

MP: Of course you are, you are because they’re giving up, I suppose they’re like little magicians where they’re wanting you to do it and they’ve got the determination because you can hear them saying oh God look at that, well done and they’re saying that like through one or two or three of them. I go in there and I got, I put it like I see young people in there and I see this coloured lady and she had a stroke and her face was, pretty girl, and she was doing this tread thing, treadmill thing, and I said go for it, go for it, don’t get nothing in your way just go for it. I was thinking me in her, you know what I mean?

**I: Yes absolutely.**

MP: And she was really, I said there’s a record up there you’ve got to beat. Oh where’s that. I said what are you looking at. And on the side of this record that she had to beat, that was how many revolutions things, she said no I’m not looking at that she said I’m looking at the cakes on the side. It was so funny, there were cakes on the right-hand side of the picture. I said oh just determination. Oh thank you so much she, just keep going, grit your teeth and keep going. That’s what I done.

**I:**  **You said that the therapists were really good at, it was really nice when they gave you praise when they said oh well done.**

MP: Yes, yes they were with you all the time. There wasn’t a moment where you were left, I’ve got to go off somewhere or.

**I: Did they give you specific feedback about what you were doing?**

MP: Amelia used to every fortnight she would give me some tests to do, not written tests but doing stuff like getting up.

**I: Ah yes so they’re part of the research.**

MP: I wasn’t getting up off the chair properly. I’ll just show you. This is something that learnt me because I just fall arse over everywhere and it was your feet have to be like that. This is all starting couldn’t walk, this is all the starting process of walking.

**I: Yes.**

MP: Like yourself you’d be sat there like that and then you’d get up and get a book off that shelf. You do it automatically don’t you.

**I: Yes you do, you’re right.**

MP: And I tried to do it automatically like I used to but it wasn’t working. It wasn’t working.

**I: What were you doing wrong do you know?**

MP: Yes because I wasn’t having my feet properly positioned. That’s how you have to have them just like that, just a gap like that. That’s what they learnt me to do and just bring them back a bit like that then you know roughly that that foot there is in position with that shoulder and that one there is in position with that shoulder. And what you do you put your hand there so that is sort of parallel with the leg to the knee down to that shoe or that foot and then put your hands there. I couldn’t even do this then and it was to grip round here.

**I: Round the chair.**

MP: I couldn’t even open this, I couldn’t even open it.

**I: Your arm is doing really well.**

MP: Like that there, your feet there and get up like that.

**I: OK.**

MP: And stand like that and focus. I’m focusing to that.

**I: To one of those pictures, yes.**

MP: Yes, one of those things in the middle and I’m looking at it and looking at it and standing up straight. You have to stand up straight. Then to sit down, say I wanted to go to that chair there, say there was a chair there, they would teach me to go to that chair. I’m not going to sit down.

**I: Don’t sit down there’s no chair. I’m just wondering if I should leap up!**

MP: I’d be there and there would be a chair already for me there keeping me safe.

**I: So if you come back and do it here you can show me what they taught you.**

MP: Yes that’s what I’m just going to do.

**I: OK.**

MP: So if I turn like that, I come round like that, they want me to position myself so I sit back in that chair. So how I do that is that like that, that like that, that like that, like that, hands back on the rail, they’re still guiding me I’ve still got the chair there so I don’t go back and I sit like that.

**I: Well done.**

MP: That was the start of me being able to sit down again and getting up.

**I: So knowing all those bits.**

MP: And that was lovely.

**I: Was that similar for when you came to doing walking as well when you started to take steps and walk?**

MP: Yes when I started taking walking that was a little bit of a long process. A lot of that was again, I’ll just do it quickly. Not too quickly I might fall over it.

**I: No, no take your time, I don’t want you falling in my interview.**

MP: No, no not in this room, no. Up like that again and just stand up tall. Focus. Stand up just like I’m doing now with my legs.

**I: So nice and straight.**

MP: Yes straight, keep your leg straight. That was drummed into me all the time. Keep your leg straight, don’t bend your legs, keep them straight and I used to keep saying that to myself, straight, keep your legs straight, keep your legs straight. Stand up straight.

**I: And this is as you are finding your balance?**

MP: Yes, yes.

**I: Yes.**

MP: Before the walking stages and I’d be looking and looking. Until I got fed up of looking then I’d say to myself right I’ve looked enough that’s it. Then I would so that same movement what I done just now by sitting down but I just do it like that because I got confidence in doing that. I can do that now without having to do that and that and then sit down.

**I: So it becomes a bit more automatic.**

MP: Yes that’s right it comes a bit clearer.

**I: So when you did start walking because you just said then oh I would say to myself all the time keep straight. When you were walking was that similar, were you thinking about what you needed to do?**

MP: They gave me a line.

**I: OK a line on the floor.**

MP: If you say that line there which is running from that door, middle of that door up through here it’s really like just through the corridor there but that line there, say I was running this way I’d be told to keep to that line. Keep to that line. Not waver, not waver. Don’t keep to the left, don’t keep to the right.

**I: Did you find that helpful to have a line?**

MP: Oh yes and I still had guidance there as well. Ashley and I can’t remember one of the physio guys he’d be to the right and Reece would be to the left and they would just be to the side of me just in case I went to the left and tripped or somebody came out with the trolley because it’s quite busy there and that’s the only run that we had.

**I: That was long.**

MP: Yes for me to get going on. You felt safe, safe, safe. And if you did drift you had a hand there and that would give you confidence so you didn’t mind if you drifted off that you still had the person there left and right and they give you confidence.

**I: Did you find any other techniques that helped with your performance with your walking in particular?**

MP: Yes that was again a lot of balancing.

**I: That was your tricky thing was it the balance?**

MP: The balance to start with because I hadn’t been on my legs, I hadn’t been on my legs for ages. They’d put these like circulation if you are not doing anything.

**I: Oh yes the pumpy things.**

MP: Pumpy things, yes. And then when I went to the physio I used to take them down with me to physio because I didn’t know no different then they took them off for the sessions and then put them back on again. But after a month I didn’t need them because all my blood was circulating. That was another little thing, not a big thing but it’s another thing towards your recovery.

**I: It’s all progress isn’t it.**

MP: But that had gone and sometimes you think why have I got to wear that, is that going to be on for life but it was only for a month whilst I could get the feeling and get the blood circulation round into my legs. If you don’t your legs can drop off. But yes that’s the most important things.

**I: And so thinking about your therapists, so you’ve talked a lot about your therapists how they made you feel very safe and how they were very positive so they were giving you lots of praise and things and encouragement. Is there anything else about how they talked to you when you were doing exercises, so how they gave you instructions or how they gave you feedback – is there anything about that that worked well?**

MP: If you did something and you didn’t do it quite correctly you’d have another go at it. So it wasn’t oh you’ve done that or that’s a failure kind of thing they say, well I said to myself that’s another bugger up I’ve done today, something like that, and I’ll have another go at it until I’ve got it right. Sometimes you got it right sometimes you get it wrong but most times [*29 mins 01 secs*] and get that right, don’t let that beat me, bloody hell I’m 70 years old.

**I: Would they tell you what you need to do to get it right or would they just let you work that out and practice yourself?**

MP: No they tell me, they tell me.

**I: OK.**

MP: Yes they tell me. Don’t put that hand so close to that one. Keep them at a distance apart. That’s where that’s just gone wrong they’d say but we’ll get that right and I just follow what they say. Then we had all pleasures then and that’s good.

**I: OK.**

MP: Yes you didn’t just forget about that stretch of it. They want you to do well.

**I: Oh I know they do, yes.**

MP: They want you to do well. They get disillusioned if you, they don’t want cry-babies.

**I: No they want you to work hard.**

MP: Cry babies sat there going, oh that hurts. Oh get up you great big twerp, you know.

**I: Did you use the Zimmer frame or anything at any point or a stick?**

MP: No.

**I: No you didn’t need to have anything between.**

MP: No.

**I: You jumped that stage.**

MP: Yes, don’t want one of those bloody things.

**I: So when you are walking now because you said you do a bit of walking by yourself.**

MP: Yes, yes.

**I: Do you think about anything whilst you are walking?**

MP: I just set myself items that I’ve got to go to.

**I: OK so aiming in the distance.**

MP: Yes. If I’m up there by the reception I focus on the coffee machine that’s always parked here. Sometimes you get people come through the other door and you have to just bow to them because two can’t go by. You do get some ignorant people that just don’t care, think you’re a cripple. I’m not a fucking cripple mate, I can walk better than you.

**I: So you don’t think too much about the actual walking you just go for it.**

MP: Yes I just put my head down. I think if you worry too much about it, which I used to do about other things, you ain’t going to get nowhere because you are so involved in your – yes as I say you’ve got your head up your arse and you don’t want that, you want to be able to focus on what you’ve got in front of you and it’s impossible.

**I: And what you are trying to achieve.**

MP: Yes. The only thing I do have a little bit of trouble with it my clothes sometimes getting a shirt on.

**I: Because of your arm weakness.**

MP: I don’t know what it is really. At home I just sit on the bed and I dress myself, here the beds are so high.

**I: Yes it’s all a bit different, isn’t it.**

MP: Yes it is different. This is one thing where they want to take me back home to see how I get on. But as my wife said to them yesterday she said he’s been ill before, not seriously ill, but like the flu and all that and I looked after him. Couldn’t understand that. When I explained it to her I said it isn’t just that, I said it’s for when I get home because I don’t really want to put too much pressure on her.

**I: No you need to make sure the support is right don’t you.**

MP: Yes because she’s got support.

**I: Yes exactly.**

MP: Trying to deal with the same company. No I mean in a nutshell do you know you know how you sometimes you can let yourself go, there was one day there where they were so kind and gentle it was the words, you know, confidence of the words they were saying to me. I burst out in bloody tears.

**I: Did you.**

MP: Honestly. I was so appreciative.

**I: I can hear that in what you are saying how much you value what has been done for you here.**

MP: Even down to anybody that works in here, everyone is so friendly.

**I: You don’t get that in a lot of places, that’s something quite special I think.**

MP: No you wouldn’t get it in some countries. You wouldn’t get your bed changed every day.

**I: No you wouldn’t.**

MP: You wouldn’t get this food you get every day.

**I: No. You’re going to miss it a bit aren’t you.**

MP: The all-inclusive without a swimming pool.

**I: Without a swimming pool, yes. So just going back to your exercises and your legs, so just the last little bit I suppose on the way the therapists were talking to you so they are very encouraging, they would tell you how to do it differently if you weren’t doing it right and you found that helpful?**

MP: Oh yes.

**I: Was it always clear what they wanted you to do?**

MP: Oh yes.

**I: Did it make sense?**

MP: Yes, yes.

**I: OK. Then you mentioned that you do some exercises outside of therapy so by yourself you do some stuff.**

MP: Yes just like in bed if I’ve got to do some footwork or just improve.

**I: Yes so you do some leg movements. How do you find that?**

MP: Yes.

**I: Getting easier I guess.**

MP: Yes it’s all a little bit helpful. It’s all very helpful because it’s like when you cut your finger you don’t expect it to heal up straight away it’s going to take a couple or three days maybe but it’s like physio, going for physio and you can be doing some higher physio, I’ve had some higher physio last week and that was going up the fire escape stairs. Have you seen them?

**I: I haven’t but I can imagine what they’re like, fire escape ones are normally quite steep aren’t they.**

MP: They were. I was going up I think it was three flights.

**I: Were you? Gosh.**

MP: Three flights.

**I: How did you find that?**

MP: Hard but I did it.

**I: Physically hard or?**

MP: Yes, oh yes. Everything in the back of your legs is aching which is good.

**I: Yes it means you’ve worked hard.**

MP: Because you know everything is clicking in. It’s like if you break your arm or something you’ve got to wait for all those little fragments all to stitch together. And I had to come down backwards.

**I: So when you were going up and down the stairs did you feel you had to concentrate quite a lot?**

MP: Oh yes you’ve got to concentrate.

**I: What were you concentrating on?**

MP: Holding the handrail but I still had people beside me which is very, very important and they make sure that they’ve got somebody there, somebody that can hold my weight if you like because when the weight falls you know yourself it takes a bit of grasping. But that’s a build up of confidence again that makes you do what you’ve got to do because you’re not frightened. You wouldn’t be frightened of falling even because you’ve got people there.

**I: No because you know they’re there. You trust.**

MP: There are some big guys there that will just snap you up. You ain’t falling on my patch mate. Not on those stairs.

**I: They don’t want that to happen any more than you.**

MP: They don’t want that to happen too much paperwork.

**I: No exactly.**

MP: So that was an achievement.

**I: With the exercises you’ve been doing by yourself are they ones the physios have given you or are they ones that you’ve just worked out yourself?**

MP: No this is all part of, oh what you mean the stairs?

**I: Sorry I’ve gone backwards, you were doing some bed exercises and moving your legs.**

MP: Oh yes it’s something you can do yourself.

**I: Have you worked those out yourself or were you told to do them?**

MP: I have been doing them exercises in the gym.

**I: Ah I see and then you were practicing them.**

MP: Yes and it was laying down exercises on the bed which you can do on the bed in your pit. Yes it was all part of the recovery.

**I: Good. I think we’ve probably talked about this quite a bit but how do you feel about the progress you’ve made since you’ve been here on the stroke unit? So if you think back to the beginning that you’ve told me about and where you are now, how do you feel about that?**

MP: There’s nothing really to talk about on that just excellent all the way through. To me it’s a team effort and that team effort has just worked, it worked for me.

**I: And you can see the progress.**

MP: Everybody knew what they were doing. Yes, just as soon as you come through the door they check the sheet to see who they are seeing and go up and get them, it’s all on wheelchairs or whatever they needs they need and they come down to the gym and then whoever is in charge of that particular patient has him or her for that period of time that they’re allocated. You just go through the motions. If you don’t want to get better then you don’t want to get better but if you do just listen to them and even if you are the biggest know all just listen to them and if you don’t want to do it just go back to your room and can’t be bothering. If you want to stay like a clown if you like and you don’t want to get better for your grandchildren or your kids at home well then you are just a defeatist.

**I: You are very determined I can hear that aren’t you.**

MP: Oh yes, it’s in the family.

**I: OK so just the very last question is so if you were talking to your family or somebody about your therapy sessions, your physiotherapy particularly, how would you describe it, what words would you use?**

MP: There’s only one isn’t there, there’s only one.

**I: What’s that?**

MP: Excellent.

**I: OK. Good. I think that’s all of my questions unless you have anything else you want to add?**

MP: Yes, on one occasion they brought a wheelchair up and they left it by my bed and I said to Reece I said are you taking this wheelchair away and he said no he said I’m going to leave that here because when your family come it’s just an idea he said you can sit in that and come down to the gym, push you down to the gym and you can show them what progress you’ve made.

**I: Ah.**

MP: Sometimes I’d be down in the gym when they visited because they’re so flexible here it’s so good. People that are working and that can get down here early afternoon and I said no I said that’s alright. But then we’re talking and I don’t know if I should just because they know what I was like but no I don’t think I’ll bother with that, I won’t show them, no. They can just seem me as I am. They’ve seen me before and they can see me now progressing to this point. Biggest aim now is to get back to driving and get focused all on that. I won’t have a job I know that because I can’t drive.

**I: No. It’s still quite early days in your recovery though. It must feel like you’ve been here for forever but actually it takes time to recover from a stroke. You’ve made such a lot of progress in the time you have been here.**

MP: You’ve got so many different strokes that’s the difference.

**I: Everybody is different. Yes you’ll hear that all the time but it is true.**

MP: Yes you’ve got a clot here on the side of your brain that affected the left-hand side of your body but you would think it would affect here wouldn’t you. In hindsight you think why has that gone over there.

**I: Yes all the nerves cross over they do.**

MP: Yes. But no anybody can knock anything, anybody can knock anybody. In reality if something is doing you good you can’t knock it.

**I: Good. Well thank you, it’s lovely to talk to you and hear all about your experience.**

**END**

| **INTERVIEW TRANSCRIPTION**  Voice file name: 3G Discharge Interview  Duration: 23 mins 56 secs  Typist comments regarding dictation:  **KEY:**  **I – Interviewer**  FP – Female Participant |
| --- |

**I: What I’d like to do is just ask you some questions about your rehabilitation that you’ve had since you’ve been here but particularly the physiotherapy that you’ve had. And for the study we’re particularly focused on the recovery of your leg really so being able to sit, to stand, to step and to walk so I know you’ve been working on your arm as well. You can tell me about both but I might focus a little bit on the leg.**

FP: My leg is working perfectly well.

**I: It is, you’ve recovered a lot haven’t you.**

FP: Yes. I’ve got movement back in my leg and I couldn’t move it before. I had a stroke all down my right side, I had it less than three weeks ago and I couldn’t move at all but now it’s been three weeks and I can move my arm, I can move both my legs and I can get out a chair and into a chair. But the problem is my arm is still numb.

**I: OK so your legs have recovered more quickly than your arm.**

FP: Yes because we’ve been working on my legs most of the time. But I can still move my legs I just can’t feel my arm at all, it’s numb because I had carpal tunnel in the arm before I had the stroke.

**I: So when you came into hospital about three weeks ago did you say what were you, was your leg completely weak or could you move it a little bit?**

FP: I couldn’t move it at all.

**I: Couldn’t move it at all. And the same for your arm?**

FP: Same for my arm. It was all down my right side, I couldn’t move.

**I: So I’ve just seen you get in and out the chair so you’ve improved a lot haven’t you in that time.**

FP: I’ve been pushing my arm and pushing my legs to work because I don’t want to be a cripple. I want to get back on to my feet which I can do but I still need a lot of work done on my legs.

**I: Yes so you’ve made a lot of improvement but you’ve still got a little way to go.**

FP: I’ve still got quite a way to go.

**I: OK. So thinking about that, so thinking about your leg recovery can you tell me a bit about what you’ve been doing in your physiotherapy sessions, what sorts of things?**

FP: Well we’ve been doing stretches and movement with my foot up and down, left and right to make my legs more stronger. It seems to be working really well. I’ve got two good physiotherapists doing it and they’re worth the money that you pay them because they do a hard job.

**I: Yes. You’ve been working hard, they’ve been working hard together.**

FP: Yes.

**I: So when you said about getting movement and strength back in your leg can you tell me any specific things you’ve done in your therapy sessions to help with that? What types of activities or exercises?**

FP: Well basically mostly stretching my foot and moving my foot left and right, back and forth to strengthen my leg which has worked quite well but I still need to push my arm as I’ve got carpal tunnel in this hand and it’s dead at the moment. It’s very numb. I can massage it but it’s still not working properly so I’ve got to push it.

**I: Working on that.**

FP: In different ways to get my arm moving.

**I: When you first came into hospital could you stand up by yourself?**

FP: No, I couldn’t stand up. I was actually paralysed down one side and I couldn’t move. They have to help me out of bed because that’s where I was when I had the stroke, I was in bed, and I had to get down on the floor to get the phone to phone an ambulance to come and get me and get me out of the house because I couldn’t move. I have two dogs at home that needed toileting, they needed to go out. The ambulance driver went to me, how are you going to get the dogs out? I said I don’t know but you are going to have to get in the house somehow to help me. He said won’t the dogs attack me, I said no I’ll keep them under control. I said just come in the backdoor I said the backdoor is open because I left it open that night luckily and I was on the bed and I couldn’t move. He goes well if you are on a bed and you can’t move who is going to control the dogs. I said don’t’ worry about the dogs I can control them and I did.

**I: They listened to you.**

FP: So I got down on the floor and I had Storm this side and Cookie that side. Cookie’s head was here and Storm’s head was there on my leg and, now stay there, stay there. Now you can deal with me.

**I: You must have all been very frightened.**

FP: They lifted me up on to the bed and I goes right can you put the two dogs out in the garden to go toilet. So they did that. Cookie came running in peeing everywhere and the ambulance driver went I can’t leave you like this I’m going to have to mop it up. So he had to mop the toilet up. I said thank you very much, you didn’t have to. He goes no anything for you.

**I: And they sorted it out.**

FP: He sorted it out. He goes it’s because you’ve got a stroke you can’t do it so I done it. Oh right. He goes it’s a trick for me to say that I helped someone who is poor and couldn’t move and I’ve done it. I went thank you.

**I: Ah it sounds like they were really helpful.**

FP: Yes they were.

**I: So you were obviously very weak down this side, you couldn’t stand up. Could you sit up by yourself or did you need help with that?**

FP: No they had to help me up.

**I: When you came into hospital in those first few days and you had therapy.**

FP: I couldn’t move.

**I: You couldn’t sit up either?**

FP: No I was sat up in the bed but I couldn’t move out of the bed.

**I: And sit like we are on a chair.**

FP: I was stuck.

**I: Did you work on sitting to being able to sit, was that one of the things you did in therapy?**

FP: No.

**I: Or did that just come back?**

FP: That came back naturally.

**I: Yes, OK.**

FP: But my hand hasn’t and nor has my hip. At the moment my hip is hurting and I’ve got kidney failure as well so that could be helping.

**I: You’ve got a few different things going on haven’t you.**

FP: Yes.

**I: What about when you first started to do some standing up in your therapy sessions with the physios?**

FP: That was hard.

**I: Was it?**

FP: Yes it was very hard. I couldn’t stand up. Trying to walk was hard as well but he told me an easier way to do it so I did what he’s asked me to do and I’ve done it.

**I: What did he ask you to do?**

FP: He asked me to move my, put my weight over my leg and move more forward with my head to make me move better and it works. But I don’t get it right all the time but most of the time I do. Practice makes perfect.

**I: Yes absolutely.**

FP: It’s just at the moment my hand is totally numb but I have been doing exercises and I have been stretching my thumb and everything because my thumbs got to move and I could just about move it an inch. If you look that’s it.

**I: Ah yes there’s a wiggle there.**

FP: Yes that’s all I can move it at the moment. But I can move it, it’s moving but it takes all my energy just to do it.

**I: Really think about it. Was your leg similar to that in the beginning where you had to really use all your energy?**

FP: Yes. I’ve still got numbness here in my knee but it’s not as bad as it was when I first came into the hospital, I couldn’t move my leg at all.

**I: So when you think back to that first week maybe and you were starting to practice standing up with the therapist was there anything that you focused on when you did that or you thought about?**

FP: No.

**I: You just went for it and had a go?**

FP: I’ll give it a go, I’ll try my hardest I said but it won’t be perfect. He goes I didn’t expect it to be perfect just as long as you try to do something. So I try and stand up, he goes your hand put that, I was no not at the moment I’m going to try and see if I can stand up on my own, which I couldn’t. He goes you can’t can you and I goes no, so you are going to have to help me with one arm. He helped me, he held this arm while I got up. He said there you are you’ve done it, you’ve done a quite good job as well. I said yes but I can’t stay up this tall for long because my legs ache. He said oh right you can sit down now. So we just done small ways of getting out of chairs and stepping.

**I: OK. Was there anything in all of that that was particularly difficult or was it all quite difficult?**

FP: It was all quite difficult for me at first because I couldn’t walk because I couldn’t feel nothing. But now I’m getting feeling back.

**I: It’s getting easier.**

FP: It’s getting easier but I do need more help with this arm and my hand and part of my knee and that’s it. Otherwise I’m fine.

**I: So what’s the help that you need with your knee?**

FP: Because it goes stiff and that’s why I need it to be massaged and it works because he massaged my foot day before yesterday and I could move it better than I did before it was massaged.

**I: So that helps to loosen it up do you think?**

FP: Yes.

**I: OK.**

FP: He loosened the whole, he was turning my ankle and moving my ankle in different directions to make it move and it worked better.

**I: Good. Obviously you are doing some stepping now with your walking stick here and getting from bed to chair with a little bit of help.**

FP: Yes.

**I: So as you’ve been relearning to do that to take some steps is there anything that you think about when you are stepping?**

FP: No.

**I: No.**

FP: I keep it blank because I don’t want to get confused with my stepping. I keep what I’ve got to do in here above anything else because that’s got to go perfect.

**I: So you find it easier not to think too much is that what you mean and you just give it a go.**

FP: I just give it a go.

**I: And see how it goes.**

FP: Yes.

**I: Fine. So nothing really about the movement that you try and improve.**

FP: No not at the moment.

**I: You just have a go.**

FP: I’m just doing very basics at the moment learning to walk again. That’s the main thing that I’m sticking to is learning to walk again and then gradually building it up to a stronger walk. At the moment I can only do one and that’s from A to B and that’s it. But it’s working.

**I: You’ve been here three weeks and that might feel like a really long time but actually you’ve come a long way.**

FP: It does feel like, it feels like I’ve been here forever but I haven’t.

**I: Does it. Are there any other techniques that help with your performance of stepping and walking?**

FP: No, no.

**I: Just practicing.**

FP: It’s just pure determination to do it.

**I: And if you think about, so have you seen some different therapists whilst you’ve been here?**

FP: No.

**I: Do you tend to see the same person each time?**

FP: Same person.

**I: OK. How have they worked with you in your therapy sessions, can you describe their approach?**

FP: They do an open approach to do it, hold any keys or any tap they say right we’re going to do this now, yes OK fine I can try and do it, I’ll let you see I can try but that’s it. I said I’m not pushing it. He says no you do what you can do and that’s it.

**I: So when you say an open approach you mean they’re quite clear what they want you to do and you like that?**

FP: I like that.

**I: It works well for you.**

FP: It’s better to be straight than hide around behind in your bushes. I’d rather have them straight to my face say you are doing this today. Right, OK. Show me what I’ve got to do and I’ll do it.

**I: So it’s clear, you know what they expect.**

FP: Yes I do.

**I: Anything that you would like to have been different about your therapy?**

FP: No, so far therapy has been good.

**I: OK good.**

FP: I can’t wait, I need to just carry on and get better at it.

**I: Carry on at home. Yes absolutely.**

FP: Without letting anybody down.

**I: And often in therapy sessions physios give you instructions like they tell you what to do or they might tell you how to move or what they want you to do differently.**

FP: Yes they gave me pictures of what you’ve got to do and I do that every day.

**I: So they gave you pictures, oh for you to practice things?**

FP: Yes.

**I: Some exercises to do.**

FP: Yes and I do them. There’s one that I have to do on the table which is moving my hand but just with the bad hand but not with the good hand so I have to hold my hand and do that with my bad hand to make it move but it works. I did 40 of them the other night.

**I: Oh did you? Well done.**

FP: Yes.

**I: With those exercises that they’ve asked you to do by yourself was it always clear what you needed to do, do you feel that you need?**

FP: Yes they told me what I had to do and I did it.

**I: And it made sense. How did you know if you were doing it in the right way?**

FP: Because of the pain.

**I: The pain. Feel it working?**

FP: You can feel it working, you know you are doing it right. If you don’t feel it you’re not doing it right. And I could feel everything that I’ve done so far. It’s been great.

**I: Some of your therapy presumably you were down on this ward weren’t you before?**

FP: Yes.

**I: And in the gym here. When you go to the gym and you are practicing exercises do the therapists give you much feedback about what you are doing, what you are doing right, what you need to do differently?**

FP: No.

**I: No.**

FP: He just says I’m doing quite well and that’s all he says. That’s all I’m doing quite well and he goes no you are doing very well but I’m not telling you what you are doing right and what you are doing wrong, you’ve got to figure that out yourself.

**I: OK. How do you feel about that?**

FP: I find that a lot better. I think it’s better for them to be straight then lie.

**I: Yes, oh yes.**

FP: I get all the full gist of what I’m doing wrong and what I’m doing right.

**I: So with the figuring it out yourself bit do you feel that you manage to figure it out?**

FP: I figured it out and he goes how come you figured it out so quick. I says because I ain’t dumb. He goes what do you mean, I says well I don’t act like a dumb like you think I should. I don’t I act the opposite. If I’m determined to do something I have to be determined and straight forward myself to do it so that’s why I do it.

**I: And that approach worked.**

FP: Yes.

**I: OK. So it was always quite clear what you were being asked to do. What about the way that you move, so when you are stepping on things how do you feel about the way that you are doing that at the moment?**

FP: I’ve improved so far but I still need to improve the way I walk.

**I: What do you think needs to improve?**

FP: Well the movement basically with my leg and my hip because they’re still quite stiff but I’m pushing myself to do it and I’m pushing myself to get things done otherwise I won’t do it. And I’m not having that, I’m not going to be a hypocrite or stuck in a wheelchair for the rest of my life. I’m going to get out and walk.

**I: You’re working hard, aren’t you.**

FP: And I’ve been working hard for the last two/three weeks.

**I: So when you say they are stiff is that your hip and your knee?**

FP: Yes. It’s only in this hip it’s not in this hip.

**I: No, no but on the left, on the side that’s been affected.**

FP: It’s on the side that’s been affected my hip hurts more plus I’ve got kidney failure so that’s not helping. It’s made my kidneys pain.

**I: Yes, OK. We’ve talked about exercise. You’ve talked about having exercises for your arm, did they give you exercises to do for your leg as well when you had therapy.**

FP: Yes that stretching my knees and bringing my leg up as far as I can get it to go. I’ve been doing them all.

**I: You do those on the bed do you?**

FP: I do them in bed when I’m sitting and when I’m in bed to go to sleep I do more exercises in the bed because then I can do more and I don’t get disturbed by anyone and I don’t disturb no one doing them.

**I: You can just get on and do it.**

FP: I can just get on and do it.

**I: More broadly how are you feeling, we’ve talked about it a bit but about the progress you’ve made in the last three weeks, how do you feel?**

FP: I feel 100%, I feel I’ve made good progress and I want to do more. So I’m going to be pushing myself this week and next week.

**I: So what’s the next goal with regards to your leg in particular? What do you want to achieve?**

FP: I want to get my leg better and I want to get my arm better and my hand moving. So that’s what I’ve been doing for the last three weeks I’ve been pushing this arm, pushing my leg and trying to make my arm move because I’m not getting stuck with a dead arm.

**I: When you say you want to get your leg better what do you want to be able to do that you are not doing at the moment with regards to your leg?**

FP: I’d like to go upstairs because I couldn’t go upstairs before I’m scared of falling backwards because I was getting dead legs but once that works then I will be moving.

**I: And going upstairs is a goal for you.**

FP: Yes that’s my goal.

**I: What do you think you need to do with your leg to be able to get upstairs?**

FP: Just more muscle exercises.

**I: Get stronger.**

FP: To get stronger, yes.

**I: Yes, good. So last few questions, we’re nearly there. I told you it wouldn’t take long. It might feel like a strange question but if you were to use three or four words to describe your therapy sessions what words would you use?**

FP: Really good, strong and powerful sessions. They need to push harder to get people to do more.

**I: OK therapists need to push hard to get people to do more.**

FP: Yes.

**I: Do you tell your family, do you have visitors come in to see you?**

FP: Yes.

**I: What do you tell them about therapy?**

FP: I said that the therapists had me walking 10 yards today but I only done 7 and I said I couldn’t do the others because they blocked the door off with something. I said I didn’t, I just couldn’t move, couldn’t go through a door that was shut. So they were like OK we’ll let you off. But I said you don’t have to let me off I’m doing everything I can to get home. I said do you think I want to be in hospital. No I want to go home. It’s just getting my leg moving properly and my hand moving properly to go home.

**I: I think that’s what everybody wants isn’t it, what everyone wants for you. And during those therapy sessions down in the gym particularly with the physios how do you feel when you are actually in there working on your leg?**

FP: I feel powerful. I feel that I’m doing my [*22 mins 47 secs*] good.

**I: So it feels like a positive thing because you know you are working on getting better.**

FP: I’m working to get better and to me that’s the best bit of the therapists is to push me and make me do better things.

**I: Perfect. Is there anything else about your therapy that stands out that you want to tell me about?**

FP: No.

**I: That’s good.**

FP: The therapists do it all, they hit every goal, they push you to your limit and I make sure I push to the limit to get my arm better and my legs better. That’s the reason why I’m doing this with you because I wanted to do it.

**I: It’s good. You are doing really, really well. Are you having therapy when you get home as well, are you carrying on with some therapy?**

FP: Yes. I don’t know who is coming in but I know I’m supposed to be having therapy.

**I: Hopeful that you will be going home quite soon.**

FP: Yes.

**I: I’m going to turn this off now.**

**END**

| **INTERVIEW TRANSCRIPTION**  Voice file name: 4A Discharge Interview  Duration: 18 mins 41 secs  Typist comments regarding dictation:  **KEY:**  **I – Interviewer**  MP – Male Participant |
| --- |

**I: If it’s OK I’m just going to ask you some questions about the rehabilitation you’ve had since you’ve been here. As I said there’s no right or wrong thing to say it’s your experience and how you feel. I’m particularly going to ask about the therapy you’ve had that’s been more focused on your legs, so on being able to stand and step and that side of things, so the work you’ve been doing with the chap – I can’t remember his name who you’ve just said hello to.**

MP: Liam.

**I: Liam, that’s right and with Gillian and the other physios. It would just be nice to know to start with how long have you been in hospital roughly?**

MP: Just over two weeks. I came in on the 21^st^. No is it the 21^st^.

**I: No it must have been a bit before that actually.**

MP: Before that wasn’t it. What’s the matter with me. 16^th^ sorry.

**I: OK. When you came into hospital what kind of problems were you having?**

MP: None at all, well not before I came in. All the problems I’ve got are due to the stroke.

**I: Yes so before the stroke you were fit and well and active?**

MP: I was OK.

**I: And then how has the stroke affected you?**

MP: Well initially it affected my speech which has come back as you can see. And coordination, everything was going past my ear. I had to really concentrate to get stuff in my mouth. That’s much better, so that’s improved.

**I: And that was your arm and leg was it?**

MP: Arm and leg, right one.

**I: And any weakness in your arm and leg or is it more.**

MP: Not before I came in.

**I: No but since you did come in?**

MP: Oh yes I can’t control the leg or arm as you saw.

**I: Are they weak or just difficult to control?**

MP: Difficult to control because of the stroke.

**I: OK. So what kinds of things have you been working on in therapy with the physios?**

MP: They’ve been very good. I’ve got a contraption as you can see on my foot.

**I: You’ve got a splint on there.**

MP: A splint and stocking, which I find very helpful quite honestly. And I’ve got shoes now, I had slippers and Liam suggested shoes so my son brought two pairs in and I put these on because I thought they were best. That has helped as well better than the slippers.

**I: Yes a bit more support.**

MP: A bit more support, yes.

**I: So when you first came into hospital after you had the stroke were you able to stand up by yourself, were you able to sit by yourself or did you need help?**

MP: I needed help. I still need some help, I shuffle along but I walked unaided back from the therapy yesterday with Liam coming along behind.

**I: Oh that’s good.**

MP: With a chair just in case.

**I: Was that the first time you’d done that unaided?**

MP: No I’d done it before but I got very tired before but it’s gradually getting better.

**I: Good. OK, good. So in your therapy sessions with Liam and Gillian what are the specific things that you’ve been working on?**

MP: The knee.

**I: OK.**

MP: As much as anything. The knee goes all over the place. Now I’ve got them these do-das on the, the splint, it’s definitely helped.

**I: So you’ve been focusing on the control of the knee.**

MP: Control of the knee as much as anything.

**I: Can you describe any specific exercises you’ve been doing or activities you’ve been doing?**

MP: Yes I’ve been standing up from the chair catching the frame, in the bars and I’ve been crouching down but not sitting.

**I: OK like squatting.**

MP: Squats is what I mean. Squats and up again, I’ve done loads of those. I’ve also done stepping up and down with the left and the right leg, both with Liam or whoever is with him supporting the knee again to stop it from going out. But that’s OK I’m improving. And I’ve been cycling several times.

**I: Ah good, OK.**

MP: I’ve done the do-da and again Liam sits and supports, makes sure the right knee is OK. But I find it very helpful, it’s all helping me which is what I want because I want to get home.

**I: Absolutely. So it sounds like you’ve been doing lots of work around strengthening your leg and stepping and those kinds of things. When you do those exercises, when you are in the bars practicing squats or practicing stepping up what do you think about, what are you thinking about?**

MP: I’m trying to concentrate so I do it properly but it’s not easy. I think to myself you’ve got to go through this which I’m quite willing to do because as I say the aim is to get me home. I was hoping that they were going to fix a day this week because they are arranging a home visit obviously which I’ll have to go home as well and my son and daughter-in-law who live in the village the nearest carers now they would like to be there probably with the granddaughter because she does an awful lot for me, she’s a lovely girl. But I’m not sure when it’s going to happen yet.

**I: What do you need to be able to do before you go home?**

MP: I don’t want to be dependent on my son and daughter-in-law and if I can get home I’ve got used to the wheel frame. I used to walk with a stick you see and did all my own washing, cooking. I had nobody in to help with washing, cooking and whatnot, housework. I used to go into Dorchester to do my shopping on the local bus because I don’t’ drive a car, not anymore. And if I can get home I appreciate I’ll probably have to use the do-uns but I want to be able to if to get round that house steady. That’s my aim.

**I: Yes so just working on your walking. OK, great. Sounds like you are well on the way, you are improving definitely. So just going back to thinking about those exercises and you said to me you were concentrating. What are you concentrating on, what are you focusing on?**

MP: Well the knee I suppose really the right knee as I say because I have to use, I have to as you’ve seen with my right I really have to concentrate to do things the way that they want.

**I: So you are thinking about what your knee is doing and how you are controlling it.**

MP: Yes.

**I: Is that the main thing that’s difficult – anything else that’s particularly difficult?**

MP: Not really it’s only that.

**I: OK. Perfect. Lovely. If you think about the therapists, the physios that have been working with you how would you describe their approach when you are practicing exercises?**

MP: I think they’re all wonderful I really do. They’re very good indeed, very attentive, very patient I must say. I can’t fault them at all, they’re lovely all of them. Not paid enough. They’re not. There aren’t enough of them probably.

**I: Is there anything particularly about how they give you instructions around the exercise or give you feedback that you’ve noticed that’s good or that you would prefer to be different?**

MP: It’s all good. I can’t fault it at all. Yes they always give feedback and tell you how you’ve done to encourage you and that and if you don’t think you’ve been particularly good they say that you have.

**I: OK. So lots of encouragement.**

MP: Lots of encouragement.

**I: Do they give very specific feedback about what you need to do differently or is it more just encouraging?**

MP: Encouraging but they do give specific feedback in as much as how to keep the leg OK.

**I: These are funny questions, it’s hard to think isn’t it don’t worry, it’s hard to think of the specifics. When they give you instructions about what exercise they want you to do is it always clear what you need to do?**

MP: Always clear, always.

**I: And it’s clear what you need to do to improve it.**

MP: Yes.

**I: Does that focus tend to be on what you need to do with your leg and your body or does it tend to be on just what the task is do you think?**

MP: I think it’s the leg and the body as a whole, I must say they seem to regard you as a whole person, they don’t just regard you as a leg.

**I: No, good. Fine. I suppose it relates a bit to feedback so when you are doing things how do you know if you are doing them in the right way, so if you are practicing stepping or walking when you’ve been doing walking back to the ward from the gym yesterday were you getting feedback from the therapist about how you were walking.**

MP: Yes Liam was correcting, he said your leg has gone again, and I said I know Liam but it was OK.

**I: What does he mean when he says your leg has gone?**

MP: He means it’s gone out again.

**I: It’s not where it should be.**

MP: He said if we are going along here a minute because on the way back he said I’ll get you a wheelchair because we were walking doing the round, round the unit, and so he sat me in the chair and that’s when he came back with the splint. He said you’ll find that.

**I: Ah OK so that’s quite new.**

MP: Hopefully helpful.

**I: OK. What’s the next thing that you are working on, what’s the bit that needs to improve with your walking?**

MP: It’s just I want to get home so I know more or less what I’ve got to do. I want to go home, I don’t want to be here any longer than necessary. When I get home as I said I don’t want to be dependent on anybody. I know life will change because for one thing I’ll be using the Zimmer frame and that instead of a walking stick and it’s going to take a while but my aim as I say is to get home, not to be too dependent on anyone because although my daughter-in-law is retired I don’t want her to be, she’s got her own life to lead and my son works anyway so he can’t be there. My granddaughter she’s got three lovely kiddies who keep me going but again she’s got her own life. But she’s very good, very good to me.

**I: So you want to be good enough on your feet that you can manage.**

MP: Yes so that I can be fairly independent.

**I: With a bit of help from the distance. Have you been doing any exercises by yourself, so not when you are in therapy but when you are by your bed or in your chair – what do you do?**

MP: My feet, I lift them up. I can’t get this one out of the do-dah.

**I: Do you want me to help, you’re a bit stuck there.**

MP: Yes, I lift them.

**I: Yes so lifting up your feet. Are they things that the therapy team have taught you to do or have you just worked out to practice that?**

MP: I just do them especially if I’m lying in bed.

**I: So lifting your leg up.**

MP: I move my feet around.

**I: And moving your ankle up and down.**

MP: Do that, yes.

**I: When you are doing those type of exercises what do you think about? Do you have to concentrate for those?**

MP: I have to concentrate.

**I: Are you thinking about your leg and what your leg is doing or?**

MP: Thinking about getting home mainly.

**I: You’re just thinking about getting home. The goals, you’re thinking about the overall goals.**

MP: The goals, yes.

**I: OK. And just keeping everything moving. That’s fine. Have the physios given you any exercises to do as well or not specifically?**

MP: Not specifically.

**I: OK, perfect. So we’re nearly there, nearly finished with my long list of questions**.

MP: That’s alright.

**I: I’ve got a bit of a sense of this but how do you feel generally about the progress you’ve made since you’ve been here?**

MP: I think I’ve made good progress.

**I: You can see the improvements.**

MP: Yes especially in speech and in some ways coordination. But I don’t have to have anybody feeding me or anything like that so I’m OK.

**I: Good.**

MP: I manage to concentrate and cut things up that I need to but yes I’m doing alright.

**I: Good.**

MP: We had an hilarious day yesterday making pancakes.

**I: Oh did you?**

MP: Hilarious. The therapists that we were with we made two jokes, we made the pancake mixture and she promptly knocked one over.

**I: Oh what the therapist did?**

MP: Yes.

**I: Oh no.**

MP: We all said we’re glad it’s you.

**I: Oh gosh I bet that made a mess, sticky to mop up.**

MP: Yes cleaned it all up and that. But we had some left so we were able to make, I think we made a couple of pancakes and then of course we had the spare jug so we were OK. But it was lovely to be able to do things like that. Day before we made cupcakes and that was alright.

**I: Do you do baking at home or is this new skills, do you tend to bake at home?**

MP: I used to, used to do a lot, not so much now I must admit. It’s more ready meals and that because it’s easier but it’s good ready meals, I get my meals in Cook.

**I: Oh yes.**

MP: I like their meals. But otherwise it’s, yes that’s what it is.

**I: Good, so they’re keeping you busy here making you bake pancakes for them.**

MP: They are wonderful. Oh no it’s for myself. We ate them all.

**I: Even better. So last few questions, so if you think again about your physiotherapy when you are working with the team how do you feel during your physio sessions and how do you feel after them?**

MP: I must admit that they’re a bit exhausting but as I say the aim is good.

**I: Exhausting but you get why.**

MP: Yes I understand why. Otherwise yes fine.

**I: OK. If you were describing them to your family how would you describe what you’ve been doing?**

MP: Well they are interested of course and they say to me any physio today and I say yes and I tell my daughter and my son and everybody, I said I find they’re exhausting and I have to concentrate a lot but I said it’s worthwhile that’s it.

**I: Do you think the concentrating makes you tired as well because the physical makes you tired but sometimes.**

MP: Yes sometimes the concentrating does as well.

**I: So both of them together is a bit of a double whammy.**

MP: Yes.

**I: Have a snooze afterwards. Perfect. Anything else you want to tell me about the therapy that you’ve been having here?**

MP: I think that it’s wonderful I honestly do. As I said before I can’t fault them, fault any of the team there they’re really good.

**I: Sounds like you are doing well, it’s early days actually on the path being home. Perfect that’s all my questions. Short and sweet isn’t it. END**
