# Supplementary material for: Understanding implementation of a complex intervention in a stroke rehabilitation research trial: A qualitative evaluation using Normalisation Process Theory
Source: PLoS One. 2023 Sep 8;18(9):e0282612. doi: 10.1371/journal.pone.0282612 (PMC10490858; doi:10.1371/journal.pone.0282612)
Supplement: S1 Data — (ZIP) [file pone.0282612.s004.zip › Supporting Information - Data/Interview Transcripts - Intervention Group.docx]

| **INTERVIEW TRANSCRIPTION**  Voice file name: 2E Discharge Interview  Duration: 19 mins 48 secs  Typist comments regarding dictation:  **KEY:**  **I – Interviewer**  FP – Female Participant |
| --- |

**I: Thank you for talking to me. What I would like to do is ask you some questions about what you’ve been doing in rehabilitation since you’ve been here in hospital.**

FP: Exercises.

**I: Yes and particularly exercises that have been focused on getting your leg stronger and moving. But to start with can you just tell me a bit about how long have you been in hospital?**

FP: Three weeks on Sunday.

**I: Yes OK. So just over three weeks you’ve been in hospital.**

FP: Yes.

**I: When you first came in how had the stroke affected you?**

FP: I couldn’t speak because I didn’t know the words and I tell you what they did do brilliant I couldn’t write anything because my hands were too shaky and I couldn’t.

**I: So you used some picture charts to point.**

FP: [*1 mins 16 secs* – background noise]. So like that I couldn’t tell them.

**I: Yes. So your communication, your speaking was affected.**

FP: I didn’t have any words.

**I: OK so you had no words at all. Gosh so you are doing really well, really well.**

FP: [*1 mins 40 secs* - background noise].

**I: How about your arm and your leg?**

FP: That was just totally nothing.

**I: Nothing, so nothing in your arm, couldn’t move it at all. And your leg was there nothing?**

FP: Yes there was nothing.

**I: So could you sit up by yourself or did you need help to sit up?**

FP: In this sort of chair. I probably was a bit frightened of falling out but it was OK in this chair. In bed I had bars.

**I: OK. So thinking about the physio that you’ve been having here, you’ve seen the physios and particularly about what you’ve been doing on working on things like sitting, standing, starting to step, so everything to do with your leg really. Can you tell me about what you’ve been working towards in your therapy sessions.**

FP: The big one was the skateboard.

**I: Oh yes.**

FP: And they used to put my bad leg on it and.

**I: Get you to scoot it back.**

FP: Pull it back and [*2 mins 58 secs*] toys.

**I: OK.**

FP: So I pull it right back because it wouldn’t go very far, well hardly at all to start with. Also they put me on the bed and make me [*3 mins 18 secs*] my legs up. The other one didn’t work, it does now. I’m trying to think what else. So loads of them. Where the bars are they would put your knee on there in the wheelchair and then put my arms and try and get them to go up.

**I: OK to stand up.**

FP: They did that here as well. Because they were in my way I couldn’t fall and they take me to here and then I stand up and take me to the end stand back back all the time and then they repeat.

**I: OK so lots of repeating of the same thing.**

FP: Now we’re up to 30.

**I: Wow, OK. So that’s a big improvement is it to a couple of weeks ago.**

FP: Loads of different things were there. So clever.

**I: Is it useful for you to count the numbers and know that you are improving.**

FP: Yes well for a start it was really difficult to think oh I’ll only do another five and I have to do that because they are really good and they say is that too much. I think no I want to go home.

**I: Good for you. Great.**

FP: So they’re really good.

**I: OK so lots of active things to help. What are those exercises, what do you think they are aiming to do? What are you working on when you do those exercises?**

FP: To walk.

**I: The goal is to walk.**

FP: [*5 mins 14 secs*].

**I: Yes, OK. And when you do those things so maybe when you think about when you stand up at the bars what are you thinking about when you do it?**

FP: Walking.

**I: You’re just thinking about the overall goal of walking. Yes, OK.**

FP: As well sometimes they put [*5 mins 32 secs*] so they stop me just going over.

**I: OK so to help you work out.**

FP: I’m trying to throw myself without being all over the place and just they said stand up straight, which is hard to do, and the other thing they did on the bars they had the kicking toy. I did only realise until after that it was the other one.

**I: OK.**

FP: So the one I was focusing on the other one then I realised that I was standing on the other one.

**I: Oh I see so you were focusing on your left leg which is your stronger leg doing something and didn’t realise but that was actually making you work your right leg quite hard because you were stood on it.**

FP: Yes it was balancing.

**I: Yes, good.**

FP: Good.

**I: That’s really good. Is there anything you’ve been doing that’s been particularly difficult, anything you found really hard?**

FP: Just trying to eat properly because I couldn’t quite.

**I: OK so using your arm.**

FP: I’d have food all over the place and it is really hard when you.

**I: Yes doing it one handed, yes. Any of the things you’ve been doing in therapy that are particularly hard to do with the standing?**

FP: With that foot because it wouldn’t raise until recent like so that was hard because anything I did was only just like the old foot [*7 mins 34 secs*] so that was hard. But at least it’s doing it.

**I: Yes. Good. Have you generally seen the same therapist or have you seen lots of different therapists?**

FP: Three or four.

**I: Three or four.**

FP: They went off and the one that came now.

**I: Claire.**

FP: I see her.

**I: OK. So how would you describe their approach in therapy?**

FP: Great. They quite often they just when they got some time they come and see me and they are quite happy if somebody else is here and can they come like my son came last week.

**I: OK that’s good.**

FP: And because they are so busy it’s best if they.

**I: Sorry carry on. I thought it’s not recording but it is.**

FP: But they fit it in when they can.

**I: Good. When they are asking you to do different exercises how do they give you instructions, how would you describe their instructions. Are they clear, do you know what you are being asked?**

FP: Well they’re so enthusiastic with everything, all of them.

**I: The therapists are enthusiastic. How does that make you feel?**

FP: Well yes because then I think I’d better get this right especially this skateboard because I thought they were joking with that.

**I: Is it always clear what they want you to do?**

FP: Or they show and explain to me what it’s doing.

**I: So they might demonstrate it to you is that what you mean show you?**

FP: Yes.

**I: OK perfect. What about feedback? So when you’ve been practising things how do you know if you are doing it right or not? Do they give you feedback?**

FP: Encourage me to do it the right way. Say if I do it three times and they said oh that’s done right and then they say shall we do another three and we carry on with that. So I always know what they want.

**I: Good so it’s always clear and you know you are doing the right thing. If you were doing it and it wasn’t quite right then what would?**

FP: They stop and they step in maybe Claire would bend down and she would say this is what.

**I: So she’d show you by touching your leg and showing you how to move it.**

FP: So it goes in the right way.

**I: So she’d guide it a little bit.**

FP: Yes.

**I: Yes, OK. So do you feel you’ve had the right amount of feedback that you would like in therapy, not too much, not too little?**

FP: They are so enthusiastic and Georgie, all of them.

**I: Good. OK. Do you do any exercises when you are here by your bed or in your bed? Do you do any by yourself?**

FP: They keep giving me an apron for stepping.

**I: OK yes to make it slippery.**

FP: [*11 mins 11 secs*].

**I: Oh yes they see an apron and they pop it in the bin I bet don’t they. Do you do some exercises on the bed or in the chair?**

FP: I do push the table away and then I do this.

**I: Ah good, so tapping toes, pushing the table.**

FP: Before I could only do it with the two of them. [*11 mins 33 secs*] so I can just be sitting there. And also Claire I think it was she’s showing me, can I just.

**I: Do you want me to pull it away, so move the table.**

FP: Yes. Try doing it with a pot but it doesn’t work. We tried to close that just hold it a bit.

**I: Yes. Brilliant. You are getting some really good movement coming back in your arm and leg aren’t you. So are you doing lots of practice by yourself?**

FP: With them. At night sometimes I don’t sleep because it’s how things jump where it’s and I said to the doctor and the girls I don’t know if that’s normal.

**I: Yes so what sometimes it’s just moving by itself? Yes it is quite normal sometimes when you are resting at night as well you might have a few movements. It’s just because all the messages are going to the brain and back to your arm or to your leg and not quite doing the right thing but something is happening if it’s moving so that’s not a bad thing. So perhaps if you were sat here doing some exercise for your leg with the slidy apron or on the bed where is your focus, what would you be focusing on.**

FP: Getting my leg moving.

**I: So do you think about the leg or do you just think about the thing sliding?**

FP: Oh I just think it’s going to do it. But I don’t, I haven’t got any doubt.

**I: You don’t have any doubt you just.**

FP: Work.

**I: Yes you are positive about it.**

FP: My youngest son moved out in October/November and I live on my own so I don’t want problems.

**I: So you need to be.**

FP: As well I worked up until recently so I’m quite fit because it was a job in a shop.

**I: OK so on your feet for a lot of the day walking.**

FP: Heavy things. So if I do it quickly I’ve got more chance whereas if I sit here for months.

**I: Oh no you’re quite right.**

FP: I’ll be too tired.

**I: No it’s important. Just more generally how do you feel about the progress you’ve made whilst you’ve been here for the last three and a bit weeks?**

FP: Great.

**I: So you feel positive about the progress.**

FP: I’ve had to turn it all round because they put this side [*14 mins 57 secs*] and I had no speech and I couldn’t write and I couldn’t do anything with my leg and my arm so everything turned around.

**I: Yes.**

FP: So that’s fine.

**I: You feel that you are making good progress. So last few questions, so what’s next, what are you working on next? So you are doing standing at the moment. Are you doing some walking yet or not quite?**

FP: Yes. I went down the steps. And Claire took me round to the end and her friend that end and I could do it up to the end and then go back again with her.

**I: OK so you walked with two people one either side, is that right?**

FP: Well she was in front of us.

**I: Oh she was in front, OK so only one person helping you.**

FP: Yes.

**I: Oh OK.**

FP: But they can take me back from there.

**I: So how is your walking, how does it feel, what is your stepping like?**

FP: I feel fine but I thought I was allowed to go to the toilet but they don’t.

**I: Not just yet. OK.**

FP: No.

**I: You’ll get there.**

FP: I think it wasn’t safe.

**I: Yes they just want to be sure that it’s going to be safe. In terms of what your walking felt like or looked like how close was it to normal, what do you think?**

FP: Yes fine.

**I: That’s quite good.**

FP: The few times that I went to the loo and just held onto the bar and this morning when I have a shower I’m not frightened of falling all the time like I was before because I could have just gone straight down and now [*17 mins 08 secs*].

**I: Your leg is feeling stronger and you trust it a bit more. If we were to get up and I was to help you and we walked now when you are actually doing the walking.**

FP: I couldn’t go far.

**I: No I wouldn’t but if we did what would your focus be as you are walking? What do you think about?**

FP: If you were going to there I would be trying to think where this leg is going to go next. It’s still not right.

**I: So you are thinking about what your leg is doing and where it’s going.**

FP: Yes.

**I: Yes OK.**

FP: When I’m on the toilet because the big bar next to the toilet you just hang on to it.

**I: Yes. You find a way don’t you, you find ways to do things. So final few questions. Your family come and visit you don’t they and you have people come to see you. If you were describing your physiotherapy sessions to them how would you describe them?**

FP: Two friends of mine witnessed.

**I: Ah they came along.**

FP: They came just after they come and my son came and he went along to the gym so they’ve been there.

**I: So they’ve seen it. If some friends who hadn’t been there had seen it or even to me if you had to just choose three or four words to describe those sessions how would you describe them?**

FP: Well one of them the skateboard that was very, it has helped.

**I: Good so you could see the point of the exercises could you? You could see how they were useful.**

FP: Sitting on the bed with my legs and that helped me stay fit.

**I: Great, good. So it sounds like you feel quite positive about the therapy you’ve had.**

FP: They are as well.

**I: Yes that rubs off doesn’t it.**

FP: They are so pleased when I get something right.

**I: Is that important to you when you see that they are pleased?**

FP: Yes.

**I: That’s fine. So we’re all done unless there’s anything else you’d like to tell me about what you’ve been doing?**

FP: No I’m just hoping they can give me a coffee.

**I: A coffee. Did they put their nose in whilst we were here?** **No. Oh they’re due to come round. I’m going to turn this off.**

**END**

| **INTERVIEW TRANSCRIPTION**  Voice file name: 2F Discharge Interview  Duration: 35 mins 16 secs  Typist comments regarding dictation: Participant spoke with accent.  **KEY:**  **I – Interviewer**  MP – Male Participant |
| --- |

**I: So this is participant B-06 and it’s the follow up interview. So if I just start, I’ll follow my questions a bit broadly but can you just start by telling me a little bit about what you remember from what you actually did in your therapy sessions in hospital. Can you remember what kinds of things you were doing?**

MP: Well there was some exercises such as [demonstrates].

**I: OK, yes.**

MP: The exercises I should do in order to improve my mobility and so the first thing I have to mention is that my upper limb was maybe at the beginning 75% back after the stroke after two days. The leg was absolutely dead. It was the very, very beginning.

**I: When you say dead you mean you couldn’t move it or you couldn’t feel it?**

MP: The hip joint, the knee joint or the rest of the joints they were dead. I couldn’t move them at all. They were just hanging.

**I: So heavy leg.**

MP: Yes hanging but the hand was 75%. So I received some exercise, well some plans, some paperwork with the exercises I should do in order to improve the mobility. After maybe 1½ week I managed to get my hand to 90% back so the only thing what was left, well maybe to this day when I was discharged from hospital I think it was 90% and the only thing what was left was this [*2 mins 30 secs*] practicing with the [*2 mins 33 secs*] and pretty much it and all the detail work like very, very the strength in the hand was very, very powerful. I think even maybe it could get a little bit tired earlier than the right side but.

**I: So your arm improved quite quickly.**

MP: Yes but the leg I think I, like I mentioned today the first physio research I had with you I managed, that was the first day I managed to move with the hip joint, I mean to lift that type up in the vertical, no in the horizontal position I mean I was laying on my side and I moved twice I remember.

**I: Yes I remember.**

MP: And since then I could practice because I think the path was made and I could practice more with the tight lifting it up. I think when I was discharged I was able to lift this knee on the step maybe the step was 4” high and I was able to do the stepping with 40 reps.

**I: Yes brilliant.**

MP: With Claire. So pretty much good. The next thing was the ankle, ankle is still weak, it’s still weak but I was able to do the squats after maybe a few days after I started to move with my hip. So I managed to do the squats but I couldn’t stand or rely just on the other leg because it was very, very weak.

**I: So when you were in hospital your main goals around your leg recovery what were the main goals that you were working towards?**

MP: Well I wanted to become independent walker.

**I: Walking, yes.**

MP: Yes, in walking. So that would allow me to be discharged from the hospital and it was the main goal. I put a lot of effort and the physiotherapist they put a lot of effort to get this goal. The day I was discharged from the hospital I managed to walk with a stick and with the strap.

**I: OK like a splint.**

MP: Yes attached to my foot.

**I: Great, OK.**

MP: This is the progress I manage to, we manage to do to the day of discharging.

**I: Brilliant, yes, you made a lot of improvement didn’t you, quite quickly.**

MP: It was from zero to. At that time because I would say when I came home it was 60% in the joint movement, 60% in ankle, in knee. The ankle was dead so zero and the place where the toes meet the base of the foot this joint 0% as well the movement. But I was happy that I can move on my own.

**I: So you mentioned an exercise where you were stepping up onto a step were you and seeing how many you could do in a certain time?**

MP: I don’t know if she measured the time, we didn’t measure the time.

**I: Oh OK.**

MP: It was just the technique and 40 reps.

**I: OK. So when you are practising something like that with your leg what are you thinking about, what are you focusing on?**

MP: I try to concentrate on this movement but I have in mind that I need to have the back straight.

**I: OK so posture.**

MP: I do not look how it looks I just look forward.

**I: You look ahead.**

MP: Yes just ahead. And just to think about that I’m lifting and I feel this joint and I feel the muscles which I need to, you know.

**I: Yes contract.**

MP: In order to lift this leg. I know that I can’t rush, I can’t rush and it’s better to do a little break when you have your foot on the step and you want to put back because it was very, very weak so if you rush it just goes on its own and it’s just dragging and it’s not the technique or anything. It doesn’t make sense so I think just stepping up with the right angle obviously I have support around me and they told me what I should do to avoid bad movements and stuff like that. So everything is in mind. It’s very hard because there is a lot to do and it costs lot of energy to do but I remember I started maybe from the ten reps and after the ten reps I got back on the ward and I went sleep for two or three hours because I was absolutely exhausted. But I was happy that I managed to do something and when something is done it keeps you optimistic and you know that you can have more and more.

**I: You can build on it definitely. You mentioned technique and when you were doing a bad technique or not doing it right, how did the therapist help you to correct that. Can you remember what sort of things they?**

MP: I don’t know the stair maybe you help me when you walk and you [*9 mins 48 secs*] the leg like that.

**I: So we call that circumduction, it’s a big word, but when you are swinging the leg out.**

MP: Yes, so with the stepping I was doing like that and Claire told me how you need to remember to get knee to the opposite corner to the right corner, your left knee you need to think that it needs to go to the right corner and that will help you to avoid this movement, this bad movement. What else I remember? So that walking I have a lot of instruction how to walk but because the foot was there I could not use any of it you know. But I can maybe the long step, this is the first as you, weak side is the left side and when the left side is, the left leg stays behind and you step with your right you need to make a longer step. So that will help lift the heel from the floor and support the leg on your toes. Then you need to switch the legs and again and you should put the heel but I wasn’t able to do that at that time but I was always, I remember what I was told by the physio that think knee heel, yes. So as you walk when you switch the legs so you are going with your knee up and then you put your heel and you go and you step and you knee and heel and step and knee and heel. Those things. But I couldn’t move with the heel but I think it helps when you repeat it in your mind when you are walking how it should be and you just go knee and heel, knee and heel, yes. Heel, yes. Am I right.

**I: Yes quite right. OK so you would be thinking about those things as you walked and that was helpful. Do you find that was helpful?**

MP: I think it’s very helpful to visualise the target, your objective what you want to achieve. So it’s very, very important. As you are talking to yourself you just feel, well your brain is listening and I think it stays and it stays and the focus stays. It’s exactly like you do to yourself like just relax and you have this feelings that you just relaxing yes.

**I: So would you say when you were practising exercises or practicing walking you were concentrating a lot as well as what you, you’re saying you were thinking a lot about what you are doing.**

MP: I think with this progress I make there are some stages and at the beginning obviously you have to concentrate a lot, you need to focus, you’ve got to focus. But the next stage is just get rid of the focus and make this move unconscious like it is like automating. We do not think in normal life that we are walking.

**I: No you’re right.**

MP: We’re just walking. But when you become a little bit better with your walking you just see that the concentrate on the walking is not good, it’s better to do something else. So I think it’s very important sometimes to switch from the training when you use the focus on the leg, on your walking and sometimes it’s better just walking and singing a song or talk to someone. So I think the physiotherapy is quite big and is very complex process you know. This is what I discovered.

**I: OK that’s really interesting. Do you think that you naturally just began to focus less and become more automatic because you were getting better or did you think OK I need to not focus so much. Did you make a deliberate decision not to focus?**

MP: At that time it was very, very hard for me to stay focused for a long time. So sometimes I was just.

**I: Just going for it.**

MP: Didn’t think. I was focus, focus and suddenly I was losing and after a few minutes I just realised I’m doing something else than focus on this leg.

**I: Yes, OK.**

MP: When I have problem with my hand and I was doing shifting, well just making something with the pencil when at the beginning was like every move was dropping the pencil so sometimes I was sitting and I took this pencil and I was watching the movie and I was doing this. After a few, I don’t know how long, maybe several minutes I just oh I’m tending to.

**I: Yes you realised you were doing it.**

MP: Yes that I was doing it. So I think you know everything helps is good.

**I: Is there anything else that you found that particularly helps your performance, helps as you are practicing things?**

MP: Meditation. Just calming myself because I really want to, I’m a hyperactive person and sometimes I just want to rush and have it done, have it done. But I learnt a lot after the stroke that this is no good way especially for the beginners where it’s very important to go for the quality but it’s hard. OK it’s very important to go for the quality rather than the numbers of reps or how it looks but on the other hand after the stroke after the first week you are not able to do anything but if you want to do anything right with quality you can’t do anything. When you lay down and you want to do the scissors like lifting the heel and support it on the other knee but the leg is going like that the knee, yes and it’s so hard. So you have to do it, you have to do it and you will think about quality later on when the strength will come.

**I: So there’s a balance, there’s a bit of a balance.**

MP: So I did a lot of exercising and I have therapist last week and she came and she told me how you need to concentrate now on the quality. So I think as you have a little bit of strength in the limb and if you can do 10 or 15 reps just go back in that point go back and start again and do five reps but try to do them correctly.

**I: Do you always know if you are doing it correctly? Do you think you understand the quality?**

MP: No, no, no I think there is big piece of work done with the NHS staff, with the support from NHS and the people they come, they listen to you, they see what you do and they tell you what to do. So it’s like they can control you and direct you and it’s very, very helpful because I could go around, you never where to go.

**I: You wouldn’t be sure.**

MP: You have no experience, you know nothing so I think it’s good to have someone around you who can direct you.

**I: They are a little bit like a coach, they help to fine tune what you are doing now to improve.**

MP: Yes, yes. Well I tell you what, yesterday, the day before yesterday I make my [*19 mins 53 secs*] on my own that day and I was very, very frustrated because of this foot so I couldn’t do the up movement that I’m lifting up the foot. But a week ago the movement was initiated by physiotherapy and I was very happy but as we came to the day two days ago I wasn’t able to do much and I was very frustrated. Yesterday I have the physiotherapist and I told her and she gave me a few more exercises and I started to move. I can see maybe I was frustrated because I only [*20 mins 56 secs*] tendon here. The tendon on the inside of the leg but I couldn’t see the tendons which goes to the toes which are outside and I know that practising more will cause only problems because the foot will be twisted and I will have to correct it later on. But she gave me some exercises. She told me what to do and after a few minutes, after a few reps of the exercises I saw the tendons raising up.

**I: You just needed that guidance of what to do. OK. Just again so thinking about when you had therapy in hospital and thinking about the therapists I don’t know if you saw lots of, did you see lots of different therapists, physios?**

MP: Well yes the team is quite complex, there are many, many people.

**I: So is there anything, the therapist who worked with you the physios how would you describe their general approach with you?**

MP: Professional you know. Professionals.

**I: How did you feel in your therapy sessions generally?**

MP: How did I feel?

**I: Yes.**

MP: Well very hard at the beginning. The feeling after the stroke is something you know which you can’t explain to yourself. Suddenly the life of death it becomes clear that we won’t stay forever and so fragile the life.

**I: Yes life is fragile.**

MP: One day you are running and the other day you are not able to get from the bed on your own. So all these makes a little bit, it brings you spirit up and you start thinking a little bit deep and more. I thought after a week that I won’t be able to walk anymore in my life. I was trying to keep optimistic there are many things what I can do or even on the wheelchair there is no problem, the walking is not working but because I’m an active person and I spent the last few years and I gave much to Tai Chi and I really like it so I was very, very frustrated because I spent a lot of time but now everything went.

**I: Very suddenly it changes.**

MP: It can go to the rubbish bin and that’s it. But as soon as I had this session with you and I managed to raise the knee I said there is something, the miracle happened and I know that if I move this knee up the thigh up so I can carry on and I will get when I want and you know the progress is not big, it’s very, very small [*24 mins 35 secs*]. You put day by day and you build up but definitely you are going up, you are going in a good way.

**I: So seeing progress is important you hope keeps you motivated.**

MP: Yes, yes of course. After a week or week and a half when you can’t move your leg your motivation and everything just disappears.

**I: When you are doing actual therapy and exercises is there anything about the way the therapist gave you instructions or gave you feedback about what you were doing. Did you find that helpful?**

MP: I don’t know if I should say.

**I: It’s confidential.**

MP: OK they always will say you are doing very good, yes, very good, so obviously it’s good keep the optimistic way but you know if you can’t do nothing and the nurse comes in and oh you are doing well, you are doing well, it’s good to hear that definitely it’s better to hear that she would come oh you are do bad. But because me as a stroker I have no experience and I didn’t talk much to the people who had a stroke and how do they, what they were thinking about. But when you have this staff, people around and they are very careful, I mean careful they look after you. I was a little bit, sometimes I was a little bit crazy, I did some things on my own but they were you should not be doing, you know you can fall on the floor, you have problems you can break your hip. You know. You can make bad things, very mess. But they always get motivated myself to do more and more because I thought that sometimes you feel the person you meet is a professional how she behaves, how she does or he does the tasks, how the person deal with you, their way. And you know that you say you just feel that.

**I: You feel safe.**

MP: You can do it because you know when you meet people you know sometimes in your day life with no professional approach. They are not too good yet and you just doubt. This feeling of the so sorry.

**I: So with the feedback, they gave you lots of encouragement, lots of you are doing really well it’s good, is there anything you would have wanted to be different or that would have been more helpful?**

MP: I can not much difference because they only thing I wanted, I want to go closer and quicker to home when I could do some things on my own but I know that in the institution like hospital the people they have certain rules and they need to abide by the rules. We have Michael who wants to do it on his own who knows everything better so I had to calm down. I know that the people they were responsible for myself, for my health but because I do not use this kind of service much in my life so I don’t really know how to deal with these people as well you know. So I decided to just slow down, to just, yes.

**I: OK. When you were in therapy and you were practising tasks or exercises and they told you what they wanted you to do was it always clear, did you always know what was going on?**

MP: Yes.

**I: The instructions were clear.**

MP: I was shown by the person who provided training, who gave the training what the move, how it should be.

**I: Shown like they demonstrated it to you?**

MP: Yes, yes they did demonstrate it.

**I: And that was helpful?**

MP: Very helpful.

**I: Did you find because you saw a few different therapists were they all quite similar in their approach?**

MP: Yes I think yes, yes they good teamwork and they good in their singular approach.

**I: All quite similar. We talked about it a little bit but if you were doing something and you maybe weren’t quite doing it in the right way like you said when you were stepping swinging your leg out what did they do to correct that? Did they tell you what to do differently, did they show you?**

MP: Yes, yes straight away when the issue was discovered the instructions of how to improve this move, what to do.

**I: Was that helpful?**

MP: Very helpful, very helpful because it’s good because when you have stroke and your mobility is affected you don’t really know you should do things. So for me that I was doing this.

**I: Circumduction.**

MP: Yes the circumduction I thought it’s OK, it’s normal with this thing, it’s normal thing with this thing. But she told me no it’s not normal you will have problems and it will go worse and worse so we need to switch for the right technique. So when you walk you need to lift the thigh up, knee up and try to keep this knee to the, well it’s not that you are walking and you move this movement to the right corner.

**I: So like a target that you are aiming for.**

MP: Yes it’s just thinking that you are doing to the right corner but in real life the knee goes straight up.

**I: Yes got you. OK. We’re nearly done. We’ve talked a bit about your progress and the progress you made on the stroke unit so ultimately you would like everything to be faster but you feel positive about progress.**

MP: Every goal I achieved I wish it would be bigger goal and I want the progress was quicker but it is what it is and you can’t really do but I think that it’s like a big train which just started and it starts slowly, slowly, slowly and it gradually pick up the speed.

**I: The momentum.**

MP: And it goes and it goes and it goes and I know that week after week the progress will be done bigger and bigger because when you become more independent with doing things you can do more and more and more, you can exercise. From the beginning you can’t exercise too much just exercise an hour after one and a half week after the stroke an hour of exercising makes you so exhausted so you will be sleeping for 24 hours or 12 hours you know and that’s it.

**I: So you have to pace yourself.**

MP: Yes.

**I: Great, good. So we’re done. Is there anything else that you wanted to tell me about your therapy?**

MP: I want to thank you guys for your work and for what you do. This is so good. Very kind, very kind.

**I: We enjoy seeing people like yourself, you work hard and you see the benefits and it’s great to see improvements that you are making and keep making, you will keep making them.**

MP: I think seeing all the research, all this work what you do for the people you know and yes I forget to tell you when I was leaving the hospital on the ward my roommate the day I was leaving he asked me to tell you that if you can do for him what you do for me and I forgot to tell you that but now please tell it to the.

**I: I will do.**

MP: Those guys.

**I: Yes I will do. Right I shall turn this off.**

**END**

| **INTERVIEW TRANSCRIPTION**  Voice file name: 2G Discharge Interview  Duration: 29 mins 12 secs  Typist comments regarding dictation:  **KEY:**  **I – Interviewer**  MP - Male Participant |
| --- |

**I: If you’re happy we’re going to be talking a little bit about your rehab particularly your physiotherapy in hospital and particularly the work you did, more to do with your leg than your arm.**

MP: When I was on the parallel bars up there.

**I: So all that kind of thing. Can you remember back when you first came into hospital and what was difficult for you at that time?**

MP: Difficult was trying to accept it. I knew what had happened but it was very difficult to get my head around it. It was almost like a panic mode. Complete and utter disbelief it’s happened to me. I think the worst time was the first day I was there was that I hadn’t, well I couldn’t go for a number two and the next day they were going to give me some laxatives and of course they’re very, very short staffed and there’s this one Chinese gentleman. I said to him I need to go to the toilet now but he was still busy seeing to someone else and I stupidly got up and of course I couldn’t even stand up then let alone balance and I fell on the floor and then the next minute I had about 30 people around me with the inflatable balloon to get me up.

**I: Yes it lifts you off the floor.**

MP: So I then went into another ward but I’d say that’s probably the worst experience I had there. It was my own fault because obviously he couldn’t see to me straight away but it was one of those things where you had to go you had to go and stupidly I got up.

**I: So the stroke had affected obviously your arm and your leg. Can you tell me a bit about how the stroke affected you specifically?**

MP: Well I think probably most of it was to do with I felt immensely tired all the time, mega tired. There were so many things running through my mind of absolute stress, panic, not being able to work, will it get any better. It was just basically the stress was unbelievable, it was off the charts. Angry as well even though I didn’t really show it much. But it’s the most stressful thing I’ve ever had in my life. I’ve had some bad things happen but nothing like that at all. I couldn’t accept it. Of course I went to [name] Hospital before that a few days before that and they let me out but it was too soon and of course I came home for one night and of course I couldn’t even stand up let alone balance and of course I fell down to the toilet upstairs in the house, landed on the toilet pan and the cistern and smashed all my back ribs in.

**I: Oh gosh.**

MP: It was so agony and of course I had to go back in because it had got actually worse overnight. That was stressful having to go back to the same hospital again. I stayed in there for a couple more days and then they transferred me to [name] hospital then, which has got a better rehabilitation centre.

**I: So when you went over to the hospital at that time how much could you move your arm and leg and how much could you do for yourself?**

MP: Well my leg itself to be honest with you I wasn’t paying too much attention which part of my leg wasn’t working, all I knew that I just couldn’t walk and my arm itself I couldn’t even move it. I couldn’t move it at all, nothing in it. I was just basically wondering really what was happening to me.

**I: Could you feel your arm and leg it was just that it was very weak.**

MP: I’ve always been able to feel everything which is not working, I managed to feel everything. I had a slight tingling on my face which I noticed about three days later after going to the hospital. My mouth had dropped slightly on the right hand side because the left hand of the brain had caught but that tingling seems to have gone off of my face now. I’ve been doing all these exercises, moving your mouth and smiling and grimacing and all that and that tingling seems to have gone off now. They’ve been very good, some of the early exercises they didn’t get me to walk until I’d been there about two weeks. It was all very well, I started doing a few arm exercises and a few leg exercises and they put an electrical stimulus on my leg and that seemed to have got my ankle working upwards and downwards, which they left on for half an hour. That seemed to make that work a bit better. The first time I walked I think was about two weeks after being there was actually with the gutter.

**I: OK gutter frame where you have your arms up.**

MP: Which I walked into the therapy room with and I found it a bit awkward because if I leant on my right hand I couldn’t walk properly or anything like that because it was so weak on my right-hand side of the body that leaning on it I was going like that downwards on one side, on my right-hand side and I couldn’t keep my balance so in the end they gave me the [*5 mins 26 secs*] stick after about a week. That was a little bit better.

**I: So when you went over to the hospital because that’s when you really got going with your rehab isn’t it, can you remember what were your main goals? Was it around being able to walk?**

MP: Well being able to walk and try and get better really if I could. It’s really a question of this is not me I’m always so fit and healthy, not fit but healthy, just really wanted to try and get things going. But it’s a hell of a strain and a hell of a worry. It’s the worst thing I’ve ever had to deal with in my life. The stress is off the charts it really is.

**I: Does that affect how you feel about your rehab, at the time do you think that affected?**

MP: I think it did actually really because I didn’t really know what they were trying to do most of the time, how this exercise will actually help. [*6 mins 26 secs*] get back home make me feel a lot better, I was missing home and all that, wife, the dogs and all that and I’d been in there, how long was I in there for, a month I think it was.

**I: Yes I’m not 100% sure. It must have been three or four weeks.**

MP: About four weeks I think it was. I just wanted to come home and I thought I’m not going to do anymore here because I’m just so not homesick but I just wanted to get back to normality if I could.

**I: Nobody wants to be in hospital if they don’t have to be.**

MP: That’s right.

**I: So you said with some of the exercises sometimes you weren’t really sure how they were helping or what they were for.**

MP: That’s not on pause is it?

**I: No it’s not, no. Don’t press that otherwise it will be on pause. No it’s definitely recording.**

MP: Sorry.

**I: That’s alright. I’ve lost my train of thought. So sometimes you weren’t sure what the exercises were for, can you give any examples?**

MP: Well it was like when we did the exercises with the glass and the water in the cup and the jug I knew full well I couldn’t do it and I was picking the jug up and of course some water went everywhere. Did all that probably a dozen times over the four weeks I was there and well the only thing I really wanted to do was try and get walking if I could. I didn’t see much improvement with the hand at all. The walking I did after about a third session it was getting a bit better but I was wondering what they were trying to do. I thought how is this actually helping my rehabilitation and of course I was so stressed out, so stressed out in the hospital it was unbelievable.

**I: Can you remember any of the specific exercises you did more for your leg or for walking? Are there any that stick in your mind?**

MP: Well they either made me walk or asked me if I wanted to walk or they pushed me through in the wheelchair but they originally took me through on the, what’s the stand-up thing like a [sack truck] you stepped onto it.

**I: Yes steady we call it but it’s like a sack truck.**

MP: Yes they were originally wheeling me in on that. I thought oh bloody hell this is pathetic, I couldn’t even wipe my own backside if you know what I mean. Of course I fully praised the male nurses, I thought to myself what have things come to when you’ve got to go to the toilet and you can’t even wipe your own backside. It was so personal. I said to them, I’ve forgotten what his name was now, I said I don’t know how you can do this job. He said I’ve been doing it for 6 or 7 years and I said it’s so lowering for me to have somebody wipe my backside it’s unbelievable. So I forced myself to try and make sure I could do it myself, which I did after about three weeks. But everything about it I’ve always thought I’ve been so independent now I’ve got to rely on someone to actually dress me, wipe my own backside, it’s terrible.

**I: It must be incredibly hard.**

MP: Oh it was incredibly hard.

**I: Your dignity.**

MP: My dignity went out the window and I mean completely went out the window. It’s different now, I’ve taken the frame around from the toilet and I can do it myself obviously with my left hand but I do manage it and I can have a shower now obviously with the wife monitoring me.

**I: You’re doing so well.**

MP: And I can get up the stairs but I think it’s sheer determination really. I just want to try and get back to normal if I can.

**I: So at the very beginning were you having the sack truck thing for getting in and out of bed as well?**

MP: Yes from the very beginning if I wanted to go for a wee or something like that because the toilet pan was so small if you didn’t make sure everything was all ready to go you were all over the place and it was terrible. A little bit embarrassing. But yes they were taking me on the sack truck and because I was quite a big guy there was two people, they had to muster up two people. If I was bursting to go I had to wait 15 minutes. It’s not their fault because they’re doing other people but it was just a question of trying to time it right thinking I’m going to have to go now I’d better press the buzzer now and that’s it really. But they were busy because obviously no-one came to the buzzer immediately because they are dealing with other people.

**I: So you obviously were working on being able to get in and out of bed to transfer without that equipment, the physiotherapists were helping you with that. What sorts of things were you practising to help you be able to stand?**

MP: Well I was doing the arm exercise. The arm exercises sheet they gave me when I first came back home I was doing all those exercises some of them with the leg, some of them was with the electronic stimulation on the right leg to make the toes come up. That helped me a little bit. With the arm itself she did electronic on my arm which actually flipped up my wrist. She put it on to try stimulation and it will actually go up further on the wrist, that helped me a little bit. Then I used that gripper ball I think it is which is a little video play thing.

**I: Yes I know that one.**

MP: Have you found the lead for that yet?

**I: No.**

MP: I haven’t got it.

**I: No don’t worry, it’ll be on that ward somewhere. Sometimes things get scooped up in the laundry.**

MP: I had another look in the green bag I had because I’ve got another charger in there as well and it’s not in there.

**I: Don’t worry we can get a replacement that’s fine.**

MP: But no I used the gripper ball thing and that helped me with my hand a little bit when it was working because the wi-fi was unreliable and it had to have, it had to be connected to the wi-fi and the blue tooth wouldn’t work sometimes after you’d used it for 20 minutes the hand grip ran out of battery power.

**I: Yes the wi-fi is quite dodgy in the hospital as well.**

MP: Yes and if the wi-fi is not working you couldn’t get on to the machine. It was a good idea, it did actually work but I think it’s probably a little bit unreliable. I think they need it non-wi-fi-able.

**I: Yes I think you’re right.**

MP: Something you plug in like a video game and do it with nothing to do with wi-fi or whatever it is.

**I: So that’s quite a lot of things for your arm were there any specific things you remember about doing for leg strength or being** **able to stand? What did you work on with the physios.**

MP: First of all rather than going on the bars that was the first thing they put me on but they put me on that sofa bed in the physio room and just seeing which way doing the exercises like sitting down and moving your legs outwards like that and then lying on my side and seeing if I could bring my leg round to my back. Then basically it was in the wheelchair between the bars just standing up to see if I could walk along. I could hardly walk but I did that probably about a dozen times over the four weeks. It gradually got better, gradually got better but I just go so tired and I was so stressed out. Of course they were so busy doing other things as well I thought to myself well am I having any therapy today and some days I didn’t and that used to worry me a little bit because I used to sit there like a vegetable in the hospital in the room. But I think one of the worst things I had in the first week and a half was because I fell over they put me into another room further down and of course on that room there was a bloke on there who kept on talking all through the night and he kept on getting out of bed so obviously on the ward when they had to watch people getting out he’s particularly trying to get out of the bed every five minutes so someone had to sit with him all the time. Basically he was talking all night, he wouldn’t go to sleep, the only time he went to sleep was in the daytime when I was awake and I had two weeks of that and I said I can’t take this anymore, I’m not getting any sleep. I was getting irritable. Even one night I told him to shut up Christ sake. It’s not like me.

**I: No but you don’t normally share your bedroom with other people do you.**

MP: And there was a man opposite me who was probably about 80/90 years old and he was in bed all the time and he kept on weeing himself so he needed changing about every two hours. That used to wake me up in the middle of the night. But this other chap here it was just constant the whole night and sometimes I was getting half an hour sleep and he was talking. He wasn’t just talking quietly he was and of course there was so much noise going on where they were talking to him to go to sleep, go to sleep and then five minutes later he’d get out of bed again. Then I think it was about 12 o’clock at night I can’t remember what happened they were doing something on the ward and he was in his chair and he got out of his chair and I shouted to one of the nurses, I said he’s getting out of his chair. Too late he fell down and crashed headlong into the ground. He didn’t hurt himself but I thought to myself that’s it I’ve had enough. I said to one of the doctors I said look I’m not getting any sleep and then they put me into a separate side ward which was a massive improvement, I was getting sleep.

**I: You need the sleep for recovery don’t you.**

MP: Yes and I was in there for about a week and a half I suppose and that was absolutely brilliant having a separate side room. I said I wouldn’t mind going home and they had to arrange all the back-up for the after care and all that which is very good, everything was delivered here.

**I: So going back to the parallel bars you mentioned. When you were first standing up and trying to take some steps in the parallel bars can you recall what were you thinking about to do with when you were trying to walk. Were you thinking about anything in particular?**

MP: First thing I was thinking it was the amount of effort it’s taking me and I was getting angry because I couldn’t move my leg and I thought just move you blooming leg. I was thinking what’s the problem just work. I noticed a slight improvement after about a week and they put me on the bike again and that helped me a little bit. They did a few mental tests with me as well and of course trying to remember things, my brain well even now it’s not 100% really. If you’ve got to think of something you sometimes have trouble recalling something sometimes occasionally if I’m trying to get something out. I can speak OK normally but if I’m trying to think about something I’ll turn around and say Christ I know this, why can’t I remember it. But it’s a bit frustrating. It’s only because I’m so stressed as well. But I wasn’t getting any sleep much either.

**I: It’s a bit of a vicious circle when you are not sleeping and you are feeling stressed and then your brain is like argh it’s harder.**

MP: Yes exactly, exactly.

**I: It sounds really tough. When you are walking now, when you are practising at home what are you thinking about as you walk?**

MP: I’m just trying to think about which part of my foot is not working, why is my foot dragging. I think basically the one part that isn’t functioning properly is the walking on my heel which that machine does help quite a bit but it takes so long to set up and get it right. I have got a shoe down there, a little plastic thing that goes in the inside of your shoe that keeps your foot over 90 degrees. That makes you walk a bit better but I’m not too sure if that’s helping me at all with regards the muscle that’s not working. The main problem is that pivoting muscle there which isn’t working.

**I: That’s right so the splint essentially as you know just holds you there so you don’t catch your toes but it doesn’t encourage your muscle to work. The battery is low, I thought that might happen. So that’s where sometimes the electrical stimulation is a bit better because it makes your actual muscle work rather than just support you.**

MP: I was going to say rather than substituting it for something else.

**I: Yes exactly you are right.**

MP: So that’s why the stimulus is quite good but the problem is it’s setting it all up.

**I: That’s it.**

MP: It took me about half an hour to an hour to set it all up properly and then I was just walking around with the girl who came in up and down here to see what it was like.

**I: It will get easier though, you will get a bit more slick with it. When you were in hospital did you see quite a lot of different therapists or did you tend to see the same person?**

MP: No, no I must have seen probably three or four, four or five maybe. The occasional doctor came in and asked me how I was and the psychological person came in and asked me how I feel. I can’t remember their names to be honest with you.

**I: No that’s alright.**

MP: Half the time you couldn’t read their badges anyway or they didn’t have it on.

**I: Were the therapists all quite similar in their approach did you find?**

MP: Yes all very similar, yes. With masks on they all look the same.

**I: It’s not very good is it for seeing people.**

MP: No not really no.

**I: I’m interested when you were in your therapy sessions like when you came down to the gym how they gave you instructions and how they gave you feedback and was there anything about that that you found helpful or not helpful?**

MP: Yes they were very reassuring and said if I can’t do something they said we’ll leave that for the moment. But if I hadn’t seen one of them for maybe two or three days and they’d say oh that’s an improvement and all that and I couldn’t see the improvement. I’m not too sure if they were just saying it or not but I was improving but very, very slowly. In my mind I just sort of cancelled it basically in my mind even though I was getting better. But no they were very reassuring if I managed to do something. Some days I couldn’t do the things I did the day before and then the next day I could do it. It depends what state of mind I was in.

**I: But knowing that you were progressing was important for you like having that information.**

MP: Yes and I wanted it to be enough so that I could actually stand up, walk around. The advantage was for me to actually walk around with a crutch at least and even be able to walk, which I can do now with the aid of a quad stick or the single one. But I managed to do that so I am actually mobile even though it still affects every single second of your life. You can’t do anything, if you drop something on the floor you’ve got to plan how you pick it up but it’s made me more vigilant. But no the parallel bars they had they bore with me I was doing it once for about an hour and a half each day, well most days, and that helped a lot I must admit.

**I: That’s good. So just going back to the way that they asked you to do things was it always clear what they wanted you to do in your therapy?**

MP: Yes pretty much so. Yes pretty much so. We seemed to do the same routines all the time and then sometimes they got a group class in with four people in it and we all attempted the same things and the jug and the playing cards and the pegs on the end of the cup which I couldn’t do very well. My arm was very weak. We did that quite a few times. Some days I could do it and some days I couldn’t but it’s a bit frustrating when I could do it a week before that and I couldn’t do it that day that used to really frustrate the hell out of me.

**I: Did you feel you had much choice around what you did in your therapy sessions? Did you have any control over it?**

MP: Yes I would have thought so but because I didn’t know really what the therapies were, they tried a few different things most days and if I couldn’t do them they’d say OK we’ll leave that then. They didn’t force me to do it or anything like that. But no they were pretty concise, pretty clear with their instructions, yes.

**I: How did you know if you were doing things in the right way? Did they give you feedback? How did that work?**

MP: They used to say to me if I’ve done it just say well done you’ve done that, that’s very good. If I did something they weren’t expecting me to do and I did manage to do it they praised me. They were pretty good to be honest with you.

**I: That’s good. You’ve been practising loads of exercises not with the therapists, so you do a lot for yourself as well. Were you given things to do in hospital?**

MP: Yes but it was very limited. It was down to stretching my arm, trying to move my fingers. But as I say it was mainly the basic stuff and the electronic stimulation on the foot and on the arm as well. That’s really all you could do sat in hospital really. Then I think it was the last week I then started moving with the stick a bit which made me feel a bit better because I was actually managing to hobble along.

**I: So you went from needing the sack truck and the two people at the beginning. So when you left hospital you were walking with the quad stick were you?**

MP: Yes.

**I: How did you feel about the progress you made in hospital?**

MP: Well the problem is me being a bit impatient even though there was a bit of an improvement there was an improvement I just wanted to be completely improved. I was very impatient. I could see yes there was a small improvement but I was just so intent on getting back home.

**I: That was where your mind was and the focus was on what do you need to do to get home.**

MP: Yes exactly.

**I: I think that’s really understandable. I think that’s pretty much everything I wanted to talk to you about really. I’m just checking I’ve done everything. I do ask this question which sometimes people find a bit odd but it’s thinking again about your therapy sessions, your physio sessions in hospital, what words would you use to describe them? If you were telling someone else or someone in your family what words would you use to describe your physio sessions.**

MP: I was a little bit worried that I couldn’t do some of the things. They didn’t push me at all, if I couldn’t do something it was OK they would try something else. I just thought to myself why am I doing this particular exercise, why will that improve me and they explained to me why it would.

**I: So would you have wanted a bit more information about that do you feel?**

MP: I would have liked to have known what a particular exercise I was doing what muscles that was trying to improve or what will that improve. I would have liked a bit more feedback back to me because they knew what was going on, they knew what they were trying to do but it wasn’t explained to me completely. They just said that strengthens so and so muscle but there wasn’t enough detail in it. It might have given me a bit more meaning of knowing the reason why I’m doing some of the exercises. But as I say they know which muscles are what.

**I: It’s interesting to hear you say that. Do you think that would have helped you? Is that because you are just inquisitive and you want to know or would it have made you feel more motivated to do them?**

MP: It probably would have made me feel more motivated even though I was motivated as much as I could. Of course it wasn’t just that it was all the other problems, basic things like eating, trying to go to the toilet. All these things are on my mind which is just the whole ceremony of it all, the whole saga of it all, it was just completely alien to me completely 100%. But it would have been nice to have known exactly what a particular exercise is supposed to strengthen what part. They’d say oh that will strengthen your arm rather than that’s your so and so muscle.

**I: You wanted a bit more detail.**

MP: Yes it would have been nice but I was just being nosey what.

**I: Well it’s your body you’re allowed to be nosey.**

MP: Basically yes.

**I: Fine. That’s it. Is there anything else about your therapy that you would want to tell me about?**

MP: Well some of the things I explained to the therapist as I’m going along and I am getting slightly better is knowing why doesn’t my ankle turn. I noticed that the other day. Does that need therapy if that hasn’t got any better. Why am I still limping and all that. I still can’t get my heel down on the ground and I happened to mention it to one of the nurses or the physios that come in and they said we’ll try so and so on it then. They give me the therapy when I’m here but unless I state there’s something in particular I’ve noticed isn’t working they say oh we’ll find out about that and we’ll see if we can get a machine for that or whatever it is yet they have to be prompted a little bit. But I know it’s difficult because it’s not their body but I know which parts aren’t working.

**I: Yes, sometimes there’s lots to work on isn’t there and sometimes we need to do it in a bit of a staged way so that you are doing enough of different things. I’m wearing you out.**

MP: Even talking like this I get tired. It’s ridiculous, it’s crazy really.

**I: Well you are using your brain so it’s really common that anything that’s concentrating, thinking, I’m asking you loads of questions.**

MP: I never used to be like this at all.

**I: It does affect. That will improve.**

MP: Anytime I was like this was about 1am.

**I: Yes out on the town.**

**END**

| **INTERVIEW TRANSCRIPTION**  Voice file name: 2I Discharge Interview  Duration: 13 mins 15 secs  Typist comments regarding dictation:  **KEY:**  **I – Interviewer**  MP - Male Participant |
| --- |

**I: So what I wanted to do because you are going home quite soon is just ask you some questions about the rehab that you’ve had whilst you’ve been in hospital particularly rehab that’s focused on working on your legs, so rolling, sitting, standing, those things that you’ve been doing in your physio. It’s just to hear a bit about your experience really so again there’s not a right or wrong thing to say it’s just about what you think really and what’s happened whilst you’ve been in hospital. Did you say to me you’ve been here about three weeks?**

MP: Yes, this is the third week. I started off in [name of hospital] on Friday night, early Saturday morning. I was there until I think it was Monday, Sunday afternoon, then they brought me over here from there.

**I: Do you remember coming into hospital? Is it a bit of a blur or do you remember?**

MP: Yes I remember the ambulance driver talking to me all the way through telling me exactly where we were, going down the road, what part and he said we’re nearly coming into the carpark, now coming into the hospital and that was it.

**I: Do you remember how the stroke affected you at the very beginning. What were you able to do and not able to do?**

MP: I couldn’t move at all because my wife was told to drag me off the bed and put me on the floor in a horizontal position.

**I: Oh goodness.**

MP: On my side, yes, so, then the ambulance people turned up. I can remember them talking to me, I remember all that so I was conscious then. The most she dragged me out of bed I was conscious. I knew exactly what was going on.

**I: So you couldn’t move the right side of your body at all?**

MP: No.

**I: Could you feel it? Could you feel the right side?**

MP: I don’t think I could no.

**I: That must have been really frightening.**

MP: Yes.

**I: So you were at [hospital] for a little while and then you came back over here to [hospital] to carry on with your recovery.**

MP: That’s right, yes.

**I: In your therapy sessions can you tell me a little bit about what you’ve been doing and what you’ve been working on?**

MP: Legs, I’ve been using my arm in the bed more mostly because exercises lifting up and finger movements and that. So legs that’s the main problem.

**I: So what have you been working towards with your leg?**

MP: Rolling on the bed and trying to lift it up as much as I can, bending the knee, trying to, yes just general movements and that.

**I: When you were first in hospital were you needing the hoist to get in and out of bed or did you always use this?**

MP: I only used it once. I can’t remember what that was for now because I’d not been out of bed at all I think I used it once just to go on the commode.

**I: OK.**

MP: Yes that’s the only time I was on that.

**I: OK good. So you’ve been working on practicing getting in and out of bed as well with the return equipment.**

MP: Yes.

**I: When you’ve been practising that kind of task moving from bed to chair what do you think about, what are you focusing on?**

MP: Getting it right to be quite honest.

**I: What do you mean by that?**

MP: Well getting in the right position and getting upright in the middle, standing upright and getting my feet right and getting the grip. Just make sure I’ve got the right balance really in the centre. I keep upright and things like that.

**I: When you’ve been working on sitting and sit to stand have you been working on that with the therapist?**

MP: Yes.

**I: Again when you are doing those sorts of tasks when you are doing movements with your leg what sort of things do you focus on?**

MP: I’m not quite sure. Getting it right. I look at it and think try to push myself to do it. Always trying to force yourself to do it. Maybe I’m trying to force it too much but.

**I: OK. Do the therapists give you a lot of guidance on what to do?**

MP: Oh yes definitely.

**I: Is it always clear what they’re asking you to do?**

MP: Yes very clear, very clear indeed.

**I: Good. How about feedback, have they been giving you any feedback on how you are getting on?**

MP: Yes, yes. As I say everyone has agreed that the arm is getting ever so well it’s just the leg is a bit slower but it’s gradually getting there it’s just a slow progress.

**I: When you are in your therapy session what kind of feedback have the therapists been giving you, can you remember anything that they said?**

MP: Yes saying you have to do it right and then they correct you if you are doing it wrong and things like that. They do help a bit with bending the knee and things like that.

**I: Is that helpful?**

MP: Oh yes very much so, yes, yes. Yes definitely because I take it all in, definitely take it all in. They said I’m very determined.

**I: It makes a difference.**

MP: Makes a difference, yes.

**I: Have you been practicing things when you are not with the therapists? Have they given you some things to practice by your bed or in bed?**

MP: In bed, yes, but it’s awkward sometimes because some of the ones you can’t do on your bed like going like that.

**I: Yes bending your knee up.**

MP: And going from side to side. You can’t really do that on the bed because the moment you get up in the morning they put you in the chair. I don’t really want to lay in bed all day long.

**I: No, no.**

MP: So when I’m in the chair I just practice kicking it and trying to bring that foot forward and backwards and going like that, trying to go like that with my knee. That’s all I can do is try, keep trying.

**I: When you are doing those sorts of exercises in the chair how do you know if you are doing them right?**

MP: Just by what they tell me to do. I’m trying to keep when I lift that leg up I hold it as long as possible and each day I try and hold that longer. Try and straighten it longer.

**I: So timing it or counting.**

MP: Timing it yes.

**I: That’s a good way of indicating to yourself.**

MP: That tells myself I’m doing it better and longer each time. It’s only a couple of seconds but it all helps doesn’t it.

**I: Yes absolutely. It gives you a bit of a goal doesn’t it so you can tell.**

MP: Yes I know when until I get that knee moving properly I’m not going to be able to do any strides. I know that in my mind so.

**I: Working step by step. Pardon the pun.**

MP: Yes.

**I: OK, great. When you’ve come along to therapy you are in your therapy sessions do you feel that you have much input into what you do, do you have much choice or do the therapists guide you?**

MP: The therapists guide me so they know what process to do.

**I: Do you feel happy with that?**

MP: Oh yes definitely, yes. I tell them if there’s a problem if I’m not doing this I can’t do that then they listen to it and they try and correct it.

**I: Do you feel like you have some control over what you are practising and what you are working towards?**

MP: Oh yes I think so, yes. I think you are working as a team really. That’s the way I look at it.

**I: Yes that’s a good way to look at it.**

MP: Yes. They’re the instructors and I’m the novice.

**I: Well you might be a novice at stroke rehab but you’re not a novice in being [name] so you know what you want.**

MP: That’s right.

**I: Have you tended to see the same therapist each time or have you seen lots of different people?**

MP: Seen one mainly. Mainly [name] and [name].

**I: OK brilliant.**

MP: Which I think is a good idea because they know your movements and that.

**I: Yes it’s a bit of consistency and they can build on what you’ve done before.**

MP: They know exactly what stage you are at.

**I: Good. Great. So how are you feeling generally about the progress you’ve made in the three weeks?**

MP: Very good, very good all the time since I’ve been in here considering what happened, yes very much so. I know it’s a long process but now I’ve got more and more movement in my arm and that that’s helping me completely. That’s helping me a lot because I can do things. At least I can do my upper part.

**I: Yes and you were using your arm weren’t you on the return to pull yourself up.**

MP: Yes.

**I: Good. So you are feeling positive.**

MP: Yes, so I know I can take my shirt off and put a new one on and things like that.

**I: You can take for granted how hard those things are don’t we.**

MP: Yes. I can’t do the buttons up I just have t-shirts at the moment.

**I: Buttons are fiddly. Gosh there’s someone noisy outside interrupting our recording. Just checking on my little schedule of questions. So we’ve talked about that doing things and about your progress. If you had to describe your therapy sessions so if a friend came in who you’ve not seen for a while and you had to describe the therapy you’ve had in a few words what words would you use? It’s a funny question.**

MP: What’s the word. Not educational something similar to that. Educational is one of them, yes because it’s the sort of things that I’ve never done before so it was a new experience. It’s a completely new experience. It’s a worthwhile experience if it happened to someone else the process from start to finish, yes.

**I: Is there anything you would have wanted to be different?**

MP: I don’t think so no. No not at all because they started with the best possible part the arm which is the most important thing to get ready because it’s higher up I suppose. I know the legs do take longer but it’s getting the strength in your arm first and then working downwards. That’s the way I looked at it, that’s what I thought was happening.

**I: What are your next goals for your leg or your next steps.**

MP: Well make sure getting this leg going properly so I can do a few strides. That’s my next goal.

**I: So being able to take some steps.**

MP: Yes.

**I: When you say get it going properly you mean the movement.**

MP: Yes getting the swing right and my balance.

**I: Good.**

MP: I was told every movement to take it slowly. I seem to rush it.

**I: OK.**

MP: Which might be a bit of a hinderance at the moment but I’m trying to slow even doing my exercises now I am slowing them down like my hand exercises and things like that.

**I: I think that’s quite common sometimes people.**

MP: Yes you are rushing to get the end game.

**I: Yes, is that a bit in your nature anyway?**

MP: Yes it is.

**I: I think that’s important to remember we all sort of learn because what you are doing is relearning things and we all learn in different ways as well don’t we.**

MP: Yes definitely.

**I: So it’s adapting to what works for you.**

MP: Yes your mind telling you, right you’ve got to get this done so you can do this and do that.

**I: How are you feeling about going home?**

MP: Oh yes quite excited. I’m just a bit apprehensive that the wife can cope because she’s got a bad back. But we’ll work together.

**I: And you’ll have some support.**

MP: That’s right, yes.

**I: And the therapy team I assume will be coming to see you at home, are you having the rehab team?**

MP: Yes.

**I: Great. That’s lovely. Is there anything else about your therapy that you would want to tell me about?**

MP: I can’t think of anything, no.

**I: Fine. Brilliant.**

MP: I’m quite happy with the process I’m doing, it’s coming on day by day which I’m pleased. If that wasn’t happening then I’d be concerned. But at least I can see some improvements each day.

**I: Yes, which is really important isn’t it even if they’re small as you say a few more seconds or whatever it happens to be a little bit of extra movement seeing it going in the right direction is.**

MP: Even getting in and out of the bed that’s improved since Saturday when we started doing it.

**I: OK, great.**

MP: Rolling over.

**I: What day is it, Tuesday?**

MP: Yes. We weren’t practicing that. I think when they said to me right we’re thinking of letting you out then they said well let’s start getting you in and out of bed so at least you can do that without any help so that helps practicing that. Even today we managed to get out this side quite well. We’ve been practising on the one side.

**I: When you are doing that do you concentrate on anything in particular or is it just trying to do it?**

MP: No just doing it the way I was told, advised to do it. But I noticed each time I do it it’s getting stronger and stronger. Sitting up is getting stronger to get your balance and gripping things.

**I: Great. You’re good, you’re a star pupil. You’ve said doing what I’m told.**

MP: There’s no point in doing it otherwise.

**I: Brilliant. I’ll stop the tape just there.**

**END**

| **INTERVIEW TRANSCRIPTION**  Voice file name: 5D Discharge Interview  Duration: 16 mins 09 secs  Typist comments regarding dictation:  **KEY:**  **I – Interviewer**  MP - Male Participant |
| --- |

**I: So as I was saying I’d like to ask you some questions about your rehabilitation that you’ve been having here particularly the rehab that you’ve been having to focus on your legs so being able to sit, stand, step if you are doing that. So the physiotherapists that you’ve been seeing over on the other ward as well and what you’ve been doing. There’s no right or wrong thing to say it’s just for me to understand what you remember about what you’ve been doing and what you think about it really. Can you just start by telling me a little bit, can you remember how long you’ve been in hospital?**

MP: Roughly I think I’ve been in two weeks. I’m not sure. Somewhere like that.

**I: I think it’s about two to three weeks. When you first came in do you remember much about coming into hospital?**

MP: Yes I did.

**I: So how had the stroke affected you when you first came in?**

MP: Well I couldn’t raise my left hand or left leg and I felt pretty useless.

**I: Oh, yes. Could you move them at all or were they completely weak on that left side?**

MP: Couldn’t move them at all, no.

**I: OK. So were you able to sit up by yourself at that time?**

MP: No.

**I: So you needed help to sit?**

MP: Yes I did. It’s almost like that now, sometimes I manage to sit up but very rarely.

**I: So in the therapy sessions that you’ve been having what have you been working on with the physios.**

MP: A lot with my hand, my left hand, and my leg of course. Having lots of exercises with my hand, picking up different instruments and placing them somewhere else to try and make me use my left hand.

**I: Good. What about your leg, what have you been doing to help with your leg?**

MP: They’ve been taking me on walking things and one day I did quite a long walk. It wasn’t very good but they were happy with it.

**I: Were you having some help to walk?**

MP: Yes. Not all the time, they were right next to me by my side and I had a frame like that in front of me.

**I: OK a Zimmer frame.**

MP: And every now and again they wanted me to do it on my own and they were there just to hold me up. It was good.

**I: So practising some stepping.**

MP: Yes, I did quite a long way.

**I: Good. When you are doing that, when you are practising stepping and walking what do you think about?**

MP: Hard to say. I just think I’m pretty useless.

**I: Does it all feel hard.**

MP: Yes very hard.

**I: Before the stroke did you have no problems with walking?**

MP: Oh no, no. I was a very fit man before that. A lot of people and I might do the same have another stroke don’t they.

**I: Sometimes that can happen, yes, often it doesn’t. Once you are in hospital and you are being treated to try and prevent other strokes.**

MP: Oh right, yes.

**I: Can you remember back to the beginning so you said that actually sitting was difficult at the beginning so were you working on being able to sit by yourself? Do you remember much about what you were doing then?**

MP: No not really.

**I: How about being able to stand up, stand up from a chair or from the bed, have you been working on that?**

MP: Working hard on that at the moment but I can’t quite make it. Some days I will and other days not at all.

**I: So sometimes you can do it by yourself and sometimes you need some help?**

MP: Yes.

**I: When you are practising sitting down and standing up what do you focus on when you do that?**

MP: At the moment I’m focusing mainly on my hand. I want to get that right and then give me more time to concentrate on my leg then.

**I: So if you are in the therapy gym with the physios and you are practising standing up, sitting down, standing up, sitting down is there anything you think about to help you do it?**

MP: No only my own physical strength that’s all. I do try hard. They’re very good, they’re very good. You’ve got some good little gadgets they get you to do and they’re very clever.

**I: Are they. Can you give me an example? Can you describe something.**

MP: They’ve got some cones and they place the cones in different positions and I’ve got to try and get the cones and put them all on top of one, the four cones have all got to be on one. And then the other one is they spread the cones out and they’ve got rings and I have to pick up the ring with my left hand and put it over the cone. They’re very good. Good exercise.

**I: Yes so all to get your hands working.**

MP: A good exercise that.

**I: Do you do any similar things for your leg? Are there any exercises that you are doing to help get your leg stronger?**

MP: No apart from the walking bit.

**I: So more just actually practising the walking bit.**

MP: Yes.

**I: Do you do some walking with the nurses or just with the physios.**

MP: Yes I’ve done some today.

**I: It sounds like you are doing well then.**

MP: I’d like to think so.

**I: Is your walking close to normal for you or is it you’ve still got a way to go?**

MP: I’ve got a long way to go.

**I: Have you?**

MP: I’m afraid.

**I: What needs to improve for it to be better?**

MP: My leg really because I’m right-handed so I can get away with my left hand being but I would like to be able to walk. That’s my main worry. Once I can get up and walk I’ll be well away.

**I: So what needs to change with your leg to make walking easier?**

MP: Just lots of practice I suppose.

**I: Getting stronger.**

MP: Yes, yes.

**I: Sounds like you’ve been working really hard. So when you are perhaps doing some stepping is there anything about the movement that you focus on when you are stepping?**

MP: I’ve got to keep upright and I’ve got to keep my left knee straight. I mustn’t bend my left knee.

**I: OK so your left is your weaker knee.**

MP: Yes. They keep telling me straighten that knee which I do.

**I: So you work on that knee control.**

MP: Yes.

**I: OK that’s good. How do you feel when you are doing all those exercises, how does it feel to you?**

MP: Well I’m so pleased that they’re doing all that for me.

**I: So it’s good to just be doing something?**

MP: Yes, yes. They do lots of exercises where I have to press against them or pull against them and they said that’s good. I’m pretty good at that. That’s about the only thing. Yes that’s good exercise.

**I: Good. Do the physios when they give you instructions about what to do is it always clear what they want you to do?**

MP: Oh very clear, yes. Very clear.

**I: Do you find that their approach is quite similar the different, have you seen some different therapists?**

MP: Yes they’re about the same I think.

**I: OK. How about feedback, how do you know if you are doing something right do they give you feedback?**

MP: They do tell me if I’m doing OK but any particular time they say, oh that was very good, and that sort of thing.

**I: So lots of encouragement?**

MP: Yes lots of that, lots of that.

**I: Is that important to you?**

MP: Yes it is.

**I: When you do get feedback do you find it helpful, does it help you to know.**

MP: It does, it helps me to think I’m getting better.

**I: OK so it helps you to know you are on the right track.**

MP: Yes.

**I: Is the feedback quite specific? Is it about the movement or is it about what to do?**

MP: About what to do really and they tell me to do the things that are important.

**I: These are quite tricky questions, I think sometimes it’s hard to remember isn’t it. It’s hard to remember what goes on so don’t worry. Good. So anything else about how they communicate with you that is good or not good or that you would like to be different?**

MP: It’s all good I think. They’re very good.

**I: Do you feel that you work hard in your therapy sessions?**

MP: Yes.

**I: Do you feel that you are in control of your sessions, do you have some choice about what you’ve been working on?**

MP: Not really.

**I: Or is it guided by the physios.**

MP: I can’t say I’m in control but I’m very happy with what they do.

**I: OK that’s good. Have you been doing any exercises when you are not in therapy?**

MP: Yes when I’m in bed I do lots of it and sitting in the chair I do lots.

**I: So what kind of things have you been doing?**

MP: I can put my arm up there and I’m trying to get this hand, fingers open and I keep kicking my leg out to exercise my knee. All I can do what I can do.

**I: Good. Are they things that the therapists have taught you to do or are they things that you’ve worked out for yourself?**

MP: Well they’re the ones that they do for me so I carry on doing them.

**I: Good. So with your leg in the chair you do things like kicking your leg out do you to straighten up your knee and work your muscles.**

MP: Yes, yes.

**I: So when you do that what are you focusing on when you do that?**

MP: Well I try to get my leg as straight as I possibly can and I’ll keep kicking it and kicking it until I’m tired.

**I: So you persist.**

MP: Yes I do.

**I: You keep going, well done. Is it quite different practising when the therapist isn’t there, do you find it easy to do things by yourself, do you find it hard?**

MP: Fairly easy yes, quite happy with it.

**I: Do you have relatives visit you as well?**

MP: Oh yes, yes.

**I: Do you practice with them, do you show them?**

MP: No.

**I: OK. Generally since you’ve been here in hospital how do you feel about the progress you’ve made so far?**

MP: It’s very small but I know it’s not a quick job. I know it’s going to take some time so really I suppose I’m happy with it.

**I: Everybody would like it to be faster.**

MP: Oh wouldn’t they, yes.

**I: That goes without saying doesn’t it really.**

MP: I imagine I’ll be here for another two weeks at least.

**I: So over the next couple of weeks what do you think the main things are that you’ll be working on. What’s the next goal?**

MP: Well just my arm and leg. I’d like to be able to walk, that’s my main thing. I’m not so much bothered about my arm because I’m right-handed anyway but I would like to be able to walk. As soon as I can do that I’ll be very happy.

**I: Getting in and out of bed by yourself are you having some help still with that?**

MP: I have to have some help, yes.

**I: When the nurses help you in and out of bed do they give you instructions about what to do?**

MP: I can’t think they do specifically.

**I: I wondered if you noticed a difference between when you are doing it with a therapist and when you are doing it with the nurses in how they give you instructions.**

MP: Oh I think the therapists are better. The nurses are very good but the therapists are good, they make you do it.

**I: So what makes it different?**

MP: Well when I’m under the therapists I feel I’m getting the real treatment. Although the nurses are good I feel when I’m under the therapists I really feel it’s good things, good practice. They are really persistent, they work hard on me. I told them one day you are cruel.

**I: They have a bit of a reputation for that. Cruel to be kind.**

MP: Yes.

**I: So that’s interesting it does feel a bit different when you are practising with them.**

MP: Yes, oh yes.

**I: Does it feel like you work harder or that you are working more specifically on things?**

MP: I think I work harder. I’ve got to for them.

**I: You feel you work a bit harder when you work with the therapists.**

MP: Yes.

**I: We’ve nearly finished with my questions anyway. So over the next few weeks you feel you are going to be working on your walking.**

MP: Yes.

**I: And what is it you need to be able to do to make that walking better do you think?**

MP: Lots of practice. I certainly won’t sitting on that bed, I’ve got to get up.

**I: This is true.**

MP: I’ve got to get up as many times as I can.

**I: Yes. Is there any particular thing about your walking that needs to improve or is it just practice do you think?**

MP: When I’m trying to walk they tell me to get my left foot forward more which I do try to do. In fact at the moment I sort of drag it along and they ask me to bring it forward more.

**I: Bring your leg forward.**

MP: Work harder on it.

**I: OK. That’s good. So if you were telling your family about your therapy here and you had to choose two or three words to describe it what words would you use? It’s a tricky one, people have to think about that.**

MP: I would say the therapy was very good and very helpful. That’s about all I could say.

**I: So you feel positive about it.**

MP: Oh yes.

**I: Steady progress. That’s fine. Anything else you would like to tell me about what you’ve been doing?**

MP: I don’t think so, no. I think you’ve asked me all the questions.

**I: That’s good.**

MP: I hope I’ve been helpful.

**I: You have been helpful.**

MP: Oh good. They do videos now when I go to the gym they video it all.

**I: Yes and you are happy with that, so we use the videos just to see what you’ve been doing and to compare different sites.**

**END**

| **INTERVIEW TRANSCRIPTION**  Voice file name: 5FDischarge Interview  Duration: 12 mins 56 secs  Typist comments regarding dictation:  **KEY:**  **I – Interviewer**  MP - Male Participant |
| --- |

**I: So can you just tell me a little bit about how long you’ve been in hospital. Do you remember when you came in?**

MP: About three weeks.

**I: About three weeks now.**

MP: Three to four weeks. Beginning of September.

**I: From the beginning of September.**

MP: Or just after.

**I: Yes, OK. Can you remember back to when you came into hospital what was it like, how had the stroke affected you then?**

MP: I didn’t realise how bad it was after I got here.

**I: OK.**

MP: Because it carried on working after I got here. It didn’t stop there it carried on.

**I: Getting worse do you mean? Yes, OK. So the stroke had it affected the left side of your body?**

MP: Yes.

**I: Do you remember what you were able to do or what you were not able to do back then?**

MP: I can’t really remember.

**I: No. It can be a bit of a blur maybe.**

MP: Yes. It’s certainly progressive since getting better.

**I: Do you remember how you were getting out of bed for example when you were getting from bed to chair. Were you having help with that?**

MP: I was. I need to because I kept collapsing on the left hand side. I still do. Awful feeling that. That’s what they were working on today the balance and stretching out and working things out.

**I: So in your therapy sessions you’ve been working on your sitting balance, is that right? Yep. And what else have you been working on in therapy?**

MP: I think.

**I: Have you been working on doing any standing yet?**

MP: Yes.

**I: OK.**

MP: They tried to get me on the last bit today to stand on there but it’s very difficult.

**I: Standing on your own but was very difficult.**

MP: Very difficult.

**I: So if you think about first you working on your sitting you said you tend to lean over to the left do you and you need to work on sitting in the middle. Is that right?**

MP: Yes, she went over to the right to help me.

**I: So when you are working on sitting what sorts of things are you thinking about?**

MP: Thinking about doing it at the time.

**I: OK.**

MP: To get better. [*4 mins 5 secs*] move my fingers on the left hand.

**I: Oh great.**

MP: First time.

**I: So when you said you were thinking about what you were doing when you are sitting can you explain a bit more about that. What is it you try to concentrate on?**

MP: Well [*4 mins 37 secs*]. I live on my own you see.

**I: OK.**

MP: But I should do by the time they’ve finished with me.

**I: Yes. Thinking about managing at home. Is there anything about the movement that you focus on?**

MP: All of it really.

**I: Yes focusing on all of it but not a specific thing you think oh I really need to be able to do this or be able to do that.**

MP: Not yet.

**I: No, OK. How about the standing, so you’ve started to do some standing so going from sit to stand. Is there anything whilst you are doing that that you concentrate or focus on?**

MP: They hoist you with the mechanical.

**I: They hoist you with the mechanical hoist, yes. When you are down in your therapy session though have you been trying to do some standing without the hoist? OK so they use a standing hoist.**

MP: The physio [*5 mins 57 secs*] standing hoist.

**I: Yes use the standing hoist. OK.**

MP: Tried that but I couldn’t I was too tired by then.

**I: OK. Sounds like that’s quite hard at the moment.**

MP: Yes, it was this morning.

**I: They’ve worn you out today haven’t they. How about your leg movements more generally? How much are you able to move your leg?**

MP: My right leg is alright but my left leg is no good.

**I: No.**

MP: Not yet.

**I: Can you feel your leg?**

MP: Yes.

**I: So it’s difficult to move but you can feel. When you are in your therapy sessions is there anything about the way that the therapists give you instructions or give you feedback that you think is good or that you would like to be different?**

MP: All good.

**I: It’s all good.**

MP: Yes, they’re very helpful. They try to help me. I do appreciate what they’re doing. [*7 mins 33 secs*] hard work.

**I: Yes. [interruption about lunch]. That’s your lunch ready so we won’t be too much longer. What was I just going to ask? Yes about how they are giving you instructions and feedback. Is it always clear what they want you to do?**

MP: Yes very clear.

**I: You find their instructions helpful?**

MP: Yes. [*8 mins 10 secs*].

**I: Sorry say that again?**

MP: [*8 mins 21 secs*] put them into operation.

**I: It’s you putting the instructions into operation, yes. OK.**

MP: You can only [*8 mins 31 secs*] so much. They’re very good.

**I: When they give you feedback about what you are doing do you find that helpful?**

MP: Yes.

**I: In what way is it helpful?**

MP: They tell you when you are doing well a lot. They’ve always been very positive with me. It’s helpful.

**I: So it’s helpful because they’re positive and they tell you whether you are doing it well. Do they tell you if you’re not doing it well?**

MP: I haven’t done that at all.

**I: No, OK.**

MP: Thank goodness.

**I: You’ve been a star student have you? Good.**

MP: [*9 mins 15 secs*]

**I: Wow you must be doing something right. That’s fine.**

MP: They’re videoing the sessions.

**I: They’re videoing them, yes they are so we can have a look at what you are doing as well for the research. Do you tend to see the same therapist each time or have you seen lots of different people?**

MP: Mostly the same time and do see different ones.

**I: Are they quite similar in their approach?**

MP: Yes.

**I: When you see different people. Yes. So you feel they’re all working towards the same.**

MP: Working towards the same thing.

**I: Yes OK. Good. Have they given you any exercises for you to do when you are not in therapy, is there anything they’ve asked you to work on here?**

MP: On my arm.

**I: Mainly my arm.**

MP: Massaging it, getting it you know.

**I: Have they given you any exercises to do for your leg or is it just for your arm?**

MP: No.

**I: Just for your arm, yes, OK that’s fine. So just more generally how do you feel about the progress you’ve made since you’ve been here in the last three weeks or so?**

MP: Considering the severity of the stroke I think I’ve made good progress.

**I: Good.**

MP: Through them.

**I: So you feel positive generally about your progress.**

MP: Yes.

**I: And realise that you had a severe stroke.**

MP: It’s a case of working through it to get myself better. Sorry I’m not speaking so well.

**I: That’s alright you are tired. You are absolutely fine. Good so just finally my last two questions really. So if you had some friends or family or somebody come to visit you here how would you describe your rehab sessions to them. What words would you use to describe your therapy sessions?**

MP: Very good. It’s up to me to work with them.

**I: Yes, up to you to work with them. Yes.**

MP: For my own good.

**I: Yes.**

MP: They’re very good.

**I: OK. Is there anything about those sessions that you would want to be different? No.**

MP: No.

**I: No, OK. That’s it unless there’s anything else about your therapy that you’ve been having here that you want to tell me about?**

MP: Not really. They’re doing their best for me. Lovely girls. Really helpful. It’s up to me to work with them to get there because I am progressing in that direction.

**I: Yes. Good. Sounds like you are doing well, slow and steady wins the race doesn’t it. Thank you for talking to me about that.**

**END**

| **INTERVIEW TRANSCRIPTION**  Voice file name: 5G Interview  Duration: 15 mins 04 secs  Typist comments regarding dictation:  **KEY:**  **I – Interviewer**  FP - Female Participant |
| --- |

**I: So as I said I’m just going to be asking you a few questions about the rehab you’ve been receiving since you’ve been here and I know you’ve only been in hospital for quite a short amount of time and particularly about the therapy you’ve been having that’s been focusing on your leg, so anything to do with sitting, standing, I’ll get you to tell me a little bit about what you’ve been doing with regards to your recovery for your leg. But just to start me off can you just tell me a little bit about what it was like when you came into hospital and how you’d been affected by this stroke.**

FP: Very scary. All of a sudden I was fine one minute and the next minute I wasn’t very well and I didn’t call anybody until the morning.

**I: OK. It was your right side of your body.**

FP: On my right side has been affected.

**I: And can you describe how it was affected?**

FP: I have no feeling. I can’t move anything. Or I couldn’t.

**I: Was anything else affected or was it?**

FP: My speech and to learn how to swallow again.

**I: That must have been really frightening.**

FP: It was.

**I: When you first came in in those first few days what were you able to do in terms of moving around, did you need help and equipment?**

FP: I needed help. I couldn’t walk. I could stand but I couldn’t walk.

**I: OK. How did you get from bed to chair those first few days?**

FP: They used these.

**I: OK so a return.**

FP: Yes return.

**I: So thinking about your leg in particular but you generally as well what sorts of things have you been working towards with the physios in your therapy sessions?**

FP: Getting home again. That’s the goal but more steps.

**I: So what are the steps you are working to achieve to be able to go home?**

FP: Get my right side stronger so that I can walk. I can take my weight and I can walk.

**I: Can you tell me a little bit about how you’ve been doing that, what sorts of things do you do in your therapy sessions?**

FP: Well kicked a football. Walked around the table. Tried to bend my knees and straighten them. Table with markers on it and I had to touch it with my hand, my good hand.

**I: OK. When you are doing those sorts of exercises which are working towards getting your legs stronger aren’t they and working on balance and things.**

FP: Hopefully.

**I: When you are doing them what sort of things are you thinking about?**

FP: Just trying to do what I’ve been asked.

**I: Doing what you’ve been told.**

FP: Yes.

**I: Can you give any examples of the sorts of ways that the physios give you instructions. What sort of things do they ask you to do?**

FP: They tell me what I have to do, vocal, which is better for me because I’m not very good at prompting. So vocal commands are better for me so I know what they want me to do.

**I: Is it always clear to you what they want you to do?**

FP: Yes.

**I: It makes sense?**

FP: Very clear. Makes sense.

**I: That’s good. Is it always clear to you why they are asking you to do certain things, what you are working on?**

FP: Yes, yes it is.

**I: Is there anything that’s been particularly difficult for you in those sessions. Are there things that you are finding hard?**

FP: No I don’t think so.

**I: Are the exercises they ask you to do quite challenging or do you find them easy?**

FP: Yes sometimes challenging, sometimes easy.

**I: OK. So a little bit varied.**

FP: Yes.

**I: Are you still using the return here at the moment?**

FP: At the moment.

**I: That’s why it’s next to you.**

FP: Yes. I’m the only one that’s going to use it because I can stand. I can’t put my weight on it yet but I can stand using my left leg.

**I: Great, that sounds like you are doing really well. When you are in your physio sessions with the physios do you practice some standing not with the return?**

FP: Yes they use a walker.

**I: OK.**

FP: And I shoe shuffle around it to turn around.

**I: So like a Zimmer frame.**

FP: Yes.

**I: What are the things you think you need to get better at to be able to improve how you do that?**

FP: Get my balance right. And be steady on my feet.

**I: Yes. How do you feel on your feet at the moment?**

FP: Unsteady, wobbly.

**I: Wobbly, OK. So you are working towards being able to do that a little bit more independently in order to go home, yes?**

FP: Yes hopefully.

**I: When you are standing up so going from sitting to standing is there anything about that movement that you focus on when you are doing that?**

FP: Right hand keeps slipping off and I can use my left hand to do everything.

**I: OK. Do you mean when you are pushing up from the chair your hand slips off or on the Zimmer frame do you mean?**

FP: On the Zimmer frame because I’ve got to [*6 mins 27 secs*] keeps coming off. I’ve got no grip at the minute. They’re working on that.

**I: Yes, OK. So when you are doing those types of movements is there anything you have to concentrate on particularly or do you just give it a go?**

FP: I just give it a go. Give it a real good try. You don’t know what you can do until you do it.

**I: No that is true. Are there any things that you’ve worked out that help you. I know it’s quite early days in your rehab but anything that you think works for you in particular?**

FP: A Zimmer frame. But that one is easier at the minute.

**I: Makes you feel steadier does it?**

FP: Yes makes me feel steadier. The Zimmer frame I’m unsteady on at the minute.

**I: Do you tend to have the same physio or have you seen lots of different people.**

FP: Same physio I think.

**I: OK. If you think about how they work with you in your therapy sessions how would you describe their approach?**

FP: Yes good.

**I: What makes it good, what’s good about it?**

FP: They tell me what to do and they say if I’ve done badly or I haven’t. But so far I’ve done good they keep saying. You have to be positive.

**I: So do you find that feedback helpful?**

FP: Yes.

**I: Do they give you any specific feedback about how you are progressing. How do you know if you are getting better?**

FP: I can walk a little bit but I can’t walk unaided yet. That’s what we’re working for.

**I: So you can see the improvements in what you are doing gradually.**

FP: Yes.

**I: When you are in your therapy sessions and you are working hard how do you feel?**

FP: Very positive.

**I: Good. Is there anything that you would like to be different about your therapy?**

FP: Not at the minute no.

**I: You’re quite happy with everything.**

FP: I’m quite happy, yes.

**I: OK. If you think a little bit about how the therapists you see how they give you instructions and feedback is there anything about that that’s particularly helpful or anything that you find more challenging?**

FP: Helpful.

**I: OK. I’m just checking through my questions here. So always quite clear what they want you to do.** **Yes.**

FP: Yes.

**I: Can you give any examples of the types of instructions they give you? Can you remember.**

FP: They ask me to kick a ball with my left leg then do it with my right leg. They tell me what I do and I try and do it.

**I: You mentioned for some of the exercises they were using markers and things on the table.**

FP: Yes that’s difficult because I can’t see.

**I: Of course.**

FP: That’s the only thing, that’s the problem my eyesight.

**I: I was going to ask you do you find it helpful having those sorts of targets but obviously that’s more difficult for you. Have they used.**

FP: I can see things on the table but I can’t see things on the floor. They have to use something white on the floor for me to kick.

**I: OK.**

FP: It can be adapted for me.

**I: Good. So having those sorts of targets or objects to aim for do you find that’s helpful to get the movement?**

FP: Yes.

**I: Are you doing any exercises when you are not in therapy, have the therapists asked you to practice anything by yourself?**

FP: Yes they do.

**I: What have you been doing?**

FP: I’ve been practising kicking but I’ve got nothing, I haven’t got a ball to kick.

**I: Oh OK. So you are just kicking into the air.**

FP: Yes. And they get me to, yesterday they got me to lie on the bed and try to bend my knees up and sideways up.

**I: Would it be easier do you think to practice the kicking if you did have something to aim for?**

FP: Maybe but I wouldn’t be able to achieve it because it would roll away and I wouldn’t be able to get it.

**I: Yes.**

FP: That’s difficult. You can’t get the nurses to do it because they’re busy.

**I: Not a ball necessarily but, yes, something you could feel if you were getting there maybe.**

FP: Yes.

**I: When you are doing those exercises by yourself like in the chair how do you know if you are doing them in the right way? Do you have a way of knowing that?**

FP: I don’t know really I just do what they tell me and then I try and do it right I suppose.

**I: OK. When you are doing something like the kicking exercise or the exercises in bed what do you focus on then? What do you think about?**

FP: Trying to do what I’m told to do.

**I: If only everyone was like you, doing what you’re told to do.**

FP: Yes you’ve got some that won’t do it but if they don’t do it you are never going to get better and you want to get better.

**I: Yes. Do you count how many you do, do you try and practice lots?**

FP: Try, yes. But it’s difficult with my pumps on.

**I: Yes it can feel a bit restrictive can’t they.**

FP: Yes can be, they stick together and that makes it harder.

**I: Once you get a bit more mobile you’ll be able to ditch those.**

FP: Hopefully.

**I: So that’s a good thing to look forward to isn’t it.**

FP: Yes.

**I: Just in the time that you’ve been here how do you feel about the progress that you’ve made so far?**

FP: A little bit, quite a bit I think. Hopefully I’ll make some more improvements.

**I: You feel it’s going in the right direction.**

FP: At the minute. There are going to be setbacks I know that but hopefully at the minute it’s going forward. You’ve got to be positive with these things.

**I: It definitely helps doesn’t it.**

FP: Yes.

**I: Do you feel that when you are with your therapy and your rehab do you have much choice over what you do in your therapy sessions – do you feel that you’ve got some control over what you are doing?**

FP: I do what I’m told so I can get better. I know they’ve got my interests in getting better.

**I: So you are happy with that?**

FP: I’m happy.

**I: So my last few questions really, so if you were telling a friend or someone came to visit here in hospital and you were telling them about your physio sessions what words would you use to describe the therapy?**

FP: It’s very good I guess. You need to get therapy to get walking. Took first steps [*14 mins 41 secs*] hopefully I’m going to get much better at it.

**I: Yes, OK. Is there anything else about your therapy and rehab that you would like to tell me about?**

FP: I don’t’ think so.

**I: No, OK, that’s lovely, thank you.**

FP: That’s alright.

**I: I’m going to turn these recorders off now. END**

| **INTERVIEW TRANSCRIPTION**  Voice file name: 6A Discharge Interview  Duration: 22 mins 56 secs  Typist comments regarding dictation:  **KEY:**  **I – Interviewer**  MP – Male Participant |
| --- |

**I: So as I said if it’s OK I want to just ask you some questions about the rehab you’ve been having in the last couple of weeks that you’ve been in hospital. I’m particularly interested in talking about the therapy you’ve been having for the recovery of your leg movement, so your being able to stand, step and walk and what you’ve been doing with the physios. So just to start for me could you just tell me a little bit when you first came into hospital can you remember what it was like in terms of what you were able to do and not do?**

MP: I couldn’t walk.

**I: You couldn’t walk, OK. Was your leg completely weak?**

MP: Yes it was like dragging along behind, if I tried to walk it would drag along behind me.

**I: OK. Has your arm been affected as well?**

MP: Yes.

**I: What about the feeling in your leg, were you able to feel it?**

MP: You could feel your leg, yes.

**I: Yes you knew what it was, yes, just weak.**

MP: Well I don’t know that you knew what it was doing because you’d look round and it would be over there.

**I: OK.**

MP: But you could feel it.

**I: You could feel if someone was touching you.**

MP: Touching it. yes.

**I: So when you first came into hospital in those first few days how were you getting from bed to chair? Did you have help, were you able to do it?**

MP: No you have help. They won’t let you do it.

**I: So help from people or help from a machine because everyone is different.**

MP: Both sort of thing. I’ve got the thing, what are they called?

FP: Steady.

MP: Steady. So they chuck you into the steady and then into the chair because it goes in backwards doesn’t it.

**I: Yes.**

MP: So that’s the way they do that.

**I: Were you able to sit up by yourself when you first came in.**

MP: Yes.

**I: So you were able to sit but not to walk properly.**

MP: No.

**I: I’m just getting an idea because everyone is affected very differently. So thinking about what you’ve been doing in your physio sessions can you just tell me a little bit about what you’ve been working on in physiotherapy? It’s not a test. What have you been doing?**

MP: All sorts of things.

**I: Give me some examples.**

MP: Standing up, trying to get me to stand up. Then walking, then walking along a line to get it neater. Then not really so much balance because that will just come. We’ve just recently been doing little things to try and get my arm and hand working which I have got movement in now thank God.

**I: That’s good, really good.**

MP: Just things like that really.

**I: Did you set goals with the therapists, things that you wanted to achieve.**

MP: Yes but they’re unachievable this year.

**I: OK what were they?**

MP: Going to play golf, so that’s a minimum 5k walk.

**I: But a long-term goal.**

MP: Yes it will be. No way I can grip the golf club, no way, so that’s just gone out the window now. Well.

**I: For today.**

MP: Obviously to get out of here, to walk out of here which is not a problem. Basically that. And just get back home and do stuff.

**I: OK. So you said that you’d been working on being able to stand and then walking, can you describe any specific exercises the therapists were doing with you to help with that?**

MP: Yes, you had to get your legs moving because your weak one really didn’t want to do anything. Showing you wouldn’t be no good would it.

**I: No. Can you describe it. Something lying on the bed?**

MP: Yes what I would do is because you’ve got a lot of time in here of doing nothing so I would lie on my bed at night and what have you and pull my strong leg up but not vertically to the ceiling, the knee up to the ceiling so it would be bent at 90 degrees with my foot on the bed and bring the weak leg at the same foot on the bed, the same sort of angle, and bring that knee up to touch that one.

**I: OK, yes I know what you mean.**

MP: And then I’d also lie in the bed and move my ankle so that my toes pointed to the ceiling. Well actually on both legs but more on the weak one. That’s about it for getting to walk.

**I: Were they things that you worked out yourself you needed to do or were they things that you were shown to do?**

MP: They showed me all of this. But I’d thought right just do them, I want to get out of here.

**I: So you’ve been practicing, you’ve been really motivated. Good for you. When you do those types of exercises that you’ve described what are you thinking about, what do you focus on?**

MP: Just do them.

**I: Just getting the exercise done.**

MP: Move that leg.

**I: So that’s what I’m thinking, do you think about your leg?**

MP: Yes, you’ve got to. It’s hard to explain but you [*6 mins 31 secs*] you literally forgotten how to move your body, it’s just gone. It’s gone you don’t know how to do it or your brain doesn’t. I mean you imagine you can do it but you just can’t. You can’t control your muscles, that’s basically it. Just the tiniest movement takes some concentration.

**I: Yes, OK, so you really try and concentrate on it and focus on it.**

MP: Yes.

**I: Has that got easier over the last couple of weeks or not? No?**

MP: Not with my hand no but we’ve only really just started on the hand over the last few days. I just do, well it’s no good for that is it, yes I’ve got that but they say.

**I: Electrical? No?**

MP: No I’ve not had that. I was going to but I haven’t.

**I: Oh it’s a beanbag.**

MP: All I’ve got to do is because I can’t, this is really hard.

**I: Ah well done. For the tape that is picking up a bean bag and putting it in a cone but you did it.**

MP: Just making ice-cream.

**I: You’re making ice-cream.**

FP: That hand couldn’t do anything, could it.

**I: OK. That’s really good.**

MP: I’ve been sitting at night and just trying to move my thumb in and out which is getting there but not brilliant.

**I: I notice that you are looking at your hand so do you find that helps you so you want to really look at it and concentrate.**

MP: Concentrate on it.

**I: Was that the same with your leg as well although it’s harder to look at your leg.**

MP: No, not really. Well lying down you would you look down and see, make sure it’s all going together and that because as I say you didn’t know where that leg was.

**I: So if you are not looking at it it’s hard to know if you are doing the right thing.**

MP: Exactly, yes.

**I: In terms of your leg recovery is there anything that’s been particularly difficult – I know it’s all challenging but is there anything that was really difficult for you? Any type of activities or exercises?**

MP: Yes there was one which we only did yesterday actually as part of your test, standing on one leg.

**I: Oh OK. In what way was it hard?**

MP: I can’t do it. I kept trying, I think I tried three times but couldn’t do it.

**I: Couldn’t do it. Leg not strong enough or you just felt you were going to fall over or?**

MP: I don’t think the leg is strong enough.

**I: Not yet, OK.**

MP: And you start wobbling and then you’ve got to hold on to something.

**I: So in a good few weeks’ time when you can stand on one leg remember this conversation that you couldn’t do and things are progressing.**

MP: I will.

**I: With your leg was your leg weak throughout the whole leg or was it weak particularly at one point? Sometimes people have more weakness in the ankle or the knee.**

MP: No it’s the whole leg.

**I: The whole leg. OK. So you’ve had to work on strengthening the whole leg?**

MP: Well yes.

**I: You’ve talked about concentrating and really focusing on the leg, is there anything else that you’ve found works to help with your performance?**

MP: You’ve just got to think do it. You want to go and get back to your life and you’ve just got to have the willpower to do that.

FP: He’s very positive.

**I: It makes a big difference, it makes a really big differences. It’s tough isn’t it. It’s tough. If you just think about the therapist, have you worked with one or two therapists or lots of different physios? Have you had various?**

MP: I’ve had mainly two. When I first came in I had two different ones and they were just trying to loosen up my limbs in case they, I suppose the muscles just didn’t work at all, just went to concrete is what I feel. So they would just limber up my hands and my leg and what have you.

**I: So they would move it for you and give you some stretches.**

MP: Yes, yes, just like stretch it about. Then I had after that they said about doing this and then I had my two proper physios and just gone from there really.

**I: Is there anything about the way the physios have worked with you that you’ve, how would you describe their approach with you?**

MP: Brilliant.

**I: What makes it brilliant?**

MP: Just the way, or when you can’t walk and you suddenly can you are very happy.

**I: When they’re working with you in therapy do they give you, can you describe how they, I’m trying to think how to word it. How do they tell you what to do, how do they give you instructions? Anything about how they talk to you that you noticed?**

MP: Very nice. You’d have to watch the videos.

**I: Is it always obvious what they want you to do? Do you think it’s clear what you need to do? I know these might seem like odd questions but there’s a method in there.**

MP: I know where you are coming from. Once you’ve done a couple you can kind of figure out where they’re going. Well you can figure out where they’re going. Yes but obviously they tell you that leg is going out too far try and pull it into that line or something like that, you know what I mean. Things like that.

**I: Do you feel they gave you the right amount of instruction to help you to practice?**

MP: Yes definitely.

**I: Not too much, not too little.**

MP: Honestly I think it’s all about you wanting to do it. The first couple I had not in this room but say like here I can’t remember, oh yes some circles on the floor and just tap your foot across, your toes – that was extremely difficult. But he left them with me and said I can practice it myself.

**I: Brilliant.**

MP: And I think that’s helped a lot because I’ve tried doing everything I can to continue with what they’ve taught me.

**I: And that’s really important to help with your recovery.**

MP: Just it’s not happening.

**I: And that’s really helpful isn’t it if they’ve left you with the something so you know what to do, exactly what to do and you know if you are doing it right or not.**

MP: Yes.

**I: You found that helpful. What about feedback as you practice things with them whatever that is, did they give you much feedback about how you were doing it?**

MP: Yes they always that’s brilliant.

**I: Would they give more detailed feedback so other than the encouragement would they give feedback about how you were moving?**

MP: Yes like if I was not lifting my foot properly like if it was dragging a little bit on the ground when you are walking lift it up more, things like that. When you first start it’s not pretty. It’s not dainty, it’s not anything and actually it’s still not brilliant now but it’s getting better, it’s just practice.

**I: So what are you doing now, are you walking with some help, walking by yourself?**

MP: Walk on my own now.

**I: It’s in big letters above your head. You are walking by yourself. Brilliant. So are you going out to the bathroom, you are walking in the wards?**

MP: I am now.

**I: Gosh so you’ve come a really long way in a couple of weeks, haven’t you. When you are doing that and you’re walking are you concentrating then or is it becoming.**

MP: [makes a scoffing noise].

**I: You’re still concentrating a lot.**

MP: Mentally you’ve got to otherwise it all just goes and you’ll lose your balance. Well you just stumble but again the stumbling is still building up the muscle because it’s contracting to hold you up. So I’m not too worried about that it’s just that you don’t want to do it and you want to walk the way you remember yourself walking. You can’t.

**I: So if you were to walk out to the toilet or something now what would you be concentrating on? Just not falling or?**

MP: No, no. At one point because they’ve got square tiles I was looking down which you shouldn’t do because no one looks down do they when they’re walking.

**I: No not unless it’s a very uneven floor.**

MP: And following the line of the tiles. Like also I was trying not to step too far and rushing. So I just try and do one foot at a time and the tiles were a foot square so you know what a foot was. So I’d try to go one tile, two tiles like that, three/four along the line.

**I: Yes so you are using the tiles.**

MP: But again that’s looking down so then I pick something in the corridor in the distance and just focus on getting to that.

**I: Yes, OK. So you are really concentrating but you are actually not really concentrating on your leg you are concentrating on.**

MP: Yes you are, then you are concentrating because you’re thinking right, well I am and everyone is different, and I’m thinking this must look terrible. Pick that leg up properly and put it down nicely and that takes some, along with looking in a straight line in front of you.

**I: Yes, OK.**

MP: And then if there’s objects or people coming that really distracts you and you’ll stumble, well I will, stumble a little bit because you’ve just lost most of your concentration.

**I: What’s the next thing that you are working on in terms of your walking? What’s the next goal?**

MP: 5k.

**I: OK. 5k walk?**

MP: On a golf course with a bag on my back.

**I: Up and down a golf course.**

MP: Yes. It won’t happen this year, or I don’t think it will.

FP: It’s a goal though isn’t it.

MP: But today she said, they’ve got a little set of stairs so because I’ve got some steps at my house I said come on we’ll go and do that just make sure I can get up there safely. So we did that and she said we’ll go for a walk. So we came out of here, what’s that, say from the gym 20m, 30 maybe, all the way down to the end that’s another 30, through the doors which are controlled so you can’t get through them and all the way down to the far end of the hospital, it’s got to be half a kilometre. All the way down there.

**I: Yes they’re long corridors aren’t they.**

MP: I don’t know whether it is half a kilometre.

FP: Feels like it when you’re walking it.

MP: All the way down to the end and all the way back to here.

**I: Well that’s got to be a couple of.**

MP: I’d have said it was near on.

**I: A couple of tees on a golf course.**

MP: Yes it would be, yes it would be one, it would be two holes, yes.

FP: Surely that, does that take it, it must take it out of you mentally as well that walking.

**I: Yes.**

FP: When you’ve had a stroke.

MP: That’s why I’m going mad.

FP: Tiring.

**I: Yes it’s tiring physically and it can be tiring psychologically as well.**

MP: No physically I’m not bothered about that.

**I: Are you not. Just because your brain is working a lot harder to do that than it normally would but like you said yourself there’s stuff going on in the corridor around you and normally we process all of that without even thinking about it and you are having to be a bit more aware.**

MP: You have to think about it.

**I: So then that is tiring.**

MP: Literally trying to keep going straight and as nicely as you can otherwise you.

**I: Yes it’s lots of different things. OK. Great. So generally I’ve probably got a bit of an impression of this already but how do you feel about the progress you’ve made since you’ve been here?**

MP: Brilliant. Well happy.

**I: Glad to be having an end point to going home on Thursday. Are you having some more therapy when you go home? Yes. So it carries on. Yes, OK. So last few questions if that’s alright. These might again seem a bit odd but if you were telling your family or somebody else about your therapy sessions how would you describe them?**

MP: As in what context?

**I: Just how would you describe them in a few words? What’s the first thing that comes to mind?**

MP: You kind of think, well tip that and you think well that’s a load of rubbish that’s not teaching me anything but it is.

**I: Yes.**

MP: But that’s kind of what I think.

**I: So it’s important to understand the reasons for the things you are doing.**

MP: Just like them little taps of your toes you think what? You think oh I want to kick a football properly like you used to be able. No it doesn’t work that way. It’s things like that you just think that’s not going to work but it does, you’ve just got to go through it haven’t you.

**I: Yes, OK. When you are practicing things in therapy how do you feel?**

MP: What do you mean?

**I: When you are actually off with the therapist doing something that might be quite hard how do you feel?**

MP: With the hard ones or some of them like one of them will go, he just showed me what he wanted he me to do and then he said we’ll do five and I so I did the five and then he said you know when you did the five I’m going to make it harder I said yes I expect so. He said do ten when I’d done the five and I said no do 20.

**I: I think I’ve got a good measure of you. Fine. That is all of my questions unless there’s anything else you want to tell me about what you’ve been doing. Or anything you want to ask me about the research you’ve been doing?**

MP: No that’s alright.

**I: I’ll turn this off.**

**END**

| **INTERVIEW TRANSCRIPTION**  Voice file name: 6B Discharge Interview  Duration: 17 mins 07 secs  Typist comments regarding dictation:  **KEY:**  **I – Interviewer**  MP – Male Participant |
| --- |

**I: So is it OK just to start. Well actually I’ll tell you what I want to talk to you about a little bit, so we’re going to have a talk about your therapy particularly physiotherapy and particularly what you’ve been doing that’s been focusing on your leg recovery, so sitting, standing, stepping, walking. Do you need some water? Do you want a drink?**

MP: Thank you.

**I: It’s dry isn’t it.**

FP: The air in the hospital it’s really dry, I always go out of here thinking I need a drink.

MP: Bet you do.

**I: It feels like the heating is on, it’s warm. So we’re going to talk about that and the reason that we’re doing that is we’ve got as I said eight different stroke units taking part in the study so we’re going to interview people in all eight of them and it’s just to really understand whether your feelings about the therapy you’ve got are similar to those across the eight different stroke units. So there’s not any right or wrong thing to say and it’s not a feedback really about the therapists themselves whether you think they’re good or not although you are free to tell me whatever you like, it’s more about what you’ve actually been doing in your therapy.**

MP: [Coughing] timing is perfect.

**I: You need some more water?**

MP: No I think that’s OK now.

**I: Are you sure? It’s annoying isn’t it when you’ve got a tickle. Just tell me a little bit about how long you’ve been in hospital, what happened to you?**

MP: OK. So I had a stroke on, can you remember the date?

FP: 14^th^.

MP: 14^th^ January.

**I: OK so you’ve been here about three weeks?**

MP: Four weeks now.

**I: Four weeks, yes.**

MP: Progress has been slow but positive. I’ve found the physiotherapy unit in particular with their structure has been very effective and it’s a bit of a lifeline really because it keeps you going knowing that you’ve got another session.

**I: OK so when you say structure what do you mean?**

MP: Well as opposed to just sitting in bed waiting to get better. It’s something proactive that’s getting you there.

**I: Yes, OK.**

MP: It makes a big difference being able to look forward to something that you know is going to help and each session, at the end of each session you do feel as though you’ve achieved something.

**I: Good. So when you first came into hospital how had the stroke affected you?**

MP: Paralysis on the left side leg and arm. Speech slurred which it still is very slightly in as much as I think it sounds OK but I have to think about it a bit more than I ever used to. Apart from that not too much fortunately.

**I: Were you able to move your arm and leg at all, did you have some movement or complete paralysis?**

MP: No, I could feel it, I had sensation but I didn’t have anything drop.

**I: OK. So can you tell me a little bit about what you’ve been doing in those therapy sessions, physio sessions?**

MP: Initially they explained to me where I was at and what they were going to try and achieve which is helpful. Then a number of sessions working on my leg using things like skateboards to just try and get some movement back be it ever so slightly, it was just to show me that I had some control.

**I: And when you said they explained to where you are, or your words were, explained where you were at, can you remember what they said?**

MP: Yes just explained what had happened and what it was likely to mean to me personally.

**I: OK, yes.**

MP: And where I was likely to get to or where it might be possible to get to and how they were going to try and achieve that. That makes a big difference just having that focus and goal.

**I: So what were your goals at the beginning and your goals now?**

MP: Just to get back to where I was before the stroke really. I wasn’t expecting any improvement anyway just to recover what I had before.

**I: So when you first came into hospital were you able to sit by yourself or had the stroke affected your body?**

MP: That was a surprise the first time I sat on the edge of the bed which sounds simple enough but I was all over the place.

**I: Were you? You were wobbly.**

MP: Jelly on a plate, yes.

**I: OK so you had to start with working on.**

MP: Start with working on your core muscles just to be able to sit up right.

**I: And can you describe to me any of the specifics, you mentioned a skateboard and the specific exercises you’ve been doing in therapy?**

MP: Well quite a lot of supported actions. So they would support my arm at the elbow and support my hand by keeping it up and then it was up to me to move it backwards and forwards so it’s like a rocking motion. It at least gives you the feeling that you are doing something with it even though it was in my head quite lightweight I wasn’t particularly achieving much, I couldn’t have clapped or reached out and picked something up. But at least you could see that there was some control there.

**I: With your leg what kinds of things were you doing initially or now even, what kind of things are you doing now?**

MP: Now I’m able to stand up unaided from a seated position.

**I: That’s good.**

MP: And put the weight on my leg for five minutes or more. I think in the last session I was timed at five minutes with weight equalled on both feet.

**I: How do you know if the weight is equal on both feet?**

MP: We did one session where I stood on two sets of electronic scales and we had to try and keep the readings similar on both, which was a way to know which leg was doing the work.

**I: Do you find that useful that kind of feedback?**

MP: Well yes it was reassuring that what I was feeling was actually what was happening rather than just me thinking I was doing something there was proof down there that I was actually doing it.

**I: When you are doing those types of exercises so now you are working on standing up from sitting down what are you thinking about, what do you focus on?**

MP: Just the goal of walking again I guess. That’s what’s always in your head because it’s the one thing that seems furthest away.

**I: So you’re thinking about that long term what this is all for, what you are trying to get to.**

MP: Yes.

**I: Do you focus on anything about the exercise do you think, do you think about what you are doing or do you tend to just?**

MP: Quite intensely I think and that’s what makes it exhausting. I come back from a session down there doing the simplest actions but by the time you get back I think mentally you are exhausted because you are so focused on what you are doing and trying to achieve it. Although they are simple actions a lot of it is very difficult to achieve.

**I: So if you were practicing standing up now for example or standing even what, I’m pushing a little bit, when you say you are thinking about it what would you be thinking about?**

MP: I think it’s difficult not to think back to how it should be, how it should feel and how it’s actually feeling. You are drawing that comparison the whole time.

**I: OK so you are thinking about whether you feel that you are doing it in the way that was normal for you and that was right.**

MP: Yes absolutely, yes.

**I: Do you think about the specifics of the movement at all? How it is you need to move your leg or what you need to do with your leg?**

MP: Yes, you focus, it’s almost a mind game and you are focused on sending a signal which of course is something you’ve never had to do. You don’t know how to do it but you are being asked to do exactly that.

**I: Yes things that are normally automatic you are beginning to think about much more. OK. If you think about the therapists that you’ve been working with, the physios, have you tended to have the same ones or have you seen lots of different people?**

MP: Yes generally the same one.

**I: How would you describe their approach with you when you are practicing.**

MP: Very professional. They really are, they’re very professional. And again it’s almost as though they’ve come straight out of being taught how to do it because they seem to know in advance exactly how they are going to approach each and every exercise which is great because it gives you confidence in them.

**I: Anything about how they talk to you whilst you are practicing exercises? I know the therapists here are brill but is there anything about the way in which they coach you?**

MP: Yes very succinctly.

**I: OK succinctly.**

MP: Yes. It’s a bit like a driving test, they only tell you the things they need you to do, they don’t waffle around it, there’s no chit chat there to confuse you it’s just exactly what you need to do, focus on it, do it, move on.

**I: How do you find that approach?**

MP: Yes I find that the best way.

**I: You’d rather that?**

MP: I’d rather that yes, I mean we have conversations outside of the job that we’re doing just because we’re human beings I think but it doesn’t get in the way.

**I: OK that’s interesting. So has it always been clear what they want you to do?**

MP: Always, yes.

**I: How about feedback, do you they give you feedback? How do you know whether you are doing the right thing or things in the right way?**

MP: I think I know in myself if I’m achieving anything but whenever there are scores or something involved they do come back and tell me the progress and maybe how they viewed something a week earlier and how they view it now.

**I: OK so using quite a clear measure, something that’s got a number to it. What’s your thoughts on that?**

MP: Well that’s obviously a good yard stick, it makes it a lot easier to understand.

**I: Yes. Have you been practicing exercises outside of therapy, have you been doing some?**

MP: I’ve tried. Because it’s the leg there are only so many I can do and I can’t stand up and put weight on it to make it stronger because as soon as I try to get off the bed I have three nurses come running over.

**I: Not yet anyway, not yet.**

MP: No that’s right. So it’s been mainly focused as you will know on the leg and that seems to have come on quite well in that I don’t feel I’m that far away from walking now.

**I: Have you been doing some walking in therapy?**

MP: No we haven’t, we do step round.

**I: OK.**

MP: But not actually walking yet. I think I’m a little way off but pretty close.

**I: Moving towards it.**

MP: If that makes any sense.

**I: Yes it does make sense.**

MP: I feel like I’ve done almost everything else but that and now it’s just a case of mastering that side of it. But if I had some more strength in my arm I’d feel a lot more confident.

**I: The exercises you’ve been doing by yourself outside of therapy are they ones that you’ve worked out for yourself or are they ones that therapy have given you?**

MP: No they’ve given me. They’ve explained what not to do more than most I think, which is good especially with supporting the arm at all times. Don’t let it hang from the shoulder. So I’ve tried to do that and mirror box.

**I: Ah you’ve been doing some mirror box.**

MP: Bit of genius that is isn’t it.

**I: Oh yes. That’s good. So what’s the next bit you are working on do you think in therapy?**

MP: Hopefully the step round.

**I: What do you think you need to do to be able to do that?**

MP: Well when I’m doing it now the only thing that tends to happen is when I put the weight on my numb leg. I don’t seem to have much control over it so I’d like to strengthen my left leg and as a result I did ask on several occasions to go on the little bike they’ve got down there because I thought that action might give it a bit more strength. I think it did, we had a couple of sessions on it and I think that helps. I’m not sure whether that was part of their plan going forward but I just felt anything I could do that I had to work the leg I’d get it somewhere back to where it was before.

**I: That’s good. Sounds good. So I think I’ve got a bit of a sense of this already but how do you feel about the progress you’ve made since you’ve been here?**

MP: Yes I’m reasonably happy. I would like more progress on my arm but my leg is definitely getting there. I’m a lot more confident doing things.

**I: I think it’s never ever as fast as you would like it to be.**

MP: No that’s a fair comment.

**I: Or as we would like it to be. I think that’s really understandable.**

MP: That’s fair comment, especially me I’m not the most patient person in the world.

**I: No it’s tough isn’t it and sometimes you do have to look back to think OK three weeks ago or four weeks ago whatever what were things like then and what are they like now because it’s hard to.**

MP: Yes in my head the first day I was in here I felt I could get up and walk and it was only when somebody in another bed tried and went crashing and took everything with him that I thought oh maybe not. Then the first chance I had to do a step around highlighted to me just how difficult that was going to be.

**I: That’s understandable. How would you know, how would you know if you haven’t been here before. I think that’s human nature isn’t it really. OK so my last couple of questions, this might seem like a bit of an odd one, it flummoxes some people but I’ll ask it anyway.**

MP: OK.

**I: If you were telling a family member who perhaps had come to visit you who you’ve not seen for a while about your therapy sessions, your physio sessions, how would you describe them?**

MP: Again structured I think is the word that comes to mind.

**I: Structured, yes.**

MP: Achievable and hard work, very tiring. They have explained to me that it’s partly the brain repairing that causes the tiredness but it’s a tiredness I’ve never experienced and I’ve run marathons. You get that exhaustion tired but this is not like that. This is a sit in front of the telly with the fire on tired.

**I: Yes really difficult.**

MP: It just knocks you out and you don’t seem to be able to wake up from it without having a sleep.

**I: I’m sure as they’ve said to you that’s really common.**

MP: It’s the concentration.

**I: It’s something that nearly everybody who has had a stroke describes to us the tiredness and it gets better over time but it takes time.**

MP: I did feel stronger now.

**I: It does take time definitely.**

MP: But it’s bizarre getting used to the idea that just sitting in a chair you are working yourself. But two hours sitting in a chair can be exhausting.

**I: Absolutely. Apart from tiredness how would you describe how you feel when you are doing therapy?**

MP: Relieved I think, relieved it’s nice to actually be doing something and working towards the end goal as opposed to just sitting in bed.

**I: Good, OK. That’s perfect, thank you. Is there anything else you would want to tell me about the research you’ve been involved in?**

MP: No just thanks to the guys down the road there. They really are good, even the youngsters that sit in with them the trainees, even they seem to know their role and do it well.

**I: That’s good. I’m going to stop that now.**

**END**
