# Supplementary material for: Understanding implementation of a complex intervention in a stroke rehabilitation research trial: A qualitative evaluation using Normalisation Process Theory
Source: PLoS One. 2023 Sep 8;18(9):e0282612. doi: 10.1371/journal.pone.0282612 (PMC10490858; doi:10.1371/journal.pone.0282612)
Supplement: Attachment — Submitted filename: PLOSOne Response to Reviewers.docx [file pone.0282612.s005.docx]

| FEEDBACK | | RESPONSE |
| --- | --- | --- |
| Reviewer #1 | |  |
| 1 | p. 10: Provide one additional sentence after Table 3 that explains to the reader that you are now presenting the NPT constructs and how they are reflected in participant statements/experiences. | The following sentence has been added immediately after Table 3:  *“Findings are presented below according to each of the four NPT constructs, and how they are represented through participant views and experiences”* |
| 2 | p. 30, line 502: Did you assess context of the participating sites prior to implementing the intervention? If so, highlight it here. | Although there were inclusion criteria for the clusters (Stroke Units), we didn’t assess context specifically in relation to implementation. Understanding “readiness” to change from a service/team point of view, is something that we recommend as a result of this research – but is not something we did from the outset. The following sentence has been added to the discussion, reinforce this point:  *Understanding the readiness of potential research sites to implement the new intervention could inform site selection and/or help to shape a more bespoke approach to implementation support.* |
| 3 | p. 31, line 535: Were you able to match if those patients who did not express concern about the relationship/communication style of the therapist were those patients who attended therapists who were more comfortable adapting to implicit learning and reduce their communication with the patient? I am just wondering if those therapists who found it difficult to limit their communication may not have used implicit learning so much and therefore, these patients did also not comment negatively on a particular communication style? I am not sure if you had any measures in place to check if therapists used implicit learning or not – apart from self-report. | Thank you for this comment – it is an interesting point. We did monitor treatment fidelity through video recording of treatment sessions, which were analysed using a validated framework, by a blind assessor (who wasn’t aware whether the session was from an implicit learning or a control cluster). This data is not included within this paper – it will be reported in the paper describing the pilot cRCT. We haven’t included it here because it would be difficult to tease out specific therapists views (from the focus group) and correlate this with the experiences of patients they treated. Most (if not all) patients were treated by more than one therapist, so this level of analysis wouldn’t be possible. In addition, no patients expressed concern about relationships/communication style - this was universal regardless of who treated them. We haven’t therefore made any changes to the manuscript in relation to this point, but it is something to consider within future research. |
| 4 | p. 32, line 553: the evaluation took part at the end of the trial recruitment phase. What does that mean? Was that before the intervention took place because it was when all sites/participants were recruited? Please clarify. | We have removed the words “recruitment phase”, so it now reads “…at the end of the trial”. Hopefully this clarifies that we conducted the focus groups after all trial interventions had been delivered – as at this point the therapists had experience of implementation. |
| 5 | p. 33 line 580: This is different to what is stated in the online submission form. The authors state that interview transcripts will be made available through the University of Southampton. I am aware this is PLOS One policy, however – would an entire focus group/interview transcript make it easier to identify a site/individual? | The anonymised transcripts will be made available, in line with Uos policy. They are fully anonymised, including a change in labelling of sites/individuals to that which was used in the trial. There is nothing that would enable the site or an individual therapist to be identified. |
| Reviewer #2 | |  |
|  | Title: The authors may wish to consider including the term process evaluation in the title, this to facilitate location of the publication by search engines. But, see later comments re the use of the term process evaluation in this study and manuscript. (m) | We take on board the comments about our use of the term “process evaluation” in this manuscript, particularly given that we have will report qualitative data relating to intervention fidelity in the cRCT manuscript. For clarity, we have therefore removed the term “process evaluation” throughout the manuscript, and replaced this with “qualitative evaluation”. We believe this more accurately describes this particular aspect of the research programme. The title hasn’t therefore been changed. |
|  | Abstract - Methods: The number of stroke units participating in the pilot cCRT should be included in the methods section together with a statement that these units were in the UK/England and whether the units were in a single county or region. Similarly, the number of stroke survivors interviewed should be stated. (M) | The following sentence has been added to the abstract: Participating Stroke Units (n=8) were from the South East/South West regions of the UK.  The methods section already states how many stroke survivors were interviewed (n=19) – so this hasn’t been changed. |
|  | Abstract - Results: This section reports on therapists’ perceptions and actions with no indication of findings from data generated from interviews stroke survivors. The same can be said of the conclusions and contributions to the literature section. If the stroke survivor data contributed to the interpretation of the therapists’ data as part of the process evaluation, then some mention of what those data indicated should be made. (M) | The following statement was already within the results:  Therapists’ concerns that the intervention may impair therapeutic relationships and patient learning were not reflected in how patients experienced it.  We have added to the conclusion:  Insights into the views and experiences of patients (in relation to those complex interventions) could be used to influence and reinforce changes in therapist behaviour. |
|  | Abstract - Conclusions: Please see the comments which follow relating to determining flexibility and rule based cultures. Contributions to the literature: May need some revision based on changes suggested as being necessary for the manuscript to be considered for publication. | We have altered the conclusion (see above) but have not expanded on determining culture, as this would increase the length of the abstract. This has been addressed in the main text (see below).  Contributions to the literature have been revised. We have removed one of the more generic statements, and added one on culture, and one on patient views.   - Team culture, whether flexible or rule based, impacted the process of implementation. Evaluating culture and adapting implementation support to account for this, may be necessary to ensure fidelity. - The views and experiences of research participants (patients) provided valuable insights that could be used to support and sustain the process of implementation. |
|  | Introduction |  |
|  | 1) Lines 67-74. I appreciate the need for brevity but there needs to be some more detail provided in the description of the intervention being piloted. As a minimum, implicit learning as it was defined in the pilot cCRT needs to be explained, this in order to understand how far the data collection methods selected were likely to be effective in enabling understanding participants’ understanding of the intervention and of the implementation of the intervention. Table 3 (page 17) is very helpful but would be easier to interpret with a more detailed description of the nature of the implicit learning intervention.(M) | Thank you for this comment. We had included a brief description of implicit learning within the methods section, rather than in the introduction. In the revised manuscript, we have added further narrative in the introduction:  **[new text]** Implicit learning targets the non-conscious attributes of the motor learning process, i.e. learning without awareness. This differs from standard rehabilitation, which is known to be explicit in nature, i.e. therapists use frequent, detailed, body focussed instructions and feedback during rehabilitation.  **[text within methods section]** The principles of implicit learning, as applied in this research, involved: a) reducing the frequency of verbal instructions during therapy sessions; b) increasing the specificity of feedback, and c) adapting tasks *and* communication to promote an external focus of attention^16^. The intervention was not prescriptive; therapists were asked to adapt their practice for the individual patient(s) they worked with, whilst staying true to these principles.  The treatment guidance will also be added as a supplementary file. |
|  | 2) Lines 91 and 92. The statement ‘here we use implementation science’ is somewhat sweeping. It seems that you are using the terms implementation science and NPT interchangeably. Greater clarity is needed in defining implementation science and your rationale for selecting NPT to underpin the evaluation of implementation in the pilot cCRT. Also, please see comment 5 below re process evaluation.(M) | Thank you – we have removed the words “implementation science” from this sentence, and replaced with “NPT”.  We introduce NPT in the introduction, before describing the study objectives. In the interest of keeping the manuscript as succinct as possible, we have chosen not to add further text on this. |
|  | 3) Line 102. Do you mean principles here? (m) | Yes, this has been corrected. |
|  | 4) Line 100 to 107. Can some indication of content of the 2.5 hour training and perhaps some extracts from the handbook be included as supplementary online material to help readers make sense of the intervention and its evaluation? (m) | The training covered the theory and principles or implicit motor learning, alongside examples for how common rehabilitation activities could be adapted within practice. The text had been modified slightly to add more clarity for this. To avoid adding significant extra words to the manuscript, the training manual will now be added as a supplementary file.  …”training and a handbook containing information on the theory and principles of implicit learning, and examples (with images and video) for how this could be applied in practice. The full treatment guidance is available in Supplementary File 1. |
|  | 5) Line 108. This appears to be the only mention of the term process evaluation. As a minimum this methodology should be explained, ideally in the context of the recent MRC Framework for Developing and Implementing Complex Interventions (Skivington et al, 2021) and the MRC Guidance for Process Evaluation of Complex Interventions (Moore et al (2015). This would set up comment on the choice of qualitative rather than mixed methods in the process evaluation which should be explained here. Generating data as part of a process evaluation on stroke survivor views is most appropriate in my view but please see and consider the above comment relating to the absence of report on how these data contributed to the later interpretation of the therapists’ data.(M) | Thank you for this feedback. We have reframed this manuscript (consistent with our original title) to be a nested qualitative evaluation, and have removed the term process evaluation.  This manuscript will sit alongside a separate one outlining findings from the pilot cRCT, which will include the quantitative data relating to fidelity. We believe this is a clearer reflection on this specific paper.  Comments on stroke survivor data have been responded to separately. |
|  | 6) Line 117 and 118. Please clarify the process used for selection of the therapists and therapy assistants. Lines 128-130 suggest a convenience sample. (m) | Yes, it was a convenience sample - this has been added. |
|  | 7) Lines 135-150. The description of the process of analysis in focus group data is helpful overall but the term semantic codes probably needs explanation and a reference, some readers will not be familiar with the approach. Similarly, I suggest you define latent codes, line 146-147. Providing an example (in a table or text box) of the approach reportedly used to integrate the semantic codes and the NPT driven coding (Lines 142-144) would be very helpful.(M) | Reference has been added, along with the following explanation…. *“allowing identification of the explicit or surface meaning of the data, and describing the content as communicated by the respondents”*  Similarly, in relation to latent codes, a definition and reference was already present, but this has been expanded on:  Where hidden meanings or underlying assumptions were identified, latent codes were applied. This enabled analysis to move beyond a purely descriptive approach^21^, through the identification of ideas or ideologies that could inform interpretation of the descriptive (or semantic) data.  Table 3 provides detail on all codes, and how they map to the NHP constructs/component. |
|  | 8) Line 155 and 159. Interviews with in-patients in the hospital setting can be very different to those conducted in peoples own homes, what consideration was given to the impact of setting on data generated? Presumably the lead researcher also conducted the interviews. What adaptations, if any, were needed to allow the stroke survivor with language difficulties to participate in the interview?(M) | All but two of the interviews were conducted in hospital (numbers have been added to the transcript for clarity). Two were conducted at home for logistical reasons, as these patients had consented to be interviewed but were discharged prior to the interview taking place. We do not therefore feel there was a significant impact of setting that needs taking into consideration.  Ability to follow single stage commands was an inclusion criteria for the cRCT; in reality, by the end of the intervention, all participants that took part in the interview had intact receptive language at a conversational level. There were participants with expressive language impairment – but aside from additional time (and the skills of an interviewer with significant experience working in stroke rehabilitation) we did not need to make any adaptations. We have not made changes to the transcript. |
|  | 9) Line 165 Typo, ‘and’. | Corrected. |
|  | 10) Lines 165-166. The process of reviewing the focus group data against the stroke survivor interview data needs more explanation, this is potentially a complex process but the information provided in the manuscript is insufficient to determine the quality of the analytical processes used; this in turn impacts the confidence which can be placed on the claims made later about integration of these data. It is not clear why data from control group stroke survivors were included as against only intervention group survivors, brief explanation would be beneficial.(M) | Thank you for this comment. We have added additional text to explain the process of analysing the focus groups, and how the two datasets were reviewed together:  Analysis took an experiential orientation ^22^, prioritising how each style of therapy (implicit learning or usual care) was experienced by the participant. Initial coding was predominantly inductive; codes were transferred onto a spreadsheet, with a label identifying the participant and group (intervention or control). Codes were then considered collectively, to interpret meaning and meaningfulness across the dataset. At this stage, connections were also made to the focus group themes; with interview (patient) data being broadly mapped to the focus group (clinician) data. This process of reviewing and comparing the two datasets underwent several iterations, informed by discussion with the wider study team (SD and JM).  Data from the control group was included, where it was relevant to compare the experiences of participants who experienced the two different styles of rehabilitation delivery. A footnote has been added to table 2 to clarify this:  * for the purpose of understanding and describing experiences of the IMPS intervention, only data from the intervention group is used. For the purpose of comparing experiences between the two styles of therapy delivery, data from both intervention and control groups is used. |
|  | 11) Lines 277-291. The term’ handling’ used in the context of post-stroke physiotherapy will be familiar to those working in the field but probably needs definition (as a footnote perhaps) for those not familiar with physiotherapy/occupational therapy or stroke rehabilitation.(m) | The following footnote has been added:  * “handling” is a general term used in neurological rehabilitation to describe the provision of manual support from a therapist(s), during movement. This may be to ensure patient safety, to provide feedback (e.g. sensory), or to guide and facilitate movement. |
|  | 12) Line 438 and elsewhere in the reporting of the findings the differentiation of the teams using the concepts of fixed or rule-based and more flexible or principle based makes sense and is indirectly supported by some of the direct quotations and accompanying explanation. What is not clear is out of the four teams how many fell into each category?(M) | There were 2 teams that adopted a “fixed” view, and 2 that adopted a “flexible” view. This has been added to the main text as (n=2) |
|  | 13) Across the reporting of findings. It was encouraging to read the stroke survivor perceptions/quotations in relation to NPTs coherence and cognitive participation. It would be helpful to hear comment on why the stroke survivors’ comments did not also relate to collective action and reflexive monitoring.(m) | Please see earlier comments – we have increased the use of stroke survivor data throughout the manuscript, including within these sections. |
|  | 14) Lines 477-483. Some interesting issues raised here. Firstly, in terms of process evaluation, fidelity to the trial protocol or an intervention manual is commonly evaluated both qualitatively and qualitatively. It seems in this study the two elements were regarded as separate forms of evaluation and the opportunity to consider and synthesise the findings was not taken; for example comparing fidelity measure outcomes for fixed teams versus flexible teams. Is that the case? Secondly, if ‘trial designs (or trials teams) ‘may need to actively facilitate implementation, particularly monitoring and supporting coherence and cognitive participation’, how do the authors envisage this could occur in a trial context? (M) | Thank you for this feedback. Intervention fidelity was monitored in the pilot trial, with data primarily collected through video recording. As this was a pilot study, we have reported the quantitative fidelity data in a separate paper, alongside findings from the cRCT as a whole.  With regards to activities that support coherence and cognitive participation, we have already gone on to discuss this in the next few paragraphs, but have added a few additional points to this revised version (e.g. flexible training, action learning sets and case reviews etc):  Actively facilitating reflexive activities for teams, for example through case reviews or action learning sets, could also benefit implementation. |
|  | 15) Line 492. Typo ‘e’. | Corrected |
|  | 16) Lines 549-553. The criticism (of NPT) is largely valid but is increasingly being addressed in the literature, not least by May et al. Whilst it is encouraging that stroke survivor interviews were included in the qualitative evaluation, the use of the findings from the interview is very limited in this manuscript which in my view limits the confidence that can be placed in some of the claims made. (M) | We take this on board. We have reviewed the manuscript throughout, integrating more of the patient data (which we had previously intended to include in a separate manuscript), alongside therapist data. We believe this gives a more rounded view, and (alongside the other changes) strengthens the manuscript. |
|  | 17) Re limitations, given that there is no indication of the types of units/services and whether they were from one county or across the UK a limitation may relate to their similarity or difference. Some comment should be made regarding the context of rehabilitation service provision in the participating units. (M) | Additional detail about the setting has been added into the abstract/introduction. In addition, the following line has been added into the limitations:  In this study, participating units were from one geographically defined region (Southern UK); it is not known if or how the findings would differ in other settings, for example those with greater social or cultural diversity. |
|  | 18) Lines 567-569. Some comment on how flexible cultures might be determined and linked to that how change and service improvement might occur in inflexible cultures would be valuable if the findings of this study are to be of value to other researchers. (M) | We have added the following:  To deliver a new intervention, a healthcare organisation (or service) needs to be willing (e.g., committed) and able (e.g., capable) to embrace the required change^37^. Whilst there are theories and tools to aid understanding of readiness for implementation^38, 39^, these are not specific to research contexts. Understanding the readiness of potential research sites to implement a new intervention could inform site selection and/or help to shape a more bespoke approach to implementation support. Developing a trial specific survey to understand a) previous attitudes towards innovation and b) readiness or intention to implement the new trial interventions, may be a useful starting point. Where potentially inflexible cultures are identified, a more tailored programme of support, informed by behaviour change theory, may be required. |
